# New trabecular bone score reference values for children and adolescents according to age and sex and their relationship with body size and fracture incidence

**DOI:** 10.1093/jbmrpl/ziag159

**Published:** 2026-09-21

**Authors:** David R Weber, Halley M Wasserman, Guillaume Gatineau, Heidi Kalkwarf, Michael A Levine, Babette S Zemel

**Affiliations:** The Perelman School of Medicine at the University of Pennsylvania, Department of Pediatrics, Philadelphia, PA 19104, United States; The Children's Hospital of Philadelphia, Department of Pediatrics, Philadelphia PA, 19104, United States; Cincinnati Children's Hospital Medical Center, Department of Pediatrics, Cincinnati, OH 45229, United States; University of Cincinnati, Department of Pediatrics, Cincinnati 45267, United States; Centre of Bone Diseases, Rheumatology Unit, Bone and Joint Department, Lausanne University Hospital, University of Lausanne , 1011 Lausanne, Switzerland; Medimaps Group SA, 1228 Geneva, Switzerland; Cincinnati Children's Hospital Medical Center, Department of Pediatrics, Cincinnati, OH 45229, United States; University of Cincinnati, Department of Pediatrics, Cincinnati 45267, United States; The Perelman School of Medicine at the University of Pennsylvania, Department of Pediatrics, Philadelphia, PA 19104, United States; The Children's Hospital of Philadelphia, Department of Pediatrics, Philadelphia PA, 19104, United States; The Perelman School of Medicine at the University of Pennsylvania, Department of Pediatrics, Philadelphia, PA 19104, United States; The Children's Hospital of Philadelphia, Department of Pediatrics, Philadelphia PA, 19104, United States

**Keywords:** trabecular bone score, DXA, BMD, osteoporosis, fracture

## Abstract

Trabecular bone score (TBS) is a textural index derived from LS DXA images that provides an indirect measure of trabecular microarchitecture. We published the first pediatric TBS reference dataset in 2022. The TBS software algorithm was subsequently modified by the manufacturer to improve soft-tissue thickness correction and precision. In this study, we aimed to develop new pediatric TBS reference ranges using the updated TBS Insight v4.0 software, determine if these differed from those previously published, and investigate the associations between TBS, age, sex, anthropometrics, and incident fracture in healthy children. TBS data from 9898 LS scans in healthy US children from the Bone Mineral Density in Childhood Study were used to generate reference ranges for youth 5-20 yr by age and sex. The new TBS L1-L4 reference ranges differed from those generated using the prior software version, overall mean TBS L1-L4 1.35 ± 0.10 vs 1.33 ± 0.12, *p* < .0001. Precision was numerically improved; percent coefficient of variation of 2.43% (95% CI: 2.18-2.74) compared to 2.87% (95% CI: 2.58-3.23). TBS values increased with age and differed by sex. There were nonlinear associations between TBS Z-scores and height- and BMI Z-scores, peaking at *r* = 0.43 at 12 yr in females and 0.34 at 14 yr in males for height, and *r* = 0.38 at 15 yr in females and 0.31 at 15 yr in males for BMI (*p* < .0001 for all). TBS Z-scores were not associated with risk of incident low-to-moderate impact fracture over 6 yr (hazard ratio: 0.97, 95% CI: 0.84-1.12). In summary, the new pediatric TBS reference ranges differed from those previously published and should be used for scans obtained using TBS Insight v4.0 software. TBS values increased during childhood and were associated with height and BMI but not incident fracture in this cohort of healthy children. Future studies of TBS in children at risk of skeletal fragility are needed.

## Introduction

Standard DXA techniques provide estimates of BMC and areal BMD (aBMD). BMC and aBMD are important components of bone strength, and aBMD associates with risk of incident fracture in cohorts of healthy children and those with disorders affecting bone strength.^1^^,^^2^ Clinically, however, many children with high-level risk factors for impaired bone health sustain fragility fractures despite aBMD levels above commonly used thresholds to define low bone density.^3^ This discrepancy supports the need for additional screening tools beyond DXA to identify children at risk for skeletal fragility.

Trabecular bone score (TBS) is a textural index derived secondarily from LS DXA images that provides an indirect measure of trabecular microarchitecture,^4^ a contributor to bone strength.^5^ TBS predicts fracture independently from aBMD by DXA in adults,^6–8^ suggesting that TBS captures a clinically important skeletal characteristic. The TBS software quantifies gray-level variations from pixel to pixel in DXA LS images and does not require an additional procedure or further radiation exposure, making it an appealing technique for children.^9^ Studies reporting TBS data from various pediatric cohorts have been published,^10–17^ but interpretation of findings is challenging because of changes in the TBS algorithm across different software versions. Furthermore, TBS has not yet been adopted for clinical use in children because of limited reference data and lack of regulatory approval for clinical use.

We published the first pediatric TBS reference data in 2022, utilizing data from the Bone Mineral Density in Childhood Study (BMDCS).^18^ The TBS data were obtained using a pre-release version of TBS Insight v4.0 software. In the years since the publication of the first pediatric TBS reference dataset, substantial modifications to the TBS algorithm were made by the manufacturer prior to software release. The revised algorithm incorporated a larger number of measurements acquired at low tissue thickness/noise levels, as well as measurements obtained using scan modes designed for individuals with low soft-tissue thickness levels (including THIN mode for GE and FAST ARRAY mode for Hologic scanners, respectively). The intent of these enhancements was to refine the calibration and standardization of TBS relative to the average population, resulting in better-harmonized TBS values that are more consistent when transitioning from one age, body habitus, or acquisition regimen to another.

The primary objectives of this project were to develop updated pediatric reference ranges for TBS using data from the new TBS Insight v4.0 software and to determine whether the new TBS reference data differed from those previously published using the prior software version. The effects of age, sex, parent-reported population ancestry, and anthropometrics on TBS were investigated, and the precision of TBS and stability (tracking) of TBS Z-scores over time were determined. Finally, the relationships between TBS Z-score, aBMD Z-score, and fracture incidence in the BMDCS cohort of healthy children were investigated.

## Materials and methods

### Study sample

Existing data from BMDCS were utilized to generate pediatric TBS reference ranges. BMDCS was a multicenter, longitudinal study of bone accrual in 2014 healthy children and adolescents. Healthy, typically developing individuals aged 5-19 yr were enrolled at 5 clinical centers in the US and followed annually for up to 6 yr (7 visits), as previously described.^19^^,^^20^ Exclusion criteria included pre-term birth <37 wk gestational age, low birth weight <2300 g, BMI <3rd or >97th percentile for age and sex, premature or delayed pubertal onset, medical conditions or medication exposures known to impair bone health, recurrent long bone fractures (≥2 by age 10 yr, ≥3 fractures by age 19 yr), and the presence of skeletal deformity or indwelling surgical hardware. DXA scans, anthropometric measurements, and subject questionnaires were completed at baseline and all follow-up visits. Data collection occurred between 2002 and 2009. The Institutional Review Board for Human Subjects Protection at each clinical center approved the study protocol. Written informed consent was provided by participants 18 yr of age or older. Participants less than 18 yr of age provided assent, and a parent/legal guardian provided written informed consent. Eligible TBS scans from the full BMDCS cohort (age 5-23.4 yr) were used to generate TBS reference data, Figure S1. Analyses to investigate associations between TBS and age, sex, pubertal stage, anthropometrics, and fracture incidence were limited to data from the pediatric age range (5-20 yr).

### Dual energy X-ray absorptiometry

Posterior-anterior LS (L1-L4) DXA scans were acquired on Hologic (Hologic Inc.) densitometers (models QDR4500-A, QDR4500-W, and Delphi-A) as described previously.^19^^,^^20^ Scans were acquired in fast array mode with participants positioned according to manufacturer guidelines and analyzed for areal BMD (aBMD) at the DXA Core Laboratory at the University of California, San Francisco. Abdominal soft tissue thickness was automatically determined as a feature of the scan analysis software. Hologic software version 12.3 was used for analysis of baseline measurements and Apex 2.1 was used for follow-up measurements using the compare feature. A second spine scan was obtained on the same scanner for 155 participants at the first study visit to allow calculation of precision error. Subjects were taken off the table and then repositioned prior to obtaining the second scan.

### Trabecular bone score

TBS was ascertained as a secondary analysis of existing L1-L4 DXA scans. Scans were centrally analyzed using TBS iNsight software version 4.0 (Medimaps) by the Interdisciplinary Center for Bone Diseases.

Subsequent modifications to the pre-release version 4.0 expanded the validated tissue-thickness range down to 7 cm, and up to 30 cm. This expansion covered the range observed in the pediatric sample (8.8-28.3 cm).^18^ The validity at low tissue thicknesses of the new version 4.0 has previously been reported in the OsteoLaus study, which demonstrated low residual associations with soft-tissue measures, supporting improved robustness to soft-tissue variation.^21^

### Other variables

Height was measured in triplicate by stadiometer and weight was measured in duplicate by a digital electronic scale. BMI and height-for-age Z-scores (HAZ) were calculated using CDC reference data.^22^ Sexual maturation was assessed by physical exam by a clinician with expertise in pubertal assessment; breast development (girls) and testicular volume (boys) were evaluated and reported according to the criteria of Tanner.^23^ Sex assigned at birth and population ancestry were obtained by participant/guardian self-report. Fracture history including anatomical location, cause, and energy (low, moderate, high, and unknown) in the year prior to study visits was collected by questionnaire. Low energy fractures were those resulting from a low-impact collision or a fall from standing height or less; medium energy fractures were those that occurred with sports/physical education class, leisure activities (eg, cycling) or fighting.^24^

### Statistical methods

Sex-specific reference ranges for TBS L1-L4, for each individual vertebral body (TBS L1, L2, L3, L4) and other combinations of vertebral bodies [TBS L1-L2; L1-L3; L1(L2)L3; L1(L2, L3)L4; L1(L2)L3, L4; L1, L2(L3)L4; L2-L4; L2(L3)L4; L3-L4; where vertebral bodies in parenthesis indicate excluded variables] were generated using the LMS (Lambda, Mu, and Sigma) function in the GAMLSS (Generalized Additive Models for Location Scale and Shape) package in R (version 4.4.0 (2024-04-24), R Foundation for Statistical Computing, Vienna, Austria).^25^ TBS for vertebral body combinations of 2 or 3 vertebral bodies were determined as the average of included individual vertebral bodies. Sample weights were applied in the execution of the LMS function so that the representation of race and ethnicity groups was similar to the 2017-2018 National Health and Nutrition Examination Survey: 49% non-Hispanic White; 13% non-Hispanic Black; 26% Hispanic, 5% Asian, 7% Pacific Islander, Native American and Other,^26^ using the same methodology as the most recent BMDCS bone density reference dataset.^1^ The following sample weights were used: White: 10; Black: 6; Hispanic: 15; Asian: 7; Other: 12.

The age-specific LMS values represent the power for the Box-Cox transformation (*L*), the median (*M*), and the SD (*S*). These values are used to create reference percentiles using eqn (1):


(1) \begin{equation*} \mathrm{TBS}\ \mathrm{centile}=M\ {\left(1+ LSZ\right)}^{1/L} \end{equation*}

where *Z* is the *Z*-score that corresponds to a given percentile, for example, a *Z*-score of zero is the 50th centile. The LMS values are also used to generate individual *Z*-scores were using eqn (2): 


(2) \begin{equation*} Z=\left[\left({\left(X/M\right)}^L\right)-1\right]/L\bullet S \end{equation*}

where *X* is the measured TBS value. TBS values from all ages were used to generate the curves. However, due to the small number of individuals older than 20 yr, reference ranges were restricted to ages 5-20 yr.

Statistical analyses were performed using Stata v.19.0 (StataCorp LLC). Data distributions for continuous variables were assessed graphically and tested for skewness prior to analysis. Parametric data were expressed as mean ± SD, nonparametric data as median (IQR). Precision was calculated as root mean square error (RMSE) and percent coefficient of variation (%CV) using duplicate scans from the precision sub-study using the approach recommended by the International Society for Clinical Densitometry.^27^^,^^28^ The least significant change was calculated as 2.77 × RMSE. These estimates were determined for the entire age range, and separately for preteens (6-10 yr), younger teens (10-13 yr), and older teens (14-16 yr). Estimates of the 95% CIs for RMSE, %CV, and LSC were calculated using chi-square confidence limits for the within-subject variance, based on paired replicate measurements, with *n* − 1 degrees of freedom.^29^ Stability (tracking) of TBS Z-scores over time was assessed by determining the correlation of baseline TBS Z-scores with new and previously published TBS reference values, both overall and stratified by age category. Potential associations between height, BMI, and TBS were investigated by calculating the Pearson correlation coefficients between respective Z-scores stratified by sex and age. Cox proportional hazards models were used to test for associations between TBS and BMD Z-scores and incident fractures in the year following TBS assessment. High energy fractures were excluded. Base models included TBS Z-scores only; adjusted models included both TBS and age-group, pubertal stage, growth velocity, or aBMD to account for known associations between these variables and fracture risk.^1^^,^^30^^,^^31^

## Results

 The BMDCS enrolled 2014 individuals with 10 722 person visits. Of these, there were 9898 evaluable TBS results drawn from 2008 study participants (1018 female) used to generate the reference data, Figure S1. Missing/excluded scans were due to participants excluded from completing study visits due to developing an exclusion criterion (eg, recent glucocorticoid exposure), *n* = 634, missing or inadequate (eg, improper positioning or artifact) LS DXA scan, *n* = 32, or missing or inadequate (eg, technical error where TBS could not be calculated from image) TBS scan, *n* = 158. Descriptive statistics of the study sample used to generate TBS reference data are provided in Table 1.

**Table 1:** Characteristics of study sample used to generate new trabecular bone score pediatric reference ranges.

**Descriptive statistics**	**All**	**Males**	**Females**
***N***	9898	4838	5060
**Age, median (IQR)**	13.6 (10.4, 16.7)^a^	13.8 (10.5, 16.9)	13.3 (10.4, 16.3)
**Race/ethnicity**			
**American Indian or Alaskan**	14 (0.1%)	5 (0.1%)	9 (0.2%)
**Asian or Pacific Islander**	693 (7.0%)	352 (7.3%)	341 (6.7%)
**Black**	2209 (22.3%)	1093 (22.6%)	1116 (22.1%)
**Hispanic**	1658 (16.8%)	778 (16.1%)	880 (17.4%)
**White**	4750 (48.0%)	2327 (48.1%)	2423 (47.9%)
**Other, mixed, or unknown**	588 (5.9%)	283 (5.8%)	291 (5.8%)
**Height *Z*-score, median (IQR)**	0.17 (−0.43, 0.75)	0.19 (−0.42, 0.73)	0.15 (−0.44, 0.77)
**BMI *Z*-score, median (IQR)**	0.36 (−0.28, 0.95)	0.29 (−0.33, 0.93)	0.41 (−0.21, 0.96)
**Weight status**			
**Healthy weight**^b^	3221 (83.1%)	1513 (84.8%)	1708 (81.6%)
**Overweight**^c^	84 (2.2%)	37 (2.1%)	47 (2.2%)
**Obese**^d^	572 (14.8%)	234 (13.1%)	338 (16.1%)
**TBS-L1L4, median (IQR)**	1.350 (1.272, 1.425)	1.349 (1.277, 1.423)	1.352 (1.265, 1.427)

a Median (IQR), all such values.

b BMI <85th percentile for age and sex.

c BMI between 85th and 95th percentile for age and sex.

d BMI ≥95th percentile for age and sex.

### TBS reference ranges

Age and sex specific reference ranges showing the median and ±1 and ±2 SDs for TBS L1-L4 are presented in Figure 1; reference ranges for other L1-L4 combinations and for each individual LS vertebral body are shown in Figures S2 and S3. Individual *L*, *M*, and *S* values for calculating *Z*-scores by age and sex using eqn (2) for TBS L1-L4 are presented in Table 2. Tables that allow for calculation of each individual LS vertebrae and other LS vertebral combinations are provided in Table S1. Expanded tables for all TBS outcomes for calculation of more precise TBS Z-scores by year to the 10th decimal place are provided in Table S2.

**Figure 1:**
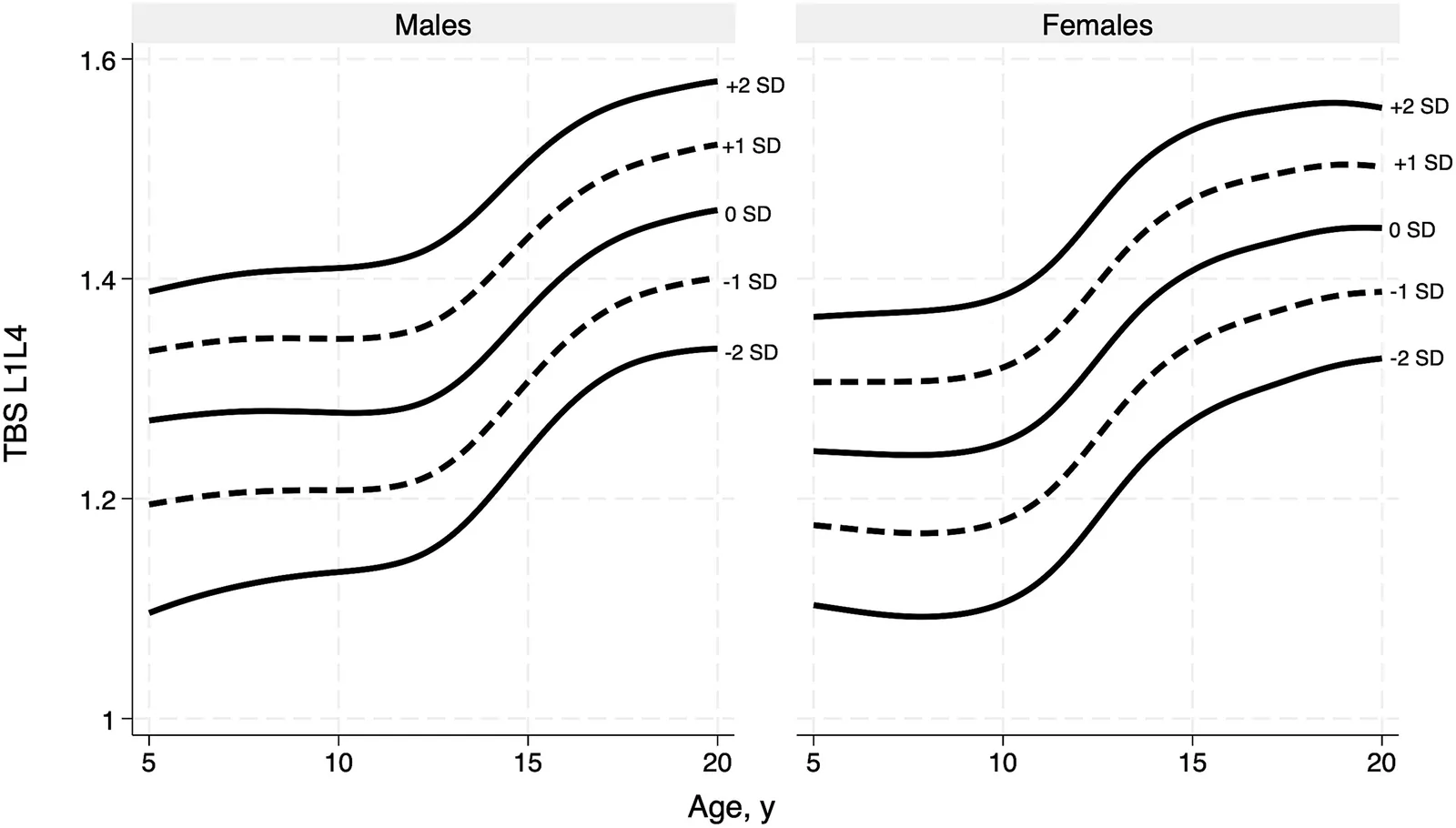
Trabecular bone score reference curves (L1-L4 vertebral bodies) for males (A) and females (B) 5-20 yr of age.

**Table 2:** LMS values and reference ranges (median, ±1 and ±2 SDs) for L1-L4 trabecular bone score for children ages 5-20 years.

	**Males**							**Females**						
**Age (yr)**	***L***	***S***	**−2 SD**	**−1 SD**	**0 SD (*M*)**	**1 SD**	**2 SD**	***L***	***S***	**−2 SD**	**−1 SD**	**0 SD (*M*)**	**1 SD**	**2 SD**
**5.0**-**5.9**	4.196	0.054	1.102	1.198	1.273	1.337	1.392	2.254	0.053	1.101	1.174	1.242	1.306	1.366
**6.0**-**6.9**	3.621	0.054	1.113	1.202	1.277	1.342	1.399	2.165	0.054	1.096	1.171	1.241	1.306	1.368
**7.0**-**7.9**	3.078	0.054	1.121	1.206	1.279	1.345	1.405	2.070	0.055	1.093	1.169	1.240	1.307	1.370
**8.0**-**8.9**	2.572	0.054	1.128	1.207	1.280	1.346	1.408	1.968	0.056	1.093	1.169	1.241	1.308	1.373
**9.0**-**9.9**	2.107	0.054	1.132	1.208	1.279	1.346	1.409	1.868	0.056	1.099	1.175	1.246	1.314	1.379
**10.0**-**10.9**	1.674	0.054	1.135	1.208	1.278	1.346	1.411	1.781	0.055	1.114	1.188	1.259	1.328	1.393
**11.0**-**11.9**	1.273	0.054	1.141	1.211	1.281	1.349	1.417	1.716	0.054	1.142	1.216	1.287	1.355	1.421
**12.0**-**12.9**	0.921	0.053	1.155	1.223	1.292	1.360	1.430	1.677	0.052	1.183	1.257	1.327	1.396	1.462
**13.0**-**13.9**	0.636	0.052	1.183	1.249	1.317	1.385	1.455	1.665	0.050	1.226	1.298	1.368	1.435	1.500
**14.0**-**14.9**	0.451	0.049	1.223	1.287	1.352	1.420	1.489	1.680	0.048	1.259	1.329	1.397	1.463	1.526
**15.0**-**15.9**	0.398	0.046	1.264	1.326	1.389	1.454	1.521	1.718	0.046	1.281	1.350	1.416	1.480	1.542
**16.0-16.9**	0.500	0.044	1.297	1.357	1.419	1.481	1.545	1.776	0.045	1.296	1.363	1.428	1.490	1.551
**17.0**-**17.9**	0.762	0.042	1.319	1.379	1.439	1.499	1.561	1.846	0.043	1.308	1.373	1.437	1.497	1.556
**18.0**-**18.9**	1.157	0.041	1.330	1.391	1.451	1.511	1.570	1.923	0.042	1.319	1.383	1.444	1.503	1.560
**19.0**-**19.9**	1.630	0.041	1.336	1.398	1.459	1.519	1.577	1.998	0.040	1.325	1.387	1.447	1.503	1.558

The new TBS L1-L4 values differed significantly from those previously published using the prior software version (overall mean TBS L1-L4: 1.35 ± 0.10 vs 1.33 ± 0.12 *p* < .0001), Figure 2. Using the new TBS L1-L4 values, we calculated Z-scores using the previously published reference range and the new reference ranges presented here. The magnitude of the difference in L1-L4 TBS Z-scores calculated from the two reference datasets was greatest in younger children 5-10 yr of age, mean difference (new vs prior) of −0.41 ± 0.15, *p* < .0001, compared to those 10-15 yr, mean difference of −0.25 ± 0.25, *p* < .0001, and those 15-20 yr, mean difference +0.07 ± 0.23, *p* < .0001. TBS values were lower in subjects reporting Asian racial group compared to the overall sample [mean difference −0.18 (95% CI: −0.31 to −0.05), *p* = .009], no other racial groups differed significantly from the sample mean.

**Figure 2:**
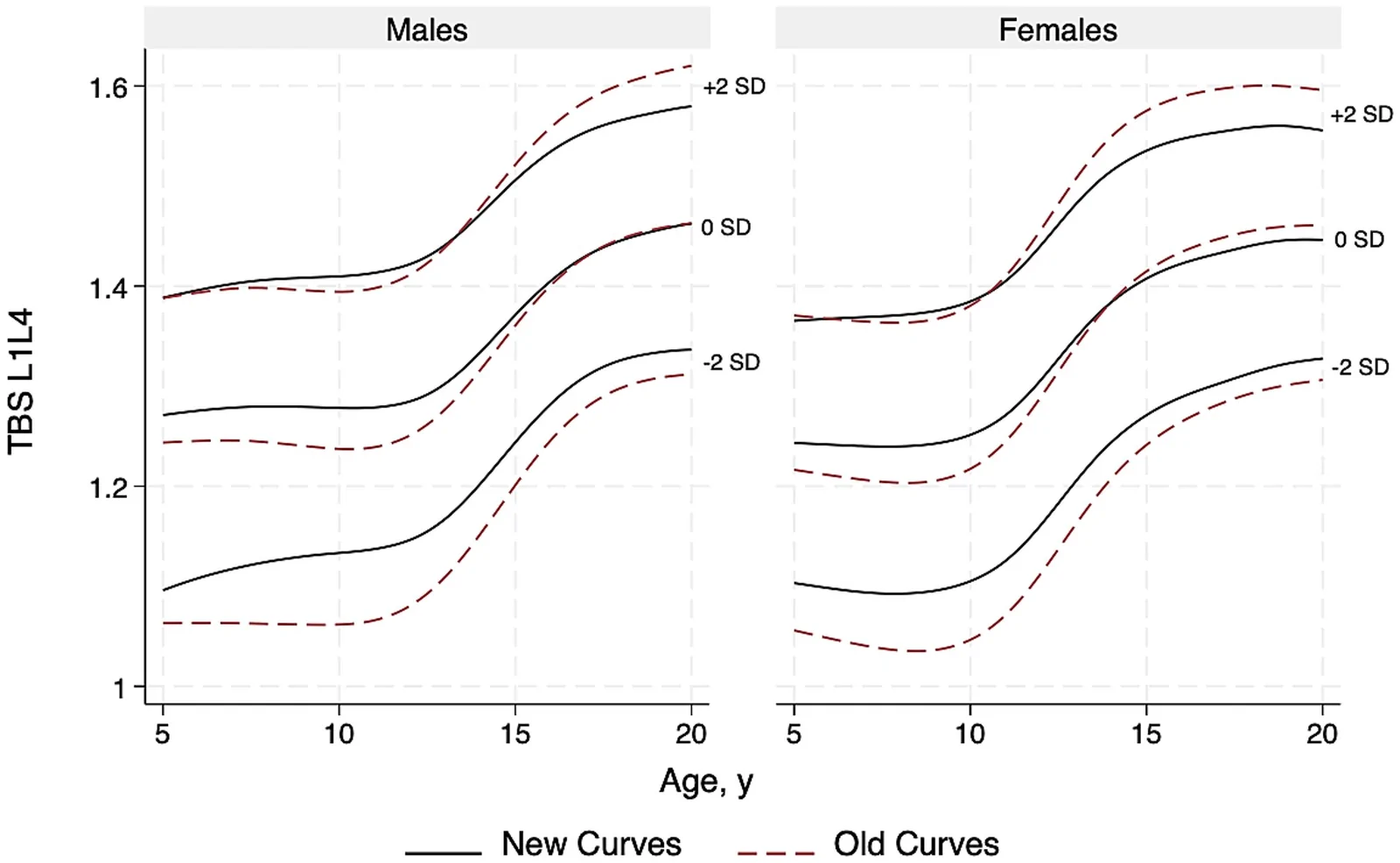
Comparison of new trabecular bone score (TBS) reference curves (L1-L4 vertebral bodies) to previously published curves. New reference curves differed significantly from previously published curves,^18^ overall mean TBS L1-L4: 1.35 ± 0.10 vs 1.33 ± 0.12, *p* < .0001.

### Precision and tracking

The overall precision for the new TBS L1-L4 values was numerically improved compared to previously published values, the %CV was 2.43% (95% CI: 2.18-2.74) compared to 2.87% (95% CI: 2.58-3.23), Table 3. The improvement in precision was greatest in the youngest 6- to 9-yr-old age group [2.83% (95% CI: 2.35-3.55) vs 3.43% (95% CI: 2.86-4.31). It is also noteworthy that the precision was best when TBS was determined for all 4 vertebrae (L1-L4) and worsened when fewer vertebrae were used to estimate precision [eg, L1 %CV = 4.54% (95% CI: 4.08-5.11) vs L1-L3 %CV = 2.69% (95% CI: 2.42-3.03)]. Precision values for TBS outcomes for other vertebral combinations and individual vertebral bodies are provided in Table S3.

**Table 3:** Precision of L1-L4 trabecular bone score (TBS) in children 5-20 years.

	**New TBS values for TBS Insight v4.0**			**Previously published TBS values for pre-release TBS Insight v4.0** ^a^		
	**RMSE**	**%CV**	**LSC**	**RMSE**	**%CV**	**LSC**
**All ages (*n* = 152 154)** ^b^	0.031 (0.028, 0.035)^c^	2.43 (2.18, 2.74)	0.09 (0.08, 0.10)	0.037 (0.033, 0.042)	2.87 (2.58, 3.23)	0.10 (0.09, 0.11)
**Age 6**-**9 yr (*n* = 47**, **49)**	0.035 (0.029, 0.044)	2.83 (2.35, 3.55)	0.10 (0.08, 0.12)	0.042 (0.035, 0.053)	3.43 (2.86, 4.31)	0.12 (0.10, 0.15)
**Age 10**-**13 yr (*n* = 54**, **54)**	0.033 (0.028, 0.041)	2.59 (2.18, 3.19)	0.09 (0.08, 0.11)	0.040 (0.034, 0.049)	3.11 (2.61, 3.83)	0.11 (0.09, 0.13)
**Age 14**-**16 yr (*n* = 51**, **51)**	0.024 (0.020, 0.030)	1.77 (1.48.2.20)	0.07 (0.06, 0.09)	0.029 (0.024, 0.036)	2.05 (1.71, 2.54)	0.08 (0.07, 0.10)

Abbreviations: %CV, percent coefficient of variation; LSC, least significant change; RMSE, root mean square error; yr, year. 95% CI shown in parenthesis.

a Kalkwarf HJ, Shepherd JA, Hans D, Gonzalez Rodriguez E, Kindler JM, Lappe JM, et al. Trabecular bone score reference values for children and adolescents according to age, sex, and ancestry. *J Bone Miner Res*. Apr 2022;37(4):776-85.

b Sample size for new and previously published TBS data, respectively.

c 95% CI, all such values.

Tracking correlations for TBS L1-L4 Z-scores from baseline to year 6 were numerically lower, but within the 95% CI for those previously reported, Table 4. For L1-L4 TBS Z-scores, the tracking correlation for all ages was 0.44 (95% CI: 0.39-0.49), compared to the previously published tracking correlation of 0.47. Tracking correlations were generally greater in older vs younger children, and when TBS was determined for multiple vs single vertebrae.

**Table 4:** Tracking of trabecular bone score (TBS) over six years in children 5-19 years at baseline visit.

**New TBS values for TBS Insight v4.0**												
	**All ages**			**Ages <10 yr**			**Ages 10**-**15 yr**			**Ages >15 yr**		
**TBS**	***n***	**R**	**95% CI**	***n***	**R**	**95% CI**	***n***	**R**	**95% CI**	***n***	**R**	**95% CI**
**L1**-**L4**	934	0.44	[0.39-0.49]	375	0.39	[0.30-0.48]	462	0.44	[0.36-0.51]	97	0.68	[0.55-0.77]
**L1**-**L3**	936	0.45	[0.40-0.50]	376	0.38	[0.29-0.47]	463	0.46	[0.39-0.53]	97	0.68	[0.56-0.77]
**L2**-**L4**	935	0.44	[0.38-0.49]	375	0.39	[0.30-0.47]	463	0.43	[0.35-0.50]	97	0.64	[0.51-0.75]
**L1**-**L2**	939	0.40	[0.35-0.46]	376	0.31	[0.22-0.40]	466	0.43	[0.35-0.50]	97	0.67	[0.54-0.77]
**L2**-**L3**	937	0.45	[0.40-0.50]	376	0.38	[0.29-0.47]	464	0.47	[0.40-0.54]	97	0.61	[0.47-0.72]
**L3**-**L4**	935	0.38	[0.33-0.44]	375	0.33	[0.24-0.42]	463	0.37	[0.29-0.44]	97	0.63	[0.49-0.73]
**L1**	940	0.34	[0.29-0.40]	376	0.25	[0.16-0.35]	467	0.35	[0.27-0.43]	97	0.65	[0.51-0.75]
**L2**	940	0.36	[0.31-0.42]	376	0.27	[0.18-0.36]	467	0.41	[0.33-0.48]	97	0.53	[0.37-0.66]
**L3**	940	0.40	[0.34-0.45]	376	0.34	[0.25-0.43]	467	0.41	[0.34-0.49]	97	0.58	[0.43-0.70]
**L4**	940	0.28	[0.22-0.33]	376	0.19	[0.09-0.29]	467	0.27	[0.18-0.35]	97	0.61	[0.47-0.72]

R, Pearson correlation coefficient.

### Impact of age, sex, and puberty on TBS

The overall trajectory for TBS L1-L4 from 5 to 20 yr of age was notable for stability during early childhood, a rapid rise during early adolescence in proximity to the time of the pubertal growth spurt, and a leveling off in the older teenage years. A similar pattern was apparent for TBS of individual and other combinations of vertebral bodies (Figures S2 and S3). Sex differences in TBS existed, and were age dependent, Figure 3A. Mean TBS values were higher in males from ages 5-10 yr (males: 1.27 ± 0.07 vs females: 1.24 ± 0.07, *p* < .0001), and then higher in females from age 11 to 17 yr (males: 1.34 ± 0.08 vs females: 1.36 ± 0.09, *p* < .0001). Between ages 18 and 20 yr, the mean sex difference in TBS values was statistically significant but numerically very small (males: 1.45 ± 0.06 vs females: 1.44 ± 0.06, *p* < .03).

**Figure 3:**
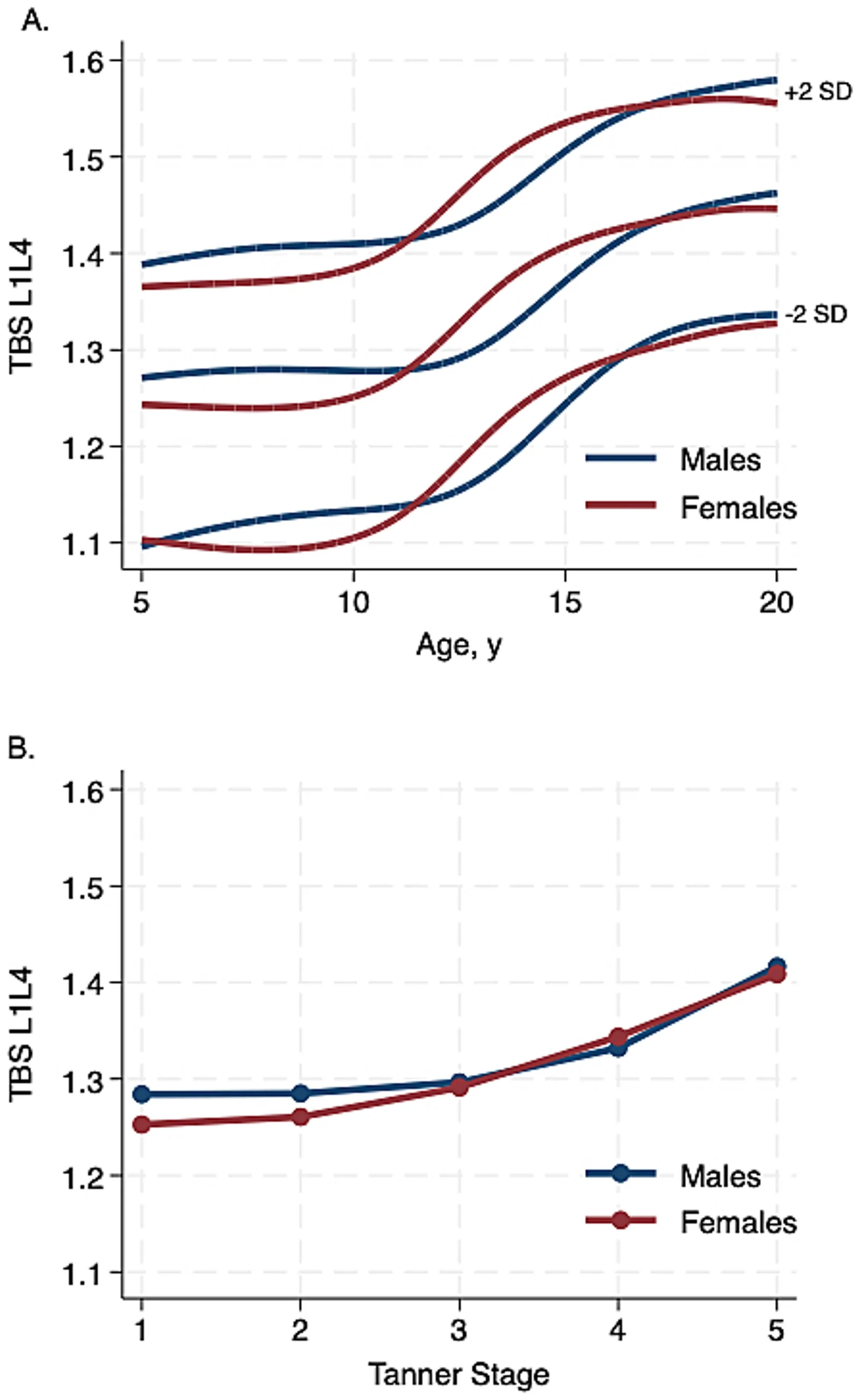
Sex differences in reference curves (L1-L4 vertebral bodies) for children 5-20 yr of age. (A) by chronological age and (B) by Tanner stage.

TBS L1-L4 values increased with Tanner stage, and sex differences varied by Tanner stage, Figure 3B. Among males, TBS L1-L4 was significantly higher with each increasing Tanner stage. Among girls, TBS L1-L4 was not significantly different in Tanner stages 1 and 2, but TBS L1-L4 was significantly greater with each advancing Tanner stage 3 through 5. In Tanner stages 1 and 2, males had higher TBS values than females, and the differences were negligible in Tanner stages 3 through 5.

### Associations between TBS, height, and BMI

The correlations between TBS L1-L4 *Z*-scores and height *Z*-scores exhibited a non-linear age pattern with a peak at age 14 yr in boys (*r* = 0.34) and age 12 yr in girls (*r* = 0.43), and declining thereafter, Figure 4. The correlations between TBS L1-L4 Z-scores and BMI Z-scores increased with age in both boys and girls. However, in boys, the correlation peaked at age 15 yr (*r* = 0.31) and then declined, whereas in girls the correlations increased from about age 5 to 14 yr and remained high thereafter.

**Figure 4:**
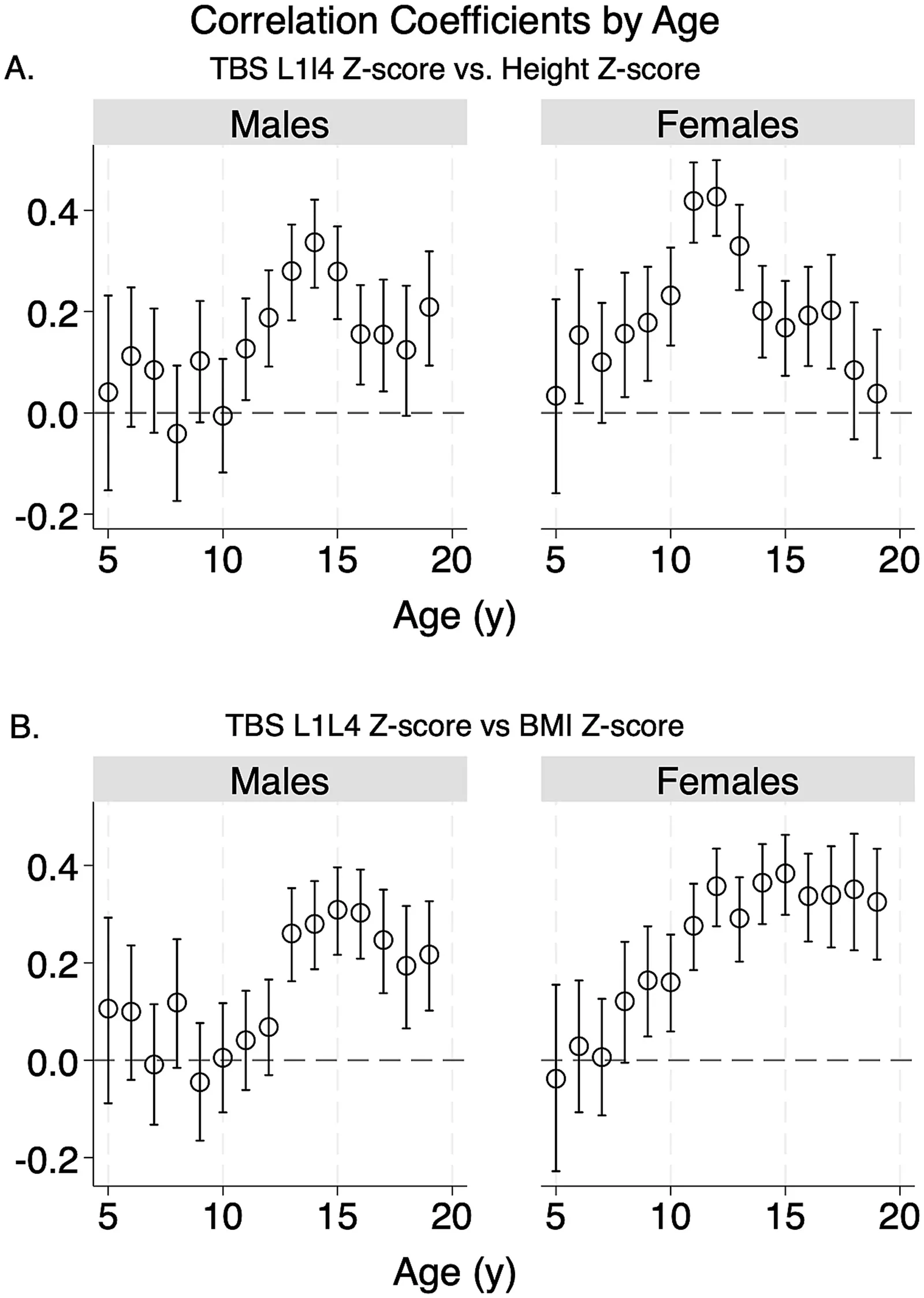
Correlations between L1-L4 trabecular bone score (TBS) Z-score and (A) height- and (B) BMI Z-scores in males and females 5-20 yr of age. Pearson correlation coefficients with 95% CI shown each year of age.

### Fracture incidence

There were 261 fractures over the 6 yr of observation in 218 children 5-20 yr of age (Table S4). The forearm/wrist/elbow was the most frequent fracture location (34.9% of fractures) and sports participation was the most common reason for fracture (48.7%). High energy fractures occurred in 35 instances and these were excluded, resulting in 226 fractures among 190 individuals used in the analysis. TBS L1-L4 Z-score was not associated with risk of prospective low and moderate energy fracture over 6 yr (HR: 0.98, 95% CI: 0.84-1.14), Table S5. The absence of an association between TBS L1-L4 Z-score and fracture persisted when age group, pubertal stage, growth velocity or aBMD (LS, subtotal body, TH, FN, distal 1/3 radius, spine bone mineral apparent density, or ultra-distal radius) *Z*-score were included as independent variables. Further sensitivity analysis considering TBS *Z*-score as a categorical variable did not show a difference in fracture risk by quintile of TBS Z-score.

## Discussion

We present new reference ranges for TBS in children using the recently updated TBS Insight v4.0 software for Hologic densitometers. There were significant differences between the new pediatric TBS values for BMDCS children presented here and those previously published using the pre-release TBS Insight software version 4.0.^18^ Investigators and clinicians interested in using normative pediatric TBS reference data must be aware of these differences and use the appropriate reference dataset that aligns with the software version used to calculate TBS. Existing and future TBS analyses applying the 2022 TBS reference data to scans analyzed using the pre-release version of the TBS Insight v4.0 software remain valid. The new reference data set is more comprehensive than the prior version and now includes TBS values for individual L1-L4 vertebrae, and all combinations of lumbar vertebral bodies. These additional data extend utility by allowing for TBS to be determined in individuals with artifacts or compression fractures of vertebral bodies that prohibit calculation of total L1-L4 TBS. The precision of TBS values from the revised software version was numerically improved compared to the previously published values, which may reflect changes in the algorithm to more accurately account for the influence of soft-tissue thickness on LS DXA image texture, particularly within the lower thickness range. Such effects may be especially relevant in pediatric populations and lean individuals, in whom lower soft-tissue thickness values are more frequently encountered. Improved precision at the lower end of the thickness spectrum may also enhance longitudinal interpretability, where small changes over time could otherwise be obscured by measurement noise.

Relationships between TBS and age, sex, pubertal status, height, and BMI were similar to those reported previously.^18^ TBS values increased across the pediatric age range with the greatest rise occurring during the pubertal growth spurt, aligning with expected gains in bone density and strength during development.^32^ The trajectory of TBS development over time differed by sex, necessitating the use of sex-specific reference data when interpreting TBS values in children. Correlations between TBS and height in both males and females peaked around the time of the pubertal growth spurt and then declined. The strength of the correlation between BMI and TBS increased throughout the pubertal years in both sexes, but then appeared to diverge, remaining stable in females, but declining in males. aBMD has also been shown to have nuanced associations with height and BMI in children,^33^^,^^34^ supporting the need for ongoing study of the impact of growth, body size, and body composition on the acquisition of bone density and strength. TBS values were lower in individuals self-reporting as Asian racial group compared to the overall pediatric sample; however, this finding should be interpreted cautiously given the relatively small prevalence of children reporting Asian racial group in this cohort. There was no difference in TBS between those reporting as black vs non-black racial group, which is consistent with what was reported in our prior study.^18^

Studies in older adults have consistently found that TBS predicts fracture independently of BMD and FRAX.^35^^,^^36^ Most of these studies were conducted in older adults and used “major osteoporotic fracture” (MOF, including spine, hip, forearm, or humerus) as the fracture outcome. However, at least one study found that TBS was associated with risk of fracture at any skeletal site.^37^ Studies conducted in younger adults have reported mixed findings. TBS was associated with risk of fracture in a cohort of pre-menopausal women, however the association was no longer significant after adjusting for aBMD.^38^ Fracture risk was significantly associated with aBMD, but not TBS, in a study of conducted in 2799 individuals aged 20-39 yr.^39^

We did not find a significant association between TBS and incident fracture risk in this cohort of healthy children, even after adjusting for known risk factors, including aBMD, age, sex, pubertal stage, and growth rate. Fracture patterns and mechanisms in healthy children are different than in older adults. Fractures of the distal forearm, hands and feet occurring from injuries during sport or active play predominate, whereas hip and spine fractures are rare.^40^ It is possible that transient deficiencies in cortical bone that arise during physiologic bone growth and modeling are more important contributors to typical childhood fracture than trabecular factors.^41^^,^^42^ TBS provides an estimate of trabecular microarchitecture and therefore may not be expected to identify individuals with cortical bone deficits. Two pediatric studies conducted in cohorts of children with osteogenesis imperfecta, a genetic disorder negatively impacting bone strength, reported significant associations between TBS (determined from TBS Insight v3.3 and v2.1, respectively) and prevalent fracture.^43^^,^^44^ Notably, in the Liu et al., study, TBS was only significantly associated with vertebral fracture rate, not overall fracture rate.^44^ Vertebral fractures are common sequelae of skeletal fragility in children with osteogenesis imperfecta, but exceedingly rare in healthy children (*n* = 3 in our study sample). The low frequency of vertebral fracture in the BMDCS cohort may have contributed to the lack of an association between TBS and fracture incidence in our study. There are other cohorts of children with risk factors for impaired trabecular bone strength, including those exposed to chronic glucocorticoid treatment or those with chronic inflammatory disease. Further studies investigating the association between TBS and fracture incidence in these populations are needed define the utility of TBS in pediatric clinical practice. Additionally, a recent study in children with Duchenne muscular dystrophy reported that increases in TBS were greater in those treated with bisphosphonates compared to no treatment, suggesting the potential utility of TBS for monitoring response to pharmacologic intervention in the pediatric population.^17^

This study has limitations. The BMDCS dataset is multi-racial/multi-ethnic but does not completely align with the racial/ethnic distribution of US children. We used sample weights in the development of the reference ranges to more closely represent the racial/ethnic distribution in the US. Children with BMI >97th percentile at enrollment and those with diseases or exposures adversely affecting bone health were excluded, so we were unable to investigate the impact of obesity, chronic disease, non-ambulation, and other bone health risk factors on TBS. Additionally, the TBS reference data presented here were derived from LS images obtained on Hologic DXA machines, it is not known whether they are valid for TBS values calculated from LS images obtained on GE/Lunar or other DXA platforms. Reference data for TBS Insight v4.0 for children from Mexico City from GE/Lunar machines were recently published.^45^ Future studies investigating the equivalency of TBS across different DXA platforms and reference datasets should be considered. Finally, we evaluated the relationship between TBS and fractures in a sample of healthy children and the findings reported here may not be generalizable to children at-risk for bone fragility.

In conclusion, we used longitudinal DXA LS images obtained in healthy children from the BMDCS study to generate new pediatric reference data for TBS using TBS Insight software version 4.0. The reference data differed significantly from those provided in our prior publication and are appropriate for use by investigators and clinicians using the version 4.0 software installed on Hologic densitometers. We expect that these reference data will facilitate clinical research to define the utility of TBS to identify and manage children with and at risk of skeletal fragility.

## Supplementary Material

Revised_TBS_Ref_Data-Supplemental_Material_v_9-17-2027_ziag159

## Data Availability

Data from the original Bone Mineral Density in Childhood Study are available through the NICHD Data and Specimen Hub: https://dash.nichd.nih.gov/. TBS data available upon request.
