# Supplementary material for: New trabecular bone score reference values for children and adolescents according to age and sex and their relationship with body size and fracture incidence
Source: JBMR Plus. 2026 Sep 21;10(12):ziag159. doi: 10.1093/jbmrpl/ziag159 (PMC13768753; doi:10.1093/jbmrpl/ziag159)
Supplement: Supplementary file 1 [file revised_tbs_ref_data-supplemental_material_v_9-17-2027_ziag159.docx]

Supplemental tables and Figures

Contents

[**Supplemental Table 1:** LMS Values and Reference Ranges (Median, ±1 and ±2 Standard Deviations) for Trabecular Bone Score for Other Combinations of L1-L4 and Individual Vertebral Bodies for Children 5 to 20 Years	2](#_Toc238390635)

[**Supplemental Table 2:** LMS Values and Reference Ranges (Median, ±1 and ±2 Standard Deviations) for All Combinations of L1-L4 Trabecular Bone Score for Children 5 to 20 Years to the Tenth Decimal Place of Year	7](#_Toc238390636)

[**Supplemental Table 3:** Precision of other TBS Outcomes in Children 5-20 Years	82](#_Toc238390637)

[**Supplemental Table 4:** Fracture Details in Children 5 to 20 Years of Age	83](#_Toc238390638)

[**Supplemental Table 5:** Associations between trabecular bone score (TBS) and incident low and moderate energy fracture risk over 6 years in children 5-20 years of age	85](#_Toc238390639)

# **Supplemental Table 1:** LMS Values and Reference Ranges (Median, ±1 and ±2 Standard Deviations) for Trabecular Bone Score for Other Combinations of L1-L4 and Individual Vertebral Bodies for Children 5 to 20 Years

|  | Males | | | | | | | Females | | | | | | |
| --- | --- | --- | --- | --- | --- | --- | --- | --- | --- | --- | --- | --- | --- | --- |
| Age | L | S | -2 SD | -1 SD | 0 SD (M) | 1 SD | 2 SD | L | S | -2 SD | -1 SD | 0 SD (M) | 1 SD | 2 SD |
| L2 to L4 TBS | | | | | | | | | | | | | | |
| 5.0-5.9 | 4.661 | 0.054 | 1.117 | 1.220 | 1.298 | 1.362 | 1.417 | 1.846 | 0.055 | 1.120 | 1.194 | 1.265 | 1.333 | 1.398 |
| 6.0-6.9 | 3.874 | 0.054 | 1.131 | 1.225 | 1.302 | 1.368 | 1.425 | 1.855 | 0.056 | 1.114 | 1.191 | 1.264 | 1.334 | 1.400 |
| 7.0-7.9 | 3.155 | 0.054 | 1.142 | 1.229 | 1.305 | 1.372 | 1.433 | 1.860 | 0.057 | 1.111 | 1.189 | 1.264 | 1.334 | 1.402 |
| 8.0-8.9 | 2.535 | 0.054 | 1.150 | 1.232 | 1.306 | 1.374 | 1.437 | 1.861 | 0.057 | 1.112 | 1.191 | 1.265 | 1.336 | 1.404 |
| 9.0-9.9 | 2.020 | 0.054 | 1.155 | 1.233 | 1.305 | 1.374 | 1.439 | 1.862 | 0.057 | 1.118 | 1.197 | 1.271 | 1.341 | 1.409 |
| 10.0-10.9 | 1.586 | 0.054 | 1.158 | 1.232 | 1.304 | 1.374 | 1.441 | 1.874 | 0.056 | 1.133 | 1.210 | 1.284 | 1.353 | 1.420 |
| 11.0-11.9 | 1.204 | 0.054 | 1.163 | 1.235 | 1.306 | 1.377 | 1.447 | 1.906 | 0.054 | 1.162 | 1.238 | 1.311 | 1.380 | 1.446 |
| 12.0-12.9 | 0.871 | 0.054 | 1.177 | 1.247 | 1.317 | 1.388 | 1.460 | 1.960 | 0.051 | 1.204 | 1.279 | 1.350 | 1.418 | 1.483 |
| 13.0-13.9 | 0.593 | 0.052 | 1.207 | 1.274 | 1.343 | 1.413 | 1.485 | 2.036 | 0.049 | 1.247 | 1.320 | 1.389 | 1.455 | 1.518 |
| 14.0-14.9 | 0.416 | 0.048 | 1.250 | 1.314 | 1.379 | 1.447 | 1.517 | 2.133 | 0.046 | 1.280 | 1.351 | 1.418 | 1.481 | 1.542 |
| 15.0-15.9 | 0.395 | 0.045 | 1.292 | 1.354 | 1.417 | 1.481 | 1.548 | 2.247 | 0.044 | 1.303 | 1.372 | 1.437 | 1.499 | 1.557 |
| 16.0-16.9 | 0.572 | 0.042 | 1.325 | 1.385 | 1.446 | 1.508 | 1.571 | 2.373 | 0.042 | 1.318 | 1.385 | 1.449 | 1.509 | 1.565 |
| 17.0-17.9 | 0.953 | 0.041 | 1.345 | 1.405 | 1.465 | 1.525 | 1.586 | 2.504 | 0.041 | 1.329 | 1.396 | 1.458 | 1.516 | 1.571 |
| 18.0-18.9 | 1.494 | 0.041 | 1.355 | 1.417 | 1.478 | 1.537 | 1.596 | 2.634 | 0.040 | 1.340 | 1.405 | 1.465 | 1.522 | 1.575 |
| 19.0-19.9 | 2.115 | 0.041 | 1.357 | 1.423 | 1.486 | 1.546 | 1.603 | 2.761 | 0.038 | 1.347 | 1.410 | 1.468 | 1.522 | 1.573 |
| L1 to L3 TBS | | | | | | | | | | | | | | |
| 5.0-5.9 | 3.448 | 0.059 | 1.078 | 1.174 | 1.254 | 1.323 | 1.384 | 1.841 | 0.054 | 1.088 | 1.159 | 1.227 | 1.292 | 1.355 |
| 6.0-6.9 | 3.037 | 0.058 | 1.091 | 1.182 | 1.260 | 1.330 | 1.393 | 1.817 | 0.056 | 1.082 | 1.156 | 1.226 | 1.293 | 1.358 |
| 7.0-7.9 | 2.640 | 0.058 | 1.100 | 1.187 | 1.264 | 1.334 | 1.399 | 1.790 | 0.057 | 1.079 | 1.155 | 1.226 | 1.295 | 1.361 |
| 8.0-8.9 | 2.257 | 0.058 | 1.106 | 1.189 | 1.265 | 1.336 | 1.403 | 1.760 | 0.058 | 1.079 | 1.156 | 1.229 | 1.298 | 1.365 |
| 9.0-9.9 | 1.893 | 0.058 | 1.111 | 1.190 | 1.266 | 1.337 | 1.406 | 1.731 | 0.058 | 1.085 | 1.163 | 1.236 | 1.307 | 1.375 |
| 10.0-10.9 | 1.538 | 0.058 | 1.115 | 1.192 | 1.267 | 1.339 | 1.409 | 1.709 | 0.058 | 1.100 | 1.178 | 1.253 | 1.325 | 1.394 |
| 11.0-11.9 | 1.181 | 0.058 | 1.122 | 1.197 | 1.271 | 1.344 | 1.417 | 1.698 | 0.058 | 1.128 | 1.207 | 1.283 | 1.355 | 1.425 |
| 12.0-12.9 | 0.840 | 0.057 | 1.139 | 1.211 | 1.284 | 1.358 | 1.432 | 1.701 | 0.056 | 1.170 | 1.249 | 1.325 | 1.398 | 1.468 |
| 13.0-13.9 | 0.546 | 0.055 | 1.168 | 1.238 | 1.309 | 1.383 | 1.458 | 1.716 | 0.054 | 1.213 | 1.292 | 1.367 | 1.439 | 1.508 |
| 14.0-14.9 | 0.346 | 0.053 | 1.207 | 1.275 | 1.345 | 1.417 | 1.492 | 1.744 | 0.051 | 1.248 | 1.324 | 1.397 | 1.467 | 1.535 |
| 15.0-15.9 | 0.283 | 0.050 | 1.248 | 1.313 | 1.381 | 1.451 | 1.524 | 1.782 | 0.049 | 1.271 | 1.346 | 1.417 | 1.486 | 1.552 |
| 16.0-16.9 | 0.381 | 0.047 | 1.282 | 1.346 | 1.411 | 1.479 | 1.548 | 1.829 | 0.048 | 1.288 | 1.361 | 1.431 | 1.498 | 1.563 |
| 17.0-17.9 | 0.645 | 0.045 | 1.307 | 1.370 | 1.433 | 1.498 | 1.564 | 1.882 | 0.046 | 1.302 | 1.373 | 1.442 | 1.507 | 1.571 |
| 18.0-18.9 | 1.052 | 0.044 | 1.321 | 1.384 | 1.447 | 1.511 | 1.574 | 1.938 | 0.045 | 1.313 | 1.383 | 1.450 | 1.514 | 1.575 |
| 19.0-19.9 | 1.553 | 0.043 | 1.328 | 1.393 | 1.457 | 1.519 | 1.580 | 1.994 | 0.043 | 1.320 | 1.388 | 1.452 | 1.514 | 1.573 |
| L1 to L2 TBS | | | | | | | | | | | | | | |
| 5.0-5.9 | 2.425 | 0.068 | 1.042 | 1.141 | 1.229 | 1.309 | 1.383 | 2.281 | 0.061 | 1.043 | 1.127 | 1.203 | 1.274 | 1.340 |
| 6.0-6.9 | 2.408 | 0.068 | 1.048 | 1.147 | 1.235 | 1.315 | 1.389 | 2.196 | 0.063 | 1.040 | 1.126 | 1.204 | 1.277 | 1.346 |
| 7.0-7.9 | 2.338 | 0.068 | 1.053 | 1.151 | 1.239 | 1.319 | 1.394 | 2.111 | 0.064 | 1.036 | 1.124 | 1.204 | 1.279 | 1.350 |
| 8.0-8.9 | 2.200 | 0.067 | 1.058 | 1.154 | 1.241 | 1.321 | 1.396 | 2.025 | 0.065 | 1.036 | 1.124 | 1.205 | 1.282 | 1.354 |
| 9.0-9.9 | 1.996 | 0.067 | 1.062 | 1.155 | 1.241 | 1.321 | 1.397 | 1.940 | 0.066 | 1.043 | 1.131 | 1.213 | 1.291 | 1.364 |
| 10.0-10.9 | 1.728 | 0.066 | 1.068 | 1.157 | 1.242 | 1.323 | 1.400 | 1.857 | 0.065 | 1.059 | 1.148 | 1.231 | 1.309 | 1.384 |
| 11.0-11.9 | 1.410 | 0.066 | 1.079 | 1.165 | 1.248 | 1.329 | 1.408 | 1.779 | 0.065 | 1.089 | 1.178 | 1.261 | 1.341 | 1.417 |
| 12.0-12.9 | 1.091 | 0.065 | 1.097 | 1.179 | 1.261 | 1.342 | 1.423 | 1.707 | 0.063 | 1.132 | 1.220 | 1.304 | 1.385 | 1.462 |
| 13.0-13.9 | 0.801 | 0.062 | 1.127 | 1.206 | 1.285 | 1.366 | 1.448 | 1.642 | 0.061 | 1.176 | 1.263 | 1.347 | 1.427 | 1.505 |
| 14.0-14.9 | 0.559 | 0.059 | 1.166 | 1.241 | 1.319 | 1.398 | 1.479 | 1.582 | 0.059 | 1.210 | 1.295 | 1.377 | 1.457 | 1.534 |
| 15.0-15.9 | 0.397 | 0.056 | 1.207 | 1.279 | 1.354 | 1.431 | 1.510 | 1.528 | 0.057 | 1.233 | 1.316 | 1.396 | 1.474 | 1.550 |
| 16.0-16.9 | 0.343 | 0.053 | 1.244 | 1.313 | 1.384 | 1.459 | 1.535 | 1.478 | 0.055 | 1.250 | 1.331 | 1.410 | 1.486 | 1.561 |
| 17.0-17.9 | 0.422 | 0.050 | 1.270 | 1.337 | 1.407 | 1.478 | 1.552 | 1.432 | 0.054 | 1.264 | 1.343 | 1.420 | 1.495 | 1.569 |
| 18.0-18.9 | 0.634 | 0.049 | 1.285 | 1.352 | 1.421 | 1.490 | 1.561 | 1.388 | 0.052 | 1.275 | 1.352 | 1.427 | 1.501 | 1.573 |
| 19.0-19.9 | 0.940 | 0.048 | 1.293 | 1.361 | 1.429 | 1.498 | 1.567 | 1.344 | 0.051 | 1.281 | 1.356 | 1.429 | 1.501 | 1.572 |
| L3 to L4 TBS | | | | | | | | | | | | | | |
| 5.0-5.9 | 2.671 | 0.063 | 1.128 | 1.228 | 1.315 | 1.393 | 1.465 | 1.745 | 0.064 | 1.109 | 1.198 | 1.281 | 1.361 | 1.438 |
| 6.0-6.9 | 2.581 | 0.061 | 1.139 | 1.233 | 1.317 | 1.393 | 1.463 | 1.772 | 0.064 | 1.105 | 1.194 | 1.278 | 1.358 | 1.434 |
| 7.0-7.9 | 2.491 | 0.059 | 1.146 | 1.237 | 1.318 | 1.392 | 1.461 | 1.800 | 0.064 | 1.104 | 1.193 | 1.276 | 1.355 | 1.431 |
| 8.0-8.9 | 2.401 | 0.058 | 1.152 | 1.239 | 1.318 | 1.391 | 1.459 | 1.827 | 0.063 | 1.107 | 1.195 | 1.277 | 1.355 | 1.429 |
| 9.0-9.9 | 2.311 | 0.057 | 1.154 | 1.239 | 1.318 | 1.390 | 1.458 | 1.855 | 0.061 | 1.114 | 1.199 | 1.280 | 1.356 | 1.428 |
| 10.0-10.9 | 2.221 | 0.057 | 1.154 | 1.238 | 1.316 | 1.389 | 1.457 | 1.882 | 0.059 | 1.129 | 1.211 | 1.290 | 1.364 | 1.434 |
| 11.0-11.9 | 2.131 | 0.057 | 1.155 | 1.239 | 1.317 | 1.389 | 1.458 | 1.910 | 0.056 | 1.158 | 1.238 | 1.314 | 1.386 | 1.455 |
| 12.0-12.9 | 2.041 | 0.056 | 1.167 | 1.249 | 1.326 | 1.398 | 1.466 | 1.937 | 0.053 | 1.199 | 1.278 | 1.352 | 1.422 | 1.489 |
| 13.0-13.9 | 1.951 | 0.054 | 1.198 | 1.276 | 1.351 | 1.421 | 1.489 | 1.965 | 0.050 | 1.242 | 1.317 | 1.389 | 1.457 | 1.522 |
| 14.0-14.9 | 1.861 | 0.050 | 1.242 | 1.317 | 1.388 | 1.457 | 1.522 | 1.992 | 0.047 | 1.276 | 1.348 | 1.417 | 1.483 | 1.546 |
| 15.0-15.9 | 1.771 | 0.047 | 1.286 | 1.357 | 1.425 | 1.491 | 1.555 | 2.019 | 0.045 | 1.299 | 1.369 | 1.436 | 1.499 | 1.560 |
| 16.0-16.9 | 1.681 | 0.045 | 1.319 | 1.387 | 1.453 | 1.517 | 1.580 | 2.047 | 0.044 | 1.313 | 1.381 | 1.446 | 1.508 | 1.567 |
| 17.0-17.9 | 1.591 | 0.044 | 1.339 | 1.406 | 1.471 | 1.534 | 1.596 | 2.074 | 0.042 | 1.324 | 1.391 | 1.454 | 1.514 | 1.572 |
| 18.0-18.9 | 1.501 | 0.043 | 1.349 | 1.416 | 1.481 | 1.545 | 1.607 | 2.102 | 0.041 | 1.335 | 1.399 | 1.461 | 1.519 | 1.575 |
| 19.0-19.9 | 1.411 | 0.044 | 1.354 | 1.422 | 1.488 | 1.553 | 1.617 | 2.129 | 0.040 | 1.341 | 1.404 | 1.463 | 1.520 | 1.575 |
| L2 TO L3 TBS | | | | | | | | | | | | | | |
| 5.0-5.9 | 4.121 | 0.060 | 1.088 | 1.197 | 1.282 | 1.353 | 1.413 | 1.916 | 0.055 | 1.108 | 1.184 | 1.255 | 1.323 | 1.387 |
| 6.0-6.9 | 3.381 | 0.059 | 1.108 | 1.207 | 1.290 | 1.361 | 1.425 | 1.884 | 0.058 | 1.102 | 1.181 | 1.255 | 1.326 | 1.393 |
| 7.0-7.9 | 2.721 | 0.059 | 1.123 | 1.214 | 1.295 | 1.368 | 1.435 | 1.822 | 0.059 | 1.100 | 1.180 | 1.257 | 1.329 | 1.399 |
| 8.0-8.9 | 2.179 | 0.059 | 1.132 | 1.218 | 1.298 | 1.372 | 1.442 | 1.713 | 0.060 | 1.103 | 1.183 | 1.260 | 1.334 | 1.405 |
| 9.0-9.9 | 1.758 | 0.059 | 1.138 | 1.220 | 1.299 | 1.374 | 1.446 | 1.591 | 0.060 | 1.111 | 1.191 | 1.269 | 1.343 | 1.415 |
| 10.0-10.9 | 1.410 | 0.059 | 1.142 | 1.222 | 1.300 | 1.376 | 1.450 | 1.523 | 0.059 | 1.127 | 1.208 | 1.285 | 1.361 | 1.434 |
| 11.0-11.9 | 1.070 | 0.059 | 1.149 | 1.227 | 1.305 | 1.382 | 1.459 | 1.557 | 0.058 | 1.157 | 1.237 | 1.315 | 1.390 | 1.464 |
| 12.0-12.9 | 0.708 | 0.058 | 1.168 | 1.242 | 1.319 | 1.396 | 1.475 | 1.692 | 0.056 | 1.199 | 1.280 | 1.357 | 1.431 | 1.502 |
| 13.0-13.9 | 0.363 | 0.056 | 1.200 | 1.272 | 1.345 | 1.422 | 1.501 | 1.906 | 0.052 | 1.245 | 1.324 | 1.398 | 1.469 | 1.537 |
| 14.0-14.9 | 0.134 | 0.052 | 1.243 | 1.311 | 1.381 | 1.455 | 1.533 | 2.187 | 0.049 | 1.280 | 1.357 | 1.428 | 1.496 | 1.560 |
| 15.0-15.9 | 0.099 | 0.049 | 1.286 | 1.351 | 1.418 | 1.489 | 1.562 | 2.509 | 0.046 | 1.304 | 1.380 | 1.450 | 1.515 | 1.576 |
| 16.0-16.9 | 0.303 | 0.046 | 1.321 | 1.384 | 1.449 | 1.516 | 1.585 | 2.838 | 0.045 | 1.320 | 1.396 | 1.464 | 1.528 | 1.587 |
| 17.0-17.9 | 0.739 | 0.044 | 1.344 | 1.407 | 1.471 | 1.535 | 1.601 | 3.138 | 0.044 | 1.332 | 1.408 | 1.476 | 1.538 | 1.595 |
| 18.0-18.9 | 1.343 | 0.043 | 1.356 | 1.421 | 1.486 | 1.549 | 1.611 | 3.372 | 0.043 | 1.343 | 1.418 | 1.485 | 1.545 | 1.600 |
| 19.0-19.9 | 2.031 | 0.043 | 1.361 | 1.430 | 1.495 | 1.558 | 1.618 | 3.532 | 0.041 | 1.351 | 1.423 | 1.488 | 1.545 | 1.598 |
| L1 TBS | | | | | | | | | | | | | | |
| 5.0-5.9 | 2.025 | 0.085 | 0.976 | 1.094 | 1.201 | 1.299 | 1.390 | 1.835 | 0.088 | 0.949 | 1.066 | 1.173 | 1.272 | 1.366 |
| 6.0-6.9 | 1.921 | 0.084 | 0.982 | 1.097 | 1.203 | 1.301 | 1.392 | 1.748 | 0.086 | 0.954 | 1.066 | 1.170 | 1.268 | 1.360 |
| 7.0-7.9 | 1.818 | 0.083 | 0.986 | 1.099 | 1.202 | 1.300 | 1.391 | 1.660 | 0.084 | 0.959 | 1.067 | 1.168 | 1.264 | 1.355 |
| 8.0-8.9 | 1.714 | 0.082 | 0.989 | 1.099 | 1.201 | 1.297 | 1.388 | 1.573 | 0.082 | 0.964 | 1.069 | 1.167 | 1.261 | 1.351 |
| 9.0-9.9 | 1.610 | 0.082 | 0.993 | 1.099 | 1.199 | 1.295 | 1.386 | 1.486 | 0.081 | 0.975 | 1.076 | 1.172 | 1.265 | 1.355 |
| 10.0-10.9 | 1.507 | 0.081 | 0.997 | 1.101 | 1.200 | 1.295 | 1.387 | 1.398 | 0.079 | 0.994 | 1.093 | 1.188 | 1.280 | 1.370 |
| 11.0-11.9 | 1.403 | 0.080 | 1.005 | 1.107 | 1.205 | 1.300 | 1.392 | 1.311 | 0.077 | 1.025 | 1.123 | 1.218 | 1.311 | 1.402 |
| 12.0-12.9 | 1.300 | 0.079 | 1.020 | 1.120 | 1.216 | 1.311 | 1.403 | 1.223 | 0.076 | 1.066 | 1.164 | 1.260 | 1.355 | 1.448 |
| 13.0-13.9 | 1.196 | 0.076 | 1.047 | 1.144 | 1.239 | 1.333 | 1.426 | 1.136 | 0.074 | 1.108 | 1.206 | 1.303 | 1.399 | 1.494 |
| 14.0-14.9 | 1.093 | 0.073 | 1.084 | 1.178 | 1.272 | 1.365 | 1.457 | 1.048 | 0.072 | 1.140 | 1.237 | 1.334 | 1.431 | 1.527 |
| 15.0-15.9 | 0.989 | 0.069 | 1.125 | 1.216 | 1.306 | 1.397 | 1.488 | 0.961 | 0.071 | 1.162 | 1.257 | 1.353 | 1.449 | 1.546 |
| 16.0-16.9 | 0.886 | 0.066 | 1.163 | 1.249 | 1.337 | 1.425 | 1.514 | 0.873 | 0.069 | 1.177 | 1.271 | 1.365 | 1.460 | 1.556 |
| 17.0-17.9 | 0.782 | 0.063 | 1.191 | 1.274 | 1.359 | 1.444 | 1.531 | 0.786 | 0.068 | 1.190 | 1.281 | 1.374 | 1.468 | 1.564 |
| 18.0-18.9 | 0.679 | 0.060 | 1.210 | 1.290 | 1.372 | 1.455 | 1.540 | 0.698 | 0.067 | 1.200 | 1.289 | 1.380 | 1.473 | 1.567 |
| 19.0-19.9 | 0.575 | 0.059 | 1.222 | 1.300 | 1.380 | 1.462 | 1.547 | 0.611 | 0.065 | 1.207 | 1.293 | 1.382 | 1.473 | 1.566 |
| L2 TBS | | | | | | | | | | | | | | |
| 5.0-5.9 | 2.775 | 0.074 | 1.040 | 1.159 | 1.259 | 1.347 | 1.426 | 2.105 | 0.066 | 1.058 | 1.150 | 1.234 | 1.312 | 1.385 |
| 6.0-6.9 | 2.538 | 0.074 | 1.054 | 1.169 | 1.268 | 1.357 | 1.437 | 2.376 | 0.068 | 1.051 | 1.150 | 1.239 | 1.320 | 1.395 |
| 7.0-7.9 | 2.303 | 0.073 | 1.067 | 1.177 | 1.276 | 1.365 | 1.447 | 2.479 | 0.070 | 1.044 | 1.149 | 1.242 | 1.325 | 1.401 |
| 8.0-8.9 | 2.081 | 0.073 | 1.076 | 1.183 | 1.280 | 1.370 | 1.454 | 2.260 | 0.072 | 1.048 | 1.152 | 1.246 | 1.331 | 1.410 |
| 9.0-9.9 | 1.871 | 0.073 | 1.082 | 1.186 | 1.282 | 1.372 | 1.458 | 1.837 | 0.071 | 1.063 | 1.162 | 1.255 | 1.342 | 1.425 |
| 10.0-10.9 | 1.670 | 0.072 | 1.089 | 1.190 | 1.284 | 1.375 | 1.461 | 1.540 | 0.070 | 1.087 | 1.182 | 1.273 | 1.361 | 1.446 |
| 11.0-11.9 | 1.479 | 0.071 | 1.102 | 1.199 | 1.292 | 1.382 | 1.469 | 1.533 | 0.068 | 1.121 | 1.214 | 1.304 | 1.391 | 1.475 |
| 12.0-12.9 | 1.306 | 0.069 | 1.124 | 1.217 | 1.308 | 1.397 | 1.484 | 1.700 | 0.064 | 1.167 | 1.259 | 1.347 | 1.431 | 1.512 |
| 13.0-13.9 | 1.169 | 0.065 | 1.159 | 1.247 | 1.335 | 1.421 | 1.506 | 1.912 | 0.060 | 1.214 | 1.305 | 1.390 | 1.470 | 1.547 |
| 14.0-14.9 | 1.084 | 0.060 | 1.202 | 1.286 | 1.368 | 1.450 | 1.532 | 2.209 | 0.056 | 1.249 | 1.338 | 1.420 | 1.497 | 1.570 |
| 15.0-15.9 | 1.063 | 0.055 | 1.247 | 1.325 | 1.403 | 1.481 | 1.558 | 2.578 | 0.053 | 1.272 | 1.361 | 1.441 | 1.515 | 1.583 |
| 16.0-16.9 | 1.114 | 0.051 | 1.285 | 1.360 | 1.434 | 1.507 | 1.580 | 2.931 | 0.051 | 1.288 | 1.377 | 1.456 | 1.527 | 1.592 |
| 17.0-17.9 | 1.242 | 0.049 | 1.312 | 1.385 | 1.456 | 1.527 | 1.597 | 3.154 | 0.050 | 1.301 | 1.389 | 1.467 | 1.537 | 1.600 |
| 18.0-18.9 | 1.438 | 0.048 | 1.328 | 1.400 | 1.471 | 1.540 | 1.608 | 3.125 | 0.049 | 1.314 | 1.400 | 1.475 | 1.543 | 1.606 |
| 19.0-19.9 | 1.681 | 0.047 | 1.334 | 1.408 | 1.479 | 1.548 | 1.615 | 2.825 | 0.047 | 1.324 | 1.404 | 1.477 | 1.544 | 1.605 |
| L3 TBS | | | | | | | | | | | | | | |
| 5.0-5.9 | 3.252 | 0.072 | 1.075 | 1.203 | 1.305 | 1.392 | 1.468 | 1.181 | 0.067 | 1.102 | 1.189 | 1.276 | 1.361 | 1.446 |
| 6.0-6.9 | 2.763 | 0.069 | 1.103 | 1.215 | 1.311 | 1.396 | 1.473 | 1.278 | 0.069 | 1.093 | 1.183 | 1.272 | 1.360 | 1.445 |
| 7.0-7.9 | 2.351 | 0.066 | 1.121 | 1.223 | 1.314 | 1.398 | 1.475 | 1.372 | 0.069 | 1.091 | 1.184 | 1.273 | 1.361 | 1.446 |
| 8.0-8.9 | 1.998 | 0.065 | 1.132 | 1.227 | 1.315 | 1.398 | 1.476 | 1.462 | 0.068 | 1.097 | 1.189 | 1.277 | 1.363 | 1.446 |
| 9.0-9.9 | 1.671 | 0.064 | 1.140 | 1.230 | 1.317 | 1.400 | 1.479 | 1.555 | 0.067 | 1.106 | 1.197 | 1.284 | 1.368 | 1.449 |
| 10.0-10.9 | 1.354 | 0.064 | 1.144 | 1.231 | 1.317 | 1.400 | 1.482 | 1.659 | 0.065 | 1.122 | 1.213 | 1.299 | 1.382 | 1.462 |
| 11.0-11.9 | 1.060 | 0.064 | 1.149 | 1.234 | 1.319 | 1.404 | 1.488 | 1.777 | 0.063 | 1.151 | 1.242 | 1.328 | 1.409 | 1.488 |
| 12.0-12.9 | 0.801 | 0.063 | 1.166 | 1.248 | 1.332 | 1.416 | 1.501 | 1.913 | 0.059 | 1.195 | 1.284 | 1.367 | 1.446 | 1.522 |
| 13.0-13.9 | 0.594 | 0.060 | 1.199 | 1.278 | 1.359 | 1.442 | 1.526 | 2.069 | 0.055 | 1.243 | 1.328 | 1.407 | 1.482 | 1.553 |
| 14.0-14.9 | 0.489 | 0.056 | 1.245 | 1.320 | 1.397 | 1.477 | 1.558 | 2.247 | 0.051 | 1.282 | 1.362 | 1.438 | 1.508 | 1.575 |
| 15.0-15.9 | 0.540 | 0.052 | 1.289 | 1.361 | 1.435 | 1.511 | 1.589 | 2.446 | 0.048 | 1.308 | 1.386 | 1.459 | 1.527 | 1.591 |
| 16.0-16.9 | 0.778 | 0.049 | 1.322 | 1.394 | 1.465 | 1.538 | 1.611 | 2.663 | 0.047 | 1.323 | 1.402 | 1.474 | 1.540 | 1.603 |
| 17.0-17.9 | 1.184 | 0.047 | 1.344 | 1.416 | 1.487 | 1.557 | 1.626 | 2.893 | 0.047 | 1.333 | 1.413 | 1.486 | 1.552 | 1.613 |
| 18.0-18.9 | 1.722 | 0.047 | 1.356 | 1.430 | 1.501 | 1.570 | 1.637 | 3.130 | 0.046 | 1.342 | 1.423 | 1.495 | 1.560 | 1.620 |
| 19.0-19.9 | 2.349 | 0.046 | 1.361 | 1.439 | 1.511 | 1.579 | 1.644 | 3.369 | 0.044 | 1.348 | 1.428 | 1.498 | 1.561 | 1.619 |
| L4 TBS | | | | | | | | | | | | | | |
| 5.0-5.9 | 2.347 | 0.077 | 1.098 | 1.221 | 1.329 | 1.426 | 1.515 | 1.901 | 0.085 | 1.049 | 1.174 | 1.288 | 1.393 | 1.492 |
| 6.0-6.9 | 2.194 | 0.077 | 1.099 | 1.219 | 1.326 | 1.423 | 1.513 | 1.890 | 0.083 | 1.051 | 1.172 | 1.284 | 1.387 | 1.484 |
| 7.0-7.9 | 2.055 | 0.076 | 1.102 | 1.218 | 1.323 | 1.421 | 1.511 | 1.880 | 0.081 | 1.054 | 1.171 | 1.279 | 1.379 | 1.474 |
| 8.0-8.9 | 1.938 | 0.075 | 1.106 | 1.218 | 1.321 | 1.417 | 1.507 | 1.869 | 0.078 | 1.060 | 1.173 | 1.276 | 1.373 | 1.464 |
| 9.0-9.9 | 1.844 | 0.074 | 1.109 | 1.217 | 1.317 | 1.411 | 1.501 | 1.859 | 0.075 | 1.070 | 1.177 | 1.276 | 1.368 | 1.456 |
| 10.0-10.9 | 1.774 | 0.072 | 1.111 | 1.215 | 1.313 | 1.406 | 1.494 | 1.848 | 0.071 | 1.087 | 1.188 | 1.281 | 1.370 | 1.453 |
| 11.0-11.9 | 1.728 | 0.071 | 1.115 | 1.216 | 1.312 | 1.402 | 1.489 | 1.838 | 0.066 | 1.119 | 1.214 | 1.302 | 1.386 | 1.466 |
| 12.0-12.9 | 1.705 | 0.068 | 1.130 | 1.227 | 1.318 | 1.405 | 1.488 | 1.827 | 0.062 | 1.162 | 1.252 | 1.336 | 1.417 | 1.493 |
| 13.0-13.9 | 1.701 | 0.063 | 1.163 | 1.254 | 1.341 | 1.423 | 1.503 | 1.817 | 0.058 | 1.205 | 1.290 | 1.371 | 1.448 | 1.522 |
| 14.0-14.9 | 1.715 | 0.059 | 1.208 | 1.294 | 1.376 | 1.455 | 1.531 | 1.807 | 0.055 | 1.237 | 1.319 | 1.398 | 1.472 | 1.544 |
| 15.0-15.9 | 1.744 | 0.054 | 1.252 | 1.334 | 1.413 | 1.488 | 1.561 | 1.796 | 0.052 | 1.259 | 1.338 | 1.414 | 1.486 | 1.556 |
| 16.0-16.9 | 1.791 | 0.052 | 1.285 | 1.364 | 1.440 | 1.512 | 1.583 | 1.786 | 0.051 | 1.269 | 1.346 | 1.420 | 1.491 | 1.559 |
| 17.0-17.9 | 1.856 | 0.050 | 1.302 | 1.380 | 1.455 | 1.526 | 1.595 | 1.775 | 0.049 | 1.277 | 1.352 | 1.423 | 1.492 | 1.559 |
| 18.0-18.9 | 1.938 | 0.050 | 1.309 | 1.388 | 1.463 | 1.534 | 1.603 | 1.765 | 0.048 | 1.286 | 1.359 | 1.428 | 1.496 | 1.560 |
| 19.0-19.9 | 2.028 | 0.051 | 1.310 | 1.392 | 1.468 | 1.541 | 1.610 | 1.754 | 0.047 | 1.293 | 1.364 | 1.431 | 1.497 | 1.560 |

# **Supplemental Table 2:** LMS Values and Reference Ranges (Median, ±1 and ±2 Standard Deviations) for All Combinations of L1-L4 Trabecular Bone Score for Children 5 to 20 Years to the Tenth Decimal Place of Year

| L1 to L4 TBS | | | | | | | | | | | | | | |
| --- | --- | --- | --- | --- | --- | --- | --- | --- | --- | --- | --- | --- | --- | --- |
|  | Males | | | | | | | Females | | | | | | |
| Age | L | S | -2 SD | -1 SD | 0 SD (M) | 1 SD | 2 SD | L | S | -2 SD | -1 SD | 0 SD (M) | 1 SD | 2 SD |
| 5.0 | 4.491 | 0.054 | 1.096 | 1.195 | 1.271 | 1.334 | 1.388 | 2.299 | 0.052 | 1.103 | 1.176 | 1.243 | 1.306 | 1.365 |
| 5.1 | 4.432 | 0.054 | 1.097 | 1.195 | 1.272 | 1.335 | 1.389 | 2.290 | 0.052 | 1.103 | 1.176 | 1.243 | 1.306 | 1.366 |
| 5.2 | 4.372 | 0.054 | 1.099 | 1.196 | 1.272 | 1.335 | 1.390 | 2.281 | 0.053 | 1.102 | 1.175 | 1.243 | 1.306 | 1.366 |
| 5.3 | 4.313 | 0.054 | 1.100 | 1.196 | 1.273 | 1.336 | 1.391 | 2.272 | 0.053 | 1.102 | 1.175 | 1.243 | 1.306 | 1.366 |
| 5.4 | 4.254 | 0.054 | 1.101 | 1.197 | 1.273 | 1.336 | 1.391 | 2.263 | 0.053 | 1.101 | 1.175 | 1.243 | 1.306 | 1.366 |
| 5.5 | 4.196 | 0.054 | 1.102 | 1.198 | 1.273 | 1.337 | 1.392 | 2.254 | 0.053 | 1.101 | 1.174 | 1.242 | 1.306 | 1.366 |
| 5.6 | 4.137 | 0.054 | 1.104 | 1.198 | 1.274 | 1.338 | 1.393 | 2.246 | 0.053 | 1.100 | 1.174 | 1.242 | 1.306 | 1.367 |
| 5.7 | 4.079 | 0.054 | 1.105 | 1.199 | 1.274 | 1.338 | 1.394 | 2.237 | 0.053 | 1.100 | 1.173 | 1.242 | 1.306 | 1.367 |
| 5.8 | 4.020 | 0.054 | 1.106 | 1.199 | 1.275 | 1.339 | 1.395 | 2.228 | 0.053 | 1.099 | 1.173 | 1.242 | 1.306 | 1.367 |
| 5.9 | 3.963 | 0.054 | 1.107 | 1.200 | 1.275 | 1.339 | 1.395 | 2.219 | 0.054 | 1.099 | 1.173 | 1.242 | 1.306 | 1.367 |
| 6.0 | 3.905 | 0.054 | 1.108 | 1.200 | 1.275 | 1.340 | 1.396 | 2.210 | 0.054 | 1.098 | 1.172 | 1.242 | 1.306 | 1.367 |
| 6.1 | 3.848 | 0.054 | 1.109 | 1.201 | 1.276 | 1.340 | 1.397 | 2.201 | 0.054 | 1.098 | 1.172 | 1.241 | 1.306 | 1.368 |
| 6.2 | 3.791 | 0.054 | 1.110 | 1.201 | 1.276 | 1.341 | 1.397 | 2.192 | 0.054 | 1.097 | 1.172 | 1.241 | 1.306 | 1.368 |
| 6.3 | 3.734 | 0.054 | 1.111 | 1.202 | 1.277 | 1.341 | 1.398 | 2.183 | 0.054 | 1.097 | 1.171 | 1.241 | 1.306 | 1.368 |
| 6.4 | 3.677 | 0.054 | 1.112 | 1.202 | 1.277 | 1.342 | 1.399 | 2.174 | 0.054 | 1.096 | 1.171 | 1.241 | 1.306 | 1.368 |
| 6.5 | 3.621 | 0.054 | 1.113 | 1.202 | 1.277 | 1.342 | 1.399 | 2.165 | 0.054 | 1.096 | 1.171 | 1.241 | 1.306 | 1.368 |
| 6.6 | 3.565 | 0.054 | 1.114 | 1.203 | 1.277 | 1.342 | 1.400 | 2.156 | 0.055 | 1.095 | 1.171 | 1.241 | 1.306 | 1.368 |
| 6.7 | 3.510 | 0.054 | 1.115 | 1.203 | 1.278 | 1.343 | 1.401 | 2.146 | 0.055 | 1.095 | 1.170 | 1.240 | 1.306 | 1.369 |
| 6.8 | 3.454 | 0.054 | 1.116 | 1.204 | 1.278 | 1.343 | 1.401 | 2.137 | 0.055 | 1.095 | 1.170 | 1.240 | 1.306 | 1.369 |
| 6.9 | 3.400 | 0.054 | 1.117 | 1.204 | 1.278 | 1.343 | 1.402 | 2.128 | 0.055 | 1.094 | 1.170 | 1.240 | 1.306 | 1.369 |
| 7.0 | 3.345 | 0.054 | 1.118 | 1.204 | 1.279 | 1.344 | 1.402 | 2.118 | 0.055 | 1.094 | 1.170 | 1.240 | 1.306 | 1.369 |
| 7.1 | 3.291 | 0.054 | 1.118 | 1.205 | 1.279 | 1.344 | 1.403 | 2.109 | 0.055 | 1.094 | 1.169 | 1.240 | 1.306 | 1.369 |
| 7.2 | 3.237 | 0.054 | 1.119 | 1.205 | 1.279 | 1.344 | 1.403 | 2.099 | 0.055 | 1.093 | 1.169 | 1.240 | 1.306 | 1.369 |
| 7.3 | 3.184 | 0.054 | 1.120 | 1.205 | 1.279 | 1.345 | 1.404 | 2.089 | 0.055 | 1.093 | 1.169 | 1.240 | 1.306 | 1.370 |
| 7.4 | 3.130 | 0.054 | 1.121 | 1.206 | 1.279 | 1.345 | 1.404 | 2.079 | 0.055 | 1.093 | 1.169 | 1.240 | 1.306 | 1.370 |
| 7.5 | 3.078 | 0.054 | 1.121 | 1.206 | 1.279 | 1.345 | 1.405 | 2.070 | 0.055 | 1.093 | 1.169 | 1.240 | 1.307 | 1.370 |
| 7.6 | 3.025 | 0.054 | 1.122 | 1.206 | 1.279 | 1.345 | 1.405 | 2.060 | 0.056 | 1.093 | 1.169 | 1.240 | 1.307 | 1.370 |
| 7.7 | 2.973 | 0.054 | 1.123 | 1.206 | 1.280 | 1.345 | 1.406 | 2.050 | 0.056 | 1.093 | 1.169 | 1.240 | 1.307 | 1.370 |
| 7.8 | 2.922 | 0.054 | 1.123 | 1.206 | 1.280 | 1.346 | 1.406 | 2.040 | 0.056 | 1.093 | 1.169 | 1.240 | 1.307 | 1.371 |
| 7.9 | 2.871 | 0.054 | 1.124 | 1.207 | 1.280 | 1.346 | 1.406 | 2.030 | 0.056 | 1.093 | 1.169 | 1.240 | 1.307 | 1.371 |
| 8.0 | 2.820 | 0.054 | 1.125 | 1.207 | 1.280 | 1.346 | 1.406 | 2.019 | 0.056 | 1.093 | 1.169 | 1.240 | 1.307 | 1.371 |
| 8.1 | 2.770 | 0.054 | 1.125 | 1.207 | 1.280 | 1.346 | 1.407 | 2.009 | 0.056 | 1.093 | 1.169 | 1.240 | 1.307 | 1.371 |
| 8.2 | 2.720 | 0.054 | 1.126 | 1.207 | 1.280 | 1.346 | 1.407 | 1.999 | 0.056 | 1.093 | 1.169 | 1.240 | 1.307 | 1.372 |
| 8.3 | 2.670 | 0.054 | 1.126 | 1.207 | 1.280 | 1.346 | 1.407 | 1.989 | 0.056 | 1.093 | 1.169 | 1.240 | 1.308 | 1.372 |
| 8.4 | 2.621 | 0.054 | 1.127 | 1.207 | 1.280 | 1.346 | 1.407 | 1.978 | 0.056 | 1.093 | 1.169 | 1.240 | 1.308 | 1.372 |
| 8.5 | 2.572 | 0.054 | 1.128 | 1.207 | 1.280 | 1.346 | 1.408 | 1.968 | 0.056 | 1.093 | 1.169 | 1.241 | 1.308 | 1.373 |
| 8.6 | 2.524 | 0.054 | 1.128 | 1.207 | 1.280 | 1.346 | 1.408 | 1.958 | 0.056 | 1.094 | 1.170 | 1.241 | 1.309 | 1.373 |
| 8.7 | 2.476 | 0.054 | 1.129 | 1.207 | 1.279 | 1.346 | 1.408 | 1.948 | 0.056 | 1.094 | 1.170 | 1.241 | 1.309 | 1.373 |
| 8.8 | 2.429 | 0.054 | 1.129 | 1.208 | 1.279 | 1.346 | 1.408 | 1.938 | 0.056 | 1.095 | 1.170 | 1.242 | 1.309 | 1.374 |
| 8.9 | 2.382 | 0.054 | 1.129 | 1.208 | 1.279 | 1.346 | 1.408 | 1.927 | 0.056 | 1.095 | 1.171 | 1.242 | 1.310 | 1.375 |
| 9.0 | 2.335 | 0.054 | 1.130 | 1.208 | 1.279 | 1.346 | 1.408 | 1.917 | 0.056 | 1.096 | 1.171 | 1.243 | 1.310 | 1.375 |
| 9.1 | 2.289 | 0.054 | 1.130 | 1.208 | 1.279 | 1.346 | 1.408 | 1.907 | 0.056 | 1.096 | 1.172 | 1.243 | 1.311 | 1.376 |
| 9.2 | 2.243 | 0.054 | 1.131 | 1.208 | 1.279 | 1.346 | 1.409 | 1.897 | 0.056 | 1.097 | 1.172 | 1.244 | 1.312 | 1.376 |
| 9.3 | 2.197 | 0.054 | 1.131 | 1.208 | 1.279 | 1.346 | 1.409 | 1.887 | 0.056 | 1.098 | 1.173 | 1.244 | 1.312 | 1.377 |
| 9.4 | 2.152 | 0.054 | 1.131 | 1.208 | 1.279 | 1.346 | 1.409 | 1.878 | 0.056 | 1.098 | 1.174 | 1.245 | 1.313 | 1.378 |
| 9.5 | 2.107 | 0.054 | 1.132 | 1.208 | 1.279 | 1.346 | 1.409 | 1.868 | 0.056 | 1.099 | 1.175 | 1.246 | 1.314 | 1.379 |
| 9.6 | 2.063 | 0.054 | 1.132 | 1.208 | 1.279 | 1.346 | 1.409 | 1.859 | 0.056 | 1.100 | 1.176 | 1.247 | 1.315 | 1.380 |
| 9.7 | 2.018 | 0.054 | 1.132 | 1.208 | 1.278 | 1.346 | 1.409 | 1.849 | 0.056 | 1.101 | 1.177 | 1.248 | 1.316 | 1.381 |
| 9.8 | 1.974 | 0.054 | 1.133 | 1.208 | 1.278 | 1.345 | 1.409 | 1.840 | 0.056 | 1.102 | 1.178 | 1.249 | 1.317 | 1.382 |
| 9.9 | 1.931 | 0.054 | 1.133 | 1.208 | 1.278 | 1.345 | 1.410 | 1.831 | 0.056 | 1.104 | 1.179 | 1.250 | 1.318 | 1.383 |
| 10.0 | 1.887 | 0.054 | 1.133 | 1.208 | 1.278 | 1.345 | 1.410 | 1.822 | 0.056 | 1.105 | 1.180 | 1.251 | 1.319 | 1.385 |
| 10.1 | 1.844 | 0.054 | 1.134 | 1.208 | 1.278 | 1.345 | 1.410 | 1.814 | 0.056 | 1.107 | 1.181 | 1.253 | 1.321 | 1.386 |
| 10.2 | 1.801 | 0.054 | 1.134 | 1.208 | 1.278 | 1.345 | 1.410 | 1.805 | 0.055 | 1.108 | 1.183 | 1.254 | 1.322 | 1.388 |
| 10.3 | 1.759 | 0.054 | 1.134 | 1.208 | 1.278 | 1.345 | 1.410 | 1.797 | 0.055 | 1.110 | 1.185 | 1.256 | 1.324 | 1.389 |
| 10.4 | 1.716 | 0.054 | 1.135 | 1.208 | 1.278 | 1.346 | 1.411 | 1.789 | 0.055 | 1.112 | 1.186 | 1.257 | 1.326 | 1.391 |
| 10.5 | 1.674 | 0.054 | 1.135 | 1.208 | 1.278 | 1.346 | 1.411 | 1.781 | 0.055 | 1.114 | 1.188 | 1.259 | 1.328 | 1.393 |
| 10.6 | 1.633 | 0.054 | 1.135 | 1.208 | 1.278 | 1.346 | 1.411 | 1.774 | 0.055 | 1.116 | 1.190 | 1.261 | 1.330 | 1.395 |
| 10.7 | 1.591 | 0.054 | 1.136 | 1.208 | 1.278 | 1.346 | 1.412 | 1.766 | 0.055 | 1.118 | 1.192 | 1.264 | 1.332 | 1.397 |
| 10.8 | 1.550 | 0.054 | 1.136 | 1.208 | 1.278 | 1.346 | 1.412 | 1.759 | 0.055 | 1.120 | 1.195 | 1.266 | 1.334 | 1.400 |
| 10.9 | 1.509 | 0.054 | 1.137 | 1.209 | 1.278 | 1.346 | 1.413 | 1.752 | 0.055 | 1.123 | 1.197 | 1.268 | 1.337 | 1.402 |
| 11.0 | 1.468 | 0.054 | 1.137 | 1.209 | 1.279 | 1.347 | 1.413 | 1.746 | 0.055 | 1.125 | 1.200 | 1.271 | 1.339 | 1.405 |
| 11.1 | 1.428 | 0.054 | 1.138 | 1.209 | 1.279 | 1.347 | 1.414 | 1.739 | 0.055 | 1.128 | 1.203 | 1.274 | 1.342 | 1.408 |
| 11.2 | 1.389 | 0.054 | 1.138 | 1.210 | 1.279 | 1.348 | 1.414 | 1.733 | 0.055 | 1.131 | 1.206 | 1.277 | 1.345 | 1.411 |
| 11.3 | 1.350 | 0.054 | 1.139 | 1.210 | 1.280 | 1.348 | 1.415 | 1.727 | 0.054 | 1.135 | 1.209 | 1.280 | 1.348 | 1.414 |
| 11.4 | 1.311 | 0.054 | 1.140 | 1.211 | 1.280 | 1.349 | 1.416 | 1.722 | 0.054 | 1.138 | 1.212 | 1.283 | 1.352 | 1.418 |
| 11.5 | 1.273 | 0.054 | 1.141 | 1.211 | 1.281 | 1.349 | 1.417 | 1.716 | 0.054 | 1.142 | 1.216 | 1.287 | 1.355 | 1.421 |
| 11.6 | 1.235 | 0.054 | 1.142 | 1.212 | 1.281 | 1.350 | 1.418 | 1.711 | 0.054 | 1.145 | 1.219 | 1.290 | 1.359 | 1.425 |
| 11.7 | 1.198 | 0.054 | 1.143 | 1.213 | 1.282 | 1.351 | 1.418 | 1.707 | 0.054 | 1.149 | 1.223 | 1.294 | 1.363 | 1.429 |
| 11.8 | 1.161 | 0.054 | 1.144 | 1.214 | 1.283 | 1.351 | 1.419 | 1.702 | 0.054 | 1.153 | 1.227 | 1.298 | 1.366 | 1.433 |
| 11.9 | 1.125 | 0.054 | 1.145 | 1.215 | 1.284 | 1.352 | 1.421 | 1.698 | 0.053 | 1.157 | 1.231 | 1.302 | 1.370 | 1.437 |
| 12.0 | 1.090 | 0.054 | 1.146 | 1.216 | 1.285 | 1.353 | 1.422 | 1.694 | 0.053 | 1.161 | 1.235 | 1.306 | 1.375 | 1.441 |
| 12.1 | 1.055 | 0.054 | 1.148 | 1.217 | 1.286 | 1.355 | 1.423 | 1.690 | 0.053 | 1.165 | 1.239 | 1.310 | 1.379 | 1.445 |
| 12.2 | 1.020 | 0.053 | 1.149 | 1.218 | 1.287 | 1.356 | 1.425 | 1.686 | 0.053 | 1.170 | 1.244 | 1.314 | 1.383 | 1.449 |
| 12.3 | 0.986 | 0.053 | 1.151 | 1.220 | 1.288 | 1.357 | 1.426 | 1.683 | 0.053 | 1.174 | 1.248 | 1.319 | 1.387 | 1.453 |
| 12.4 | 0.953 | 0.053 | 1.153 | 1.221 | 1.290 | 1.359 | 1.428 | 1.680 | 0.053 | 1.179 | 1.252 | 1.323 | 1.391 | 1.457 |
| 12.5 | 0.921 | 0.053 | 1.155 | 1.223 | 1.292 | 1.360 | 1.430 | 1.677 | 0.052 | 1.183 | 1.257 | 1.327 | 1.396 | 1.462 |
| 12.6 | 0.889 | 0.053 | 1.157 | 1.225 | 1.293 | 1.362 | 1.432 | 1.675 | 0.052 | 1.188 | 1.261 | 1.332 | 1.400 | 1.466 |
| 12.7 | 0.858 | 0.053 | 1.159 | 1.227 | 1.295 | 1.364 | 1.434 | 1.673 | 0.052 | 1.192 | 1.265 | 1.336 | 1.404 | 1.470 |
| 12.8 | 0.827 | 0.053 | 1.162 | 1.229 | 1.298 | 1.366 | 1.436 | 1.671 | 0.052 | 1.196 | 1.270 | 1.340 | 1.408 | 1.474 |
| 12.9 | 0.798 | 0.053 | 1.164 | 1.232 | 1.300 | 1.369 | 1.438 | 1.669 | 0.051 | 1.201 | 1.274 | 1.344 | 1.412 | 1.478 |
| 13.0 | 0.769 | 0.053 | 1.167 | 1.234 | 1.302 | 1.371 | 1.441 | 1.668 | 0.051 | 1.205 | 1.278 | 1.348 | 1.416 | 1.482 |
| 13.1 | 0.740 | 0.052 | 1.170 | 1.237 | 1.305 | 1.374 | 1.443 | 1.667 | 0.051 | 1.209 | 1.282 | 1.352 | 1.420 | 1.486 |
| 13.2 | 0.713 | 0.052 | 1.173 | 1.240 | 1.308 | 1.376 | 1.446 | 1.666 | 0.051 | 1.214 | 1.286 | 1.356 | 1.424 | 1.490 |
| 13.3 | 0.687 | 0.052 | 1.176 | 1.243 | 1.310 | 1.379 | 1.449 | 1.666 | 0.050 | 1.218 | 1.290 | 1.360 | 1.428 | 1.493 |
| 13.4 | 0.661 | 0.052 | 1.180 | 1.246 | 1.313 | 1.382 | 1.452 | 1.665 | 0.050 | 1.222 | 1.294 | 1.364 | 1.431 | 1.497 |
| 13.5 | 0.636 | 0.052 | 1.183 | 1.249 | 1.317 | 1.385 | 1.455 | 1.665 | 0.050 | 1.226 | 1.298 | 1.368 | 1.435 | 1.500 |
| 13.6 | 0.613 | 0.051 | 1.187 | 1.253 | 1.320 | 1.388 | 1.458 | 1.666 | 0.050 | 1.230 | 1.302 | 1.371 | 1.438 | 1.503 |
| 13.7 | 0.590 | 0.051 | 1.190 | 1.256 | 1.323 | 1.392 | 1.462 | 1.666 | 0.050 | 1.233 | 1.305 | 1.375 | 1.442 | 1.507 |
| 13.8 | 0.569 | 0.051 | 1.194 | 1.260 | 1.327 | 1.395 | 1.465 | 1.667 | 0.049 | 1.237 | 1.309 | 1.378 | 1.445 | 1.509 |
| 13.9 | 0.548 | 0.051 | 1.198 | 1.263 | 1.330 | 1.398 | 1.468 | 1.668 | 0.049 | 1.240 | 1.312 | 1.381 | 1.448 | 1.512 |
| 14.0 | 0.529 | 0.051 | 1.202 | 1.267 | 1.334 | 1.402 | 1.472 | 1.669 | 0.049 | 1.244 | 1.315 | 1.384 | 1.450 | 1.515 |
| 14.1 | 0.511 | 0.050 | 1.206 | 1.271 | 1.337 | 1.405 | 1.475 | 1.671 | 0.049 | 1.247 | 1.318 | 1.387 | 1.453 | 1.517 |
| 14.2 | 0.494 | 0.050 | 1.210 | 1.275 | 1.341 | 1.409 | 1.479 | 1.673 | 0.048 | 1.250 | 1.321 | 1.389 | 1.456 | 1.520 |
| 14.3 | 0.478 | 0.050 | 1.214 | 1.279 | 1.345 | 1.413 | 1.482 | 1.675 | 0.048 | 1.253 | 1.324 | 1.392 | 1.458 | 1.522 |
| 14.4 | 0.464 | 0.049 | 1.219 | 1.283 | 1.349 | 1.416 | 1.486 | 1.677 | 0.048 | 1.256 | 1.326 | 1.395 | 1.460 | 1.524 |
| 14.5 | 0.451 | 0.049 | 1.223 | 1.287 | 1.352 | 1.420 | 1.489 | 1.680 | 0.048 | 1.259 | 1.329 | 1.397 | 1.463 | 1.526 |
| 14.6 | 0.439 | 0.049 | 1.227 | 1.291 | 1.356 | 1.423 | 1.492 | 1.683 | 0.048 | 1.261 | 1.332 | 1.399 | 1.465 | 1.528 |
| 14.7 | 0.428 | 0.049 | 1.231 | 1.295 | 1.360 | 1.427 | 1.496 | 1.686 | 0.047 | 1.264 | 1.334 | 1.401 | 1.467 | 1.530 |
| 14.8 | 0.420 | 0.048 | 1.235 | 1.299 | 1.364 | 1.431 | 1.499 | 1.689 | 0.047 | 1.266 | 1.336 | 1.404 | 1.469 | 1.532 |
| 14.9 | 0.412 | 0.048 | 1.240 | 1.303 | 1.367 | 1.434 | 1.503 | 1.693 | 0.047 | 1.269 | 1.338 | 1.406 | 1.471 | 1.534 |
| 15.0 | 0.406 | 0.048 | 1.244 | 1.307 | 1.371 | 1.438 | 1.506 | 1.696 | 0.047 | 1.271 | 1.340 | 1.408 | 1.472 | 1.535 |
| 15.1 | 0.401 | 0.047 | 1.248 | 1.310 | 1.375 | 1.441 | 1.509 | 1.700 | 0.047 | 1.273 | 1.342 | 1.409 | 1.474 | 1.537 |
| 15.2 | 0.398 | 0.047 | 1.252 | 1.314 | 1.378 | 1.444 | 1.512 | 1.704 | 0.046 | 1.275 | 1.344 | 1.411 | 1.476 | 1.538 |
| 15.3 | 0.397 | 0.047 | 1.256 | 1.318 | 1.382 | 1.448 | 1.515 | 1.709 | 0.046 | 1.277 | 1.346 | 1.413 | 1.477 | 1.540 |
| 15.4 | 0.397 | 0.047 | 1.260 | 1.322 | 1.385 | 1.451 | 1.518 | 1.713 | 0.046 | 1.279 | 1.348 | 1.414 | 1.479 | 1.541 |
| 15.5 | 0.398 | 0.046 | 1.264 | 1.326 | 1.389 | 1.454 | 1.521 | 1.718 | 0.046 | 1.281 | 1.350 | 1.416 | 1.480 | 1.542 |
| 15.6 | 0.401 | 0.046 | 1.268 | 1.329 | 1.392 | 1.457 | 1.524 | 1.723 | 0.046 | 1.283 | 1.351 | 1.417 | 1.481 | 1.543 |
| 15.7 | 0.406 | 0.046 | 1.271 | 1.333 | 1.396 | 1.460 | 1.527 | 1.728 | 0.046 | 1.284 | 1.353 | 1.419 | 1.482 | 1.544 |
| 15.8 | 0.412 | 0.045 | 1.275 | 1.336 | 1.399 | 1.463 | 1.529 | 1.733 | 0.046 | 1.286 | 1.354 | 1.420 | 1.484 | 1.545 |
| 15.9 | 0.420 | 0.045 | 1.279 | 1.339 | 1.402 | 1.466 | 1.532 | 1.739 | 0.045 | 1.288 | 1.356 | 1.421 | 1.485 | 1.546 |
| 16.0 | 0.429 | 0.045 | 1.282 | 1.343 | 1.405 | 1.469 | 1.534 | 1.745 | 0.045 | 1.289 | 1.357 | 1.422 | 1.486 | 1.547 |
| 16.1 | 0.440 | 0.045 | 1.285 | 1.346 | 1.408 | 1.472 | 1.537 | 1.751 | 0.045 | 1.291 | 1.358 | 1.424 | 1.487 | 1.548 |
| 16.2 | 0.453 | 0.044 | 1.288 | 1.349 | 1.411 | 1.474 | 1.539 | 1.757 | 0.045 | 1.292 | 1.360 | 1.425 | 1.488 | 1.549 |
| 16.3 | 0.467 | 0.044 | 1.292 | 1.352 | 1.413 | 1.477 | 1.541 | 1.763 | 0.045 | 1.293 | 1.361 | 1.426 | 1.489 | 1.549 |
| 16.4 | 0.483 | 0.044 | 1.295 | 1.355 | 1.416 | 1.479 | 1.543 | 1.769 | 0.045 | 1.295 | 1.362 | 1.427 | 1.489 | 1.550 |
| 16.5 | 0.500 | 0.044 | 1.297 | 1.357 | 1.419 | 1.481 | 1.545 | 1.776 | 0.045 | 1.296 | 1.363 | 1.428 | 1.490 | 1.551 |
| 16.6 | 0.520 | 0.043 | 1.300 | 1.360 | 1.421 | 1.484 | 1.547 | 1.782 | 0.044 | 1.297 | 1.364 | 1.429 | 1.491 | 1.551 |
| 16.7 | 0.540 | 0.043 | 1.303 | 1.362 | 1.423 | 1.486 | 1.549 | 1.789 | 0.044 | 1.298 | 1.365 | 1.430 | 1.492 | 1.552 |
| 16.8 | 0.563 | 0.043 | 1.305 | 1.365 | 1.426 | 1.488 | 1.551 | 1.796 | 0.044 | 1.300 | 1.366 | 1.430 | 1.492 | 1.553 |
| 16.9 | 0.587 | 0.043 | 1.308 | 1.367 | 1.428 | 1.490 | 1.552 | 1.803 | 0.044 | 1.301 | 1.367 | 1.431 | 1.493 | 1.553 |
| 17.0 | 0.612 | 0.043 | 1.310 | 1.369 | 1.430 | 1.491 | 1.554 | 1.810 | 0.044 | 1.302 | 1.368 | 1.432 | 1.494 | 1.554 |
| 17.1 | 0.639 | 0.043 | 1.312 | 1.371 | 1.432 | 1.493 | 1.555 | 1.817 | 0.044 | 1.303 | 1.369 | 1.433 | 1.495 | 1.554 |
| 17.2 | 0.668 | 0.042 | 1.314 | 1.373 | 1.434 | 1.495 | 1.557 | 1.824 | 0.044 | 1.304 | 1.370 | 1.434 | 1.495 | 1.555 |
| 17.3 | 0.698 | 0.042 | 1.316 | 1.375 | 1.435 | 1.496 | 1.558 | 1.831 | 0.043 | 1.305 | 1.371 | 1.435 | 1.496 | 1.555 |
| 17.4 | 0.730 | 0.042 | 1.318 | 1.377 | 1.437 | 1.498 | 1.559 | 1.839 | 0.043 | 1.307 | 1.372 | 1.436 | 1.497 | 1.556 |
| 17.5 | 0.762 | 0.042 | 1.319 | 1.379 | 1.439 | 1.499 | 1.561 | 1.846 | 0.043 | 1.308 | 1.373 | 1.437 | 1.497 | 1.556 |
| 17.6 | 0.797 | 0.042 | 1.321 | 1.380 | 1.440 | 1.501 | 1.562 | 1.854 | 0.043 | 1.309 | 1.374 | 1.437 | 1.498 | 1.557 |
| 17.7 | 0.832 | 0.042 | 1.322 | 1.382 | 1.442 | 1.502 | 1.563 | 1.861 | 0.043 | 1.310 | 1.375 | 1.438 | 1.499 | 1.557 |
| 17.8 | 0.869 | 0.042 | 1.324 | 1.383 | 1.443 | 1.503 | 1.564 | 1.869 | 0.043 | 1.311 | 1.376 | 1.439 | 1.499 | 1.558 |
| 17.9 | 0.907 | 0.042 | 1.325 | 1.384 | 1.444 | 1.505 | 1.565 | 1.877 | 0.043 | 1.312 | 1.377 | 1.440 | 1.500 | 1.558 |
| 18.0 | 0.946 | 0.041 | 1.326 | 1.386 | 1.446 | 1.506 | 1.566 | 1.884 | 0.042 | 1.313 | 1.378 | 1.441 | 1.501 | 1.559 |
| 18.1 | 0.987 | 0.041 | 1.327 | 1.387 | 1.447 | 1.507 | 1.567 | 1.892 | 0.042 | 1.315 | 1.379 | 1.441 | 1.501 | 1.559 |
| 18.2 | 1.028 | 0.041 | 1.328 | 1.388 | 1.448 | 1.508 | 1.568 | 1.900 | 0.042 | 1.316 | 1.380 | 1.442 | 1.502 | 1.559 |
| 18.3 | 1.070 | 0.041 | 1.329 | 1.389 | 1.449 | 1.509 | 1.568 | 1.907 | 0.042 | 1.317 | 1.381 | 1.443 | 1.502 | 1.560 |
| 18.4 | 1.113 | 0.041 | 1.330 | 1.390 | 1.450 | 1.510 | 1.569 | 1.915 | 0.042 | 1.318 | 1.382 | 1.443 | 1.503 | 1.560 |
| 18.5 | 1.157 | 0.041 | 1.330 | 1.391 | 1.451 | 1.511 | 1.570 | 1.923 | 0.042 | 1.319 | 1.383 | 1.444 | 1.503 | 1.560 |
| 18.6 | 1.202 | 0.041 | 1.331 | 1.392 | 1.452 | 1.512 | 1.571 | 1.930 | 0.042 | 1.319 | 1.383 | 1.444 | 1.503 | 1.560 |
| 18.7 | 1.247 | 0.041 | 1.332 | 1.393 | 1.453 | 1.512 | 1.571 | 1.938 | 0.041 | 1.320 | 1.384 | 1.445 | 1.504 | 1.560 |
| 18.8 | 1.293 | 0.041 | 1.332 | 1.393 | 1.454 | 1.513 | 1.572 | 1.946 | 0.041 | 1.321 | 1.385 | 1.445 | 1.504 | 1.560 |
| 18.9 | 1.340 | 0.041 | 1.333 | 1.394 | 1.455 | 1.514 | 1.573 | 1.953 | 0.041 | 1.322 | 1.385 | 1.446 | 1.504 | 1.560 |
| 19.0 | 1.387 | 0.041 | 1.333 | 1.395 | 1.455 | 1.515 | 1.574 | 1.961 | 0.041 | 1.323 | 1.386 | 1.446 | 1.504 | 1.560 |
| 19.1 | 1.435 | 0.041 | 1.334 | 1.396 | 1.456 | 1.516 | 1.574 | 1.968 | 0.041 | 1.323 | 1.386 | 1.446 | 1.504 | 1.560 |
| 19.2 | 1.483 | 0.041 | 1.334 | 1.396 | 1.457 | 1.517 | 1.575 | 1.976 | 0.041 | 1.324 | 1.386 | 1.446 | 1.504 | 1.559 |
| 19.3 | 1.531 | 0.041 | 1.335 | 1.397 | 1.458 | 1.517 | 1.576 | 1.984 | 0.040 | 1.324 | 1.387 | 1.446 | 1.504 | 1.559 |
| 19.4 | 1.580 | 0.041 | 1.335 | 1.398 | 1.459 | 1.518 | 1.576 | 1.991 | 0.040 | 1.325 | 1.387 | 1.446 | 1.504 | 1.559 |
| 19.5 | 1.630 | 0.041 | 1.336 | 1.398 | 1.459 | 1.519 | 1.577 | 1.998 | 0.040 | 1.325 | 1.387 | 1.447 | 1.503 | 1.558 |
| 19.6 | 1.679 | 0.041 | 1.336 | 1.399 | 1.460 | 1.520 | 1.578 | 2.006 | 0.040 | 1.326 | 1.388 | 1.447 | 1.503 | 1.558 |
| 19.7 | 1.729 | 0.041 | 1.336 | 1.399 | 1.461 | 1.520 | 1.578 | 2.013 | 0.040 | 1.326 | 1.388 | 1.446 | 1.503 | 1.557 |
| 19.8 | 1.779 | 0.041 | 1.336 | 1.400 | 1.461 | 1.521 | 1.579 | 2.020 | 0.040 | 1.327 | 1.388 | 1.446 | 1.503 | 1.557 |
| 19.9 | 1.830 | 0.041 | 1.336 | 1.400 | 1.462 | 1.522 | 1.579 | 2.028 | 0.039 | 1.327 | 1.388 | 1.446 | 1.502 | 1.556 |
| 20.0 | 1.880 | 0.041 | 1.336 | 1.401 | 1.463 | 1.522 | 1.580 | 2.035 | 0.039 | 1.328 | 1.388 | 1.446 | 1.502 | 1.556 |

| L2 to L4 TBS | | | | | | | | | | | | | | |
| --- | --- | --- | --- | --- | --- | --- | --- | --- | --- | --- | --- | --- | --- | --- |
|  | Males | | | | | | | Females | | | | | | |
| Age | L | S | -2 SD | -1 SD | 0 SD (M) | 1 SD | 2 SD | L | S | -2 SD | -1 SD | 0 SD (M) | 1 SD | 2 SD |
| 5.0 | 5.068 | 0.054 | 1.109 | 1.217 | 1.296 | 1.360 | 1.413 | 1.841 | 0.054 | 1.123 | 1.196 | 1.266 | 1.332 | 1.396 |
| 5.1 | 4.986 | 0.054 | 1.111 | 1.218 | 1.297 | 1.360 | 1.414 | 1.842 | 0.054 | 1.122 | 1.196 | 1.266 | 1.332 | 1.396 |
| 5.2 | 4.904 | 0.054 | 1.112 | 1.218 | 1.297 | 1.361 | 1.414 | 1.843 | 0.054 | 1.121 | 1.195 | 1.265 | 1.332 | 1.397 |
| 5.3 | 4.823 | 0.054 | 1.114 | 1.219 | 1.297 | 1.361 | 1.415 | 1.844 | 0.054 | 1.121 | 1.195 | 1.265 | 1.333 | 1.397 |
| 5.4 | 4.742 | 0.054 | 1.116 | 1.219 | 1.298 | 1.362 | 1.416 | 1.845 | 0.055 | 1.120 | 1.195 | 1.265 | 1.333 | 1.397 |
| 5.5 | 4.661 | 0.054 | 1.117 | 1.220 | 1.298 | 1.362 | 1.417 | 1.846 | 0.055 | 1.120 | 1.194 | 1.265 | 1.333 | 1.398 |
| 5.6 | 4.580 | 0.054 | 1.119 | 1.221 | 1.299 | 1.363 | 1.418 | 1.847 | 0.055 | 1.119 | 1.194 | 1.265 | 1.333 | 1.398 |
| 5.7 | 4.500 | 0.054 | 1.120 | 1.221 | 1.299 | 1.363 | 1.418 | 1.848 | 0.055 | 1.118 | 1.193 | 1.265 | 1.333 | 1.398 |
| 5.8 | 4.420 | 0.054 | 1.122 | 1.222 | 1.299 | 1.364 | 1.419 | 1.849 | 0.055 | 1.118 | 1.193 | 1.265 | 1.333 | 1.399 |
| 5.9 | 4.340 | 0.054 | 1.123 | 1.222 | 1.300 | 1.364 | 1.420 | 1.850 | 0.055 | 1.117 | 1.193 | 1.265 | 1.333 | 1.399 |
| 6.0 | 4.261 | 0.054 | 1.125 | 1.223 | 1.300 | 1.365 | 1.421 | 1.851 | 0.056 | 1.117 | 1.192 | 1.265 | 1.333 | 1.399 |
| 6.1 | 4.182 | 0.054 | 1.126 | 1.223 | 1.301 | 1.365 | 1.422 | 1.852 | 0.056 | 1.116 | 1.192 | 1.264 | 1.333 | 1.399 |
| 6.2 | 4.104 | 0.054 | 1.128 | 1.224 | 1.301 | 1.366 | 1.423 | 1.853 | 0.056 | 1.115 | 1.192 | 1.264 | 1.333 | 1.400 |
| 6.3 | 4.027 | 0.054 | 1.129 | 1.224 | 1.301 | 1.366 | 1.423 | 1.853 | 0.056 | 1.115 | 1.192 | 1.264 | 1.333 | 1.400 |
| 6.4 | 3.950 | 0.054 | 1.130 | 1.225 | 1.302 | 1.367 | 1.424 | 1.854 | 0.056 | 1.114 | 1.191 | 1.264 | 1.334 | 1.400 |
| 6.5 | 3.874 | 0.054 | 1.131 | 1.225 | 1.302 | 1.368 | 1.425 | 1.855 | 0.056 | 1.114 | 1.191 | 1.264 | 1.334 | 1.400 |
| 6.6 | 3.798 | 0.054 | 1.133 | 1.226 | 1.302 | 1.368 | 1.426 | 1.856 | 0.056 | 1.113 | 1.191 | 1.264 | 1.334 | 1.400 |
| 6.7 | 3.724 | 0.054 | 1.134 | 1.226 | 1.303 | 1.369 | 1.427 | 1.857 | 0.057 | 1.113 | 1.190 | 1.264 | 1.334 | 1.401 |
| 6.8 | 3.649 | 0.054 | 1.135 | 1.227 | 1.303 | 1.369 | 1.428 | 1.857 | 0.057 | 1.113 | 1.190 | 1.264 | 1.334 | 1.401 |
| 6.9 | 3.576 | 0.054 | 1.136 | 1.227 | 1.303 | 1.369 | 1.428 | 1.858 | 0.057 | 1.112 | 1.190 | 1.264 | 1.334 | 1.401 |
| 7.0 | 3.504 | 0.054 | 1.137 | 1.227 | 1.304 | 1.370 | 1.429 | 1.858 | 0.057 | 1.112 | 1.190 | 1.264 | 1.334 | 1.401 |
| 7.1 | 3.432 | 0.054 | 1.138 | 1.228 | 1.304 | 1.370 | 1.430 | 1.859 | 0.057 | 1.112 | 1.190 | 1.264 | 1.334 | 1.401 |
| 7.2 | 3.361 | 0.054 | 1.139 | 1.228 | 1.304 | 1.371 | 1.431 | 1.859 | 0.057 | 1.111 | 1.190 | 1.264 | 1.334 | 1.401 |
| 7.3 | 3.292 | 0.054 | 1.140 | 1.229 | 1.304 | 1.371 | 1.431 | 1.860 | 0.057 | 1.111 | 1.189 | 1.264 | 1.334 | 1.402 |
| 7.4 | 3.223 | 0.054 | 1.141 | 1.229 | 1.305 | 1.372 | 1.432 | 1.860 | 0.057 | 1.111 | 1.189 | 1.264 | 1.334 | 1.402 |
| 7.5 | 3.155 | 0.054 | 1.142 | 1.229 | 1.305 | 1.372 | 1.433 | 1.860 | 0.057 | 1.111 | 1.189 | 1.264 | 1.334 | 1.402 |
| 7.6 | 3.088 | 0.054 | 1.143 | 1.230 | 1.305 | 1.372 | 1.433 | 1.861 | 0.057 | 1.111 | 1.189 | 1.264 | 1.334 | 1.402 |
| 7.7 | 3.022 | 0.054 | 1.144 | 1.230 | 1.305 | 1.373 | 1.434 | 1.861 | 0.057 | 1.111 | 1.189 | 1.264 | 1.335 | 1.402 |
| 7.8 | 2.958 | 0.054 | 1.145 | 1.230 | 1.305 | 1.373 | 1.434 | 1.861 | 0.057 | 1.111 | 1.190 | 1.264 | 1.335 | 1.403 |
| 7.9 | 2.894 | 0.054 | 1.146 | 1.230 | 1.305 | 1.373 | 1.435 | 1.861 | 0.057 | 1.111 | 1.190 | 1.264 | 1.335 | 1.403 |
| 8.0 | 2.831 | 0.054 | 1.146 | 1.231 | 1.306 | 1.373 | 1.435 | 1.861 | 0.057 | 1.111 | 1.190 | 1.264 | 1.335 | 1.403 |
| 8.1 | 2.770 | 0.054 | 1.147 | 1.231 | 1.306 | 1.373 | 1.436 | 1.861 | 0.057 | 1.111 | 1.190 | 1.264 | 1.335 | 1.403 |
| 8.2 | 2.709 | 0.054 | 1.148 | 1.231 | 1.306 | 1.374 | 1.436 | 1.861 | 0.057 | 1.111 | 1.190 | 1.265 | 1.336 | 1.403 |
| 8.3 | 2.650 | 0.054 | 1.149 | 1.231 | 1.306 | 1.374 | 1.436 | 1.861 | 0.057 | 1.112 | 1.190 | 1.265 | 1.336 | 1.404 |
| 8.4 | 2.592 | 0.054 | 1.149 | 1.232 | 1.306 | 1.374 | 1.437 | 1.861 | 0.057 | 1.112 | 1.191 | 1.265 | 1.336 | 1.404 |
| 8.5 | 2.535 | 0.054 | 1.150 | 1.232 | 1.306 | 1.374 | 1.437 | 1.861 | 0.057 | 1.112 | 1.191 | 1.265 | 1.336 | 1.404 |
| 8.6 | 2.479 | 0.054 | 1.151 | 1.232 | 1.306 | 1.374 | 1.437 | 1.861 | 0.057 | 1.113 | 1.191 | 1.266 | 1.337 | 1.404 |
| 8.7 | 2.424 | 0.054 | 1.151 | 1.232 | 1.306 | 1.374 | 1.438 | 1.861 | 0.057 | 1.113 | 1.192 | 1.266 | 1.337 | 1.405 |
| 8.8 | 2.370 | 0.054 | 1.152 | 1.232 | 1.306 | 1.374 | 1.438 | 1.861 | 0.057 | 1.114 | 1.192 | 1.267 | 1.337 | 1.405 |
| 8.9 | 2.317 | 0.054 | 1.153 | 1.232 | 1.306 | 1.374 | 1.438 | 1.861 | 0.057 | 1.114 | 1.193 | 1.267 | 1.338 | 1.405 |
| 9.0 | 2.265 | 0.054 | 1.153 | 1.232 | 1.306 | 1.374 | 1.438 | 1.861 | 0.057 | 1.115 | 1.193 | 1.267 | 1.338 | 1.406 |
| 9.1 | 2.214 | 0.054 | 1.154 | 1.232 | 1.306 | 1.374 | 1.439 | 1.861 | 0.057 | 1.115 | 1.194 | 1.268 | 1.339 | 1.406 |
| 9.2 | 2.164 | 0.054 | 1.154 | 1.232 | 1.305 | 1.374 | 1.439 | 1.861 | 0.057 | 1.116 | 1.194 | 1.269 | 1.339 | 1.407 |
| 9.3 | 2.115 | 0.054 | 1.154 | 1.232 | 1.305 | 1.374 | 1.439 | 1.861 | 0.057 | 1.117 | 1.195 | 1.269 | 1.340 | 1.407 |
| 9.4 | 2.067 | 0.054 | 1.155 | 1.233 | 1.305 | 1.374 | 1.439 | 1.861 | 0.057 | 1.118 | 1.196 | 1.270 | 1.340 | 1.408 |
| 9.5 | 2.020 | 0.054 | 1.155 | 1.233 | 1.305 | 1.374 | 1.439 | 1.862 | 0.057 | 1.118 | 1.197 | 1.271 | 1.341 | 1.409 |
| 9.6 | 1.973 | 0.054 | 1.156 | 1.232 | 1.305 | 1.374 | 1.439 | 1.862 | 0.057 | 1.119 | 1.198 | 1.272 | 1.342 | 1.409 |
| 9.7 | 1.928 | 0.054 | 1.156 | 1.232 | 1.305 | 1.374 | 1.440 | 1.863 | 0.057 | 1.121 | 1.199 | 1.272 | 1.343 | 1.410 |
| 9.8 | 1.883 | 0.054 | 1.156 | 1.232 | 1.305 | 1.374 | 1.440 | 1.864 | 0.056 | 1.122 | 1.200 | 1.273 | 1.344 | 1.411 |
| 9.9 | 1.838 | 0.054 | 1.156 | 1.232 | 1.305 | 1.374 | 1.440 | 1.865 | 0.056 | 1.123 | 1.201 | 1.275 | 1.345 | 1.412 |
| 10.0 | 1.795 | 0.054 | 1.157 | 1.232 | 1.304 | 1.374 | 1.440 | 1.866 | 0.056 | 1.124 | 1.202 | 1.276 | 1.346 | 1.413 |
| 10.1 | 1.752 | 0.054 | 1.157 | 1.232 | 1.304 | 1.374 | 1.440 | 1.867 | 0.056 | 1.126 | 1.204 | 1.277 | 1.347 | 1.414 |
| 10.2 | 1.709 | 0.054 | 1.157 | 1.232 | 1.304 | 1.373 | 1.440 | 1.869 | 0.056 | 1.127 | 1.205 | 1.279 | 1.349 | 1.416 |
| 10.3 | 1.668 | 0.054 | 1.157 | 1.232 | 1.304 | 1.373 | 1.441 | 1.870 | 0.056 | 1.129 | 1.207 | 1.280 | 1.350 | 1.417 |
| 10.4 | 1.626 | 0.054 | 1.158 | 1.232 | 1.304 | 1.374 | 1.441 | 1.872 | 0.056 | 1.131 | 1.208 | 1.282 | 1.352 | 1.418 |
| 10.5 | 1.586 | 0.054 | 1.158 | 1.232 | 1.304 | 1.374 | 1.441 | 1.874 | 0.056 | 1.133 | 1.210 | 1.284 | 1.353 | 1.420 |
| 10.6 | 1.545 | 0.054 | 1.158 | 1.232 | 1.304 | 1.374 | 1.441 | 1.876 | 0.055 | 1.135 | 1.212 | 1.286 | 1.355 | 1.422 |
| 10.7 | 1.505 | 0.054 | 1.158 | 1.232 | 1.304 | 1.374 | 1.442 | 1.879 | 0.055 | 1.137 | 1.215 | 1.288 | 1.357 | 1.424 |
| 10.8 | 1.466 | 0.054 | 1.159 | 1.232 | 1.304 | 1.374 | 1.442 | 1.881 | 0.055 | 1.140 | 1.217 | 1.290 | 1.359 | 1.426 |
| 10.9 | 1.427 | 0.054 | 1.159 | 1.233 | 1.304 | 1.374 | 1.443 | 1.884 | 0.055 | 1.142 | 1.219 | 1.292 | 1.362 | 1.428 |
| 11.0 | 1.389 | 0.054 | 1.160 | 1.233 | 1.304 | 1.374 | 1.443 | 1.887 | 0.055 | 1.145 | 1.222 | 1.295 | 1.364 | 1.431 |
| 11.1 | 1.351 | 0.054 | 1.160 | 1.233 | 1.305 | 1.375 | 1.444 | 1.890 | 0.055 | 1.148 | 1.225 | 1.298 | 1.367 | 1.433 |
| 11.2 | 1.313 | 0.054 | 1.161 | 1.233 | 1.305 | 1.375 | 1.444 | 1.894 | 0.054 | 1.151 | 1.228 | 1.301 | 1.370 | 1.436 |
| 11.3 | 1.276 | 0.054 | 1.161 | 1.234 | 1.305 | 1.376 | 1.445 | 1.898 | 0.054 | 1.155 | 1.231 | 1.304 | 1.373 | 1.439 |
| 11.4 | 1.240 | 0.054 | 1.162 | 1.234 | 1.306 | 1.376 | 1.446 | 1.902 | 0.054 | 1.158 | 1.235 | 1.307 | 1.376 | 1.442 |
| 11.5 | 1.204 | 0.054 | 1.163 | 1.235 | 1.306 | 1.377 | 1.447 | 1.906 | 0.054 | 1.162 | 1.238 | 1.311 | 1.380 | 1.446 |
| 11.6 | 1.169 | 0.054 | 1.164 | 1.236 | 1.307 | 1.377 | 1.448 | 1.910 | 0.054 | 1.165 | 1.242 | 1.314 | 1.383 | 1.449 |
| 11.7 | 1.134 | 0.054 | 1.164 | 1.236 | 1.308 | 1.378 | 1.448 | 1.915 | 0.053 | 1.169 | 1.246 | 1.318 | 1.387 | 1.452 |
| 11.8 | 1.099 | 0.054 | 1.166 | 1.237 | 1.308 | 1.379 | 1.450 | 1.920 | 0.053 | 1.173 | 1.249 | 1.322 | 1.390 | 1.456 |
| 11.9 | 1.065 | 0.054 | 1.167 | 1.238 | 1.309 | 1.380 | 1.451 | 1.925 | 0.053 | 1.177 | 1.253 | 1.325 | 1.394 | 1.460 |
| 12.0 | 1.032 | 0.054 | 1.168 | 1.239 | 1.310 | 1.381 | 1.452 | 1.930 | 0.053 | 1.181 | 1.257 | 1.329 | 1.398 | 1.463 |
| 12.1 | 0.998 | 0.054 | 1.170 | 1.240 | 1.311 | 1.382 | 1.453 | 1.936 | 0.053 | 1.186 | 1.262 | 1.333 | 1.402 | 1.467 |
| 12.2 | 0.966 | 0.054 | 1.171 | 1.242 | 1.313 | 1.384 | 1.455 | 1.941 | 0.052 | 1.190 | 1.266 | 1.338 | 1.406 | 1.471 |
| 12.3 | 0.934 | 0.054 | 1.173 | 1.243 | 1.314 | 1.385 | 1.456 | 1.947 | 0.052 | 1.195 | 1.270 | 1.342 | 1.410 | 1.475 |
| 12.4 | 0.902 | 0.054 | 1.175 | 1.245 | 1.316 | 1.387 | 1.458 | 1.953 | 0.052 | 1.199 | 1.274 | 1.346 | 1.414 | 1.479 |
| 12.5 | 0.871 | 0.054 | 1.177 | 1.247 | 1.317 | 1.388 | 1.460 | 1.960 | 0.051 | 1.204 | 1.279 | 1.350 | 1.418 | 1.483 |
| 12.6 | 0.840 | 0.054 | 1.179 | 1.249 | 1.319 | 1.390 | 1.462 | 1.967 | 0.051 | 1.208 | 1.283 | 1.354 | 1.422 | 1.487 |
| 12.7 | 0.809 | 0.053 | 1.182 | 1.251 | 1.321 | 1.392 | 1.464 | 1.973 | 0.051 | 1.213 | 1.287 | 1.358 | 1.426 | 1.490 |
| 12.8 | 0.780 | 0.053 | 1.184 | 1.254 | 1.324 | 1.394 | 1.466 | 1.980 | 0.051 | 1.217 | 1.292 | 1.363 | 1.430 | 1.494 |
| 12.9 | 0.751 | 0.053 | 1.187 | 1.256 | 1.326 | 1.397 | 1.468 | 1.988 | 0.050 | 1.221 | 1.296 | 1.367 | 1.434 | 1.498 |
| 13.0 | 0.723 | 0.053 | 1.190 | 1.259 | 1.328 | 1.399 | 1.471 | 1.995 | 0.050 | 1.226 | 1.300 | 1.371 | 1.437 | 1.501 |
| 13.1 | 0.695 | 0.053 | 1.193 | 1.262 | 1.331 | 1.402 | 1.473 | 2.003 | 0.050 | 1.230 | 1.304 | 1.374 | 1.441 | 1.505 |
| 13.2 | 0.668 | 0.052 | 1.197 | 1.265 | 1.334 | 1.404 | 1.476 | 2.011 | 0.049 | 1.234 | 1.308 | 1.378 | 1.445 | 1.508 |
| 13.3 | 0.642 | 0.052 | 1.200 | 1.268 | 1.337 | 1.407 | 1.479 | 2.019 | 0.049 | 1.239 | 1.312 | 1.382 | 1.448 | 1.512 |
| 13.4 | 0.617 | 0.052 | 1.204 | 1.271 | 1.340 | 1.410 | 1.482 | 2.028 | 0.049 | 1.243 | 1.316 | 1.386 | 1.452 | 1.515 |
| 13.5 | 0.593 | 0.052 | 1.207 | 1.274 | 1.343 | 1.413 | 1.485 | 2.036 | 0.049 | 1.247 | 1.320 | 1.389 | 1.455 | 1.518 |
| 13.6 | 0.570 | 0.051 | 1.211 | 1.278 | 1.346 | 1.416 | 1.488 | 2.045 | 0.048 | 1.251 | 1.324 | 1.393 | 1.458 | 1.521 |
| 13.7 | 0.548 | 0.051 | 1.215 | 1.282 | 1.350 | 1.419 | 1.491 | 2.054 | 0.048 | 1.254 | 1.327 | 1.396 | 1.461 | 1.524 |
| 13.8 | 0.527 | 0.051 | 1.219 | 1.285 | 1.353 | 1.423 | 1.494 | 2.063 | 0.048 | 1.258 | 1.331 | 1.399 | 1.464 | 1.526 |
| 13.9 | 0.507 | 0.050 | 1.223 | 1.289 | 1.357 | 1.426 | 1.497 | 2.073 | 0.047 | 1.262 | 1.334 | 1.402 | 1.467 | 1.529 |
| 14.0 | 0.488 | 0.050 | 1.228 | 1.293 | 1.360 | 1.430 | 1.500 | 2.082 | 0.047 | 1.265 | 1.337 | 1.405 | 1.470 | 1.531 |
| 14.1 | 0.471 | 0.050 | 1.232 | 1.297 | 1.364 | 1.433 | 1.504 | 2.092 | 0.047 | 1.268 | 1.340 | 1.408 | 1.472 | 1.534 |
| 14.2 | 0.455 | 0.049 | 1.236 | 1.301 | 1.368 | 1.437 | 1.507 | 2.102 | 0.047 | 1.271 | 1.343 | 1.411 | 1.475 | 1.536 |
| 14.3 | 0.440 | 0.049 | 1.241 | 1.305 | 1.372 | 1.440 | 1.510 | 2.112 | 0.046 | 1.274 | 1.346 | 1.413 | 1.477 | 1.538 |
| 14.4 | 0.427 | 0.049 | 1.245 | 1.309 | 1.376 | 1.444 | 1.514 | 2.123 | 0.046 | 1.277 | 1.348 | 1.416 | 1.479 | 1.540 |
| 14.5 | 0.416 | 0.048 | 1.250 | 1.314 | 1.379 | 1.447 | 1.517 | 2.133 | 0.046 | 1.280 | 1.351 | 1.418 | 1.481 | 1.542 |
| 14.6 | 0.406 | 0.048 | 1.254 | 1.318 | 1.383 | 1.451 | 1.520 | 2.144 | 0.046 | 1.283 | 1.354 | 1.420 | 1.484 | 1.544 |
| 14.7 | 0.397 | 0.048 | 1.258 | 1.322 | 1.387 | 1.454 | 1.524 | 2.155 | 0.045 | 1.285 | 1.356 | 1.422 | 1.486 | 1.546 |
| 14.8 | 0.391 | 0.047 | 1.263 | 1.326 | 1.391 | 1.458 | 1.527 | 2.166 | 0.045 | 1.288 | 1.358 | 1.425 | 1.487 | 1.547 |
| 14.9 | 0.386 | 0.047 | 1.267 | 1.330 | 1.395 | 1.461 | 1.530 | 2.177 | 0.045 | 1.290 | 1.361 | 1.427 | 1.489 | 1.549 |
| 15.0 | 0.382 | 0.047 | 1.272 | 1.334 | 1.399 | 1.465 | 1.533 | 2.188 | 0.045 | 1.293 | 1.363 | 1.429 | 1.491 | 1.551 |
| 15.1 | 0.381 | 0.046 | 1.276 | 1.338 | 1.402 | 1.468 | 1.536 | 2.200 | 0.045 | 1.295 | 1.365 | 1.430 | 1.493 | 1.552 |
| 15.2 | 0.381 | 0.046 | 1.280 | 1.342 | 1.406 | 1.472 | 1.539 | 2.212 | 0.044 | 1.297 | 1.367 | 1.432 | 1.494 | 1.553 |
| 15.3 | 0.384 | 0.046 | 1.284 | 1.346 | 1.410 | 1.475 | 1.542 | 2.223 | 0.044 | 1.299 | 1.369 | 1.434 | 1.496 | 1.555 |
| 15.4 | 0.388 | 0.045 | 1.288 | 1.350 | 1.413 | 1.478 | 1.545 | 2.235 | 0.044 | 1.301 | 1.370 | 1.435 | 1.497 | 1.556 |
| 15.5 | 0.395 | 0.045 | 1.292 | 1.354 | 1.417 | 1.481 | 1.548 | 2.247 | 0.044 | 1.303 | 1.372 | 1.437 | 1.499 | 1.557 |
| 15.6 | 0.403 | 0.045 | 1.296 | 1.357 | 1.420 | 1.484 | 1.550 | 2.260 | 0.044 | 1.305 | 1.374 | 1.438 | 1.500 | 1.558 |
| 15.7 | 0.413 | 0.044 | 1.300 | 1.361 | 1.423 | 1.487 | 1.553 | 2.272 | 0.044 | 1.306 | 1.375 | 1.440 | 1.501 | 1.559 |
| 15.8 | 0.426 | 0.044 | 1.304 | 1.364 | 1.426 | 1.490 | 1.556 | 2.284 | 0.043 | 1.308 | 1.377 | 1.441 | 1.502 | 1.560 |
| 15.9 | 0.440 | 0.044 | 1.307 | 1.367 | 1.429 | 1.493 | 1.558 | 2.297 | 0.043 | 1.310 | 1.378 | 1.442 | 1.503 | 1.561 |
| 16.0 | 0.457 | 0.044 | 1.310 | 1.371 | 1.432 | 1.496 | 1.560 | 2.309 | 0.043 | 1.311 | 1.379 | 1.444 | 1.504 | 1.562 |
| 16.1 | 0.476 | 0.043 | 1.314 | 1.374 | 1.435 | 1.498 | 1.563 | 2.322 | 0.043 | 1.313 | 1.381 | 1.445 | 1.505 | 1.563 |
| 16.2 | 0.497 | 0.043 | 1.317 | 1.377 | 1.438 | 1.501 | 1.565 | 2.335 | 0.043 | 1.314 | 1.382 | 1.446 | 1.506 | 1.563 |
| 16.3 | 0.520 | 0.043 | 1.320 | 1.380 | 1.441 | 1.503 | 1.567 | 2.347 | 0.043 | 1.315 | 1.383 | 1.447 | 1.507 | 1.564 |
| 16.4 | 0.545 | 0.043 | 1.323 | 1.382 | 1.443 | 1.505 | 1.569 | 2.360 | 0.043 | 1.316 | 1.384 | 1.448 | 1.508 | 1.565 |
| 16.5 | 0.572 | 0.042 | 1.325 | 1.385 | 1.446 | 1.508 | 1.571 | 2.373 | 0.042 | 1.318 | 1.385 | 1.449 | 1.509 | 1.565 |
| 16.6 | 0.601 | 0.042 | 1.328 | 1.388 | 1.448 | 1.510 | 1.573 | 2.386 | 0.042 | 1.319 | 1.386 | 1.450 | 1.509 | 1.566 |
| 16.7 | 0.632 | 0.042 | 1.330 | 1.390 | 1.450 | 1.512 | 1.574 | 2.399 | 0.042 | 1.320 | 1.387 | 1.451 | 1.510 | 1.566 |
| 16.8 | 0.666 | 0.042 | 1.333 | 1.392 | 1.453 | 1.514 | 1.576 | 2.412 | 0.042 | 1.321 | 1.389 | 1.451 | 1.511 | 1.567 |
| 16.9 | 0.701 | 0.042 | 1.335 | 1.394 | 1.455 | 1.516 | 1.577 | 2.425 | 0.042 | 1.322 | 1.390 | 1.452 | 1.511 | 1.567 |
| 17.0 | 0.738 | 0.042 | 1.337 | 1.396 | 1.457 | 1.518 | 1.579 | 2.438 | 0.042 | 1.324 | 1.391 | 1.453 | 1.512 | 1.568 |
| 17.1 | 0.778 | 0.041 | 1.339 | 1.398 | 1.459 | 1.519 | 1.580 | 2.451 | 0.042 | 1.325 | 1.392 | 1.454 | 1.513 | 1.569 |
| 17.2 | 0.819 | 0.041 | 1.341 | 1.400 | 1.460 | 1.521 | 1.582 | 2.464 | 0.042 | 1.326 | 1.393 | 1.455 | 1.514 | 1.569 |
| 17.3 | 0.862 | 0.041 | 1.342 | 1.402 | 1.462 | 1.522 | 1.583 | 2.478 | 0.041 | 1.327 | 1.394 | 1.456 | 1.514 | 1.570 |
| 17.4 | 0.907 | 0.041 | 1.344 | 1.404 | 1.464 | 1.524 | 1.584 | 2.491 | 0.041 | 1.328 | 1.395 | 1.457 | 1.515 | 1.570 |
| 17.5 | 0.953 | 0.041 | 1.345 | 1.405 | 1.465 | 1.525 | 1.586 | 2.504 | 0.041 | 1.329 | 1.396 | 1.458 | 1.516 | 1.571 |
| 17.6 | 1.001 | 0.041 | 1.347 | 1.407 | 1.467 | 1.527 | 1.587 | 2.517 | 0.041 | 1.331 | 1.397 | 1.458 | 1.516 | 1.571 |
| 17.7 | 1.051 | 0.041 | 1.348 | 1.408 | 1.468 | 1.528 | 1.588 | 2.530 | 0.041 | 1.332 | 1.398 | 1.459 | 1.517 | 1.572 |
| 17.8 | 1.102 | 0.041 | 1.349 | 1.410 | 1.470 | 1.529 | 1.589 | 2.543 | 0.041 | 1.333 | 1.399 | 1.460 | 1.518 | 1.572 |
| 17.9 | 1.155 | 0.041 | 1.350 | 1.411 | 1.471 | 1.531 | 1.590 | 2.556 | 0.041 | 1.334 | 1.400 | 1.461 | 1.518 | 1.573 |
| 18.0 | 1.209 | 0.041 | 1.351 | 1.412 | 1.472 | 1.532 | 1.591 | 2.569 | 0.040 | 1.335 | 1.401 | 1.462 | 1.519 | 1.573 |
| 18.1 | 1.264 | 0.041 | 1.352 | 1.413 | 1.473 | 1.533 | 1.592 | 2.582 | 0.040 | 1.336 | 1.402 | 1.463 | 1.520 | 1.574 |
| 18.2 | 1.320 | 0.041 | 1.353 | 1.414 | 1.475 | 1.534 | 1.593 | 2.595 | 0.040 | 1.337 | 1.403 | 1.463 | 1.520 | 1.574 |
| 18.3 | 1.377 | 0.041 | 1.354 | 1.415 | 1.476 | 1.535 | 1.594 | 2.608 | 0.040 | 1.338 | 1.403 | 1.464 | 1.521 | 1.574 |
| 18.4 | 1.435 | 0.041 | 1.354 | 1.416 | 1.477 | 1.536 | 1.595 | 2.621 | 0.040 | 1.339 | 1.404 | 1.465 | 1.521 | 1.575 |
| 18.5 | 1.494 | 0.041 | 1.355 | 1.417 | 1.478 | 1.537 | 1.596 | 2.634 | 0.040 | 1.340 | 1.405 | 1.465 | 1.522 | 1.575 |
| 18.6 | 1.554 | 0.041 | 1.355 | 1.418 | 1.479 | 1.538 | 1.597 | 2.647 | 0.040 | 1.341 | 1.406 | 1.466 | 1.522 | 1.575 |
| 18.7 | 1.614 | 0.041 | 1.356 | 1.418 | 1.480 | 1.539 | 1.597 | 2.660 | 0.039 | 1.342 | 1.406 | 1.466 | 1.522 | 1.575 |
| 18.8 | 1.675 | 0.041 | 1.356 | 1.419 | 1.480 | 1.540 | 1.598 | 2.672 | 0.039 | 1.343 | 1.407 | 1.467 | 1.523 | 1.575 |
| 18.9 | 1.737 | 0.041 | 1.356 | 1.420 | 1.481 | 1.541 | 1.599 | 2.685 | 0.039 | 1.344 | 1.408 | 1.467 | 1.523 | 1.575 |
| 19.0 | 1.799 | 0.041 | 1.357 | 1.421 | 1.482 | 1.542 | 1.600 | 2.698 | 0.039 | 1.344 | 1.408 | 1.467 | 1.523 | 1.575 |
| 19.1 | 1.861 | 0.041 | 1.357 | 1.421 | 1.483 | 1.543 | 1.601 | 2.710 | 0.039 | 1.345 | 1.409 | 1.468 | 1.523 | 1.575 |
| 19.2 | 1.924 | 0.041 | 1.357 | 1.422 | 1.484 | 1.544 | 1.601 | 2.723 | 0.039 | 1.346 | 1.409 | 1.468 | 1.523 | 1.575 |
| 19.3 | 1.987 | 0.041 | 1.357 | 1.422 | 1.485 | 1.544 | 1.602 | 2.736 | 0.039 | 1.346 | 1.409 | 1.468 | 1.523 | 1.574 |
| 19.4 | 2.051 | 0.041 | 1.357 | 1.423 | 1.485 | 1.545 | 1.603 | 2.748 | 0.038 | 1.347 | 1.410 | 1.468 | 1.523 | 1.574 |
| 19.5 | 2.115 | 0.041 | 1.357 | 1.423 | 1.486 | 1.546 | 1.603 | 2.761 | 0.038 | 1.347 | 1.410 | 1.468 | 1.522 | 1.573 |
| 19.6 | 2.179 | 0.041 | 1.357 | 1.424 | 1.487 | 1.547 | 1.604 | 2.773 | 0.038 | 1.348 | 1.410 | 1.468 | 1.522 | 1.573 |
| 19.7 | 2.243 | 0.041 | 1.357 | 1.424 | 1.487 | 1.547 | 1.605 | 2.786 | 0.038 | 1.348 | 1.410 | 1.468 | 1.522 | 1.572 |
| 19.8 | 2.308 | 0.041 | 1.357 | 1.424 | 1.488 | 1.548 | 1.605 | 2.798 | 0.038 | 1.349 | 1.411 | 1.468 | 1.521 | 1.572 |
| 19.9 | 2.373 | 0.042 | 1.357 | 1.424 | 1.488 | 1.548 | 1.606 | 2.810 | 0.038 | 1.349 | 1.411 | 1.468 | 1.521 | 1.571 |
| 20.0 | 2.438 | 0.042 | 1.356 | 1.425 | 1.489 | 1.549 | 1.606 | 2.823 | 0.037 | 1.349 | 1.411 | 1.468 | 1.521 | 1.571 |

| L1 to L3 TBS | | | | | | | | | | | | | | |
| --- | --- | --- | --- | --- | --- | --- | --- | --- | --- | --- | --- | --- | --- | --- |
|  | Males | | | | | | | Females | | | | | | |
| Age | L | S | -2 SD | -1 SD | 0 SD (M) | 1 SD | 2 SD | L | S | -2 SD | -1 SD | 0 SD (M) | 1 SD | 2 SD |
| 5.0 | 3.659 | 0.059 | 1.071 | 1.170 | 1.251 | 1.319 | 1.380 | 1.853 | 0.053 | 1.090 | 1.161 | 1.228 | 1.292 | 1.353 |
| 5.1 | 3.617 | 0.059 | 1.073 | 1.171 | 1.251 | 1.320 | 1.381 | 1.851 | 0.054 | 1.090 | 1.160 | 1.228 | 1.292 | 1.354 |
| 5.2 | 3.574 | 0.059 | 1.074 | 1.172 | 1.252 | 1.321 | 1.382 | 1.849 | 0.054 | 1.089 | 1.160 | 1.228 | 1.292 | 1.354 |
| 5.3 | 3.532 | 0.059 | 1.076 | 1.173 | 1.253 | 1.322 | 1.382 | 1.846 | 0.054 | 1.089 | 1.160 | 1.228 | 1.292 | 1.354 |
| 5.4 | 3.490 | 0.059 | 1.077 | 1.173 | 1.253 | 1.322 | 1.383 | 1.844 | 0.054 | 1.088 | 1.160 | 1.227 | 1.292 | 1.355 |
| 5.5 | 3.448 | 0.059 | 1.078 | 1.174 | 1.254 | 1.323 | 1.384 | 1.841 | 0.054 | 1.088 | 1.159 | 1.227 | 1.292 | 1.355 |
| 5.6 | 3.406 | 0.059 | 1.080 | 1.175 | 1.255 | 1.324 | 1.385 | 1.839 | 0.054 | 1.087 | 1.159 | 1.227 | 1.293 | 1.355 |
| 5.7 | 3.365 | 0.059 | 1.081 | 1.176 | 1.256 | 1.325 | 1.386 | 1.837 | 0.055 | 1.086 | 1.159 | 1.227 | 1.293 | 1.356 |
| 5.8 | 3.323 | 0.059 | 1.083 | 1.177 | 1.256 | 1.325 | 1.387 | 1.834 | 0.055 | 1.086 | 1.158 | 1.227 | 1.293 | 1.356 |
| 5.9 | 3.282 | 0.059 | 1.084 | 1.178 | 1.257 | 1.326 | 1.388 | 1.832 | 0.055 | 1.085 | 1.158 | 1.227 | 1.293 | 1.356 |
| 6.0 | 3.240 | 0.059 | 1.085 | 1.178 | 1.257 | 1.327 | 1.389 | 1.830 | 0.055 | 1.085 | 1.158 | 1.227 | 1.293 | 1.356 |
| 6.1 | 3.199 | 0.059 | 1.086 | 1.179 | 1.258 | 1.327 | 1.390 | 1.827 | 0.055 | 1.084 | 1.157 | 1.227 | 1.293 | 1.357 |
| 6.2 | 3.159 | 0.059 | 1.088 | 1.180 | 1.259 | 1.328 | 1.391 | 1.825 | 0.055 | 1.084 | 1.157 | 1.227 | 1.293 | 1.357 |
| 6.3 | 3.118 | 0.058 | 1.089 | 1.180 | 1.259 | 1.329 | 1.391 | 1.822 | 0.056 | 1.083 | 1.157 | 1.227 | 1.293 | 1.357 |
| 6.4 | 3.077 | 0.058 | 1.090 | 1.181 | 1.260 | 1.329 | 1.392 | 1.820 | 0.056 | 1.083 | 1.156 | 1.226 | 1.293 | 1.358 |
| 6.5 | 3.037 | 0.058 | 1.091 | 1.182 | 1.260 | 1.330 | 1.393 | 1.817 | 0.056 | 1.082 | 1.156 | 1.226 | 1.293 | 1.358 |
| 6.6 | 2.997 | 0.058 | 1.092 | 1.182 | 1.261 | 1.330 | 1.394 | 1.815 | 0.056 | 1.082 | 1.156 | 1.226 | 1.294 | 1.358 |
| 6.7 | 2.956 | 0.058 | 1.093 | 1.183 | 1.261 | 1.331 | 1.394 | 1.812 | 0.056 | 1.081 | 1.156 | 1.226 | 1.294 | 1.358 |
| 6.8 | 2.916 | 0.058 | 1.094 | 1.184 | 1.262 | 1.331 | 1.395 | 1.810 | 0.056 | 1.081 | 1.155 | 1.226 | 1.294 | 1.359 |
| 6.9 | 2.876 | 0.058 | 1.095 | 1.184 | 1.262 | 1.332 | 1.396 | 1.807 | 0.057 | 1.081 | 1.155 | 1.226 | 1.294 | 1.359 |
| 7.0 | 2.837 | 0.058 | 1.096 | 1.185 | 1.262 | 1.332 | 1.396 | 1.804 | 0.057 | 1.080 | 1.155 | 1.226 | 1.294 | 1.359 |
| 7.1 | 2.797 | 0.058 | 1.097 | 1.185 | 1.263 | 1.333 | 1.397 | 1.801 | 0.057 | 1.080 | 1.155 | 1.226 | 1.294 | 1.360 |
| 7.2 | 2.757 | 0.058 | 1.098 | 1.185 | 1.263 | 1.333 | 1.397 | 1.799 | 0.057 | 1.080 | 1.155 | 1.226 | 1.294 | 1.360 |
| 7.3 | 2.718 | 0.058 | 1.098 | 1.186 | 1.263 | 1.334 | 1.398 | 1.796 | 0.057 | 1.079 | 1.155 | 1.226 | 1.295 | 1.360 |
| 7.4 | 2.679 | 0.058 | 1.099 | 1.186 | 1.264 | 1.334 | 1.398 | 1.793 | 0.057 | 1.079 | 1.155 | 1.226 | 1.295 | 1.361 |
| 7.5 | 2.640 | 0.058 | 1.100 | 1.187 | 1.264 | 1.334 | 1.399 | 1.790 | 0.057 | 1.079 | 1.155 | 1.226 | 1.295 | 1.361 |
| 7.6 | 2.601 | 0.058 | 1.101 | 1.187 | 1.264 | 1.334 | 1.399 | 1.787 | 0.057 | 1.079 | 1.155 | 1.227 | 1.295 | 1.361 |
| 7.7 | 2.562 | 0.058 | 1.101 | 1.187 | 1.264 | 1.335 | 1.400 | 1.784 | 0.057 | 1.079 | 1.155 | 1.227 | 1.296 | 1.362 |
| 7.8 | 2.523 | 0.058 | 1.102 | 1.187 | 1.265 | 1.335 | 1.400 | 1.781 | 0.057 | 1.079 | 1.155 | 1.227 | 1.296 | 1.362 |
| 7.9 | 2.484 | 0.058 | 1.103 | 1.188 | 1.265 | 1.335 | 1.401 | 1.778 | 0.058 | 1.079 | 1.155 | 1.227 | 1.296 | 1.362 |
| 8.0 | 2.446 | 0.058 | 1.103 | 1.188 | 1.265 | 1.335 | 1.401 | 1.775 | 0.058 | 1.079 | 1.155 | 1.227 | 1.296 | 1.363 |
| 8.1 | 2.408 | 0.058 | 1.104 | 1.188 | 1.265 | 1.336 | 1.401 | 1.772 | 0.058 | 1.079 | 1.155 | 1.227 | 1.297 | 1.363 |
| 8.2 | 2.370 | 0.058 | 1.104 | 1.188 | 1.265 | 1.336 | 1.402 | 1.769 | 0.058 | 1.079 | 1.155 | 1.228 | 1.297 | 1.364 |
| 8.3 | 2.332 | 0.058 | 1.105 | 1.189 | 1.265 | 1.336 | 1.402 | 1.766 | 0.058 | 1.079 | 1.155 | 1.228 | 1.298 | 1.364 |
| 8.4 | 2.294 | 0.058 | 1.106 | 1.189 | 1.265 | 1.336 | 1.402 | 1.763 | 0.058 | 1.079 | 1.156 | 1.228 | 1.298 | 1.365 |
| 8.5 | 2.257 | 0.058 | 1.106 | 1.189 | 1.265 | 1.336 | 1.403 | 1.760 | 0.058 | 1.079 | 1.156 | 1.229 | 1.298 | 1.365 |
| 8.6 | 2.220 | 0.058 | 1.107 | 1.189 | 1.265 | 1.336 | 1.403 | 1.757 | 0.058 | 1.080 | 1.156 | 1.229 | 1.299 | 1.366 |
| 8.7 | 2.183 | 0.058 | 1.107 | 1.189 | 1.265 | 1.336 | 1.403 | 1.754 | 0.058 | 1.080 | 1.157 | 1.230 | 1.300 | 1.367 |
| 8.8 | 2.146 | 0.058 | 1.108 | 1.190 | 1.266 | 1.337 | 1.404 | 1.751 | 0.058 | 1.080 | 1.157 | 1.230 | 1.300 | 1.368 |
| 8.9 | 2.109 | 0.058 | 1.108 | 1.190 | 1.266 | 1.337 | 1.404 | 1.748 | 0.058 | 1.081 | 1.158 | 1.231 | 1.301 | 1.368 |
| 9.0 | 2.073 | 0.058 | 1.108 | 1.190 | 1.266 | 1.337 | 1.404 | 1.745 | 0.058 | 1.081 | 1.158 | 1.232 | 1.302 | 1.369 |
| 9.1 | 2.037 | 0.058 | 1.109 | 1.190 | 1.266 | 1.337 | 1.405 | 1.742 | 0.058 | 1.082 | 1.159 | 1.232 | 1.303 | 1.370 |
| 9.2 | 2.000 | 0.058 | 1.109 | 1.190 | 1.266 | 1.337 | 1.405 | 1.739 | 0.058 | 1.083 | 1.160 | 1.233 | 1.304 | 1.371 |
| 9.3 | 1.964 | 0.058 | 1.110 | 1.190 | 1.266 | 1.337 | 1.405 | 1.736 | 0.058 | 1.083 | 1.161 | 1.234 | 1.305 | 1.372 |
| 9.4 | 1.929 | 0.058 | 1.110 | 1.190 | 1.266 | 1.337 | 1.405 | 1.734 | 0.058 | 1.084 | 1.162 | 1.235 | 1.306 | 1.374 |
| 9.5 | 1.893 | 0.058 | 1.111 | 1.190 | 1.266 | 1.337 | 1.406 | 1.731 | 0.058 | 1.085 | 1.163 | 1.236 | 1.307 | 1.375 |
| 9.6 | 1.857 | 0.058 | 1.111 | 1.191 | 1.266 | 1.337 | 1.406 | 1.728 | 0.058 | 1.086 | 1.164 | 1.237 | 1.308 | 1.376 |
| 9.7 | 1.822 | 0.058 | 1.111 | 1.191 | 1.266 | 1.338 | 1.406 | 1.726 | 0.058 | 1.087 | 1.165 | 1.239 | 1.310 | 1.378 |
| 9.8 | 1.786 | 0.058 | 1.112 | 1.191 | 1.266 | 1.338 | 1.407 | 1.723 | 0.058 | 1.089 | 1.166 | 1.240 | 1.311 | 1.379 |
| 9.9 | 1.751 | 0.058 | 1.112 | 1.191 | 1.266 | 1.338 | 1.407 | 1.721 | 0.058 | 1.090 | 1.168 | 1.242 | 1.313 | 1.381 |
| 10.0 | 1.715 | 0.058 | 1.112 | 1.191 | 1.266 | 1.338 | 1.407 | 1.719 | 0.058 | 1.091 | 1.169 | 1.243 | 1.314 | 1.383 |
| 10.1 | 1.680 | 0.058 | 1.113 | 1.191 | 1.266 | 1.338 | 1.407 | 1.717 | 0.058 | 1.093 | 1.171 | 1.245 | 1.316 | 1.385 |
| 10.2 | 1.644 | 0.058 | 1.113 | 1.191 | 1.266 | 1.338 | 1.408 | 1.715 | 0.058 | 1.094 | 1.172 | 1.247 | 1.318 | 1.387 |
| 10.3 | 1.609 | 0.058 | 1.114 | 1.191 | 1.266 | 1.338 | 1.408 | 1.713 | 0.058 | 1.096 | 1.174 | 1.249 | 1.320 | 1.389 |
| 10.4 | 1.573 | 0.058 | 1.114 | 1.192 | 1.266 | 1.339 | 1.409 | 1.711 | 0.058 | 1.098 | 1.176 | 1.251 | 1.322 | 1.391 |
| 10.5 | 1.538 | 0.058 | 1.115 | 1.192 | 1.267 | 1.339 | 1.409 | 1.709 | 0.058 | 1.100 | 1.178 | 1.253 | 1.325 | 1.394 |
| 10.6 | 1.502 | 0.058 | 1.115 | 1.192 | 1.267 | 1.339 | 1.410 | 1.707 | 0.058 | 1.102 | 1.180 | 1.255 | 1.327 | 1.396 |
| 10.7 | 1.466 | 0.058 | 1.116 | 1.192 | 1.267 | 1.340 | 1.410 | 1.706 | 0.058 | 1.104 | 1.183 | 1.258 | 1.330 | 1.399 |
| 10.8 | 1.430 | 0.058 | 1.116 | 1.193 | 1.267 | 1.340 | 1.411 | 1.705 | 0.058 | 1.107 | 1.185 | 1.260 | 1.332 | 1.402 |
| 10.9 | 1.394 | 0.058 | 1.117 | 1.193 | 1.268 | 1.340 | 1.412 | 1.703 | 0.058 | 1.109 | 1.188 | 1.263 | 1.335 | 1.405 |
| 11.0 | 1.359 | 0.058 | 1.118 | 1.194 | 1.268 | 1.341 | 1.412 | 1.702 | 0.058 | 1.112 | 1.191 | 1.266 | 1.338 | 1.408 |
| 11.1 | 1.323 | 0.058 | 1.119 | 1.194 | 1.269 | 1.341 | 1.413 | 1.701 | 0.058 | 1.115 | 1.194 | 1.269 | 1.341 | 1.411 |
| 11.2 | 1.287 | 0.058 | 1.119 | 1.195 | 1.269 | 1.342 | 1.414 | 1.700 | 0.058 | 1.118 | 1.197 | 1.272 | 1.345 | 1.414 |
| 11.3 | 1.252 | 0.058 | 1.120 | 1.196 | 1.270 | 1.343 | 1.415 | 1.699 | 0.058 | 1.121 | 1.200 | 1.276 | 1.348 | 1.418 |
| 11.4 | 1.216 | 0.058 | 1.121 | 1.196 | 1.270 | 1.343 | 1.416 | 1.699 | 0.058 | 1.125 | 1.204 | 1.279 | 1.352 | 1.422 |
| 11.5 | 1.181 | 0.058 | 1.122 | 1.197 | 1.271 | 1.344 | 1.417 | 1.698 | 0.058 | 1.128 | 1.207 | 1.283 | 1.355 | 1.425 |
| 11.6 | 1.146 | 0.058 | 1.124 | 1.198 | 1.272 | 1.345 | 1.418 | 1.698 | 0.058 | 1.132 | 1.211 | 1.286 | 1.359 | 1.429 |
| 11.7 | 1.111 | 0.058 | 1.125 | 1.199 | 1.273 | 1.346 | 1.419 | 1.698 | 0.057 | 1.135 | 1.215 | 1.290 | 1.363 | 1.433 |
| 11.8 | 1.076 | 0.058 | 1.126 | 1.200 | 1.274 | 1.347 | 1.420 | 1.698 | 0.057 | 1.139 | 1.219 | 1.294 | 1.367 | 1.437 |
| 11.9 | 1.041 | 0.058 | 1.128 | 1.201 | 1.275 | 1.348 | 1.422 | 1.698 | 0.057 | 1.143 | 1.223 | 1.298 | 1.371 | 1.442 |
| 12.0 | 1.007 | 0.058 | 1.129 | 1.203 | 1.276 | 1.350 | 1.423 | 1.698 | 0.057 | 1.148 | 1.227 | 1.303 | 1.376 | 1.446 |
| 12.1 | 0.973 | 0.057 | 1.131 | 1.204 | 1.277 | 1.351 | 1.425 | 1.698 | 0.057 | 1.152 | 1.231 | 1.307 | 1.380 | 1.450 |
| 12.2 | 0.939 | 0.057 | 1.133 | 1.206 | 1.279 | 1.352 | 1.426 | 1.698 | 0.057 | 1.156 | 1.235 | 1.311 | 1.384 | 1.455 |
| 12.3 | 0.906 | 0.057 | 1.135 | 1.207 | 1.280 | 1.354 | 1.428 | 1.699 | 0.057 | 1.161 | 1.240 | 1.316 | 1.389 | 1.459 |
| 12.4 | 0.873 | 0.057 | 1.137 | 1.209 | 1.282 | 1.356 | 1.430 | 1.700 | 0.056 | 1.165 | 1.244 | 1.320 | 1.393 | 1.464 |
| 12.5 | 0.840 | 0.057 | 1.139 | 1.211 | 1.284 | 1.358 | 1.432 | 1.701 | 0.056 | 1.170 | 1.249 | 1.325 | 1.398 | 1.468 |
| 12.6 | 0.808 | 0.057 | 1.141 | 1.213 | 1.286 | 1.360 | 1.434 | 1.702 | 0.056 | 1.174 | 1.253 | 1.329 | 1.402 | 1.472 |
| 12.7 | 0.776 | 0.057 | 1.144 | 1.215 | 1.288 | 1.362 | 1.436 | 1.703 | 0.056 | 1.179 | 1.258 | 1.334 | 1.407 | 1.477 |
| 12.8 | 0.745 | 0.057 | 1.146 | 1.218 | 1.290 | 1.364 | 1.439 | 1.704 | 0.055 | 1.183 | 1.262 | 1.338 | 1.411 | 1.481 |
| 12.9 | 0.714 | 0.057 | 1.149 | 1.220 | 1.293 | 1.366 | 1.441 | 1.705 | 0.055 | 1.188 | 1.267 | 1.342 | 1.415 | 1.485 |
| 13.0 | 0.684 | 0.056 | 1.152 | 1.223 | 1.295 | 1.369 | 1.444 | 1.707 | 0.055 | 1.192 | 1.271 | 1.347 | 1.419 | 1.490 |
| 13.1 | 0.655 | 0.056 | 1.155 | 1.226 | 1.298 | 1.371 | 1.446 | 1.708 | 0.055 | 1.197 | 1.275 | 1.351 | 1.423 | 1.494 |
| 13.2 | 0.627 | 0.056 | 1.158 | 1.228 | 1.300 | 1.374 | 1.449 | 1.710 | 0.055 | 1.201 | 1.280 | 1.355 | 1.427 | 1.497 |
| 13.3 | 0.599 | 0.056 | 1.161 | 1.231 | 1.303 | 1.377 | 1.452 | 1.712 | 0.054 | 1.205 | 1.284 | 1.359 | 1.431 | 1.501 |
| 13.4 | 0.572 | 0.056 | 1.165 | 1.235 | 1.306 | 1.380 | 1.455 | 1.714 | 0.054 | 1.209 | 1.288 | 1.363 | 1.435 | 1.505 |
| 13.5 | 0.546 | 0.055 | 1.168 | 1.238 | 1.309 | 1.383 | 1.458 | 1.716 | 0.054 | 1.213 | 1.292 | 1.367 | 1.439 | 1.508 |
| 13.6 | 0.521 | 0.055 | 1.172 | 1.241 | 1.313 | 1.386 | 1.461 | 1.718 | 0.054 | 1.217 | 1.295 | 1.370 | 1.442 | 1.512 |
| 13.7 | 0.497 | 0.055 | 1.175 | 1.245 | 1.316 | 1.389 | 1.465 | 1.720 | 0.053 | 1.221 | 1.299 | 1.374 | 1.446 | 1.515 |
| 13.8 | 0.473 | 0.055 | 1.179 | 1.248 | 1.319 | 1.393 | 1.468 | 1.723 | 0.053 | 1.225 | 1.303 | 1.377 | 1.449 | 1.518 |
| 13.9 | 0.451 | 0.054 | 1.183 | 1.252 | 1.323 | 1.396 | 1.471 | 1.726 | 0.053 | 1.229 | 1.306 | 1.380 | 1.452 | 1.521 |
| 14.0 | 0.431 | 0.054 | 1.187 | 1.256 | 1.326 | 1.399 | 1.475 | 1.728 | 0.053 | 1.232 | 1.309 | 1.383 | 1.455 | 1.524 |
| 14.1 | 0.411 | 0.054 | 1.191 | 1.259 | 1.330 | 1.403 | 1.478 | 1.731 | 0.052 | 1.235 | 1.312 | 1.386 | 1.458 | 1.526 |
| 14.2 | 0.393 | 0.054 | 1.195 | 1.263 | 1.334 | 1.406 | 1.482 | 1.734 | 0.052 | 1.239 | 1.315 | 1.389 | 1.460 | 1.529 |
| 14.3 | 0.376 | 0.053 | 1.199 | 1.267 | 1.337 | 1.410 | 1.485 | 1.737 | 0.052 | 1.242 | 1.318 | 1.392 | 1.463 | 1.531 |
| 14.4 | 0.360 | 0.053 | 1.203 | 1.271 | 1.341 | 1.413 | 1.488 | 1.740 | 0.052 | 1.245 | 1.321 | 1.395 | 1.465 | 1.533 |
| 14.5 | 0.346 | 0.053 | 1.207 | 1.275 | 1.345 | 1.417 | 1.492 | 1.744 | 0.051 | 1.248 | 1.324 | 1.397 | 1.467 | 1.535 |
| 14.6 | 0.333 | 0.053 | 1.211 | 1.279 | 1.348 | 1.420 | 1.495 | 1.747 | 0.051 | 1.250 | 1.326 | 1.399 | 1.470 | 1.538 |
| 14.7 | 0.321 | 0.052 | 1.216 | 1.283 | 1.352 | 1.424 | 1.499 | 1.750 | 0.051 | 1.253 | 1.329 | 1.402 | 1.472 | 1.539 |
| 14.8 | 0.311 | 0.052 | 1.220 | 1.287 | 1.356 | 1.428 | 1.502 | 1.754 | 0.051 | 1.256 | 1.331 | 1.404 | 1.474 | 1.541 |
| 14.9 | 0.302 | 0.052 | 1.224 | 1.290 | 1.359 | 1.431 | 1.505 | 1.758 | 0.051 | 1.258 | 1.334 | 1.406 | 1.476 | 1.543 |
| 15.0 | 0.295 | 0.051 | 1.228 | 1.294 | 1.363 | 1.434 | 1.508 | 1.761 | 0.050 | 1.260 | 1.336 | 1.408 | 1.478 | 1.545 |
| 15.1 | 0.290 | 0.051 | 1.232 | 1.298 | 1.367 | 1.438 | 1.512 | 1.765 | 0.050 | 1.263 | 1.338 | 1.410 | 1.479 | 1.546 |
| 15.2 | 0.286 | 0.051 | 1.236 | 1.302 | 1.370 | 1.441 | 1.515 | 1.769 | 0.050 | 1.265 | 1.340 | 1.412 | 1.481 | 1.548 |
| 15.3 | 0.283 | 0.051 | 1.240 | 1.306 | 1.374 | 1.445 | 1.518 | 1.773 | 0.050 | 1.267 | 1.342 | 1.414 | 1.483 | 1.549 |
| 15.4 | 0.282 | 0.050 | 1.244 | 1.309 | 1.377 | 1.448 | 1.521 | 1.778 | 0.050 | 1.269 | 1.344 | 1.415 | 1.484 | 1.551 |
| 15.5 | 0.283 | 0.050 | 1.248 | 1.313 | 1.381 | 1.451 | 1.524 | 1.782 | 0.049 | 1.271 | 1.346 | 1.417 | 1.486 | 1.552 |
| 15.6 | 0.285 | 0.050 | 1.252 | 1.317 | 1.384 | 1.454 | 1.526 | 1.786 | 0.049 | 1.273 | 1.348 | 1.419 | 1.487 | 1.553 |
| 15.7 | 0.289 | 0.049 | 1.255 | 1.320 | 1.388 | 1.457 | 1.529 | 1.791 | 0.049 | 1.275 | 1.349 | 1.420 | 1.489 | 1.555 |
| 15.8 | 0.295 | 0.049 | 1.259 | 1.324 | 1.391 | 1.460 | 1.532 | 1.795 | 0.049 | 1.277 | 1.351 | 1.422 | 1.490 | 1.556 |
| 15.9 | 0.302 | 0.049 | 1.263 | 1.327 | 1.394 | 1.463 | 1.534 | 1.800 | 0.049 | 1.279 | 1.352 | 1.423 | 1.491 | 1.557 |
| 16.0 | 0.311 | 0.048 | 1.266 | 1.331 | 1.397 | 1.466 | 1.537 | 1.804 | 0.049 | 1.280 | 1.354 | 1.425 | 1.493 | 1.558 |
| 16.1 | 0.322 | 0.048 | 1.270 | 1.334 | 1.400 | 1.469 | 1.539 | 1.809 | 0.048 | 1.282 | 1.356 | 1.426 | 1.494 | 1.559 |
| 16.2 | 0.334 | 0.048 | 1.273 | 1.337 | 1.403 | 1.471 | 1.542 | 1.814 | 0.048 | 1.283 | 1.357 | 1.427 | 1.495 | 1.560 |
| 16.3 | 0.348 | 0.048 | 1.276 | 1.340 | 1.406 | 1.474 | 1.544 | 1.819 | 0.048 | 1.285 | 1.358 | 1.429 | 1.496 | 1.561 |
| 16.4 | 0.364 | 0.047 | 1.279 | 1.343 | 1.409 | 1.476 | 1.546 | 1.824 | 0.048 | 1.287 | 1.360 | 1.430 | 1.497 | 1.562 |
| 16.5 | 0.381 | 0.047 | 1.282 | 1.346 | 1.411 | 1.479 | 1.548 | 1.829 | 0.048 | 1.288 | 1.361 | 1.431 | 1.498 | 1.563 |
| 16.6 | 0.400 | 0.047 | 1.285 | 1.349 | 1.414 | 1.481 | 1.550 | 1.834 | 0.048 | 1.289 | 1.362 | 1.432 | 1.499 | 1.564 |
| 16.7 | 0.420 | 0.047 | 1.288 | 1.352 | 1.417 | 1.483 | 1.552 | 1.839 | 0.048 | 1.291 | 1.364 | 1.433 | 1.500 | 1.565 |
| 16.8 | 0.443 | 0.046 | 1.291 | 1.354 | 1.419 | 1.486 | 1.554 | 1.844 | 0.047 | 1.292 | 1.365 | 1.434 | 1.501 | 1.566 |
| 16.9 | 0.467 | 0.046 | 1.293 | 1.357 | 1.421 | 1.488 | 1.556 | 1.849 | 0.047 | 1.294 | 1.366 | 1.435 | 1.502 | 1.566 |
| 17.0 | 0.492 | 0.046 | 1.296 | 1.359 | 1.424 | 1.490 | 1.557 | 1.855 | 0.047 | 1.295 | 1.367 | 1.437 | 1.503 | 1.567 |
| 17.1 | 0.520 | 0.046 | 1.298 | 1.361 | 1.426 | 1.492 | 1.559 | 1.860 | 0.047 | 1.296 | 1.369 | 1.438 | 1.504 | 1.568 |
| 17.2 | 0.549 | 0.045 | 1.301 | 1.364 | 1.428 | 1.493 | 1.560 | 1.865 | 0.047 | 1.298 | 1.370 | 1.439 | 1.505 | 1.569 |
| 17.3 | 0.579 | 0.045 | 1.303 | 1.366 | 1.430 | 1.495 | 1.562 | 1.871 | 0.047 | 1.299 | 1.371 | 1.440 | 1.506 | 1.569 |
| 17.4 | 0.611 | 0.045 | 1.305 | 1.368 | 1.432 | 1.497 | 1.563 | 1.876 | 0.047 | 1.300 | 1.372 | 1.441 | 1.507 | 1.570 |
| 17.5 | 0.645 | 0.045 | 1.307 | 1.370 | 1.433 | 1.498 | 1.564 | 1.882 | 0.046 | 1.302 | 1.373 | 1.442 | 1.507 | 1.571 |
| 17.6 | 0.680 | 0.045 | 1.309 | 1.371 | 1.435 | 1.500 | 1.566 | 1.887 | 0.046 | 1.303 | 1.374 | 1.443 | 1.508 | 1.571 |
| 17.7 | 0.716 | 0.045 | 1.310 | 1.373 | 1.437 | 1.501 | 1.567 | 1.893 | 0.046 | 1.304 | 1.375 | 1.444 | 1.509 | 1.572 |
| 17.8 | 0.754 | 0.044 | 1.312 | 1.375 | 1.438 | 1.503 | 1.568 | 1.898 | 0.046 | 1.305 | 1.377 | 1.445 | 1.510 | 1.573 |
| 17.9 | 0.793 | 0.044 | 1.313 | 1.376 | 1.440 | 1.504 | 1.569 | 1.904 | 0.046 | 1.307 | 1.378 | 1.445 | 1.510 | 1.573 |
| 18.0 | 0.834 | 0.044 | 1.315 | 1.378 | 1.441 | 1.505 | 1.570 | 1.910 | 0.046 | 1.308 | 1.379 | 1.446 | 1.511 | 1.574 |
| 18.1 | 0.875 | 0.044 | 1.316 | 1.379 | 1.443 | 1.506 | 1.571 | 1.915 | 0.046 | 1.309 | 1.380 | 1.447 | 1.512 | 1.574 |
| 18.2 | 0.918 | 0.044 | 1.317 | 1.380 | 1.444 | 1.508 | 1.571 | 1.921 | 0.045 | 1.310 | 1.381 | 1.448 | 1.512 | 1.574 |
| 18.3 | 0.962 | 0.044 | 1.318 | 1.382 | 1.445 | 1.509 | 1.572 | 1.926 | 0.045 | 1.311 | 1.381 | 1.448 | 1.513 | 1.575 |
| 18.4 | 1.006 | 0.044 | 1.320 | 1.383 | 1.446 | 1.510 | 1.573 | 1.932 | 0.045 | 1.312 | 1.382 | 1.449 | 1.513 | 1.575 |
| 18.5 | 1.052 | 0.044 | 1.321 | 1.384 | 1.447 | 1.511 | 1.574 | 1.938 | 0.045 | 1.313 | 1.383 | 1.450 | 1.514 | 1.575 |
| 18.6 | 1.099 | 0.044 | 1.322 | 1.385 | 1.448 | 1.512 | 1.574 | 1.943 | 0.045 | 1.314 | 1.384 | 1.450 | 1.514 | 1.575 |
| 18.7 | 1.147 | 0.044 | 1.322 | 1.386 | 1.450 | 1.512 | 1.575 | 1.949 | 0.045 | 1.315 | 1.384 | 1.451 | 1.514 | 1.575 |
| 18.8 | 1.195 | 0.043 | 1.323 | 1.387 | 1.451 | 1.513 | 1.576 | 1.955 | 0.045 | 1.316 | 1.385 | 1.451 | 1.514 | 1.575 |
| 18.9 | 1.244 | 0.043 | 1.324 | 1.388 | 1.452 | 1.514 | 1.576 | 1.960 | 0.044 | 1.317 | 1.386 | 1.451 | 1.514 | 1.575 |
| 19.0 | 1.294 | 0.043 | 1.325 | 1.389 | 1.452 | 1.515 | 1.577 | 1.966 | 0.044 | 1.317 | 1.386 | 1.452 | 1.514 | 1.575 |
| 19.1 | 1.345 | 0.043 | 1.325 | 1.390 | 1.453 | 1.516 | 1.578 | 1.971 | 0.044 | 1.318 | 1.387 | 1.452 | 1.514 | 1.575 |
| 19.2 | 1.396 | 0.043 | 1.326 | 1.391 | 1.454 | 1.517 | 1.578 | 1.977 | 0.044 | 1.319 | 1.387 | 1.452 | 1.514 | 1.574 |
| 19.3 | 1.448 | 0.043 | 1.327 | 1.392 | 1.455 | 1.518 | 1.579 | 1.982 | 0.044 | 1.319 | 1.387 | 1.452 | 1.514 | 1.574 |
| 19.4 | 1.500 | 0.043 | 1.327 | 1.392 | 1.456 | 1.518 | 1.579 | 1.988 | 0.044 | 1.320 | 1.387 | 1.452 | 1.514 | 1.574 |
| 19.5 | 1.553 | 0.043 | 1.328 | 1.393 | 1.457 | 1.519 | 1.580 | 1.994 | 0.043 | 1.320 | 1.388 | 1.452 | 1.514 | 1.573 |
| 19.6 | 1.606 | 0.043 | 1.328 | 1.394 | 1.458 | 1.520 | 1.580 | 1.999 | 0.043 | 1.321 | 1.388 | 1.452 | 1.514 | 1.573 |
| 19.7 | 1.660 | 0.043 | 1.329 | 1.395 | 1.458 | 1.520 | 1.581 | 2.005 | 0.043 | 1.321 | 1.388 | 1.452 | 1.513 | 1.572 |
| 19.8 | 1.714 | 0.043 | 1.329 | 1.395 | 1.459 | 1.521 | 1.581 | 2.010 | 0.043 | 1.322 | 1.388 | 1.452 | 1.513 | 1.571 |
| 19.9 | 1.769 | 0.043 | 1.329 | 1.396 | 1.460 | 1.522 | 1.582 | 2.016 | 0.043 | 1.322 | 1.388 | 1.452 | 1.512 | 1.571 |
| 20.0 | 1.824 | 0.043 | 1.330 | 1.396 | 1.460 | 1.522 | 1.582 | 2.021 | 0.043 | 1.322 | 1.389 | 1.452 | 1.512 | 1.570 |

| L1 to L2 TBS | | | | | | | | | | | | | | |
| --- | --- | --- | --- | --- | --- | --- | --- | --- | --- | --- | --- | --- | --- | --- |
|  | Males | | | | | | | Females | | | | | | |
| Age | L | S | -2 SD | -1 SD | 0 SD (M) | 1 SD | 2 SD | L | S | -2 SD | -1 SD | 0 SD (M) | 1 SD | 2 SD |
| 5.0 | 2.425 | 0.068 | 1.038 | 1.137 | 1.226 | 1.306 | 1.379 | 2.322 | 0.060 | 1.044 | 1.126 | 1.202 | 1.271 | 1.336 |
| 5.1 | 2.425 | 0.068 | 1.039 | 1.138 | 1.226 | 1.306 | 1.380 | 2.314 | 0.060 | 1.044 | 1.126 | 1.202 | 1.271 | 1.336 |
| 5.2 | 2.426 | 0.068 | 1.040 | 1.139 | 1.227 | 1.307 | 1.381 | 2.306 | 0.060 | 1.044 | 1.126 | 1.202 | 1.272 | 1.337 |
| 5.3 | 2.426 | 0.068 | 1.040 | 1.140 | 1.228 | 1.308 | 1.381 | 2.297 | 0.061 | 1.043 | 1.127 | 1.202 | 1.273 | 1.338 |
| 5.4 | 2.425 | 0.068 | 1.041 | 1.140 | 1.228 | 1.309 | 1.382 | 2.289 | 0.061 | 1.043 | 1.127 | 1.203 | 1.273 | 1.339 |
| 5.5 | 2.425 | 0.068 | 1.042 | 1.141 | 1.229 | 1.309 | 1.383 | 2.281 | 0.061 | 1.043 | 1.127 | 1.203 | 1.274 | 1.340 |
| 5.6 | 2.425 | 0.068 | 1.042 | 1.141 | 1.230 | 1.310 | 1.384 | 2.272 | 0.061 | 1.043 | 1.126 | 1.203 | 1.274 | 1.340 |
| 5.7 | 2.424 | 0.068 | 1.043 | 1.142 | 1.230 | 1.311 | 1.384 | 2.264 | 0.061 | 1.042 | 1.126 | 1.203 | 1.275 | 1.341 |
| 5.8 | 2.423 | 0.068 | 1.043 | 1.143 | 1.231 | 1.311 | 1.385 | 2.255 | 0.062 | 1.042 | 1.126 | 1.204 | 1.275 | 1.342 |
| 5.9 | 2.422 | 0.068 | 1.044 | 1.143 | 1.232 | 1.312 | 1.386 | 2.247 | 0.062 | 1.042 | 1.126 | 1.204 | 1.275 | 1.342 |
| 6.0 | 2.421 | 0.068 | 1.045 | 1.144 | 1.232 | 1.312 | 1.386 | 2.239 | 0.062 | 1.041 | 1.126 | 1.204 | 1.276 | 1.343 |
| 6.1 | 2.419 | 0.068 | 1.045 | 1.145 | 1.233 | 1.313 | 1.387 | 2.230 | 0.062 | 1.041 | 1.126 | 1.204 | 1.276 | 1.344 |
| 6.2 | 2.417 | 0.068 | 1.046 | 1.145 | 1.233 | 1.314 | 1.387 | 2.222 | 0.062 | 1.041 | 1.126 | 1.204 | 1.277 | 1.344 |
| 6.3 | 2.414 | 0.068 | 1.047 | 1.146 | 1.234 | 1.314 | 1.388 | 2.213 | 0.063 | 1.040 | 1.126 | 1.204 | 1.277 | 1.345 |
| 6.4 | 2.411 | 0.068 | 1.047 | 1.146 | 1.235 | 1.315 | 1.389 | 2.205 | 0.063 | 1.040 | 1.126 | 1.204 | 1.277 | 1.345 |
| 6.5 | 2.408 | 0.068 | 1.048 | 1.147 | 1.235 | 1.315 | 1.389 | 2.196 | 0.063 | 1.040 | 1.126 | 1.204 | 1.277 | 1.346 |
| 6.6 | 2.404 | 0.068 | 1.048 | 1.147 | 1.236 | 1.316 | 1.390 | 2.188 | 0.063 | 1.039 | 1.125 | 1.204 | 1.278 | 1.346 |
| 6.7 | 2.399 | 0.068 | 1.049 | 1.148 | 1.236 | 1.316 | 1.390 | 2.179 | 0.063 | 1.039 | 1.125 | 1.204 | 1.278 | 1.347 |
| 6.8 | 2.393 | 0.068 | 1.049 | 1.148 | 1.237 | 1.317 | 1.391 | 2.171 | 0.063 | 1.039 | 1.125 | 1.204 | 1.278 | 1.347 |
| 6.9 | 2.388 | 0.068 | 1.050 | 1.149 | 1.237 | 1.317 | 1.391 | 2.162 | 0.064 | 1.038 | 1.125 | 1.204 | 1.278 | 1.348 |
| 7.0 | 2.381 | 0.068 | 1.050 | 1.149 | 1.237 | 1.318 | 1.392 | 2.154 | 0.064 | 1.038 | 1.125 | 1.204 | 1.279 | 1.348 |
| 7.1 | 2.374 | 0.068 | 1.051 | 1.150 | 1.238 | 1.318 | 1.392 | 2.145 | 0.064 | 1.037 | 1.124 | 1.204 | 1.279 | 1.348 |
| 7.2 | 2.366 | 0.068 | 1.052 | 1.150 | 1.238 | 1.318 | 1.393 | 2.137 | 0.064 | 1.037 | 1.124 | 1.204 | 1.279 | 1.349 |
| 7.3 | 2.357 | 0.068 | 1.052 | 1.150 | 1.239 | 1.319 | 1.393 | 2.128 | 0.064 | 1.037 | 1.124 | 1.204 | 1.279 | 1.349 |
| 7.4 | 2.348 | 0.068 | 1.053 | 1.151 | 1.239 | 1.319 | 1.393 | 2.120 | 0.064 | 1.037 | 1.124 | 1.204 | 1.279 | 1.349 |
| 7.5 | 2.338 | 0.068 | 1.053 | 1.151 | 1.239 | 1.319 | 1.394 | 2.111 | 0.064 | 1.036 | 1.124 | 1.204 | 1.279 | 1.350 |
| 7.6 | 2.327 | 0.068 | 1.054 | 1.151 | 1.239 | 1.320 | 1.394 | 2.103 | 0.065 | 1.036 | 1.124 | 1.204 | 1.279 | 1.350 |
| 7.7 | 2.316 | 0.068 | 1.054 | 1.152 | 1.240 | 1.320 | 1.394 | 2.094 | 0.065 | 1.036 | 1.123 | 1.204 | 1.279 | 1.350 |
| 7.8 | 2.304 | 0.068 | 1.055 | 1.152 | 1.240 | 1.320 | 1.395 | 2.085 | 0.065 | 1.036 | 1.123 | 1.204 | 1.280 | 1.350 |
| 7.9 | 2.291 | 0.068 | 1.055 | 1.152 | 1.240 | 1.320 | 1.395 | 2.077 | 0.065 | 1.035 | 1.123 | 1.204 | 1.280 | 1.351 |
| 8.0 | 2.278 | 0.067 | 1.056 | 1.153 | 1.240 | 1.320 | 1.395 | 2.068 | 0.065 | 1.035 | 1.123 | 1.204 | 1.280 | 1.351 |
| 8.1 | 2.264 | 0.067 | 1.056 | 1.153 | 1.240 | 1.321 | 1.395 | 2.060 | 0.065 | 1.035 | 1.123 | 1.204 | 1.280 | 1.352 |
| 8.2 | 2.249 | 0.067 | 1.056 | 1.153 | 1.240 | 1.321 | 1.395 | 2.051 | 0.065 | 1.035 | 1.123 | 1.205 | 1.281 | 1.352 |
| 8.3 | 2.233 | 0.067 | 1.057 | 1.153 | 1.241 | 1.321 | 1.396 | 2.042 | 0.065 | 1.035 | 1.123 | 1.205 | 1.281 | 1.352 |
| 8.4 | 2.217 | 0.067 | 1.057 | 1.153 | 1.241 | 1.321 | 1.396 | 2.034 | 0.065 | 1.036 | 1.124 | 1.205 | 1.281 | 1.353 |
| 8.5 | 2.200 | 0.067 | 1.058 | 1.154 | 1.241 | 1.321 | 1.396 | 2.025 | 0.065 | 1.036 | 1.124 | 1.205 | 1.282 | 1.354 |
| 8.6 | 2.183 | 0.067 | 1.058 | 1.154 | 1.241 | 1.321 | 1.396 | 2.017 | 0.065 | 1.036 | 1.124 | 1.206 | 1.282 | 1.354 |
| 8.7 | 2.165 | 0.067 | 1.059 | 1.154 | 1.241 | 1.321 | 1.396 | 2.008 | 0.065 | 1.037 | 1.125 | 1.206 | 1.283 | 1.355 |
| 8.8 | 2.146 | 0.067 | 1.059 | 1.154 | 1.241 | 1.321 | 1.396 | 1.999 | 0.065 | 1.037 | 1.125 | 1.207 | 1.284 | 1.356 |
| 8.9 | 2.127 | 0.067 | 1.059 | 1.154 | 1.241 | 1.321 | 1.396 | 1.991 | 0.065 | 1.038 | 1.126 | 1.208 | 1.284 | 1.357 |
| 9.0 | 2.107 | 0.067 | 1.060 | 1.154 | 1.241 | 1.321 | 1.396 | 1.982 | 0.066 | 1.038 | 1.126 | 1.208 | 1.285 | 1.358 |
| 9.1 | 2.086 | 0.067 | 1.060 | 1.154 | 1.241 | 1.321 | 1.397 | 1.974 | 0.066 | 1.039 | 1.127 | 1.209 | 1.286 | 1.359 |
| 9.2 | 2.064 | 0.067 | 1.061 | 1.155 | 1.241 | 1.321 | 1.397 | 1.965 | 0.066 | 1.040 | 1.128 | 1.210 | 1.287 | 1.360 |
| 9.3 | 2.042 | 0.067 | 1.061 | 1.155 | 1.241 | 1.321 | 1.397 | 1.957 | 0.066 | 1.041 | 1.129 | 1.211 | 1.288 | 1.361 |
| 9.4 | 2.020 | 0.067 | 1.062 | 1.155 | 1.241 | 1.321 | 1.397 | 1.948 | 0.066 | 1.042 | 1.130 | 1.212 | 1.289 | 1.362 |
| 9.5 | 1.996 | 0.067 | 1.062 | 1.155 | 1.241 | 1.321 | 1.397 | 1.940 | 0.066 | 1.043 | 1.131 | 1.213 | 1.291 | 1.364 |
| 9.6 | 1.972 | 0.067 | 1.063 | 1.155 | 1.241 | 1.321 | 1.397 | 1.931 | 0.066 | 1.044 | 1.132 | 1.215 | 1.292 | 1.365 |
| 9.7 | 1.948 | 0.067 | 1.063 | 1.155 | 1.241 | 1.321 | 1.397 | 1.923 | 0.066 | 1.045 | 1.134 | 1.216 | 1.294 | 1.367 |
| 9.8 | 1.922 | 0.067 | 1.064 | 1.155 | 1.241 | 1.321 | 1.398 | 1.914 | 0.066 | 1.047 | 1.135 | 1.217 | 1.295 | 1.369 |
| 9.9 | 1.896 | 0.067 | 1.064 | 1.156 | 1.241 | 1.321 | 1.398 | 1.906 | 0.066 | 1.048 | 1.136 | 1.219 | 1.297 | 1.371 |
| 10.0 | 1.870 | 0.067 | 1.065 | 1.156 | 1.241 | 1.322 | 1.398 | 1.898 | 0.066 | 1.050 | 1.138 | 1.221 | 1.299 | 1.373 |
| 10.1 | 1.843 | 0.067 | 1.065 | 1.156 | 1.241 | 1.322 | 1.398 | 1.889 | 0.066 | 1.051 | 1.140 | 1.223 | 1.301 | 1.375 |
| 10.2 | 1.815 | 0.067 | 1.066 | 1.156 | 1.241 | 1.322 | 1.399 | 1.881 | 0.066 | 1.053 | 1.142 | 1.224 | 1.303 | 1.377 |
| 10.3 | 1.786 | 0.067 | 1.067 | 1.157 | 1.242 | 1.322 | 1.399 | 1.873 | 0.066 | 1.055 | 1.144 | 1.226 | 1.305 | 1.379 |
| 10.4 | 1.757 | 0.066 | 1.067 | 1.157 | 1.242 | 1.322 | 1.399 | 1.865 | 0.066 | 1.057 | 1.146 | 1.229 | 1.307 | 1.381 |
| 10.5 | 1.728 | 0.066 | 1.068 | 1.157 | 1.242 | 1.323 | 1.400 | 1.857 | 0.065 | 1.059 | 1.148 | 1.231 | 1.309 | 1.384 |
| 10.6 | 1.697 | 0.066 | 1.069 | 1.158 | 1.242 | 1.323 | 1.400 | 1.849 | 0.065 | 1.062 | 1.150 | 1.233 | 1.312 | 1.387 |
| 10.7 | 1.667 | 0.066 | 1.070 | 1.158 | 1.243 | 1.323 | 1.401 | 1.841 | 0.065 | 1.064 | 1.153 | 1.236 | 1.314 | 1.389 |
| 10.8 | 1.636 | 0.066 | 1.071 | 1.159 | 1.243 | 1.324 | 1.402 | 1.833 | 0.065 | 1.067 | 1.155 | 1.238 | 1.317 | 1.392 |
| 10.9 | 1.604 | 0.066 | 1.072 | 1.160 | 1.244 | 1.324 | 1.402 | 1.825 | 0.065 | 1.069 | 1.158 | 1.241 | 1.320 | 1.395 |
| 11.0 | 1.572 | 0.066 | 1.073 | 1.160 | 1.244 | 1.325 | 1.403 | 1.817 | 0.065 | 1.072 | 1.161 | 1.244 | 1.323 | 1.399 |
| 11.1 | 1.540 | 0.066 | 1.074 | 1.161 | 1.245 | 1.326 | 1.404 | 1.810 | 0.065 | 1.075 | 1.164 | 1.247 | 1.327 | 1.402 |
| 11.2 | 1.508 | 0.066 | 1.075 | 1.162 | 1.245 | 1.326 | 1.405 | 1.802 | 0.065 | 1.079 | 1.167 | 1.251 | 1.330 | 1.406 |
| 11.3 | 1.475 | 0.066 | 1.076 | 1.163 | 1.246 | 1.327 | 1.406 | 1.794 | 0.065 | 1.082 | 1.171 | 1.254 | 1.333 | 1.409 |
| 11.4 | 1.443 | 0.066 | 1.077 | 1.164 | 1.247 | 1.328 | 1.407 | 1.787 | 0.065 | 1.085 | 1.174 | 1.258 | 1.337 | 1.413 |
| 11.5 | 1.410 | 0.066 | 1.079 | 1.165 | 1.248 | 1.329 | 1.408 | 1.779 | 0.065 | 1.089 | 1.178 | 1.261 | 1.341 | 1.417 |
| 11.6 | 1.378 | 0.066 | 1.080 | 1.166 | 1.249 | 1.330 | 1.409 | 1.772 | 0.065 | 1.093 | 1.181 | 1.265 | 1.345 | 1.421 |
| 11.7 | 1.345 | 0.066 | 1.082 | 1.167 | 1.250 | 1.331 | 1.410 | 1.764 | 0.064 | 1.097 | 1.185 | 1.269 | 1.349 | 1.425 |
| 11.8 | 1.313 | 0.066 | 1.083 | 1.168 | 1.251 | 1.332 | 1.411 | 1.757 | 0.064 | 1.101 | 1.189 | 1.273 | 1.353 | 1.430 |
| 11.9 | 1.281 | 0.065 | 1.085 | 1.169 | 1.252 | 1.333 | 1.413 | 1.750 | 0.064 | 1.105 | 1.194 | 1.278 | 1.358 | 1.434 |
| 12.0 | 1.249 | 0.065 | 1.087 | 1.171 | 1.253 | 1.334 | 1.414 | 1.742 | 0.064 | 1.109 | 1.198 | 1.282 | 1.362 | 1.439 |
| 12.1 | 1.217 | 0.065 | 1.089 | 1.172 | 1.255 | 1.336 | 1.416 | 1.735 | 0.064 | 1.114 | 1.202 | 1.286 | 1.366 | 1.443 |
| 12.2 | 1.185 | 0.065 | 1.091 | 1.174 | 1.256 | 1.337 | 1.417 | 1.728 | 0.064 | 1.118 | 1.207 | 1.291 | 1.371 | 1.448 |
| 12.3 | 1.154 | 0.065 | 1.093 | 1.176 | 1.258 | 1.339 | 1.419 | 1.721 | 0.063 | 1.123 | 1.211 | 1.295 | 1.376 | 1.453 |
| 12.4 | 1.122 | 0.065 | 1.095 | 1.177 | 1.259 | 1.340 | 1.421 | 1.714 | 0.063 | 1.127 | 1.216 | 1.300 | 1.380 | 1.457 |
| 12.5 | 1.091 | 0.065 | 1.097 | 1.179 | 1.261 | 1.342 | 1.423 | 1.707 | 0.063 | 1.132 | 1.220 | 1.304 | 1.385 | 1.462 |
| 12.6 | 1.061 | 0.064 | 1.100 | 1.182 | 1.263 | 1.344 | 1.425 | 1.700 | 0.063 | 1.136 | 1.225 | 1.309 | 1.389 | 1.467 |
| 12.7 | 1.030 | 0.064 | 1.102 | 1.184 | 1.265 | 1.346 | 1.427 | 1.694 | 0.063 | 1.141 | 1.229 | 1.313 | 1.394 | 1.471 |
| 12.8 | 1.001 | 0.064 | 1.105 | 1.186 | 1.267 | 1.348 | 1.429 | 1.687 | 0.062 | 1.145 | 1.234 | 1.318 | 1.398 | 1.476 |
| 12.9 | 0.971 | 0.064 | 1.108 | 1.189 | 1.269 | 1.351 | 1.432 | 1.680 | 0.062 | 1.150 | 1.238 | 1.322 | 1.403 | 1.480 |
| 13.0 | 0.942 | 0.064 | 1.111 | 1.191 | 1.272 | 1.353 | 1.434 | 1.674 | 0.062 | 1.154 | 1.243 | 1.327 | 1.407 | 1.485 |
| 13.1 | 0.913 | 0.063 | 1.114 | 1.194 | 1.274 | 1.355 | 1.437 | 1.667 | 0.062 | 1.159 | 1.247 | 1.331 | 1.411 | 1.489 |
| 13.2 | 0.884 | 0.063 | 1.117 | 1.197 | 1.277 | 1.358 | 1.439 | 1.661 | 0.062 | 1.163 | 1.251 | 1.335 | 1.416 | 1.493 |
| 13.3 | 0.856 | 0.063 | 1.120 | 1.200 | 1.280 | 1.361 | 1.442 | 1.654 | 0.061 | 1.168 | 1.255 | 1.339 | 1.420 | 1.497 |
| 13.4 | 0.828 | 0.063 | 1.124 | 1.203 | 1.283 | 1.363 | 1.445 | 1.648 | 0.061 | 1.172 | 1.259 | 1.343 | 1.424 | 1.501 |
| 13.5 | 0.801 | 0.062 | 1.127 | 1.206 | 1.285 | 1.366 | 1.448 | 1.642 | 0.061 | 1.176 | 1.263 | 1.347 | 1.427 | 1.505 |
| 13.6 | 0.774 | 0.062 | 1.131 | 1.209 | 1.289 | 1.369 | 1.451 | 1.636 | 0.061 | 1.180 | 1.267 | 1.351 | 1.431 | 1.509 |
| 13.7 | 0.747 | 0.062 | 1.134 | 1.212 | 1.292 | 1.372 | 1.454 | 1.629 | 0.060 | 1.184 | 1.271 | 1.354 | 1.434 | 1.512 |
| 13.8 | 0.722 | 0.062 | 1.138 | 1.216 | 1.295 | 1.375 | 1.457 | 1.623 | 0.060 | 1.187 | 1.274 | 1.358 | 1.438 | 1.515 |
| 13.9 | 0.696 | 0.061 | 1.142 | 1.219 | 1.298 | 1.378 | 1.460 | 1.617 | 0.060 | 1.191 | 1.278 | 1.361 | 1.441 | 1.518 |
| 14.0 | 0.672 | 0.061 | 1.146 | 1.223 | 1.301 | 1.382 | 1.463 | 1.611 | 0.060 | 1.194 | 1.281 | 1.364 | 1.444 | 1.521 |
| 14.1 | 0.648 | 0.061 | 1.150 | 1.226 | 1.305 | 1.385 | 1.467 | 1.605 | 0.060 | 1.198 | 1.284 | 1.367 | 1.447 | 1.524 |
| 14.2 | 0.624 | 0.060 | 1.154 | 1.230 | 1.308 | 1.388 | 1.470 | 1.600 | 0.059 | 1.201 | 1.287 | 1.370 | 1.449 | 1.527 |
| 14.3 | 0.602 | 0.060 | 1.158 | 1.234 | 1.312 | 1.391 | 1.473 | 1.594 | 0.059 | 1.204 | 1.290 | 1.372 | 1.452 | 1.529 |
| 14.4 | 0.580 | 0.060 | 1.162 | 1.238 | 1.315 | 1.395 | 1.476 | 1.588 | 0.059 | 1.207 | 1.293 | 1.375 | 1.454 | 1.531 |
| 14.5 | 0.559 | 0.059 | 1.166 | 1.241 | 1.319 | 1.398 | 1.479 | 1.582 | 0.059 | 1.210 | 1.295 | 1.377 | 1.457 | 1.534 |
| 14.6 | 0.539 | 0.059 | 1.170 | 1.245 | 1.322 | 1.401 | 1.483 | 1.577 | 0.058 | 1.213 | 1.298 | 1.380 | 1.459 | 1.536 |
| 14.7 | 0.519 | 0.059 | 1.174 | 1.249 | 1.326 | 1.405 | 1.486 | 1.571 | 0.058 | 1.215 | 1.300 | 1.382 | 1.461 | 1.538 |
| 14.8 | 0.501 | 0.058 | 1.179 | 1.253 | 1.329 | 1.408 | 1.489 | 1.566 | 0.058 | 1.218 | 1.302 | 1.384 | 1.463 | 1.540 |
| 14.9 | 0.483 | 0.058 | 1.183 | 1.257 | 1.333 | 1.411 | 1.492 | 1.560 | 0.058 | 1.220 | 1.305 | 1.386 | 1.465 | 1.541 |
| 15.0 | 0.466 | 0.058 | 1.187 | 1.260 | 1.336 | 1.415 | 1.495 | 1.555 | 0.058 | 1.222 | 1.307 | 1.388 | 1.467 | 1.543 |
| 15.1 | 0.450 | 0.057 | 1.191 | 1.264 | 1.340 | 1.418 | 1.498 | 1.549 | 0.057 | 1.225 | 1.309 | 1.390 | 1.468 | 1.545 |
| 15.2 | 0.436 | 0.057 | 1.195 | 1.268 | 1.343 | 1.421 | 1.501 | 1.544 | 0.057 | 1.227 | 1.311 | 1.392 | 1.470 | 1.546 |
| 15.3 | 0.422 | 0.057 | 1.199 | 1.272 | 1.347 | 1.424 | 1.504 | 1.539 | 0.057 | 1.229 | 1.313 | 1.393 | 1.472 | 1.548 |
| 15.4 | 0.409 | 0.056 | 1.203 | 1.275 | 1.350 | 1.428 | 1.507 | 1.533 | 0.057 | 1.231 | 1.314 | 1.395 | 1.473 | 1.549 |
| 15.5 | 0.397 | 0.056 | 1.207 | 1.279 | 1.354 | 1.431 | 1.510 | 1.528 | 0.057 | 1.233 | 1.316 | 1.396 | 1.474 | 1.550 |
| 15.6 | 0.386 | 0.056 | 1.211 | 1.283 | 1.357 | 1.434 | 1.513 | 1.523 | 0.056 | 1.235 | 1.318 | 1.398 | 1.476 | 1.552 |
| 15.7 | 0.377 | 0.055 | 1.215 | 1.286 | 1.360 | 1.437 | 1.516 | 1.518 | 0.056 | 1.237 | 1.319 | 1.399 | 1.477 | 1.553 |
| 15.8 | 0.368 | 0.055 | 1.219 | 1.290 | 1.364 | 1.440 | 1.518 | 1.513 | 0.056 | 1.239 | 1.321 | 1.401 | 1.478 | 1.554 |
| 15.9 | 0.361 | 0.055 | 1.223 | 1.293 | 1.367 | 1.443 | 1.521 | 1.508 | 0.056 | 1.240 | 1.323 | 1.402 | 1.480 | 1.555 |
| 16.0 | 0.355 | 0.054 | 1.226 | 1.297 | 1.370 | 1.445 | 1.524 | 1.503 | 0.056 | 1.242 | 1.324 | 1.404 | 1.481 | 1.556 |
| 16.1 | 0.350 | 0.054 | 1.230 | 1.300 | 1.373 | 1.448 | 1.526 | 1.498 | 0.056 | 1.244 | 1.326 | 1.405 | 1.482 | 1.557 |
| 16.2 | 0.346 | 0.054 | 1.234 | 1.303 | 1.376 | 1.451 | 1.529 | 1.493 | 0.055 | 1.245 | 1.327 | 1.406 | 1.483 | 1.558 |
| 16.3 | 0.344 | 0.053 | 1.237 | 1.307 | 1.379 | 1.453 | 1.531 | 1.488 | 0.055 | 1.247 | 1.328 | 1.407 | 1.484 | 1.559 |
| 16.4 | 0.343 | 0.053 | 1.240 | 1.310 | 1.382 | 1.456 | 1.533 | 1.483 | 0.055 | 1.249 | 1.330 | 1.409 | 1.485 | 1.560 |
| 16.5 | 0.343 | 0.053 | 1.244 | 1.313 | 1.384 | 1.459 | 1.535 | 1.478 | 0.055 | 1.250 | 1.331 | 1.410 | 1.486 | 1.561 |
| 16.6 | 0.345 | 0.052 | 1.247 | 1.316 | 1.387 | 1.461 | 1.537 | 1.474 | 0.055 | 1.252 | 1.332 | 1.411 | 1.487 | 1.562 |
| 16.7 | 0.348 | 0.052 | 1.250 | 1.318 | 1.390 | 1.463 | 1.539 | 1.469 | 0.055 | 1.253 | 1.334 | 1.412 | 1.488 | 1.563 |
| 16.8 | 0.352 | 0.052 | 1.253 | 1.321 | 1.392 | 1.465 | 1.541 | 1.464 | 0.055 | 1.254 | 1.335 | 1.413 | 1.489 | 1.564 |
| 16.9 | 0.358 | 0.052 | 1.256 | 1.324 | 1.394 | 1.468 | 1.543 | 1.459 | 0.054 | 1.256 | 1.336 | 1.414 | 1.490 | 1.564 |
| 17.0 | 0.365 | 0.051 | 1.258 | 1.326 | 1.397 | 1.470 | 1.545 | 1.455 | 0.054 | 1.257 | 1.337 | 1.415 | 1.491 | 1.565 |
| 17.1 | 0.374 | 0.051 | 1.261 | 1.329 | 1.399 | 1.471 | 1.546 | 1.450 | 0.054 | 1.258 | 1.338 | 1.416 | 1.492 | 1.566 |
| 17.2 | 0.384 | 0.051 | 1.263 | 1.331 | 1.401 | 1.473 | 1.548 | 1.446 | 0.054 | 1.260 | 1.339 | 1.417 | 1.493 | 1.567 |
| 17.3 | 0.395 | 0.051 | 1.266 | 1.333 | 1.403 | 1.475 | 1.549 | 1.441 | 0.054 | 1.261 | 1.341 | 1.418 | 1.494 | 1.567 |
| 17.4 | 0.408 | 0.050 | 1.268 | 1.335 | 1.405 | 1.477 | 1.551 | 1.436 | 0.054 | 1.262 | 1.342 | 1.419 | 1.494 | 1.568 |
| 17.5 | 0.422 | 0.050 | 1.270 | 1.337 | 1.407 | 1.478 | 1.552 | 1.432 | 0.054 | 1.264 | 1.343 | 1.420 | 1.495 | 1.569 |
| 17.6 | 0.438 | 0.050 | 1.272 | 1.339 | 1.409 | 1.480 | 1.553 | 1.427 | 0.054 | 1.265 | 1.344 | 1.421 | 1.496 | 1.570 |
| 17.7 | 0.455 | 0.050 | 1.274 | 1.341 | 1.410 | 1.481 | 1.554 | 1.423 | 0.053 | 1.266 | 1.345 | 1.422 | 1.497 | 1.570 |
| 17.8 | 0.473 | 0.050 | 1.275 | 1.343 | 1.412 | 1.483 | 1.555 | 1.419 | 0.053 | 1.267 | 1.346 | 1.422 | 1.497 | 1.571 |
| 17.9 | 0.493 | 0.049 | 1.277 | 1.344 | 1.413 | 1.484 | 1.556 | 1.414 | 0.053 | 1.268 | 1.347 | 1.423 | 1.498 | 1.571 |
| 18.0 | 0.513 | 0.049 | 1.279 | 1.346 | 1.415 | 1.485 | 1.557 | 1.410 | 0.053 | 1.270 | 1.348 | 1.424 | 1.499 | 1.572 |
| 18.1 | 0.535 | 0.049 | 1.280 | 1.347 | 1.416 | 1.486 | 1.558 | 1.405 | 0.053 | 1.271 | 1.349 | 1.425 | 1.499 | 1.572 |
| 18.2 | 0.558 | 0.049 | 1.281 | 1.348 | 1.417 | 1.487 | 1.559 | 1.401 | 0.053 | 1.272 | 1.349 | 1.425 | 1.500 | 1.573 |
| 18.3 | 0.583 | 0.049 | 1.283 | 1.350 | 1.418 | 1.488 | 1.560 | 1.396 | 0.053 | 1.273 | 1.350 | 1.426 | 1.500 | 1.573 |
| 18.4 | 0.608 | 0.049 | 1.284 | 1.351 | 1.419 | 1.489 | 1.560 | 1.392 | 0.052 | 1.274 | 1.351 | 1.427 | 1.501 | 1.573 |
| 18.5 | 0.634 | 0.049 | 1.285 | 1.352 | 1.421 | 1.490 | 1.561 | 1.388 | 0.052 | 1.275 | 1.352 | 1.427 | 1.501 | 1.573 |
| 18.6 | 0.661 | 0.049 | 1.286 | 1.353 | 1.422 | 1.491 | 1.562 | 1.383 | 0.052 | 1.275 | 1.352 | 1.427 | 1.501 | 1.573 |
| 18.7 | 0.689 | 0.048 | 1.287 | 1.354 | 1.423 | 1.492 | 1.562 | 1.379 | 0.052 | 1.276 | 1.353 | 1.428 | 1.501 | 1.574 |
| 18.8 | 0.718 | 0.048 | 1.288 | 1.355 | 1.423 | 1.493 | 1.563 | 1.375 | 0.052 | 1.277 | 1.353 | 1.428 | 1.502 | 1.574 |
| 18.9 | 0.748 | 0.048 | 1.289 | 1.356 | 1.424 | 1.494 | 1.564 | 1.370 | 0.052 | 1.278 | 1.354 | 1.429 | 1.502 | 1.574 |
| 19.0 | 0.778 | 0.048 | 1.289 | 1.357 | 1.425 | 1.494 | 1.564 | 1.366 | 0.052 | 1.278 | 1.354 | 1.429 | 1.502 | 1.573 |
| 19.1 | 0.809 | 0.048 | 1.290 | 1.358 | 1.426 | 1.495 | 1.565 | 1.362 | 0.051 | 1.279 | 1.355 | 1.429 | 1.502 | 1.573 |
| 19.2 | 0.841 | 0.048 | 1.291 | 1.359 | 1.427 | 1.496 | 1.565 | 1.357 | 0.051 | 1.280 | 1.355 | 1.429 | 1.502 | 1.573 |
| 19.3 | 0.873 | 0.048 | 1.291 | 1.359 | 1.428 | 1.497 | 1.566 | 1.353 | 0.051 | 1.280 | 1.355 | 1.429 | 1.501 | 1.573 |
| 19.4 | 0.906 | 0.048 | 1.292 | 1.360 | 1.429 | 1.497 | 1.567 | 1.349 | 0.051 | 1.281 | 1.356 | 1.429 | 1.501 | 1.572 |
| 19.5 | 0.940 | 0.048 | 1.293 | 1.361 | 1.429 | 1.498 | 1.567 | 1.344 | 0.051 | 1.281 | 1.356 | 1.429 | 1.501 | 1.572 |
| 19.6 | 0.974 | 0.048 | 1.293 | 1.362 | 1.430 | 1.499 | 1.568 | 1.340 | 0.051 | 1.282 | 1.356 | 1.429 | 1.501 | 1.571 |
| 19.7 | 1.008 | 0.048 | 1.294 | 1.362 | 1.431 | 1.500 | 1.568 | 1.336 | 0.050 | 1.282 | 1.356 | 1.429 | 1.501 | 1.571 |
| 19.8 | 1.043 | 0.048 | 1.294 | 1.363 | 1.432 | 1.500 | 1.569 | 1.331 | 0.050 | 1.282 | 1.356 | 1.429 | 1.500 | 1.570 |
| 19.9 | 1.078 | 0.048 | 1.295 | 1.364 | 1.432 | 1.501 | 1.569 | 1.327 | 0.050 | 1.283 | 1.356 | 1.429 | 1.500 | 1.570 |
| 20.0 | 1.114 | 0.048 | 1.295 | 1.364 | 1.433 | 1.501 | 1.569 | 1.323 | 0.050 | 1.283 | 1.357 | 1.429 | 1.500 | 1.569 |

| L3 to L4 TBS | | | | | | | | | | | | | | |
| --- | --- | --- | --- | --- | --- | --- | --- | --- | --- | --- | --- | --- | --- | --- |
|  | Males | | | | | | | Females | | | | | | |
| Age | L | S | -2 SD | -1 SD | 0 SD (M) | 1 SD | 2 SD | L | S | -2 SD | -1 SD | 0 SD (M) | 1 SD | 2 SD |
| 5.0 | 2.716 | 0.064 | 1.122 | 1.224 | 1.313 | 1.393 | 1.466 | 1.731 | 0.064 | 1.112 | 1.200 | 1.284 | 1.363 | 1.440 |
| 5.1 | 2.707 | 0.064 | 1.123 | 1.225 | 1.314 | 1.393 | 1.466 | 1.734 | 0.064 | 1.111 | 1.200 | 1.283 | 1.363 | 1.439 |
| 5.2 | 2.698 | 0.064 | 1.125 | 1.225 | 1.314 | 1.393 | 1.466 | 1.737 | 0.064 | 1.111 | 1.199 | 1.283 | 1.362 | 1.439 |
| 5.3 | 2.689 | 0.063 | 1.126 | 1.226 | 1.314 | 1.393 | 1.465 | 1.739 | 0.064 | 1.110 | 1.199 | 1.282 | 1.362 | 1.439 |
| 5.4 | 2.680 | 0.063 | 1.127 | 1.227 | 1.314 | 1.393 | 1.465 | 1.742 | 0.064 | 1.110 | 1.198 | 1.282 | 1.362 | 1.438 |
| 5.5 | 2.671 | 0.063 | 1.128 | 1.228 | 1.315 | 1.393 | 1.465 | 1.745 | 0.064 | 1.109 | 1.198 | 1.281 | 1.361 | 1.438 |
| 5.6 | 2.662 | 0.062 | 1.130 | 1.228 | 1.315 | 1.393 | 1.465 | 1.748 | 0.064 | 1.109 | 1.197 | 1.281 | 1.361 | 1.437 |
| 5.7 | 2.653 | 0.062 | 1.131 | 1.229 | 1.315 | 1.393 | 1.465 | 1.750 | 0.064 | 1.108 | 1.197 | 1.280 | 1.360 | 1.437 |
| 5.8 | 2.644 | 0.062 | 1.132 | 1.229 | 1.316 | 1.393 | 1.465 | 1.753 | 0.064 | 1.108 | 1.196 | 1.280 | 1.360 | 1.436 |
| 5.9 | 2.635 | 0.062 | 1.133 | 1.230 | 1.316 | 1.393 | 1.464 | 1.756 | 0.064 | 1.107 | 1.196 | 1.280 | 1.360 | 1.436 |
| 6.0 | 2.626 | 0.062 | 1.134 | 1.231 | 1.316 | 1.393 | 1.464 | 1.759 | 0.064 | 1.107 | 1.195 | 1.279 | 1.359 | 1.436 |
| 6.1 | 2.617 | 0.061 | 1.135 | 1.231 | 1.316 | 1.393 | 1.464 | 1.761 | 0.064 | 1.106 | 1.195 | 1.279 | 1.359 | 1.435 |
| 6.2 | 2.608 | 0.061 | 1.136 | 1.232 | 1.316 | 1.393 | 1.464 | 1.764 | 0.064 | 1.106 | 1.195 | 1.279 | 1.358 | 1.435 |
| 6.3 | 2.599 | 0.061 | 1.137 | 1.232 | 1.317 | 1.393 | 1.464 | 1.767 | 0.064 | 1.106 | 1.194 | 1.278 | 1.358 | 1.435 |
| 6.4 | 2.590 | 0.061 | 1.138 | 1.233 | 1.317 | 1.393 | 1.463 | 1.770 | 0.064 | 1.105 | 1.194 | 1.278 | 1.358 | 1.434 |
| 6.5 | 2.581 | 0.061 | 1.139 | 1.233 | 1.317 | 1.393 | 1.463 | 1.772 | 0.064 | 1.105 | 1.194 | 1.278 | 1.358 | 1.434 |
| 6.6 | 2.572 | 0.060 | 1.140 | 1.234 | 1.317 | 1.393 | 1.463 | 1.775 | 0.064 | 1.105 | 1.194 | 1.277 | 1.357 | 1.434 |
| 6.7 | 2.563 | 0.060 | 1.141 | 1.234 | 1.317 | 1.393 | 1.463 | 1.778 | 0.064 | 1.105 | 1.193 | 1.277 | 1.357 | 1.433 |
| 6.8 | 2.554 | 0.060 | 1.142 | 1.234 | 1.317 | 1.393 | 1.463 | 1.781 | 0.064 | 1.104 | 1.193 | 1.277 | 1.357 | 1.433 |
| 6.9 | 2.545 | 0.060 | 1.142 | 1.235 | 1.317 | 1.393 | 1.462 | 1.783 | 0.064 | 1.104 | 1.193 | 1.277 | 1.356 | 1.433 |
| 7.0 | 2.536 | 0.060 | 1.143 | 1.235 | 1.318 | 1.393 | 1.462 | 1.786 | 0.064 | 1.104 | 1.193 | 1.277 | 1.356 | 1.432 |
| 7.1 | 2.527 | 0.059 | 1.144 | 1.235 | 1.318 | 1.393 | 1.462 | 1.789 | 0.064 | 1.104 | 1.193 | 1.276 | 1.356 | 1.432 |
| 7.2 | 2.518 | 0.059 | 1.145 | 1.236 | 1.318 | 1.393 | 1.462 | 1.791 | 0.064 | 1.104 | 1.193 | 1.276 | 1.356 | 1.432 |
| 7.3 | 2.509 | 0.059 | 1.145 | 1.236 | 1.318 | 1.392 | 1.462 | 1.794 | 0.064 | 1.104 | 1.193 | 1.276 | 1.356 | 1.432 |
| 7.4 | 2.500 | 0.059 | 1.146 | 1.236 | 1.318 | 1.392 | 1.461 | 1.797 | 0.064 | 1.104 | 1.193 | 1.276 | 1.356 | 1.431 |
| 7.5 | 2.491 | 0.059 | 1.146 | 1.237 | 1.318 | 1.392 | 1.461 | 1.800 | 0.064 | 1.104 | 1.193 | 1.276 | 1.355 | 1.431 |
| 7.6 | 2.482 | 0.059 | 1.147 | 1.237 | 1.318 | 1.392 | 1.461 | 1.802 | 0.064 | 1.105 | 1.193 | 1.276 | 1.355 | 1.431 |
| 7.7 | 2.473 | 0.059 | 1.148 | 1.237 | 1.318 | 1.392 | 1.461 | 1.805 | 0.064 | 1.105 | 1.193 | 1.276 | 1.355 | 1.431 |
| 7.8 | 2.464 | 0.058 | 1.148 | 1.237 | 1.318 | 1.392 | 1.461 | 1.808 | 0.063 | 1.105 | 1.193 | 1.276 | 1.355 | 1.431 |
| 7.9 | 2.455 | 0.058 | 1.149 | 1.238 | 1.318 | 1.392 | 1.460 | 1.811 | 0.063 | 1.105 | 1.193 | 1.276 | 1.355 | 1.430 |
| 8.0 | 2.446 | 0.058 | 1.149 | 1.238 | 1.318 | 1.392 | 1.460 | 1.813 | 0.063 | 1.105 | 1.193 | 1.276 | 1.355 | 1.430 |
| 8.1 | 2.437 | 0.058 | 1.150 | 1.238 | 1.318 | 1.392 | 1.460 | 1.816 | 0.063 | 1.106 | 1.194 | 1.276 | 1.355 | 1.430 |
| 8.2 | 2.428 | 0.058 | 1.150 | 1.238 | 1.318 | 1.392 | 1.460 | 1.819 | 0.063 | 1.106 | 1.194 | 1.276 | 1.355 | 1.430 |
| 8.3 | 2.419 | 0.058 | 1.151 | 1.238 | 1.318 | 1.391 | 1.460 | 1.822 | 0.063 | 1.107 | 1.194 | 1.277 | 1.355 | 1.430 |
| 8.4 | 2.410 | 0.058 | 1.151 | 1.239 | 1.318 | 1.391 | 1.459 | 1.824 | 0.063 | 1.107 | 1.194 | 1.277 | 1.355 | 1.429 |
| 8.5 | 2.401 | 0.058 | 1.152 | 1.239 | 1.318 | 1.391 | 1.459 | 1.827 | 0.063 | 1.107 | 1.195 | 1.277 | 1.355 | 1.429 |
| 8.6 | 2.392 | 0.058 | 1.152 | 1.239 | 1.318 | 1.391 | 1.459 | 1.830 | 0.063 | 1.108 | 1.195 | 1.277 | 1.355 | 1.429 |
| 8.7 | 2.383 | 0.057 | 1.153 | 1.239 | 1.318 | 1.391 | 1.459 | 1.833 | 0.062 | 1.108 | 1.195 | 1.277 | 1.355 | 1.429 |
| 8.8 | 2.374 | 0.057 | 1.153 | 1.239 | 1.318 | 1.391 | 1.459 | 1.835 | 0.062 | 1.109 | 1.196 | 1.277 | 1.355 | 1.429 |
| 8.9 | 2.365 | 0.057 | 1.153 | 1.239 | 1.318 | 1.391 | 1.459 | 1.838 | 0.062 | 1.110 | 1.196 | 1.277 | 1.355 | 1.429 |
| 9.0 | 2.356 | 0.057 | 1.153 | 1.239 | 1.318 | 1.391 | 1.458 | 1.841 | 0.062 | 1.110 | 1.196 | 1.278 | 1.355 | 1.428 |
| 9.1 | 2.347 | 0.057 | 1.154 | 1.239 | 1.318 | 1.391 | 1.458 | 1.844 | 0.062 | 1.111 | 1.197 | 1.278 | 1.355 | 1.428 |
| 9.2 | 2.338 | 0.057 | 1.154 | 1.239 | 1.318 | 1.390 | 1.458 | 1.846 | 0.062 | 1.112 | 1.197 | 1.278 | 1.355 | 1.428 |
| 9.3 | 2.329 | 0.057 | 1.154 | 1.239 | 1.318 | 1.390 | 1.458 | 1.849 | 0.061 | 1.112 | 1.198 | 1.279 | 1.355 | 1.428 |
| 9.4 | 2.320 | 0.057 | 1.154 | 1.239 | 1.318 | 1.390 | 1.458 | 1.852 | 0.061 | 1.113 | 1.199 | 1.279 | 1.355 | 1.428 |
| 9.5 | 2.311 | 0.057 | 1.154 | 1.239 | 1.318 | 1.390 | 1.458 | 1.855 | 0.061 | 1.114 | 1.199 | 1.280 | 1.356 | 1.428 |
| 9.6 | 2.302 | 0.057 | 1.154 | 1.239 | 1.317 | 1.390 | 1.458 | 1.857 | 0.061 | 1.115 | 1.200 | 1.280 | 1.356 | 1.429 |
| 9.7 | 2.293 | 0.057 | 1.154 | 1.239 | 1.317 | 1.390 | 1.458 | 1.860 | 0.061 | 1.116 | 1.201 | 1.281 | 1.356 | 1.429 |
| 9.8 | 2.284 | 0.057 | 1.154 | 1.239 | 1.317 | 1.390 | 1.457 | 1.863 | 0.060 | 1.117 | 1.202 | 1.281 | 1.357 | 1.429 |
| 9.9 | 2.275 | 0.057 | 1.154 | 1.239 | 1.317 | 1.389 | 1.457 | 1.866 | 0.060 | 1.119 | 1.203 | 1.282 | 1.358 | 1.430 |
| 10.0 | 2.266 | 0.057 | 1.154 | 1.239 | 1.317 | 1.389 | 1.457 | 1.868 | 0.060 | 1.120 | 1.204 | 1.283 | 1.358 | 1.430 |
| 10.1 | 2.257 | 0.057 | 1.154 | 1.239 | 1.317 | 1.389 | 1.457 | 1.871 | 0.060 | 1.121 | 1.205 | 1.284 | 1.359 | 1.431 |
| 10.2 | 2.248 | 0.057 | 1.154 | 1.239 | 1.317 | 1.389 | 1.457 | 1.874 | 0.060 | 1.123 | 1.207 | 1.285 | 1.360 | 1.431 |
| 10.3 | 2.239 | 0.057 | 1.154 | 1.239 | 1.316 | 1.389 | 1.457 | 1.877 | 0.059 | 1.125 | 1.208 | 1.287 | 1.361 | 1.432 |
| 10.4 | 2.230 | 0.057 | 1.154 | 1.238 | 1.316 | 1.389 | 1.457 | 1.879 | 0.059 | 1.127 | 1.210 | 1.288 | 1.362 | 1.433 |
| 10.5 | 2.221 | 0.057 | 1.154 | 1.238 | 1.316 | 1.389 | 1.457 | 1.882 | 0.059 | 1.129 | 1.211 | 1.290 | 1.364 | 1.434 |
| 10.6 | 2.212 | 0.057 | 1.154 | 1.238 | 1.316 | 1.389 | 1.457 | 1.885 | 0.059 | 1.131 | 1.213 | 1.291 | 1.365 | 1.436 |
| 10.7 | 2.203 | 0.057 | 1.154 | 1.238 | 1.316 | 1.389 | 1.457 | 1.888 | 0.058 | 1.133 | 1.215 | 1.293 | 1.367 | 1.437 |
| 10.8 | 2.194 | 0.057 | 1.154 | 1.238 | 1.316 | 1.389 | 1.457 | 1.890 | 0.058 | 1.136 | 1.218 | 1.295 | 1.369 | 1.439 |
| 10.9 | 2.185 | 0.057 | 1.154 | 1.238 | 1.316 | 1.389 | 1.457 | 1.893 | 0.058 | 1.138 | 1.220 | 1.297 | 1.371 | 1.441 |
| 11.0 | 2.176 | 0.057 | 1.154 | 1.238 | 1.316 | 1.389 | 1.457 | 1.896 | 0.058 | 1.141 | 1.223 | 1.300 | 1.373 | 1.443 |
| 11.1 | 2.167 | 0.057 | 1.154 | 1.238 | 1.316 | 1.389 | 1.457 | 1.899 | 0.057 | 1.144 | 1.225 | 1.302 | 1.375 | 1.445 |
| 11.2 | 2.158 | 0.057 | 1.154 | 1.238 | 1.316 | 1.389 | 1.457 | 1.901 | 0.057 | 1.147 | 1.228 | 1.305 | 1.378 | 1.447 |
| 11.3 | 2.149 | 0.057 | 1.155 | 1.238 | 1.316 | 1.389 | 1.458 | 1.904 | 0.057 | 1.151 | 1.232 | 1.308 | 1.381 | 1.450 |
| 11.4 | 2.140 | 0.057 | 1.155 | 1.239 | 1.316 | 1.389 | 1.458 | 1.907 | 0.057 | 1.154 | 1.235 | 1.311 | 1.383 | 1.453 |
| 11.5 | 2.131 | 0.057 | 1.155 | 1.239 | 1.317 | 1.389 | 1.458 | 1.910 | 0.056 | 1.158 | 1.238 | 1.314 | 1.386 | 1.455 |
| 11.6 | 2.122 | 0.057 | 1.156 | 1.239 | 1.317 | 1.390 | 1.459 | 1.912 | 0.056 | 1.161 | 1.242 | 1.318 | 1.390 | 1.458 |
| 11.7 | 2.113 | 0.057 | 1.157 | 1.240 | 1.317 | 1.390 | 1.459 | 1.915 | 0.056 | 1.165 | 1.245 | 1.321 | 1.393 | 1.462 |
| 11.8 | 2.104 | 0.057 | 1.157 | 1.241 | 1.318 | 1.391 | 1.460 | 1.918 | 0.055 | 1.169 | 1.249 | 1.325 | 1.396 | 1.465 |
| 11.9 | 2.095 | 0.057 | 1.158 | 1.241 | 1.319 | 1.391 | 1.460 | 1.921 | 0.055 | 1.173 | 1.253 | 1.328 | 1.400 | 1.468 |
| 12.0 | 2.086 | 0.057 | 1.159 | 1.242 | 1.320 | 1.392 | 1.461 | 1.923 | 0.055 | 1.178 | 1.257 | 1.332 | 1.403 | 1.471 |
| 12.1 | 2.077 | 0.057 | 1.161 | 1.243 | 1.320 | 1.393 | 1.462 | 1.926 | 0.055 | 1.182 | 1.261 | 1.336 | 1.407 | 1.475 |
| 12.2 | 2.068 | 0.056 | 1.162 | 1.245 | 1.322 | 1.394 | 1.463 | 1.929 | 0.054 | 1.186 | 1.265 | 1.340 | 1.411 | 1.478 |
| 12.3 | 2.059 | 0.056 | 1.164 | 1.246 | 1.323 | 1.395 | 1.464 | 1.932 | 0.054 | 1.191 | 1.269 | 1.344 | 1.414 | 1.482 |
| 12.4 | 2.050 | 0.056 | 1.165 | 1.247 | 1.324 | 1.396 | 1.465 | 1.934 | 0.054 | 1.195 | 1.273 | 1.348 | 1.418 | 1.486 |
| 12.5 | 2.041 | 0.056 | 1.167 | 1.249 | 1.326 | 1.398 | 1.466 | 1.937 | 0.053 | 1.199 | 1.278 | 1.352 | 1.422 | 1.489 |
| 12.6 | 2.032 | 0.056 | 1.170 | 1.251 | 1.327 | 1.400 | 1.468 | 1.940 | 0.053 | 1.204 | 1.282 | 1.355 | 1.426 | 1.493 |
| 12.7 | 2.023 | 0.056 | 1.172 | 1.253 | 1.329 | 1.401 | 1.470 | 1.943 | 0.053 | 1.208 | 1.286 | 1.359 | 1.429 | 1.496 |
| 12.8 | 2.014 | 0.055 | 1.175 | 1.256 | 1.331 | 1.403 | 1.472 | 1.945 | 0.052 | 1.213 | 1.290 | 1.363 | 1.433 | 1.500 |
| 12.9 | 2.005 | 0.055 | 1.177 | 1.258 | 1.334 | 1.405 | 1.474 | 1.948 | 0.052 | 1.217 | 1.294 | 1.367 | 1.437 | 1.503 |
| 13.0 | 1.996 | 0.055 | 1.180 | 1.261 | 1.336 | 1.408 | 1.476 | 1.951 | 0.052 | 1.221 | 1.298 | 1.371 | 1.440 | 1.506 |
| 13.1 | 1.987 | 0.055 | 1.183 | 1.263 | 1.339 | 1.410 | 1.478 | 1.954 | 0.051 | 1.226 | 1.302 | 1.375 | 1.444 | 1.510 |
| 13.2 | 1.978 | 0.054 | 1.187 | 1.266 | 1.341 | 1.413 | 1.481 | 1.956 | 0.051 | 1.230 | 1.306 | 1.378 | 1.447 | 1.513 |
| 13.3 | 1.969 | 0.054 | 1.190 | 1.270 | 1.344 | 1.415 | 1.483 | 1.959 | 0.051 | 1.234 | 1.310 | 1.382 | 1.451 | 1.516 |
| 13.4 | 1.960 | 0.054 | 1.194 | 1.273 | 1.348 | 1.418 | 1.486 | 1.962 | 0.050 | 1.238 | 1.314 | 1.386 | 1.454 | 1.519 |
| 13.5 | 1.951 | 0.054 | 1.198 | 1.276 | 1.351 | 1.421 | 1.489 | 1.965 | 0.050 | 1.242 | 1.317 | 1.389 | 1.457 | 1.522 |
| 13.6 | 1.942 | 0.053 | 1.202 | 1.280 | 1.354 | 1.425 | 1.492 | 1.967 | 0.050 | 1.246 | 1.321 | 1.392 | 1.460 | 1.525 |
| 13.7 | 1.933 | 0.053 | 1.206 | 1.284 | 1.358 | 1.428 | 1.495 | 1.970 | 0.050 | 1.250 | 1.325 | 1.395 | 1.463 | 1.528 |
| 13.8 | 1.924 | 0.053 | 1.210 | 1.288 | 1.361 | 1.431 | 1.498 | 1.973 | 0.049 | 1.253 | 1.328 | 1.399 | 1.466 | 1.530 |
| 13.9 | 1.915 | 0.052 | 1.214 | 1.292 | 1.365 | 1.435 | 1.502 | 1.976 | 0.049 | 1.257 | 1.331 | 1.401 | 1.469 | 1.533 |
| 14.0 | 1.906 | 0.052 | 1.219 | 1.296 | 1.369 | 1.438 | 1.505 | 1.978 | 0.049 | 1.260 | 1.334 | 1.404 | 1.471 | 1.535 |
| 14.1 | 1.897 | 0.052 | 1.223 | 1.300 | 1.373 | 1.442 | 1.508 | 1.981 | 0.048 | 1.264 | 1.337 | 1.407 | 1.474 | 1.537 |
| 14.2 | 1.888 | 0.051 | 1.228 | 1.304 | 1.376 | 1.446 | 1.512 | 1.984 | 0.048 | 1.267 | 1.340 | 1.410 | 1.476 | 1.540 |
| 14.3 | 1.879 | 0.051 | 1.233 | 1.308 | 1.380 | 1.449 | 1.515 | 1.987 | 0.048 | 1.270 | 1.343 | 1.412 | 1.478 | 1.542 |
| 14.4 | 1.870 | 0.051 | 1.237 | 1.313 | 1.384 | 1.453 | 1.519 | 1.989 | 0.048 | 1.273 | 1.346 | 1.415 | 1.481 | 1.544 |
| 14.5 | 1.861 | 0.050 | 1.242 | 1.317 | 1.388 | 1.457 | 1.522 | 1.992 | 0.047 | 1.276 | 1.348 | 1.417 | 1.483 | 1.546 |
| 14.6 | 1.852 | 0.050 | 1.247 | 1.321 | 1.392 | 1.460 | 1.526 | 1.995 | 0.047 | 1.278 | 1.351 | 1.419 | 1.485 | 1.548 |
| 14.7 | 1.843 | 0.050 | 1.251 | 1.325 | 1.396 | 1.464 | 1.529 | 1.997 | 0.047 | 1.281 | 1.353 | 1.422 | 1.487 | 1.549 |
| 14.8 | 1.834 | 0.049 | 1.256 | 1.329 | 1.400 | 1.468 | 1.533 | 2.000 | 0.047 | 1.284 | 1.355 | 1.424 | 1.489 | 1.551 |
| 14.9 | 1.825 | 0.049 | 1.260 | 1.334 | 1.404 | 1.471 | 1.536 | 2.003 | 0.047 | 1.286 | 1.358 | 1.426 | 1.490 | 1.553 |
| 15.0 | 1.816 | 0.049 | 1.265 | 1.338 | 1.408 | 1.475 | 1.539 | 2.006 | 0.046 | 1.289 | 1.360 | 1.428 | 1.492 | 1.554 |
| 15.1 | 1.807 | 0.048 | 1.269 | 1.342 | 1.411 | 1.478 | 1.543 | 2.008 | 0.046 | 1.291 | 1.362 | 1.429 | 1.494 | 1.555 |
| 15.2 | 1.798 | 0.048 | 1.274 | 1.346 | 1.415 | 1.482 | 1.546 | 2.011 | 0.046 | 1.293 | 1.364 | 1.431 | 1.495 | 1.557 |
| 15.3 | 1.789 | 0.048 | 1.278 | 1.350 | 1.419 | 1.485 | 1.549 | 2.014 | 0.046 | 1.295 | 1.366 | 1.433 | 1.497 | 1.558 |
| 15.4 | 1.780 | 0.047 | 1.282 | 1.353 | 1.422 | 1.488 | 1.552 | 2.017 | 0.046 | 1.297 | 1.367 | 1.434 | 1.498 | 1.559 |
| 15.5 | 1.771 | 0.047 | 1.286 | 1.357 | 1.425 | 1.491 | 1.555 | 2.019 | 0.045 | 1.299 | 1.369 | 1.436 | 1.499 | 1.560 |
| 15.6 | 1.762 | 0.047 | 1.290 | 1.361 | 1.429 | 1.494 | 1.558 | 2.022 | 0.045 | 1.301 | 1.371 | 1.437 | 1.500 | 1.561 |
| 15.7 | 1.753 | 0.046 | 1.294 | 1.364 | 1.432 | 1.497 | 1.561 | 2.025 | 0.045 | 1.302 | 1.372 | 1.438 | 1.502 | 1.562 |
| 15.8 | 1.744 | 0.046 | 1.297 | 1.368 | 1.435 | 1.500 | 1.564 | 2.028 | 0.045 | 1.304 | 1.373 | 1.439 | 1.503 | 1.563 |
| 15.9 | 1.735 | 0.046 | 1.301 | 1.371 | 1.438 | 1.503 | 1.566 | 2.030 | 0.045 | 1.305 | 1.375 | 1.441 | 1.503 | 1.564 |
| 16.0 | 1.726 | 0.046 | 1.304 | 1.374 | 1.441 | 1.506 | 1.569 | 2.033 | 0.044 | 1.307 | 1.376 | 1.442 | 1.504 | 1.564 |
| 16.1 | 1.717 | 0.045 | 1.308 | 1.377 | 1.444 | 1.508 | 1.571 | 2.036 | 0.044 | 1.308 | 1.377 | 1.443 | 1.505 | 1.565 |
| 16.2 | 1.708 | 0.045 | 1.311 | 1.380 | 1.446 | 1.511 | 1.573 | 2.039 | 0.044 | 1.310 | 1.378 | 1.443 | 1.506 | 1.566 |
| 16.3 | 1.699 | 0.045 | 1.314 | 1.382 | 1.449 | 1.513 | 1.576 | 2.041 | 0.044 | 1.311 | 1.379 | 1.444 | 1.507 | 1.566 |
| 16.4 | 1.690 | 0.045 | 1.317 | 1.385 | 1.451 | 1.515 | 1.578 | 2.044 | 0.044 | 1.312 | 1.380 | 1.445 | 1.507 | 1.567 |
| 16.5 | 1.681 | 0.045 | 1.319 | 1.387 | 1.453 | 1.517 | 1.580 | 2.047 | 0.044 | 1.313 | 1.381 | 1.446 | 1.508 | 1.567 |
| 16.6 | 1.672 | 0.045 | 1.322 | 1.390 | 1.456 | 1.519 | 1.582 | 2.050 | 0.044 | 1.314 | 1.382 | 1.447 | 1.508 | 1.568 |
| 16.7 | 1.663 | 0.044 | 1.324 | 1.392 | 1.458 | 1.521 | 1.583 | 2.052 | 0.043 | 1.315 | 1.383 | 1.448 | 1.509 | 1.568 |
| 16.8 | 1.654 | 0.044 | 1.326 | 1.394 | 1.460 | 1.523 | 1.585 | 2.055 | 0.043 | 1.317 | 1.384 | 1.448 | 1.510 | 1.568 |
| 16.9 | 1.645 | 0.044 | 1.329 | 1.396 | 1.461 | 1.525 | 1.587 | 2.058 | 0.043 | 1.318 | 1.385 | 1.449 | 1.510 | 1.569 |
| 17.0 | 1.636 | 0.044 | 1.331 | 1.398 | 1.463 | 1.527 | 1.588 | 2.061 | 0.043 | 1.319 | 1.386 | 1.450 | 1.511 | 1.569 |
| 17.1 | 1.627 | 0.044 | 1.333 | 1.400 | 1.465 | 1.528 | 1.590 | 2.063 | 0.043 | 1.320 | 1.387 | 1.451 | 1.511 | 1.570 |
| 17.2 | 1.618 | 0.044 | 1.334 | 1.401 | 1.466 | 1.530 | 1.591 | 2.066 | 0.043 | 1.321 | 1.388 | 1.451 | 1.512 | 1.570 |
| 17.3 | 1.609 | 0.044 | 1.336 | 1.403 | 1.468 | 1.531 | 1.593 | 2.069 | 0.043 | 1.322 | 1.389 | 1.452 | 1.513 | 1.571 |
| 17.4 | 1.600 | 0.044 | 1.338 | 1.404 | 1.469 | 1.533 | 1.594 | 2.072 | 0.042 | 1.323 | 1.390 | 1.453 | 1.513 | 1.571 |
| 17.5 | 1.591 | 0.044 | 1.339 | 1.406 | 1.471 | 1.534 | 1.596 | 2.074 | 0.042 | 1.324 | 1.391 | 1.454 | 1.514 | 1.572 |
| 17.6 | 1.582 | 0.043 | 1.341 | 1.407 | 1.472 | 1.535 | 1.597 | 2.077 | 0.042 | 1.325 | 1.392 | 1.454 | 1.514 | 1.572 |
| 17.7 | 1.573 | 0.043 | 1.342 | 1.408 | 1.473 | 1.536 | 1.598 | 2.080 | 0.042 | 1.327 | 1.392 | 1.455 | 1.515 | 1.572 |
| 17.8 | 1.564 | 0.043 | 1.343 | 1.410 | 1.474 | 1.538 | 1.599 | 2.083 | 0.042 | 1.328 | 1.393 | 1.456 | 1.516 | 1.573 |
| 17.9 | 1.555 | 0.043 | 1.344 | 1.411 | 1.475 | 1.539 | 1.601 | 2.085 | 0.042 | 1.329 | 1.394 | 1.457 | 1.516 | 1.573 |
| 18.0 | 1.546 | 0.043 | 1.345 | 1.412 | 1.476 | 1.540 | 1.602 | 2.088 | 0.042 | 1.330 | 1.395 | 1.457 | 1.517 | 1.574 |
| 18.1 | 1.537 | 0.043 | 1.346 | 1.413 | 1.478 | 1.541 | 1.603 | 2.091 | 0.042 | 1.331 | 1.396 | 1.458 | 1.517 | 1.574 |
| 18.2 | 1.528 | 0.043 | 1.347 | 1.414 | 1.478 | 1.542 | 1.604 | 2.094 | 0.041 | 1.332 | 1.397 | 1.459 | 1.518 | 1.575 |
| 18.3 | 1.519 | 0.043 | 1.348 | 1.414 | 1.479 | 1.543 | 1.605 | 2.096 | 0.041 | 1.333 | 1.398 | 1.459 | 1.518 | 1.575 |
| 18.4 | 1.510 | 0.043 | 1.349 | 1.415 | 1.480 | 1.544 | 1.606 | 2.099 | 0.041 | 1.334 | 1.398 | 1.460 | 1.519 | 1.575 |
| 18.5 | 1.501 | 0.043 | 1.349 | 1.416 | 1.481 | 1.545 | 1.607 | 2.102 | 0.041 | 1.335 | 1.399 | 1.461 | 1.519 | 1.575 |
| 18.6 | 1.492 | 0.044 | 1.350 | 1.417 | 1.482 | 1.546 | 1.608 | 2.105 | 0.041 | 1.336 | 1.400 | 1.461 | 1.520 | 1.576 |
| 18.7 | 1.483 | 0.044 | 1.351 | 1.417 | 1.483 | 1.547 | 1.610 | 2.107 | 0.041 | 1.336 | 1.401 | 1.462 | 1.520 | 1.576 |
| 18.8 | 1.474 | 0.044 | 1.351 | 1.418 | 1.484 | 1.548 | 1.611 | 2.110 | 0.041 | 1.337 | 1.401 | 1.462 | 1.520 | 1.576 |
| 18.9 | 1.465 | 0.044 | 1.352 | 1.419 | 1.484 | 1.549 | 1.612 | 2.113 | 0.041 | 1.338 | 1.402 | 1.462 | 1.520 | 1.576 |
| 19.0 | 1.456 | 0.044 | 1.352 | 1.419 | 1.485 | 1.549 | 1.613 | 2.116 | 0.040 | 1.339 | 1.402 | 1.463 | 1.520 | 1.576 |
| 19.1 | 1.447 | 0.044 | 1.353 | 1.420 | 1.486 | 1.550 | 1.614 | 2.118 | 0.040 | 1.339 | 1.403 | 1.463 | 1.521 | 1.576 |
| 19.2 | 1.438 | 0.044 | 1.353 | 1.420 | 1.486 | 1.551 | 1.615 | 2.121 | 0.040 | 1.340 | 1.403 | 1.463 | 1.521 | 1.576 |
| 19.3 | 1.429 | 0.044 | 1.353 | 1.421 | 1.487 | 1.552 | 1.616 | 2.124 | 0.040 | 1.340 | 1.403 | 1.463 | 1.521 | 1.576 |
| 19.4 | 1.420 | 0.044 | 1.354 | 1.421 | 1.488 | 1.553 | 1.617 | 2.127 | 0.040 | 1.341 | 1.404 | 1.463 | 1.521 | 1.575 |
| 19.5 | 1.411 | 0.044 | 1.354 | 1.422 | 1.488 | 1.553 | 1.617 | 2.129 | 0.040 | 1.341 | 1.404 | 1.463 | 1.520 | 1.575 |
| 19.6 | 1.402 | 0.044 | 1.354 | 1.422 | 1.489 | 1.554 | 1.618 | 2.132 | 0.040 | 1.342 | 1.404 | 1.464 | 1.520 | 1.575 |
| 19.7 | 1.393 | 0.044 | 1.355 | 1.422 | 1.489 | 1.555 | 1.619 | 2.135 | 0.040 | 1.342 | 1.404 | 1.464 | 1.520 | 1.574 |
| 19.8 | 1.384 | 0.044 | 1.355 | 1.423 | 1.490 | 1.555 | 1.620 | 2.138 | 0.039 | 1.343 | 1.405 | 1.463 | 1.520 | 1.574 |
| 19.9 | 1.375 | 0.045 | 1.355 | 1.423 | 1.490 | 1.556 | 1.621 | 2.140 | 0.039 | 1.343 | 1.405 | 1.463 | 1.520 | 1.574 |
| 20.0 | 1.366 | 0.045 | 1.355 | 1.423 | 1.490 | 1.556 | 1.621 | 2.143 | 0.039 | 1.343 | 1.405 | 1.463 | 1.519 | 1.573 |

| L2 to L3 TBS | | | | | | | | | | | | | | |
| --- | --- | --- | --- | --- | --- | --- | --- | --- | --- | --- | --- | --- | --- | --- |
|  | Males | | | | | | | Females | | | | | | |
| Age | L | S | -2 SD | -1 SD | 0 SD (M) | 1 SD | 2 SD | L | S | -2 SD | -1 SD | 0 SD (M) | 1 SD | 2 SD |
| 5.0 | 4.506 | 0.060 | 1.076 | 1.192 | 1.278 | 1.348 | 1.407 | 1.931 | 0.054 | 1.111 | 1.185 | 1.255 | 1.321 | 1.384 |
| 5.1 | 4.429 | 0.060 | 1.078 | 1.193 | 1.279 | 1.349 | 1.408 | 1.928 | 0.054 | 1.111 | 1.185 | 1.255 | 1.321 | 1.385 |
| 5.2 | 4.351 | 0.060 | 1.081 | 1.194 | 1.280 | 1.350 | 1.409 | 1.925 | 0.055 | 1.110 | 1.185 | 1.255 | 1.322 | 1.386 |
| 5.3 | 4.274 | 0.060 | 1.083 | 1.195 | 1.281 | 1.351 | 1.411 | 1.922 | 0.055 | 1.109 | 1.184 | 1.255 | 1.322 | 1.386 |
| 5.4 | 4.197 | 0.060 | 1.086 | 1.196 | 1.282 | 1.352 | 1.412 | 1.919 | 0.055 | 1.109 | 1.184 | 1.255 | 1.322 | 1.387 |
| 5.5 | 4.121 | 0.060 | 1.088 | 1.197 | 1.282 | 1.353 | 1.413 | 1.916 | 0.055 | 1.108 | 1.184 | 1.255 | 1.323 | 1.387 |
| 5.6 | 4.044 | 0.060 | 1.090 | 1.198 | 1.283 | 1.354 | 1.414 | 1.913 | 0.056 | 1.108 | 1.183 | 1.255 | 1.323 | 1.388 |
| 5.7 | 3.968 | 0.060 | 1.093 | 1.200 | 1.284 | 1.354 | 1.415 | 1.911 | 0.056 | 1.107 | 1.183 | 1.255 | 1.323 | 1.389 |
| 5.8 | 3.893 | 0.060 | 1.095 | 1.201 | 1.285 | 1.355 | 1.417 | 1.908 | 0.056 | 1.106 | 1.183 | 1.255 | 1.324 | 1.389 |
| 5.9 | 3.818 | 0.059 | 1.097 | 1.202 | 1.285 | 1.356 | 1.418 | 1.905 | 0.056 | 1.106 | 1.182 | 1.255 | 1.324 | 1.390 |
| 6.0 | 3.744 | 0.059 | 1.099 | 1.203 | 1.286 | 1.357 | 1.419 | 1.902 | 0.057 | 1.105 | 1.182 | 1.255 | 1.324 | 1.390 |
| 6.1 | 3.670 | 0.059 | 1.101 | 1.204 | 1.287 | 1.358 | 1.420 | 1.899 | 0.057 | 1.104 | 1.182 | 1.255 | 1.325 | 1.391 |
| 6.2 | 3.596 | 0.059 | 1.103 | 1.204 | 1.288 | 1.359 | 1.421 | 1.895 | 0.057 | 1.104 | 1.182 | 1.255 | 1.325 | 1.392 |
| 6.3 | 3.524 | 0.059 | 1.105 | 1.205 | 1.288 | 1.360 | 1.423 | 1.892 | 0.057 | 1.103 | 1.181 | 1.255 | 1.325 | 1.392 |
| 6.4 | 3.452 | 0.059 | 1.107 | 1.206 | 1.289 | 1.360 | 1.424 | 1.888 | 0.057 | 1.103 | 1.181 | 1.255 | 1.325 | 1.393 |
| 6.5 | 3.381 | 0.059 | 1.108 | 1.207 | 1.290 | 1.361 | 1.425 | 1.884 | 0.058 | 1.102 | 1.181 | 1.255 | 1.326 | 1.393 |
| 6.6 | 3.310 | 0.059 | 1.110 | 1.208 | 1.290 | 1.362 | 1.426 | 1.880 | 0.058 | 1.102 | 1.181 | 1.255 | 1.326 | 1.394 |
| 6.7 | 3.241 | 0.059 | 1.112 | 1.209 | 1.291 | 1.363 | 1.427 | 1.875 | 0.058 | 1.101 | 1.180 | 1.255 | 1.326 | 1.394 |
| 6.8 | 3.172 | 0.059 | 1.113 | 1.210 | 1.292 | 1.364 | 1.428 | 1.870 | 0.058 | 1.101 | 1.180 | 1.255 | 1.327 | 1.395 |
| 6.9 | 3.105 | 0.059 | 1.115 | 1.210 | 1.292 | 1.364 | 1.429 | 1.864 | 0.058 | 1.100 | 1.180 | 1.255 | 1.327 | 1.395 |
| 7.0 | 3.038 | 0.059 | 1.116 | 1.211 | 1.293 | 1.365 | 1.430 | 1.859 | 0.059 | 1.100 | 1.180 | 1.256 | 1.327 | 1.396 |
| 7.1 | 2.972 | 0.059 | 1.118 | 1.212 | 1.293 | 1.366 | 1.431 | 1.852 | 0.059 | 1.100 | 1.180 | 1.256 | 1.328 | 1.396 |
| 7.2 | 2.908 | 0.059 | 1.119 | 1.212 | 1.294 | 1.366 | 1.432 | 1.845 | 0.059 | 1.100 | 1.180 | 1.256 | 1.328 | 1.397 |
| 7.3 | 2.844 | 0.059 | 1.120 | 1.213 | 1.294 | 1.367 | 1.433 | 1.838 | 0.059 | 1.100 | 1.180 | 1.256 | 1.328 | 1.398 |
| 7.4 | 2.782 | 0.059 | 1.122 | 1.214 | 1.295 | 1.367 | 1.434 | 1.830 | 0.059 | 1.100 | 1.180 | 1.256 | 1.329 | 1.398 |
| 7.5 | 2.721 | 0.059 | 1.123 | 1.214 | 1.295 | 1.368 | 1.435 | 1.822 | 0.059 | 1.100 | 1.180 | 1.257 | 1.329 | 1.399 |
| 7.6 | 2.661 | 0.059 | 1.124 | 1.215 | 1.295 | 1.368 | 1.436 | 1.813 | 0.059 | 1.100 | 1.180 | 1.257 | 1.330 | 1.399 |
| 7.7 | 2.602 | 0.059 | 1.125 | 1.215 | 1.296 | 1.369 | 1.436 | 1.803 | 0.059 | 1.100 | 1.181 | 1.257 | 1.330 | 1.400 |
| 7.8 | 2.545 | 0.059 | 1.126 | 1.216 | 1.296 | 1.369 | 1.437 | 1.793 | 0.059 | 1.100 | 1.181 | 1.257 | 1.331 | 1.401 |
| 7.9 | 2.489 | 0.059 | 1.127 | 1.216 | 1.296 | 1.370 | 1.438 | 1.783 | 0.060 | 1.100 | 1.181 | 1.258 | 1.331 | 1.401 |
| 8.0 | 2.434 | 0.059 | 1.128 | 1.216 | 1.297 | 1.370 | 1.439 | 1.772 | 0.060 | 1.101 | 1.181 | 1.258 | 1.332 | 1.402 |
| 8.1 | 2.380 | 0.059 | 1.129 | 1.217 | 1.297 | 1.371 | 1.439 | 1.761 | 0.060 | 1.101 | 1.182 | 1.259 | 1.332 | 1.403 |
| 8.2 | 2.328 | 0.059 | 1.130 | 1.217 | 1.297 | 1.371 | 1.440 | 1.749 | 0.060 | 1.101 | 1.182 | 1.259 | 1.333 | 1.403 |
| 8.3 | 2.277 | 0.059 | 1.130 | 1.217 | 1.297 | 1.371 | 1.440 | 1.737 | 0.060 | 1.102 | 1.183 | 1.260 | 1.333 | 1.404 |
| 8.4 | 2.227 | 0.059 | 1.131 | 1.218 | 1.297 | 1.372 | 1.441 | 1.725 | 0.060 | 1.102 | 1.183 | 1.260 | 1.334 | 1.405 |
| 8.5 | 2.179 | 0.059 | 1.132 | 1.218 | 1.298 | 1.372 | 1.442 | 1.713 | 0.060 | 1.103 | 1.183 | 1.260 | 1.334 | 1.405 |
| 8.6 | 2.132 | 0.059 | 1.133 | 1.218 | 1.298 | 1.372 | 1.442 | 1.700 | 0.060 | 1.103 | 1.184 | 1.261 | 1.335 | 1.406 |
| 8.7 | 2.086 | 0.059 | 1.133 | 1.219 | 1.298 | 1.372 | 1.443 | 1.688 | 0.060 | 1.104 | 1.185 | 1.262 | 1.336 | 1.407 |
| 8.8 | 2.041 | 0.059 | 1.134 | 1.219 | 1.298 | 1.373 | 1.443 | 1.675 | 0.060 | 1.104 | 1.185 | 1.262 | 1.336 | 1.408 |
| 8.9 | 1.998 | 0.059 | 1.135 | 1.219 | 1.298 | 1.373 | 1.444 | 1.662 | 0.060 | 1.105 | 1.186 | 1.263 | 1.337 | 1.409 |
| 9.0 | 1.955 | 0.059 | 1.135 | 1.219 | 1.299 | 1.373 | 1.444 | 1.650 | 0.060 | 1.106 | 1.187 | 1.264 | 1.338 | 1.410 |
| 9.1 | 1.914 | 0.059 | 1.136 | 1.220 | 1.299 | 1.373 | 1.445 | 1.637 | 0.060 | 1.107 | 1.187 | 1.264 | 1.339 | 1.411 |
| 9.2 | 1.874 | 0.059 | 1.136 | 1.220 | 1.299 | 1.374 | 1.445 | 1.625 | 0.060 | 1.108 | 1.188 | 1.265 | 1.340 | 1.412 |
| 9.3 | 1.834 | 0.059 | 1.137 | 1.220 | 1.299 | 1.374 | 1.446 | 1.613 | 0.060 | 1.109 | 1.189 | 1.266 | 1.341 | 1.413 |
| 9.4 | 1.796 | 0.059 | 1.137 | 1.220 | 1.299 | 1.374 | 1.446 | 1.602 | 0.060 | 1.110 | 1.190 | 1.267 | 1.342 | 1.414 |
| 9.5 | 1.758 | 0.059 | 1.138 | 1.220 | 1.299 | 1.374 | 1.446 | 1.591 | 0.060 | 1.111 | 1.191 | 1.269 | 1.343 | 1.415 |
| 9.6 | 1.721 | 0.059 | 1.138 | 1.220 | 1.299 | 1.374 | 1.447 | 1.581 | 0.060 | 1.112 | 1.192 | 1.270 | 1.344 | 1.417 |
| 9.7 | 1.685 | 0.059 | 1.139 | 1.221 | 1.299 | 1.374 | 1.447 | 1.571 | 0.060 | 1.113 | 1.194 | 1.271 | 1.346 | 1.418 |
| 9.8 | 1.649 | 0.059 | 1.139 | 1.221 | 1.299 | 1.375 | 1.447 | 1.562 | 0.060 | 1.115 | 1.195 | 1.272 | 1.347 | 1.420 |
| 9.9 | 1.614 | 0.059 | 1.139 | 1.221 | 1.299 | 1.375 | 1.448 | 1.553 | 0.060 | 1.116 | 1.197 | 1.274 | 1.349 | 1.422 |
| 10.0 | 1.579 | 0.059 | 1.140 | 1.221 | 1.299 | 1.375 | 1.448 | 1.546 | 0.060 | 1.118 | 1.198 | 1.276 | 1.351 | 1.423 |
| 10.1 | 1.545 | 0.059 | 1.140 | 1.221 | 1.299 | 1.375 | 1.448 | 1.539 | 0.060 | 1.120 | 1.200 | 1.277 | 1.352 | 1.425 |
| 10.2 | 1.511 | 0.059 | 1.140 | 1.221 | 1.299 | 1.375 | 1.449 | 1.533 | 0.060 | 1.121 | 1.202 | 1.279 | 1.354 | 1.427 |
| 10.3 | 1.477 | 0.059 | 1.141 | 1.221 | 1.299 | 1.375 | 1.449 | 1.529 | 0.060 | 1.123 | 1.204 | 1.281 | 1.356 | 1.429 |
| 10.4 | 1.443 | 0.059 | 1.141 | 1.222 | 1.300 | 1.376 | 1.450 | 1.525 | 0.060 | 1.125 | 1.206 | 1.283 | 1.358 | 1.432 |
| 10.5 | 1.410 | 0.059 | 1.142 | 1.222 | 1.300 | 1.376 | 1.450 | 1.523 | 0.059 | 1.127 | 1.208 | 1.285 | 1.361 | 1.434 |
| 10.6 | 1.377 | 0.059 | 1.142 | 1.222 | 1.300 | 1.376 | 1.451 | 1.521 | 0.059 | 1.130 | 1.210 | 1.288 | 1.363 | 1.436 |
| 10.7 | 1.343 | 0.059 | 1.143 | 1.222 | 1.300 | 1.377 | 1.451 | 1.521 | 0.059 | 1.132 | 1.212 | 1.290 | 1.366 | 1.439 |
| 10.8 | 1.310 | 0.059 | 1.143 | 1.223 | 1.301 | 1.377 | 1.452 | 1.521 | 0.059 | 1.134 | 1.215 | 1.293 | 1.368 | 1.442 |
| 10.9 | 1.276 | 0.059 | 1.144 | 1.223 | 1.301 | 1.377 | 1.453 | 1.523 | 0.059 | 1.137 | 1.218 | 1.296 | 1.371 | 1.444 |
| 11.0 | 1.242 | 0.059 | 1.145 | 1.224 | 1.301 | 1.378 | 1.454 | 1.526 | 0.059 | 1.140 | 1.221 | 1.298 | 1.374 | 1.447 |
| 11.1 | 1.209 | 0.059 | 1.145 | 1.224 | 1.302 | 1.379 | 1.454 | 1.530 | 0.059 | 1.143 | 1.224 | 1.301 | 1.377 | 1.450 |
| 11.2 | 1.174 | 0.059 | 1.146 | 1.225 | 1.302 | 1.379 | 1.455 | 1.535 | 0.059 | 1.146 | 1.227 | 1.305 | 1.380 | 1.453 |
| 11.3 | 1.140 | 0.059 | 1.147 | 1.225 | 1.303 | 1.380 | 1.456 | 1.541 | 0.059 | 1.149 | 1.230 | 1.308 | 1.383 | 1.457 |
| 11.4 | 1.105 | 0.059 | 1.148 | 1.226 | 1.304 | 1.381 | 1.457 | 1.549 | 0.058 | 1.153 | 1.234 | 1.311 | 1.387 | 1.460 |
| 11.5 | 1.070 | 0.059 | 1.149 | 1.227 | 1.305 | 1.382 | 1.459 | 1.557 | 0.058 | 1.157 | 1.237 | 1.315 | 1.390 | 1.464 |
| 11.6 | 1.035 | 0.059 | 1.151 | 1.228 | 1.305 | 1.383 | 1.460 | 1.566 | 0.058 | 1.160 | 1.241 | 1.319 | 1.394 | 1.467 |
| 11.7 | 1.000 | 0.059 | 1.152 | 1.229 | 1.307 | 1.384 | 1.461 | 1.577 | 0.058 | 1.164 | 1.245 | 1.323 | 1.398 | 1.471 |
| 11.8 | 0.964 | 0.059 | 1.153 | 1.230 | 1.308 | 1.385 | 1.462 | 1.588 | 0.058 | 1.168 | 1.249 | 1.327 | 1.402 | 1.475 |
| 11.9 | 0.928 | 0.059 | 1.155 | 1.232 | 1.309 | 1.386 | 1.464 | 1.600 | 0.057 | 1.172 | 1.253 | 1.331 | 1.406 | 1.479 |
| 12.0 | 0.891 | 0.059 | 1.157 | 1.233 | 1.310 | 1.388 | 1.466 | 1.613 | 0.057 | 1.177 | 1.257 | 1.335 | 1.410 | 1.482 |
| 12.1 | 0.855 | 0.059 | 1.159 | 1.235 | 1.312 | 1.389 | 1.467 | 1.627 | 0.057 | 1.181 | 1.262 | 1.339 | 1.414 | 1.486 |
| 12.2 | 0.818 | 0.059 | 1.161 | 1.236 | 1.313 | 1.391 | 1.469 | 1.642 | 0.057 | 1.185 | 1.266 | 1.343 | 1.418 | 1.490 |
| 12.3 | 0.782 | 0.059 | 1.163 | 1.238 | 1.315 | 1.392 | 1.471 | 1.658 | 0.056 | 1.190 | 1.271 | 1.348 | 1.422 | 1.494 |
| 12.4 | 0.745 | 0.058 | 1.165 | 1.240 | 1.317 | 1.394 | 1.473 | 1.675 | 0.056 | 1.195 | 1.275 | 1.352 | 1.426 | 1.498 |
| 12.5 | 0.708 | 0.058 | 1.168 | 1.242 | 1.319 | 1.396 | 1.475 | 1.692 | 0.056 | 1.199 | 1.280 | 1.357 | 1.431 | 1.502 |
| 12.6 | 0.671 | 0.058 | 1.170 | 1.245 | 1.321 | 1.398 | 1.477 | 1.710 | 0.055 | 1.204 | 1.284 | 1.361 | 1.435 | 1.506 |
| 12.7 | 0.634 | 0.058 | 1.173 | 1.247 | 1.323 | 1.400 | 1.480 | 1.729 | 0.055 | 1.209 | 1.289 | 1.365 | 1.439 | 1.510 |
| 12.8 | 0.598 | 0.058 | 1.176 | 1.250 | 1.325 | 1.403 | 1.482 | 1.749 | 0.055 | 1.214 | 1.293 | 1.370 | 1.443 | 1.514 |
| 12.9 | 0.562 | 0.058 | 1.179 | 1.252 | 1.328 | 1.405 | 1.484 | 1.769 | 0.054 | 1.218 | 1.298 | 1.374 | 1.447 | 1.517 |
| 13.0 | 0.527 | 0.057 | 1.182 | 1.255 | 1.330 | 1.408 | 1.487 | 1.790 | 0.054 | 1.223 | 1.302 | 1.378 | 1.451 | 1.521 |
| 13.1 | 0.493 | 0.057 | 1.186 | 1.258 | 1.333 | 1.410 | 1.490 | 1.812 | 0.053 | 1.227 | 1.307 | 1.382 | 1.455 | 1.524 |
| 13.2 | 0.459 | 0.057 | 1.189 | 1.261 | 1.336 | 1.413 | 1.492 | 1.835 | 0.053 | 1.232 | 1.311 | 1.386 | 1.458 | 1.528 |
| 13.3 | 0.426 | 0.056 | 1.193 | 1.265 | 1.339 | 1.416 | 1.495 | 1.858 | 0.053 | 1.236 | 1.315 | 1.390 | 1.462 | 1.531 |
| 13.4 | 0.394 | 0.056 | 1.196 | 1.268 | 1.342 | 1.419 | 1.498 | 1.882 | 0.052 | 1.241 | 1.319 | 1.394 | 1.466 | 1.534 |
| 13.5 | 0.363 | 0.056 | 1.200 | 1.272 | 1.345 | 1.422 | 1.501 | 1.906 | 0.052 | 1.245 | 1.324 | 1.398 | 1.469 | 1.537 |
| 13.6 | 0.333 | 0.056 | 1.204 | 1.275 | 1.349 | 1.425 | 1.504 | 1.932 | 0.052 | 1.249 | 1.327 | 1.402 | 1.472 | 1.540 |
| 13.7 | 0.305 | 0.055 | 1.208 | 1.279 | 1.352 | 1.428 | 1.507 | 1.958 | 0.051 | 1.253 | 1.331 | 1.405 | 1.475 | 1.543 |
| 13.8 | 0.278 | 0.055 | 1.213 | 1.283 | 1.356 | 1.431 | 1.510 | 1.984 | 0.051 | 1.257 | 1.335 | 1.408 | 1.478 | 1.545 |
| 13.9 | 0.252 | 0.055 | 1.217 | 1.287 | 1.359 | 1.435 | 1.514 | 2.012 | 0.051 | 1.261 | 1.338 | 1.412 | 1.481 | 1.548 |
| 14.0 | 0.228 | 0.054 | 1.221 | 1.290 | 1.363 | 1.438 | 1.517 | 2.040 | 0.050 | 1.264 | 1.342 | 1.415 | 1.484 | 1.550 |
| 14.1 | 0.206 | 0.054 | 1.226 | 1.294 | 1.366 | 1.442 | 1.520 | 2.068 | 0.050 | 1.268 | 1.345 | 1.418 | 1.487 | 1.552 |
| 14.2 | 0.185 | 0.053 | 1.230 | 1.298 | 1.370 | 1.445 | 1.523 | 2.097 | 0.050 | 1.271 | 1.348 | 1.421 | 1.489 | 1.554 |
| 14.3 | 0.166 | 0.053 | 1.234 | 1.303 | 1.374 | 1.448 | 1.526 | 2.127 | 0.049 | 1.274 | 1.351 | 1.423 | 1.492 | 1.556 |
| 14.4 | 0.149 | 0.053 | 1.239 | 1.307 | 1.378 | 1.452 | 1.529 | 2.157 | 0.049 | 1.277 | 1.354 | 1.426 | 1.494 | 1.558 |
| 14.5 | 0.134 | 0.052 | 1.243 | 1.311 | 1.381 | 1.455 | 1.533 | 2.187 | 0.049 | 1.280 | 1.357 | 1.428 | 1.496 | 1.560 |
| 14.6 | 0.121 | 0.052 | 1.248 | 1.315 | 1.385 | 1.459 | 1.536 | 2.218 | 0.048 | 1.283 | 1.359 | 1.431 | 1.498 | 1.562 |
| 14.7 | 0.109 | 0.052 | 1.252 | 1.319 | 1.389 | 1.462 | 1.539 | 2.249 | 0.048 | 1.286 | 1.362 | 1.433 | 1.500 | 1.564 |
| 14.8 | 0.100 | 0.051 | 1.257 | 1.323 | 1.393 | 1.466 | 1.542 | 2.281 | 0.048 | 1.288 | 1.365 | 1.436 | 1.502 | 1.566 |
| 14.9 | 0.093 | 0.051 | 1.261 | 1.327 | 1.397 | 1.469 | 1.545 | 2.313 | 0.048 | 1.291 | 1.367 | 1.438 | 1.504 | 1.567 |
| 15.0 | 0.088 | 0.050 | 1.265 | 1.331 | 1.400 | 1.473 | 1.548 | 2.345 | 0.047 | 1.293 | 1.369 | 1.440 | 1.506 | 1.569 |
| 15.1 | 0.086 | 0.050 | 1.270 | 1.335 | 1.404 | 1.476 | 1.551 | 2.377 | 0.047 | 1.296 | 1.372 | 1.442 | 1.508 | 1.570 |
| 15.2 | 0.086 | 0.050 | 1.274 | 1.339 | 1.408 | 1.479 | 1.554 | 2.410 | 0.047 | 1.298 | 1.374 | 1.444 | 1.510 | 1.572 |
| 15.3 | 0.088 | 0.049 | 1.278 | 1.343 | 1.411 | 1.482 | 1.557 | 2.443 | 0.047 | 1.300 | 1.376 | 1.446 | 1.511 | 1.573 |
| 15.4 | 0.092 | 0.049 | 1.282 | 1.347 | 1.415 | 1.486 | 1.560 | 2.476 | 0.047 | 1.302 | 1.378 | 1.448 | 1.513 | 1.575 |
| 15.5 | 0.099 | 0.049 | 1.286 | 1.351 | 1.418 | 1.489 | 1.562 | 2.509 | 0.046 | 1.304 | 1.380 | 1.450 | 1.515 | 1.576 |
| 15.6 | 0.108 | 0.048 | 1.290 | 1.354 | 1.422 | 1.492 | 1.565 | 2.542 | 0.046 | 1.306 | 1.381 | 1.451 | 1.516 | 1.577 |
| 15.7 | 0.120 | 0.048 | 1.294 | 1.358 | 1.425 | 1.495 | 1.568 | 2.575 | 0.046 | 1.308 | 1.383 | 1.453 | 1.518 | 1.578 |
| 15.8 | 0.134 | 0.048 | 1.298 | 1.362 | 1.428 | 1.498 | 1.570 | 2.608 | 0.046 | 1.309 | 1.385 | 1.455 | 1.519 | 1.580 |
| 15.9 | 0.151 | 0.047 | 1.301 | 1.365 | 1.431 | 1.500 | 1.572 | 2.641 | 0.046 | 1.311 | 1.387 | 1.456 | 1.520 | 1.581 |
| 16.0 | 0.170 | 0.047 | 1.305 | 1.368 | 1.435 | 1.503 | 1.575 | 2.675 | 0.046 | 1.313 | 1.388 | 1.458 | 1.522 | 1.582 |
| 16.1 | 0.192 | 0.047 | 1.308 | 1.372 | 1.438 | 1.506 | 1.577 | 2.708 | 0.046 | 1.314 | 1.390 | 1.459 | 1.523 | 1.583 |
| 16.2 | 0.216 | 0.046 | 1.312 | 1.375 | 1.440 | 1.509 | 1.579 | 2.741 | 0.045 | 1.316 | 1.391 | 1.460 | 1.524 | 1.584 |
| 16.3 | 0.243 | 0.046 | 1.315 | 1.378 | 1.443 | 1.511 | 1.581 | 2.773 | 0.045 | 1.317 | 1.393 | 1.462 | 1.526 | 1.585 |
| 16.4 | 0.272 | 0.046 | 1.318 | 1.381 | 1.446 | 1.513 | 1.583 | 2.806 | 0.045 | 1.318 | 1.394 | 1.463 | 1.527 | 1.586 |
| 16.5 | 0.303 | 0.046 | 1.321 | 1.384 | 1.449 | 1.516 | 1.585 | 2.838 | 0.045 | 1.320 | 1.396 | 1.464 | 1.528 | 1.587 |
| 16.6 | 0.337 | 0.045 | 1.324 | 1.386 | 1.451 | 1.518 | 1.587 | 2.870 | 0.045 | 1.321 | 1.397 | 1.466 | 1.529 | 1.588 |
| 16.7 | 0.373 | 0.045 | 1.326 | 1.389 | 1.454 | 1.520 | 1.589 | 2.902 | 0.045 | 1.322 | 1.398 | 1.467 | 1.530 | 1.589 |
| 16.8 | 0.411 | 0.045 | 1.329 | 1.392 | 1.456 | 1.522 | 1.590 | 2.933 | 0.045 | 1.323 | 1.399 | 1.468 | 1.531 | 1.590 |
| 16.9 | 0.452 | 0.045 | 1.331 | 1.394 | 1.459 | 1.525 | 1.592 | 2.964 | 0.045 | 1.325 | 1.401 | 1.469 | 1.532 | 1.590 |
| 17.0 | 0.495 | 0.044 | 1.334 | 1.397 | 1.461 | 1.527 | 1.594 | 2.994 | 0.045 | 1.326 | 1.402 | 1.471 | 1.533 | 1.591 |
| 17.1 | 0.540 | 0.044 | 1.336 | 1.399 | 1.463 | 1.528 | 1.595 | 3.024 | 0.044 | 1.327 | 1.403 | 1.472 | 1.534 | 1.592 |
| 17.2 | 0.587 | 0.044 | 1.338 | 1.401 | 1.465 | 1.530 | 1.597 | 3.054 | 0.044 | 1.328 | 1.404 | 1.473 | 1.535 | 1.593 |
| 17.3 | 0.635 | 0.044 | 1.340 | 1.403 | 1.467 | 1.532 | 1.598 | 3.082 | 0.044 | 1.330 | 1.406 | 1.474 | 1.536 | 1.594 |
| 17.4 | 0.686 | 0.044 | 1.342 | 1.405 | 1.469 | 1.534 | 1.599 | 3.111 | 0.044 | 1.331 | 1.407 | 1.475 | 1.537 | 1.594 |
| 17.5 | 0.739 | 0.044 | 1.344 | 1.407 | 1.471 | 1.535 | 1.601 | 3.138 | 0.044 | 1.332 | 1.408 | 1.476 | 1.538 | 1.595 |
| 17.6 | 0.793 | 0.044 | 1.346 | 1.409 | 1.473 | 1.537 | 1.602 | 3.165 | 0.044 | 1.333 | 1.409 | 1.477 | 1.539 | 1.596 |
| 17.7 | 0.849 | 0.043 | 1.347 | 1.411 | 1.474 | 1.539 | 1.603 | 3.191 | 0.044 | 1.334 | 1.410 | 1.478 | 1.540 | 1.597 |
| 17.8 | 0.906 | 0.043 | 1.349 | 1.412 | 1.476 | 1.540 | 1.604 | 3.216 | 0.044 | 1.336 | 1.411 | 1.479 | 1.541 | 1.597 |
| 17.9 | 0.965 | 0.043 | 1.350 | 1.414 | 1.478 | 1.541 | 1.605 | 3.241 | 0.043 | 1.337 | 1.413 | 1.480 | 1.542 | 1.598 |
| 18.0 | 1.025 | 0.043 | 1.351 | 1.415 | 1.479 | 1.543 | 1.607 | 3.265 | 0.043 | 1.338 | 1.414 | 1.481 | 1.542 | 1.598 |
| 18.1 | 1.087 | 0.043 | 1.352 | 1.417 | 1.480 | 1.544 | 1.608 | 3.288 | 0.043 | 1.339 | 1.415 | 1.482 | 1.543 | 1.599 |
| 18.2 | 1.149 | 0.043 | 1.354 | 1.418 | 1.482 | 1.545 | 1.608 | 3.310 | 0.043 | 1.340 | 1.416 | 1.483 | 1.544 | 1.599 |
| 18.3 | 1.213 | 0.043 | 1.355 | 1.419 | 1.483 | 1.547 | 1.609 | 3.332 | 0.043 | 1.341 | 1.417 | 1.484 | 1.544 | 1.600 |
| 18.4 | 1.278 | 0.043 | 1.355 | 1.420 | 1.484 | 1.548 | 1.610 | 3.352 | 0.043 | 1.342 | 1.417 | 1.484 | 1.545 | 1.600 |
| 18.5 | 1.343 | 0.043 | 1.356 | 1.421 | 1.486 | 1.549 | 1.611 | 3.372 | 0.043 | 1.343 | 1.418 | 1.485 | 1.545 | 1.600 |
| 18.6 | 1.409 | 0.043 | 1.357 | 1.422 | 1.487 | 1.550 | 1.612 | 3.391 | 0.042 | 1.344 | 1.419 | 1.485 | 1.545 | 1.600 |
| 18.7 | 1.476 | 0.043 | 1.358 | 1.423 | 1.488 | 1.551 | 1.613 | 3.410 | 0.042 | 1.345 | 1.420 | 1.486 | 1.546 | 1.600 |
| 18.8 | 1.544 | 0.043 | 1.358 | 1.424 | 1.489 | 1.552 | 1.613 | 3.427 | 0.042 | 1.346 | 1.420 | 1.486 | 1.546 | 1.600 |
| 18.9 | 1.612 | 0.043 | 1.359 | 1.425 | 1.490 | 1.553 | 1.614 | 3.444 | 0.042 | 1.347 | 1.421 | 1.487 | 1.546 | 1.600 |
| 19.0 | 1.681 | 0.043 | 1.359 | 1.426 | 1.491 | 1.554 | 1.615 | 3.460 | 0.042 | 1.348 | 1.422 | 1.487 | 1.546 | 1.600 |
| 19.1 | 1.750 | 0.043 | 1.360 | 1.427 | 1.492 | 1.555 | 1.616 | 3.476 | 0.042 | 1.349 | 1.422 | 1.487 | 1.546 | 1.600 |
| 19.2 | 1.820 | 0.043 | 1.360 | 1.428 | 1.493 | 1.555 | 1.616 | 3.491 | 0.041 | 1.349 | 1.423 | 1.487 | 1.546 | 1.599 |
| 19.3 | 1.890 | 0.043 | 1.360 | 1.428 | 1.494 | 1.556 | 1.617 | 3.505 | 0.041 | 1.350 | 1.423 | 1.488 | 1.546 | 1.599 |
| 19.4 | 1.960 | 0.043 | 1.361 | 1.429 | 1.494 | 1.557 | 1.617 | 3.518 | 0.041 | 1.351 | 1.423 | 1.488 | 1.546 | 1.599 |
| 19.5 | 2.031 | 0.043 | 1.361 | 1.430 | 1.495 | 1.558 | 1.618 | 3.532 | 0.041 | 1.351 | 1.423 | 1.488 | 1.545 | 1.598 |
| 19.6 | 2.102 | 0.043 | 1.361 | 1.430 | 1.496 | 1.558 | 1.619 | 3.544 | 0.041 | 1.352 | 1.424 | 1.488 | 1.545 | 1.597 |
| 19.7 | 2.173 | 0.043 | 1.361 | 1.431 | 1.496 | 1.559 | 1.619 | 3.556 | 0.040 | 1.352 | 1.424 | 1.487 | 1.545 | 1.597 |
| 19.8 | 2.245 | 0.043 | 1.361 | 1.431 | 1.497 | 1.560 | 1.619 | 3.568 | 0.040 | 1.353 | 1.424 | 1.487 | 1.544 | 1.596 |
| 19.9 | 2.317 | 0.043 | 1.361 | 1.431 | 1.498 | 1.560 | 1.620 | 3.579 | 0.040 | 1.353 | 1.424 | 1.487 | 1.544 | 1.595 |
| 20.0 | 2.389 | 0.043 | 1.360 | 1.432 | 1.498 | 1.561 | 1.620 | 3.589 | 0.040 | 1.354 | 1.424 | 1.487 | 1.543 | 1.595 |

| L1(L2)L3 | | | | | | | | | | | | | | |
| --- | --- | --- | --- | --- | --- | --- | --- | --- | --- | --- | --- | --- | --- | --- |
|  | Males | | | | | | | Females | | | | | | |
| Age | L | S | -2 SD | -1 SD | 0 SD (M) | 1 SD | 2 SD | L | S | -2 SD | -1 SD | 0 SD (M) | 1 SD | 2 SD |
| 5.0 | 3.201 | 0.063 | 1.062 | 1.163 | 1.248 | 1.322 | 1.387 | 1.590 | 0.062 | 1.068 | 1.149 | 1.227 | 1.302 | 1.375 |
| 5.1 | 3.168 | 0.063 | 1.064 | 1.164 | 1.249 | 1.322 | 1.388 | 1.590 | 0.062 | 1.068 | 1.149 | 1.227 | 1.302 | 1.374 |
| 5.2 | 3.136 | 0.063 | 1.065 | 1.165 | 1.249 | 1.323 | 1.389 | 1.591 | 0.062 | 1.067 | 1.148 | 1.226 | 1.301 | 1.374 |
| 5.3 | 3.103 | 0.063 | 1.067 | 1.166 | 1.250 | 1.324 | 1.390 | 1.591 | 0.062 | 1.067 | 1.148 | 1.226 | 1.301 | 1.373 |
| 5.4 | 3.071 | 0.063 | 1.068 | 1.167 | 1.251 | 1.324 | 1.390 | 1.591 | 0.062 | 1.066 | 1.147 | 1.225 | 1.300 | 1.373 |
| 5.5 | 3.039 | 0.062 | 1.070 | 1.168 | 1.252 | 1.325 | 1.391 | 1.591 | 0.062 | 1.066 | 1.147 | 1.225 | 1.300 | 1.372 |
| 5.6 | 3.006 | 0.062 | 1.071 | 1.169 | 1.252 | 1.326 | 1.392 | 1.591 | 0.062 | 1.066 | 1.147 | 1.224 | 1.299 | 1.371 |
| 5.7 | 2.974 | 0.062 | 1.072 | 1.170 | 1.253 | 1.326 | 1.393 | 1.591 | 0.062 | 1.065 | 1.146 | 1.224 | 1.299 | 1.371 |
| 5.8 | 2.943 | 0.062 | 1.074 | 1.170 | 1.253 | 1.327 | 1.394 | 1.591 | 0.062 | 1.065 | 1.146 | 1.223 | 1.298 | 1.370 |
| 5.9 | 2.911 | 0.062 | 1.075 | 1.171 | 1.254 | 1.328 | 1.394 | 1.591 | 0.062 | 1.064 | 1.145 | 1.223 | 1.298 | 1.370 |
| 6.0 | 2.879 | 0.062 | 1.076 | 1.172 | 1.255 | 1.328 | 1.395 | 1.591 | 0.062 | 1.064 | 1.145 | 1.222 | 1.297 | 1.370 |
| 6.1 | 2.848 | 0.062 | 1.077 | 1.172 | 1.255 | 1.329 | 1.395 | 1.592 | 0.062 | 1.064 | 1.144 | 1.222 | 1.297 | 1.369 |
| 6.2 | 2.817 | 0.062 | 1.079 | 1.173 | 1.255 | 1.329 | 1.396 | 1.592 | 0.062 | 1.063 | 1.144 | 1.222 | 1.296 | 1.369 |
| 6.3 | 2.786 | 0.062 | 1.080 | 1.174 | 1.256 | 1.329 | 1.396 | 1.592 | 0.062 | 1.063 | 1.144 | 1.221 | 1.296 | 1.368 |
| 6.4 | 2.755 | 0.062 | 1.081 | 1.174 | 1.256 | 1.330 | 1.397 | 1.592 | 0.062 | 1.063 | 1.144 | 1.221 | 1.296 | 1.368 |
| 6.5 | 2.725 | 0.062 | 1.082 | 1.175 | 1.257 | 1.330 | 1.397 | 1.592 | 0.062 | 1.063 | 1.143 | 1.221 | 1.295 | 1.368 |
| 6.6 | 2.694 | 0.061 | 1.083 | 1.175 | 1.257 | 1.330 | 1.398 | 1.592 | 0.062 | 1.062 | 1.143 | 1.221 | 1.295 | 1.367 |
| 6.7 | 2.664 | 0.061 | 1.084 | 1.176 | 1.257 | 1.331 | 1.398 | 1.592 | 0.062 | 1.062 | 1.143 | 1.220 | 1.295 | 1.367 |
| 6.8 | 2.634 | 0.061 | 1.084 | 1.176 | 1.257 | 1.331 | 1.398 | 1.592 | 0.062 | 1.062 | 1.143 | 1.220 | 1.295 | 1.367 |
| 6.9 | 2.604 | 0.061 | 1.085 | 1.176 | 1.258 | 1.331 | 1.398 | 1.593 | 0.062 | 1.062 | 1.143 | 1.220 | 1.295 | 1.367 |
| 7.0 | 2.574 | 0.061 | 1.086 | 1.177 | 1.258 | 1.331 | 1.399 | 1.593 | 0.062 | 1.062 | 1.143 | 1.220 | 1.295 | 1.367 |
| 7.1 | 2.544 | 0.061 | 1.087 | 1.177 | 1.258 | 1.331 | 1.399 | 1.593 | 0.062 | 1.062 | 1.143 | 1.220 | 1.295 | 1.367 |
| 7.2 | 2.515 | 0.061 | 1.087 | 1.177 | 1.258 | 1.331 | 1.399 | 1.593 | 0.062 | 1.062 | 1.143 | 1.220 | 1.295 | 1.367 |
| 7.3 | 2.485 | 0.061 | 1.088 | 1.178 | 1.258 | 1.331 | 1.399 | 1.593 | 0.062 | 1.062 | 1.143 | 1.220 | 1.295 | 1.367 |
| 7.4 | 2.456 | 0.061 | 1.089 | 1.178 | 1.258 | 1.331 | 1.399 | 1.593 | 0.062 | 1.062 | 1.143 | 1.220 | 1.295 | 1.367 |
| 7.5 | 2.427 | 0.061 | 1.089 | 1.178 | 1.258 | 1.331 | 1.399 | 1.593 | 0.062 | 1.062 | 1.143 | 1.220 | 1.295 | 1.367 |
| 7.6 | 2.398 | 0.061 | 1.090 | 1.178 | 1.258 | 1.331 | 1.399 | 1.593 | 0.062 | 1.062 | 1.143 | 1.220 | 1.295 | 1.367 |
| 7.7 | 2.369 | 0.061 | 1.090 | 1.178 | 1.258 | 1.331 | 1.399 | 1.594 | 0.062 | 1.063 | 1.143 | 1.220 | 1.295 | 1.367 |
| 7.8 | 2.340 | 0.061 | 1.091 | 1.178 | 1.258 | 1.331 | 1.399 | 1.594 | 0.062 | 1.063 | 1.143 | 1.220 | 1.295 | 1.367 |
| 7.9 | 2.311 | 0.061 | 1.091 | 1.179 | 1.258 | 1.331 | 1.400 | 1.594 | 0.062 | 1.063 | 1.143 | 1.220 | 1.295 | 1.367 |
| 8.0 | 2.282 | 0.060 | 1.092 | 1.179 | 1.258 | 1.331 | 1.400 | 1.594 | 0.062 | 1.063 | 1.143 | 1.221 | 1.295 | 1.367 |
| 8.1 | 2.254 | 0.060 | 1.092 | 1.179 | 1.258 | 1.331 | 1.400 | 1.594 | 0.062 | 1.063 | 1.144 | 1.221 | 1.295 | 1.367 |
| 8.2 | 2.225 | 0.060 | 1.093 | 1.179 | 1.258 | 1.331 | 1.400 | 1.594 | 0.062 | 1.064 | 1.144 | 1.221 | 1.295 | 1.367 |
| 8.3 | 2.197 | 0.060 | 1.093 | 1.179 | 1.258 | 1.331 | 1.400 | 1.594 | 0.062 | 1.064 | 1.144 | 1.221 | 1.295 | 1.367 |
| 8.4 | 2.168 | 0.060 | 1.094 | 1.179 | 1.258 | 1.331 | 1.400 | 1.594 | 0.062 | 1.064 | 1.144 | 1.221 | 1.296 | 1.367 |
| 8.5 | 2.140 | 0.060 | 1.094 | 1.179 | 1.258 | 1.331 | 1.400 | 1.595 | 0.062 | 1.065 | 1.145 | 1.222 | 1.296 | 1.368 |
| 8.6 | 2.112 | 0.060 | 1.095 | 1.179 | 1.258 | 1.331 | 1.400 | 1.595 | 0.062 | 1.065 | 1.145 | 1.222 | 1.296 | 1.368 |
| 8.7 | 2.084 | 0.060 | 1.095 | 1.180 | 1.258 | 1.331 | 1.400 | 1.595 | 0.062 | 1.065 | 1.146 | 1.222 | 1.296 | 1.368 |
| 8.8 | 2.056 | 0.060 | 1.096 | 1.180 | 1.258 | 1.331 | 1.400 | 1.595 | 0.062 | 1.066 | 1.146 | 1.223 | 1.297 | 1.369 |
| 8.9 | 2.028 | 0.060 | 1.096 | 1.180 | 1.258 | 1.331 | 1.401 | 1.595 | 0.062 | 1.067 | 1.147 | 1.223 | 1.297 | 1.369 |
| 9.0 | 2.000 | 0.060 | 1.096 | 1.180 | 1.258 | 1.331 | 1.401 | 1.595 | 0.062 | 1.067 | 1.147 | 1.224 | 1.298 | 1.369 |
| 9.1 | 1.973 | 0.060 | 1.097 | 1.180 | 1.258 | 1.331 | 1.401 | 1.595 | 0.061 | 1.068 | 1.148 | 1.224 | 1.298 | 1.370 |
| 9.2 | 1.945 | 0.060 | 1.097 | 1.180 | 1.258 | 1.331 | 1.401 | 1.595 | 0.061 | 1.069 | 1.148 | 1.225 | 1.299 | 1.371 |
| 9.3 | 1.918 | 0.060 | 1.098 | 1.180 | 1.258 | 1.331 | 1.401 | 1.596 | 0.061 | 1.069 | 1.149 | 1.226 | 1.300 | 1.371 |
| 9.4 | 1.891 | 0.060 | 1.098 | 1.180 | 1.258 | 1.331 | 1.401 | 1.596 | 0.061 | 1.070 | 1.150 | 1.227 | 1.301 | 1.372 |
| 9.5 | 1.864 | 0.060 | 1.098 | 1.181 | 1.258 | 1.331 | 1.402 | 1.596 | 0.061 | 1.071 | 1.151 | 1.228 | 1.302 | 1.373 |
| 9.6 | 1.837 | 0.060 | 1.099 | 1.181 | 1.258 | 1.332 | 1.402 | 1.596 | 0.061 | 1.072 | 1.152 | 1.229 | 1.303 | 1.374 |
| 9.7 | 1.810 | 0.060 | 1.099 | 1.181 | 1.258 | 1.332 | 1.402 | 1.596 | 0.061 | 1.073 | 1.153 | 1.230 | 1.304 | 1.375 |
| 9.8 | 1.784 | 0.060 | 1.100 | 1.181 | 1.258 | 1.332 | 1.402 | 1.596 | 0.061 | 1.075 | 1.155 | 1.231 | 1.305 | 1.377 |
| 9.9 | 1.758 | 0.060 | 1.100 | 1.181 | 1.258 | 1.332 | 1.402 | 1.596 | 0.061 | 1.076 | 1.156 | 1.233 | 1.306 | 1.378 |
| 10.0 | 1.732 | 0.060 | 1.100 | 1.181 | 1.258 | 1.332 | 1.403 | 1.596 | 0.061 | 1.078 | 1.157 | 1.234 | 1.308 | 1.379 |
| 10.1 | 1.706 | 0.060 | 1.101 | 1.181 | 1.258 | 1.332 | 1.403 | 1.596 | 0.061 | 1.079 | 1.159 | 1.236 | 1.309 | 1.381 |
| 10.2 | 1.680 | 0.060 | 1.101 | 1.181 | 1.258 | 1.332 | 1.403 | 1.597 | 0.061 | 1.081 | 1.161 | 1.237 | 1.311 | 1.383 |
| 10.3 | 1.654 | 0.060 | 1.101 | 1.181 | 1.258 | 1.332 | 1.403 | 1.597 | 0.061 | 1.083 | 1.162 | 1.239 | 1.313 | 1.385 |
| 10.4 | 1.629 | 0.060 | 1.102 | 1.182 | 1.258 | 1.332 | 1.404 | 1.597 | 0.061 | 1.085 | 1.164 | 1.241 | 1.315 | 1.386 |
| 10.5 | 1.604 | 0.060 | 1.102 | 1.182 | 1.258 | 1.332 | 1.404 | 1.597 | 0.061 | 1.087 | 1.166 | 1.243 | 1.317 | 1.389 |
| 10.6 | 1.579 | 0.060 | 1.102 | 1.182 | 1.259 | 1.333 | 1.404 | 1.597 | 0.060 | 1.089 | 1.169 | 1.245 | 1.319 | 1.391 |
| 10.7 | 1.555 | 0.060 | 1.103 | 1.182 | 1.259 | 1.333 | 1.405 | 1.597 | 0.060 | 1.091 | 1.171 | 1.248 | 1.322 | 1.393 |
| 10.8 | 1.531 | 0.060 | 1.103 | 1.183 | 1.259 | 1.333 | 1.405 | 1.597 | 0.060 | 1.094 | 1.173 | 1.250 | 1.324 | 1.396 |
| 10.9 | 1.507 | 0.060 | 1.104 | 1.183 | 1.259 | 1.333 | 1.406 | 1.597 | 0.060 | 1.096 | 1.176 | 1.253 | 1.327 | 1.399 |
| 11.0 | 1.483 | 0.060 | 1.104 | 1.183 | 1.260 | 1.334 | 1.406 | 1.598 | 0.060 | 1.099 | 1.179 | 1.256 | 1.330 | 1.402 |
| 11.1 | 1.460 | 0.060 | 1.105 | 1.184 | 1.260 | 1.334 | 1.407 | 1.598 | 0.060 | 1.102 | 1.182 | 1.259 | 1.333 | 1.405 |
| 11.2 | 1.437 | 0.060 | 1.106 | 1.184 | 1.260 | 1.335 | 1.407 | 1.598 | 0.060 | 1.105 | 1.185 | 1.262 | 1.336 | 1.408 |
| 11.3 | 1.414 | 0.060 | 1.107 | 1.185 | 1.261 | 1.335 | 1.408 | 1.598 | 0.060 | 1.108 | 1.188 | 1.265 | 1.339 | 1.411 |
| 11.4 | 1.392 | 0.060 | 1.107 | 1.185 | 1.261 | 1.336 | 1.408 | 1.598 | 0.060 | 1.112 | 1.192 | 1.269 | 1.343 | 1.415 |
| 11.5 | 1.370 | 0.060 | 1.108 | 1.186 | 1.262 | 1.336 | 1.409 | 1.598 | 0.059 | 1.115 | 1.195 | 1.272 | 1.346 | 1.418 |
| 11.6 | 1.348 | 0.059 | 1.109 | 1.187 | 1.263 | 1.337 | 1.410 | 1.598 | 0.059 | 1.119 | 1.199 | 1.276 | 1.350 | 1.422 |
| 11.7 | 1.327 | 0.059 | 1.110 | 1.188 | 1.263 | 1.338 | 1.411 | 1.598 | 0.059 | 1.122 | 1.203 | 1.280 | 1.354 | 1.426 |
| 11.8 | 1.306 | 0.059 | 1.111 | 1.189 | 1.264 | 1.339 | 1.412 | 1.599 | 0.059 | 1.126 | 1.207 | 1.284 | 1.358 | 1.430 |
| 11.9 | 1.286 | 0.059 | 1.112 | 1.190 | 1.265 | 1.340 | 1.413 | 1.599 | 0.059 | 1.130 | 1.211 | 1.288 | 1.362 | 1.434 |
| 12.0 | 1.266 | 0.059 | 1.114 | 1.191 | 1.266 | 1.341 | 1.414 | 1.599 | 0.059 | 1.134 | 1.215 | 1.292 | 1.366 | 1.439 |
| 12.1 | 1.246 | 0.059 | 1.115 | 1.192 | 1.267 | 1.342 | 1.415 | 1.599 | 0.058 | 1.139 | 1.219 | 1.296 | 1.371 | 1.443 |
| 12.2 | 1.226 | 0.059 | 1.117 | 1.193 | 1.269 | 1.343 | 1.417 | 1.599 | 0.058 | 1.143 | 1.223 | 1.300 | 1.375 | 1.447 |
| 12.3 | 1.207 | 0.059 | 1.118 | 1.195 | 1.270 | 1.345 | 1.418 | 1.599 | 0.058 | 1.147 | 1.228 | 1.305 | 1.379 | 1.452 |
| 12.4 | 1.189 | 0.059 | 1.120 | 1.197 | 1.272 | 1.346 | 1.420 | 1.599 | 0.058 | 1.152 | 1.232 | 1.309 | 1.384 | 1.456 |
| 12.5 | 1.170 | 0.059 | 1.122 | 1.198 | 1.274 | 1.348 | 1.422 | 1.599 | 0.058 | 1.156 | 1.236 | 1.314 | 1.388 | 1.461 |
| 12.6 | 1.152 | 0.059 | 1.124 | 1.200 | 1.275 | 1.350 | 1.424 | 1.600 | 0.058 | 1.161 | 1.241 | 1.318 | 1.393 | 1.465 |
| 12.7 | 1.135 | 0.059 | 1.127 | 1.202 | 1.277 | 1.352 | 1.426 | 1.600 | 0.057 | 1.165 | 1.245 | 1.322 | 1.397 | 1.469 |
| 12.8 | 1.118 | 0.058 | 1.129 | 1.205 | 1.280 | 1.354 | 1.428 | 1.600 | 0.057 | 1.169 | 1.250 | 1.327 | 1.401 | 1.474 |
| 12.9 | 1.101 | 0.058 | 1.132 | 1.207 | 1.282 | 1.356 | 1.431 | 1.600 | 0.057 | 1.174 | 1.254 | 1.331 | 1.406 | 1.478 |
| 13.0 | 1.085 | 0.058 | 1.134 | 1.210 | 1.284 | 1.359 | 1.433 | 1.600 | 0.057 | 1.178 | 1.258 | 1.335 | 1.410 | 1.482 |
| 13.1 | 1.069 | 0.058 | 1.137 | 1.212 | 1.287 | 1.362 | 1.436 | 1.600 | 0.057 | 1.182 | 1.262 | 1.340 | 1.414 | 1.486 |
| 13.2 | 1.054 | 0.058 | 1.140 | 1.215 | 1.290 | 1.364 | 1.439 | 1.600 | 0.056 | 1.187 | 1.267 | 1.344 | 1.418 | 1.490 |
| 13.3 | 1.039 | 0.058 | 1.143 | 1.218 | 1.293 | 1.367 | 1.441 | 1.600 | 0.056 | 1.191 | 1.271 | 1.348 | 1.422 | 1.494 |
| 13.4 | 1.025 | 0.057 | 1.146 | 1.221 | 1.296 | 1.370 | 1.444 | 1.601 | 0.056 | 1.195 | 1.275 | 1.351 | 1.426 | 1.498 |
| 13.5 | 1.011 | 0.057 | 1.150 | 1.224 | 1.299 | 1.373 | 1.448 | 1.601 | 0.056 | 1.199 | 1.279 | 1.355 | 1.430 | 1.502 |
| 13.6 | 0.999 | 0.057 | 1.153 | 1.228 | 1.302 | 1.376 | 1.451 | 1.601 | 0.055 | 1.203 | 1.282 | 1.359 | 1.433 | 1.505 |
| 13.7 | 0.986 | 0.057 | 1.157 | 1.231 | 1.305 | 1.380 | 1.454 | 1.601 | 0.055 | 1.206 | 1.286 | 1.362 | 1.436 | 1.508 |
| 13.8 | 0.974 | 0.057 | 1.161 | 1.235 | 1.309 | 1.383 | 1.458 | 1.601 | 0.055 | 1.210 | 1.289 | 1.366 | 1.440 | 1.512 |
| 13.9 | 0.963 | 0.056 | 1.165 | 1.238 | 1.313 | 1.387 | 1.461 | 1.601 | 0.055 | 1.214 | 1.293 | 1.369 | 1.443 | 1.515 |
| 14.0 | 0.953 | 0.056 | 1.168 | 1.242 | 1.316 | 1.390 | 1.465 | 1.601 | 0.055 | 1.217 | 1.296 | 1.372 | 1.446 | 1.517 |
| 14.1 | 0.943 | 0.056 | 1.172 | 1.246 | 1.320 | 1.394 | 1.468 | 1.601 | 0.054 | 1.220 | 1.299 | 1.375 | 1.449 | 1.520 |
| 14.2 | 0.934 | 0.056 | 1.176 | 1.250 | 1.323 | 1.397 | 1.472 | 1.601 | 0.054 | 1.223 | 1.302 | 1.378 | 1.451 | 1.523 |
| 14.3 | 0.926 | 0.056 | 1.180 | 1.254 | 1.327 | 1.401 | 1.475 | 1.602 | 0.054 | 1.226 | 1.305 | 1.381 | 1.454 | 1.525 |
| 14.4 | 0.919 | 0.055 | 1.184 | 1.257 | 1.331 | 1.405 | 1.479 | 1.602 | 0.054 | 1.229 | 1.308 | 1.383 | 1.457 | 1.528 |
| 14.5 | 0.912 | 0.055 | 1.188 | 1.261 | 1.335 | 1.408 | 1.482 | 1.602 | 0.054 | 1.232 | 1.310 | 1.386 | 1.459 | 1.530 |
| 14.6 | 0.906 | 0.055 | 1.192 | 1.265 | 1.338 | 1.412 | 1.486 | 1.602 | 0.053 | 1.235 | 1.313 | 1.388 | 1.461 | 1.532 |
| 14.7 | 0.901 | 0.055 | 1.197 | 1.269 | 1.342 | 1.416 | 1.489 | 1.602 | 0.053 | 1.238 | 1.315 | 1.391 | 1.463 | 1.534 |
| 14.8 | 0.897 | 0.054 | 1.201 | 1.273 | 1.346 | 1.419 | 1.493 | 1.602 | 0.053 | 1.240 | 1.318 | 1.393 | 1.465 | 1.536 |
| 14.9 | 0.893 | 0.054 | 1.205 | 1.277 | 1.350 | 1.423 | 1.496 | 1.602 | 0.053 | 1.243 | 1.320 | 1.395 | 1.467 | 1.538 |
| 15.0 | 0.890 | 0.054 | 1.209 | 1.281 | 1.353 | 1.426 | 1.500 | 1.602 | 0.053 | 1.245 | 1.322 | 1.397 | 1.469 | 1.539 |
| 15.1 | 0.888 | 0.054 | 1.213 | 1.285 | 1.357 | 1.430 | 1.503 | 1.603 | 0.052 | 1.247 | 1.324 | 1.399 | 1.471 | 1.541 |
| 15.2 | 0.887 | 0.053 | 1.217 | 1.288 | 1.361 | 1.433 | 1.506 | 1.603 | 0.052 | 1.250 | 1.326 | 1.401 | 1.473 | 1.543 |
| 15.3 | 0.887 | 0.053 | 1.221 | 1.292 | 1.364 | 1.437 | 1.510 | 1.603 | 0.052 | 1.252 | 1.328 | 1.403 | 1.474 | 1.544 |
| 15.4 | 0.887 | 0.053 | 1.224 | 1.296 | 1.368 | 1.440 | 1.513 | 1.603 | 0.052 | 1.254 | 1.330 | 1.404 | 1.476 | 1.546 |
| 15.5 | 0.888 | 0.052 | 1.228 | 1.300 | 1.371 | 1.443 | 1.516 | 1.603 | 0.052 | 1.256 | 1.332 | 1.406 | 1.477 | 1.547 |
| 15.6 | 0.890 | 0.052 | 1.232 | 1.303 | 1.375 | 1.446 | 1.519 | 1.603 | 0.051 | 1.258 | 1.334 | 1.408 | 1.479 | 1.548 |
| 15.7 | 0.893 | 0.052 | 1.236 | 1.307 | 1.378 | 1.449 | 1.521 | 1.603 | 0.051 | 1.260 | 1.336 | 1.409 | 1.480 | 1.549 |
| 15.8 | 0.897 | 0.052 | 1.239 | 1.310 | 1.381 | 1.453 | 1.524 | 1.603 | 0.051 | 1.261 | 1.337 | 1.410 | 1.482 | 1.551 |
| 15.9 | 0.901 | 0.051 | 1.243 | 1.313 | 1.384 | 1.455 | 1.527 | 1.604 | 0.051 | 1.263 | 1.339 | 1.412 | 1.483 | 1.552 |
| 16.0 | 0.907 | 0.051 | 1.247 | 1.317 | 1.387 | 1.458 | 1.530 | 1.604 | 0.051 | 1.265 | 1.340 | 1.413 | 1.484 | 1.553 |
| 16.1 | 0.913 | 0.051 | 1.250 | 1.320 | 1.390 | 1.461 | 1.532 | 1.604 | 0.051 | 1.266 | 1.342 | 1.415 | 1.485 | 1.554 |
| 16.2 | 0.920 | 0.050 | 1.253 | 1.323 | 1.393 | 1.464 | 1.535 | 1.604 | 0.051 | 1.268 | 1.343 | 1.416 | 1.486 | 1.555 |
| 16.3 | 0.928 | 0.050 | 1.256 | 1.326 | 1.396 | 1.466 | 1.537 | 1.604 | 0.050 | 1.270 | 1.345 | 1.417 | 1.487 | 1.556 |
| 16.4 | 0.936 | 0.050 | 1.260 | 1.329 | 1.399 | 1.469 | 1.539 | 1.604 | 0.050 | 1.271 | 1.346 | 1.418 | 1.488 | 1.557 |
| 16.5 | 0.946 | 0.050 | 1.263 | 1.332 | 1.402 | 1.471 | 1.541 | 1.604 | 0.050 | 1.273 | 1.347 | 1.419 | 1.489 | 1.557 |
| 16.6 | 0.956 | 0.049 | 1.266 | 1.335 | 1.404 | 1.474 | 1.543 | 1.604 | 0.050 | 1.274 | 1.348 | 1.420 | 1.490 | 1.558 |
| 16.7 | 0.967 | 0.049 | 1.268 | 1.337 | 1.407 | 1.476 | 1.545 | 1.605 | 0.050 | 1.276 | 1.350 | 1.422 | 1.491 | 1.559 |
| 16.8 | 0.979 | 0.049 | 1.271 | 1.340 | 1.409 | 1.478 | 1.547 | 1.605 | 0.050 | 1.277 | 1.351 | 1.423 | 1.492 | 1.560 |
| 16.9 | 0.992 | 0.049 | 1.274 | 1.343 | 1.411 | 1.480 | 1.549 | 1.605 | 0.049 | 1.278 | 1.352 | 1.424 | 1.493 | 1.561 |
| 17.0 | 1.006 | 0.048 | 1.276 | 1.345 | 1.413 | 1.482 | 1.551 | 1.605 | 0.049 | 1.280 | 1.353 | 1.425 | 1.494 | 1.561 |
| 17.1 | 1.020 | 0.048 | 1.279 | 1.347 | 1.416 | 1.484 | 1.552 | 1.605 | 0.049 | 1.281 | 1.355 | 1.426 | 1.495 | 1.562 |
| 17.2 | 1.035 | 0.048 | 1.281 | 1.349 | 1.418 | 1.486 | 1.554 | 1.605 | 0.049 | 1.282 | 1.356 | 1.427 | 1.496 | 1.563 |
| 17.3 | 1.051 | 0.048 | 1.283 | 1.351 | 1.419 | 1.487 | 1.555 | 1.605 | 0.049 | 1.284 | 1.357 | 1.428 | 1.497 | 1.564 |
| 17.4 | 1.068 | 0.048 | 1.285 | 1.353 | 1.421 | 1.489 | 1.556 | 1.605 | 0.049 | 1.285 | 1.358 | 1.429 | 1.497 | 1.564 |
| 17.5 | 1.086 | 0.047 | 1.287 | 1.355 | 1.423 | 1.490 | 1.558 | 1.606 | 0.049 | 1.286 | 1.359 | 1.430 | 1.498 | 1.565 |
| 17.6 | 1.104 | 0.047 | 1.289 | 1.357 | 1.425 | 1.492 | 1.559 | 1.606 | 0.048 | 1.288 | 1.360 | 1.431 | 1.499 | 1.566 |
| 17.7 | 1.124 | 0.047 | 1.291 | 1.359 | 1.426 | 1.493 | 1.560 | 1.606 | 0.048 | 1.289 | 1.361 | 1.432 | 1.500 | 1.566 |
| 17.8 | 1.144 | 0.047 | 1.293 | 1.361 | 1.428 | 1.494 | 1.561 | 1.606 | 0.048 | 1.290 | 1.362 | 1.432 | 1.500 | 1.567 |
| 17.9 | 1.164 | 0.047 | 1.295 | 1.362 | 1.429 | 1.496 | 1.562 | 1.606 | 0.048 | 1.291 | 1.363 | 1.433 | 1.501 | 1.567 |
| 18.0 | 1.185 | 0.047 | 1.296 | 1.364 | 1.431 | 1.497 | 1.563 | 1.606 | 0.048 | 1.292 | 1.364 | 1.434 | 1.502 | 1.568 |
| 18.1 | 1.207 | 0.046 | 1.298 | 1.365 | 1.432 | 1.498 | 1.564 | 1.606 | 0.048 | 1.293 | 1.365 | 1.435 | 1.502 | 1.568 |
| 18.2 | 1.230 | 0.046 | 1.299 | 1.366 | 1.433 | 1.499 | 1.564 | 1.606 | 0.048 | 1.295 | 1.366 | 1.435 | 1.503 | 1.569 |
| 18.3 | 1.253 | 0.046 | 1.300 | 1.368 | 1.434 | 1.500 | 1.565 | 1.607 | 0.047 | 1.296 | 1.367 | 1.436 | 1.503 | 1.569 |
| 18.4 | 1.277 | 0.046 | 1.302 | 1.369 | 1.435 | 1.501 | 1.566 | 1.607 | 0.047 | 1.297 | 1.368 | 1.437 | 1.504 | 1.569 |
| 18.5 | 1.302 | 0.046 | 1.303 | 1.370 | 1.437 | 1.502 | 1.567 | 1.607 | 0.047 | 1.297 | 1.368 | 1.437 | 1.504 | 1.569 |
| 18.6 | 1.327 | 0.046 | 1.304 | 1.371 | 1.438 | 1.503 | 1.567 | 1.607 | 0.047 | 1.298 | 1.369 | 1.438 | 1.504 | 1.569 |
| 18.7 | 1.352 | 0.046 | 1.305 | 1.372 | 1.439 | 1.504 | 1.568 | 1.607 | 0.047 | 1.299 | 1.370 | 1.438 | 1.505 | 1.570 |
| 18.8 | 1.378 | 0.046 | 1.306 | 1.373 | 1.440 | 1.505 | 1.569 | 1.607 | 0.047 | 1.300 | 1.370 | 1.439 | 1.505 | 1.570 |
| 18.9 | 1.405 | 0.045 | 1.307 | 1.375 | 1.441 | 1.505 | 1.569 | 1.607 | 0.047 | 1.301 | 1.371 | 1.439 | 1.505 | 1.569 |
| 19.0 | 1.432 | 0.045 | 1.308 | 1.376 | 1.442 | 1.506 | 1.570 | 1.607 | 0.046 | 1.301 | 1.371 | 1.439 | 1.505 | 1.569 |
| 19.1 | 1.459 | 0.045 | 1.309 | 1.377 | 1.442 | 1.507 | 1.570 | 1.607 | 0.046 | 1.302 | 1.372 | 1.439 | 1.505 | 1.569 |
| 19.2 | 1.487 | 0.045 | 1.310 | 1.378 | 1.443 | 1.508 | 1.571 | 1.608 | 0.046 | 1.303 | 1.372 | 1.439 | 1.505 | 1.569 |
| 19.3 | 1.515 | 0.045 | 1.311 | 1.378 | 1.444 | 1.509 | 1.572 | 1.608 | 0.046 | 1.303 | 1.372 | 1.440 | 1.505 | 1.569 |
| 19.4 | 1.544 | 0.045 | 1.312 | 1.379 | 1.445 | 1.509 | 1.572 | 1.608 | 0.046 | 1.304 | 1.373 | 1.440 | 1.505 | 1.568 |
| 19.5 | 1.573 | 0.045 | 1.313 | 1.380 | 1.446 | 1.510 | 1.573 | 1.608 | 0.046 | 1.304 | 1.373 | 1.440 | 1.505 | 1.568 |
| 19.6 | 1.602 | 0.045 | 1.313 | 1.381 | 1.447 | 1.511 | 1.573 | 1.608 | 0.046 | 1.305 | 1.373 | 1.440 | 1.504 | 1.567 |
| 19.7 | 1.631 | 0.045 | 1.314 | 1.382 | 1.448 | 1.511 | 1.574 | 1.608 | 0.045 | 1.305 | 1.373 | 1.440 | 1.504 | 1.567 |
| 19.8 | 1.661 | 0.045 | 1.315 | 1.383 | 1.448 | 1.512 | 1.574 | 1.608 | 0.045 | 1.305 | 1.373 | 1.440 | 1.504 | 1.567 |
| 19.9 | 1.691 | 0.045 | 1.315 | 1.383 | 1.449 | 1.513 | 1.574 | 1.608 | 0.045 | 1.306 | 1.374 | 1.439 | 1.504 | 1.566 |
| 20.0 | 1.721 | 0.045 | 1.316 | 1.384 | 1.450 | 1.513 | 1.575 | 1.609 | 0.045 | 1.306 | 1.374 | 1.439 | 1.503 | 1.566 |

| L1(L2)(L3)L4 | | | | | | | | | | | | | | |
| --- | --- | --- | --- | --- | --- | --- | --- | --- | --- | --- | --- | --- | --- | --- |
|  | Males | | | | | | | Females | | | | | | |
| Age | L | S | -2 SD | -1 SD | 0 SD (M) | 1 SD | 2 SD | L | S | -2 SD | -1 SD | 0 SD (M) | 1 SD | 2 SD |
| 5.0 | 2.634 | 0.066 | 1.075 | 1.175 | 1.263 | 1.342 | 2.634 | 2.623 | 0.073 | 1.025 | 1.136 | 1.232 | 1.317 | 1.394 |
| 5.1 | 2.622 | 0.066 | 1.075 | 1.175 | 1.263 | 1.342 | 2.622 | 2.607 | 0.073 | 1.025 | 1.136 | 1.231 | 1.316 | 1.393 |
| 5.2 | 2.610 | 0.066 | 1.076 | 1.176 | 1.263 | 1.342 | 2.610 | 2.590 | 0.073 | 1.026 | 1.136 | 1.231 | 1.316 | 1.392 |
| 5.3 | 2.598 | 0.066 | 1.076 | 1.176 | 1.264 | 1.342 | 2.598 | 2.574 | 0.072 | 1.027 | 1.136 | 1.230 | 1.315 | 1.392 |
| 5.4 | 2.586 | 0.066 | 1.076 | 1.176 | 1.264 | 1.343 | 2.586 | 2.558 | 0.072 | 1.027 | 1.136 | 1.230 | 1.314 | 1.391 |
| 5.5 | 2.574 | 0.066 | 1.077 | 1.176 | 1.264 | 1.343 | 2.574 | 2.542 | 0.072 | 1.028 | 1.136 | 1.230 | 1.314 | 1.390 |
| 5.6 | 2.562 | 0.066 | 1.077 | 1.176 | 1.264 | 1.343 | 2.562 | 2.525 | 0.072 | 1.029 | 1.136 | 1.229 | 1.313 | 1.389 |
| 5.7 | 2.550 | 0.066 | 1.077 | 1.176 | 1.264 | 1.343 | 2.550 | 2.509 | 0.071 | 1.030 | 1.136 | 1.229 | 1.312 | 1.389 |
| 5.8 | 2.538 | 0.066 | 1.078 | 1.177 | 1.264 | 1.343 | 2.538 | 2.492 | 0.071 | 1.030 | 1.136 | 1.229 | 1.312 | 1.388 |
| 5.9 | 2.526 | 0.066 | 1.078 | 1.177 | 1.264 | 1.343 | 2.526 | 2.476 | 0.071 | 1.031 | 1.136 | 1.228 | 1.311 | 1.387 |
| 6.0 | 2.514 | 0.066 | 1.078 | 1.177 | 1.264 | 1.343 | 2.514 | 2.460 | 0.071 | 1.032 | 1.136 | 1.228 | 1.311 | 1.386 |
| 6.1 | 2.502 | 0.066 | 1.078 | 1.177 | 1.264 | 1.343 | 2.502 | 2.443 | 0.071 | 1.033 | 1.136 | 1.228 | 1.310 | 1.386 |
| 6.2 | 2.491 | 0.066 | 1.079 | 1.177 | 1.264 | 1.343 | 2.491 | 2.427 | 0.070 | 1.033 | 1.136 | 1.227 | 1.310 | 1.385 |
| 6.3 | 2.479 | 0.066 | 1.079 | 1.177 | 1.264 | 1.343 | 2.479 | 2.410 | 0.070 | 1.034 | 1.136 | 1.227 | 1.309 | 1.384 |
| 6.4 | 2.467 | 0.066 | 1.079 | 1.177 | 1.264 | 1.343 | 2.467 | 2.394 | 0.070 | 1.035 | 1.136 | 1.227 | 1.308 | 1.384 |
| 6.5 | 2.455 | 0.066 | 1.079 | 1.177 | 1.264 | 1.343 | 2.455 | 2.377 | 0.070 | 1.035 | 1.136 | 1.226 | 1.308 | 1.383 |
| 6.6 | 2.443 | 0.065 | 1.079 | 1.177 | 1.264 | 1.343 | 2.443 | 2.361 | 0.069 | 1.036 | 1.137 | 1.226 | 1.307 | 1.382 |
| 6.7 | 2.431 | 0.065 | 1.080 | 1.177 | 1.264 | 1.343 | 2.431 | 2.344 | 0.069 | 1.037 | 1.137 | 1.226 | 1.307 | 1.382 |
| 6.8 | 2.419 | 0.065 | 1.080 | 1.177 | 1.264 | 1.343 | 2.419 | 2.327 | 0.069 | 1.038 | 1.137 | 1.225 | 1.306 | 1.381 |
| 6.9 | 2.407 | 0.065 | 1.080 | 1.177 | 1.264 | 1.343 | 2.407 | 2.311 | 0.069 | 1.038 | 1.137 | 1.225 | 1.306 | 1.381 |
| 7.0 | 2.395 | 0.065 | 1.080 | 1.177 | 1.264 | 1.343 | 2.395 | 2.294 | 0.069 | 1.039 | 1.137 | 1.225 | 1.305 | 1.380 |
| 7.1 | 2.383 | 0.065 | 1.081 | 1.177 | 1.264 | 1.343 | 2.383 | 2.277 | 0.068 | 1.040 | 1.137 | 1.225 | 1.305 | 1.379 |
| 7.2 | 2.371 | 0.065 | 1.081 | 1.177 | 1.264 | 1.343 | 2.371 | 2.260 | 0.068 | 1.040 | 1.137 | 1.224 | 1.304 | 1.379 |
| 7.3 | 2.359 | 0.065 | 1.081 | 1.177 | 1.263 | 1.342 | 2.359 | 2.243 | 0.068 | 1.041 | 1.137 | 1.224 | 1.304 | 1.378 |
| 7.4 | 2.347 | 0.065 | 1.081 | 1.177 | 1.263 | 1.342 | 2.347 | 2.226 | 0.068 | 1.042 | 1.137 | 1.224 | 1.303 | 1.378 |
| 7.5 | 2.336 | 0.065 | 1.082 | 1.177 | 1.263 | 1.342 | 2.336 | 2.209 | 0.067 | 1.042 | 1.137 | 1.224 | 1.303 | 1.377 |
| 7.6 | 2.324 | 0.065 | 1.082 | 1.177 | 1.263 | 1.342 | 2.324 | 2.192 | 0.067 | 1.043 | 1.138 | 1.223 | 1.303 | 1.376 |
| 7.7 | 2.312 | 0.065 | 1.082 | 1.177 | 1.263 | 1.341 | 2.312 | 2.175 | 0.067 | 1.044 | 1.138 | 1.223 | 1.302 | 1.376 |
| 7.8 | 2.300 | 0.065 | 1.083 | 1.177 | 1.263 | 1.341 | 2.300 | 2.158 | 0.067 | 1.045 | 1.138 | 1.223 | 1.302 | 1.375 |
| 7.9 | 2.288 | 0.065 | 1.083 | 1.177 | 1.262 | 1.341 | 2.288 | 2.140 | 0.067 | 1.045 | 1.138 | 1.223 | 1.301 | 1.375 |
| 8.0 | 2.276 | 0.065 | 1.083 | 1.177 | 1.262 | 1.341 | 2.276 | 2.123 | 0.066 | 1.046 | 1.138 | 1.223 | 1.301 | 1.374 |
| 8.1 | 2.264 | 0.065 | 1.083 | 1.177 | 1.262 | 1.340 | 2.264 | 2.106 | 0.066 | 1.047 | 1.138 | 1.222 | 1.301 | 1.374 |
| 8.2 | 2.252 | 0.064 | 1.084 | 1.177 | 1.262 | 1.340 | 2.252 | 2.088 | 0.066 | 1.048 | 1.139 | 1.222 | 1.300 | 1.373 |
| 8.3 | 2.240 | 0.064 | 1.084 | 1.177 | 1.261 | 1.340 | 2.240 | 2.071 | 0.066 | 1.048 | 1.139 | 1.222 | 1.300 | 1.373 |
| 8.4 | 2.228 | 0.064 | 1.084 | 1.177 | 1.261 | 1.339 | 2.228 | 2.053 | 0.066 | 1.049 | 1.139 | 1.222 | 1.300 | 1.373 |
| 8.5 | 2.216 | 0.064 | 1.085 | 1.177 | 1.261 | 1.339 | 2.216 | 2.036 | 0.065 | 1.050 | 1.139 | 1.222 | 1.299 | 1.372 |
| 8.6 | 2.204 | 0.064 | 1.085 | 1.177 | 1.261 | 1.338 | 2.204 | 2.018 | 0.065 | 1.051 | 1.140 | 1.222 | 1.299 | 1.372 |
| 8.7 | 2.192 | 0.064 | 1.085 | 1.177 | 1.260 | 1.338 | 2.192 | 2.001 | 0.065 | 1.052 | 1.140 | 1.222 | 1.299 | 1.372 |
| 8.8 | 2.181 | 0.064 | 1.085 | 1.177 | 1.260 | 1.338 | 2.181 | 1.983 | 0.065 | 1.053 | 1.141 | 1.222 | 1.299 | 1.372 |
| 8.9 | 2.169 | 0.064 | 1.086 | 1.177 | 1.260 | 1.337 | 2.169 | 1.965 | 0.065 | 1.053 | 1.141 | 1.222 | 1.299 | 1.371 |
| 9.0 | 2.157 | 0.064 | 1.086 | 1.176 | 1.260 | 1.337 | 2.157 | 1.948 | 0.064 | 1.054 | 1.141 | 1.223 | 1.299 | 1.371 |
| 9.1 | 2.145 | 0.063 | 1.086 | 1.176 | 1.259 | 1.337 | 2.145 | 1.930 | 0.064 | 1.055 | 1.142 | 1.223 | 1.299 | 1.371 |
| 9.2 | 2.133 | 0.063 | 1.086 | 1.176 | 1.259 | 1.336 | 2.133 | 1.912 | 0.064 | 1.056 | 1.143 | 1.223 | 1.299 | 1.371 |
| 9.3 | 2.121 | 0.063 | 1.087 | 1.176 | 1.259 | 1.336 | 2.121 | 1.894 | 0.064 | 1.057 | 1.143 | 1.223 | 1.299 | 1.371 |
| 9.4 | 2.109 | 0.063 | 1.087 | 1.176 | 1.259 | 1.335 | 2.109 | 1.877 | 0.063 | 1.059 | 1.144 | 1.224 | 1.300 | 1.371 |
| 9.5 | 2.097 | 0.063 | 1.087 | 1.176 | 1.258 | 1.335 | 2.097 | 1.859 | 0.063 | 1.060 | 1.145 | 1.224 | 1.300 | 1.372 |
| 9.6 | 2.085 | 0.063 | 1.087 | 1.176 | 1.258 | 1.335 | 2.085 | 1.841 | 0.063 | 1.061 | 1.145 | 1.225 | 1.300 | 1.372 |
| 9.7 | 2.073 | 0.063 | 1.087 | 1.176 | 1.258 | 1.334 | 2.073 | 1.824 | 0.063 | 1.062 | 1.146 | 1.225 | 1.301 | 1.372 |
| 9.8 | 2.061 | 0.063 | 1.088 | 1.176 | 1.258 | 1.334 | 2.061 | 1.806 | 0.063 | 1.064 | 1.147 | 1.226 | 1.301 | 1.373 |
| 9.9 | 2.049 | 0.063 | 1.088 | 1.176 | 1.257 | 1.334 | 2.049 | 1.788 | 0.062 | 1.065 | 1.148 | 1.227 | 1.302 | 1.373 |
| 10.0 | 2.037 | 0.063 | 1.088 | 1.176 | 1.257 | 1.334 | 2.037 | 1.771 | 0.062 | 1.067 | 1.149 | 1.228 | 1.302 | 1.374 |
| 10.1 | 2.026 | 0.062 | 1.088 | 1.176 | 1.257 | 1.333 | 2.026 | 1.753 | 0.062 | 1.068 | 1.151 | 1.229 | 1.303 | 1.375 |
| 10.2 | 2.014 | 0.062 | 1.089 | 1.176 | 1.257 | 1.333 | 2.014 | 1.735 | 0.062 | 1.070 | 1.152 | 1.230 | 1.304 | 1.376 |
| 10.3 | 2.002 | 0.062 | 1.089 | 1.176 | 1.257 | 1.333 | 2.002 | 1.718 | 0.062 | 1.072 | 1.153 | 1.231 | 1.305 | 1.377 |
| 10.4 | 1.990 | 0.062 | 1.089 | 1.176 | 1.257 | 1.333 | 1.990 | 1.700 | 0.061 | 1.074 | 1.155 | 1.232 | 1.307 | 1.378 |
| 10.5 | 1.978 | 0.062 | 1.090 | 1.176 | 1.257 | 1.333 | 1.978 | 1.683 | 0.061 | 1.076 | 1.157 | 1.234 | 1.308 | 1.379 |
| 10.6 | 1.966 | 0.062 | 1.090 | 1.176 | 1.257 | 1.333 | 1.966 | 1.665 | 0.061 | 1.078 | 1.159 | 1.236 | 1.310 | 1.381 |
| 10.7 | 1.954 | 0.062 | 1.090 | 1.176 | 1.257 | 1.332 | 1.954 | 1.648 | 0.061 | 1.080 | 1.161 | 1.237 | 1.311 | 1.383 |
| 10.8 | 1.942 | 0.062 | 1.091 | 1.177 | 1.257 | 1.332 | 1.942 | 1.631 | 0.061 | 1.083 | 1.163 | 1.240 | 1.313 | 1.385 |
| 10.9 | 1.930 | 0.062 | 1.091 | 1.177 | 1.257 | 1.333 | 1.930 | 1.613 | 0.061 | 1.085 | 1.165 | 1.242 | 1.316 | 1.387 |
| 11.0 | 1.918 | 0.062 | 1.092 | 1.177 | 1.257 | 1.333 | 1.918 | 1.596 | 0.060 | 1.088 | 1.168 | 1.244 | 1.318 | 1.389 |
| 11.1 | 1.906 | 0.062 | 1.092 | 1.177 | 1.257 | 1.333 | 1.906 | 1.579 | 0.060 | 1.091 | 1.170 | 1.247 | 1.320 | 1.392 |
| 11.2 | 1.894 | 0.062 | 1.093 | 1.178 | 1.257 | 1.333 | 1.894 | 1.562 | 0.060 | 1.094 | 1.173 | 1.250 | 1.323 | 1.395 |
| 11.3 | 1.882 | 0.061 | 1.094 | 1.178 | 1.258 | 1.333 | 1.882 | 1.545 | 0.060 | 1.097 | 1.176 | 1.252 | 1.326 | 1.398 |
| 11.4 | 1.871 | 0.061 | 1.094 | 1.179 | 1.258 | 1.333 | 1.871 | 1.528 | 0.060 | 1.101 | 1.180 | 1.256 | 1.329 | 1.401 |
| 11.5 | 1.859 | 0.061 | 1.095 | 1.179 | 1.258 | 1.333 | 1.859 | 1.511 | 0.059 | 1.104 | 1.183 | 1.259 | 1.333 | 1.404 |
| 11.6 | 1.847 | 0.061 | 1.096 | 1.180 | 1.259 | 1.334 | 1.847 | 1.494 | 0.059 | 1.108 | 1.187 | 1.262 | 1.336 | 1.408 |
| 11.7 | 1.835 | 0.061 | 1.097 | 1.180 | 1.259 | 1.334 | 1.835 | 1.478 | 0.059 | 1.112 | 1.190 | 1.266 | 1.340 | 1.411 |
| 11.8 | 1.823 | 0.061 | 1.098 | 1.181 | 1.260 | 1.335 | 1.823 | 1.461 | 0.059 | 1.116 | 1.194 | 1.270 | 1.343 | 1.415 |
| 11.9 | 1.811 | 0.061 | 1.099 | 1.182 | 1.260 | 1.335 | 1.811 | 1.445 | 0.059 | 1.120 | 1.198 | 1.273 | 1.347 | 1.419 |
| 12.0 | 1.799 | 0.061 | 1.100 | 1.183 | 1.261 | 1.336 | 1.799 | 1.428 | 0.058 | 1.124 | 1.202 | 1.277 | 1.351 | 1.423 |
| 12.1 | 1.787 | 0.060 | 1.101 | 1.184 | 1.262 | 1.336 | 1.787 | 1.412 | 0.058 | 1.128 | 1.206 | 1.281 | 1.355 | 1.427 |
| 12.2 | 1.775 | 0.060 | 1.103 | 1.185 | 1.263 | 1.337 | 1.775 | 1.396 | 0.058 | 1.132 | 1.210 | 1.285 | 1.359 | 1.431 |
| 12.3 | 1.763 | 0.060 | 1.104 | 1.186 | 1.264 | 1.338 | 1.763 | 1.380 | 0.058 | 1.137 | 1.214 | 1.289 | 1.363 | 1.436 |
| 12.4 | 1.751 | 0.060 | 1.106 | 1.188 | 1.265 | 1.339 | 1.751 | 1.364 | 0.058 | 1.141 | 1.218 | 1.294 | 1.367 | 1.440 |
| 12.5 | 1.739 | 0.060 | 1.108 | 1.189 | 1.267 | 1.341 | 1.739 | 1.349 | 0.058 | 1.145 | 1.222 | 1.298 | 1.372 | 1.444 |
| 12.6 | 1.727 | 0.060 | 1.110 | 1.191 | 1.268 | 1.342 | 1.727 | 1.333 | 0.057 | 1.150 | 1.227 | 1.302 | 1.376 | 1.448 |
| 12.7 | 1.716 | 0.059 | 1.112 | 1.193 | 1.270 | 1.344 | 1.716 | 1.318 | 0.057 | 1.154 | 1.231 | 1.306 | 1.380 | 1.453 |
| 12.8 | 1.704 | 0.059 | 1.114 | 1.195 | 1.272 | 1.346 | 1.704 | 1.303 | 0.057 | 1.158 | 1.235 | 1.310 | 1.384 | 1.457 |
| 12.9 | 1.692 | 0.059 | 1.117 | 1.197 | 1.274 | 1.348 | 1.692 | 1.287 | 0.057 | 1.162 | 1.239 | 1.314 | 1.388 | 1.461 |
| 13.0 | 1.680 | 0.059 | 1.119 | 1.200 | 1.276 | 1.350 | 1.680 | 1.273 | 0.057 | 1.166 | 1.243 | 1.318 | 1.392 | 1.465 |
| 13.1 | 1.668 | 0.059 | 1.122 | 1.202 | 1.279 | 1.352 | 1.668 | 1.258 | 0.056 | 1.171 | 1.247 | 1.322 | 1.396 | 1.469 |
| 13.2 | 1.656 | 0.058 | 1.125 | 1.205 | 1.281 | 1.354 | 1.656 | 1.243 | 0.056 | 1.175 | 1.251 | 1.326 | 1.400 | 1.473 |
| 13.3 | 1.644 | 0.058 | 1.128 | 1.208 | 1.284 | 1.357 | 1.644 | 1.229 | 0.056 | 1.179 | 1.255 | 1.330 | 1.404 | 1.477 |
| 13.4 | 1.632 | 0.058 | 1.132 | 1.211 | 1.287 | 1.360 | 1.632 | 1.215 | 0.056 | 1.182 | 1.258 | 1.333 | 1.407 | 1.481 |
| 13.5 | 1.620 | 0.058 | 1.135 | 1.214 | 1.290 | 1.363 | 1.620 | 1.200 | 0.056 | 1.186 | 1.262 | 1.337 | 1.411 | 1.484 |
| 13.6 | 1.608 | 0.057 | 1.139 | 1.217 | 1.293 | 1.366 | 1.608 | 1.187 | 0.056 | 1.190 | 1.265 | 1.340 | 1.414 | 1.488 |
| 13.7 | 1.596 | 0.057 | 1.142 | 1.221 | 1.296 | 1.369 | 1.596 | 1.173 | 0.055 | 1.193 | 1.269 | 1.344 | 1.418 | 1.491 |
| 13.8 | 1.584 | 0.057 | 1.146 | 1.224 | 1.299 | 1.372 | 1.584 | 1.159 | 0.055 | 1.197 | 1.272 | 1.347 | 1.421 | 1.494 |
| 13.9 | 1.572 | 0.057 | 1.150 | 1.228 | 1.303 | 1.375 | 1.572 | 1.146 | 0.055 | 1.200 | 1.275 | 1.350 | 1.424 | 1.497 |
| 14.0 | 1.561 | 0.056 | 1.154 | 1.231 | 1.306 | 1.379 | 1.561 | 1.133 | 0.055 | 1.203 | 1.278 | 1.353 | 1.427 | 1.500 |
| 14.1 | 1.549 | 0.056 | 1.158 | 1.235 | 1.310 | 1.382 | 1.549 | 1.120 | 0.055 | 1.206 | 1.281 | 1.355 | 1.429 | 1.503 |
| 14.2 | 1.537 | 0.056 | 1.162 | 1.239 | 1.313 | 1.386 | 1.537 | 1.107 | 0.054 | 1.209 | 1.284 | 1.358 | 1.432 | 1.505 |
| 14.3 | 1.525 | 0.056 | 1.166 | 1.243 | 1.317 | 1.389 | 1.525 | 1.094 | 0.054 | 1.212 | 1.287 | 1.361 | 1.434 | 1.508 |
| 14.4 | 1.513 | 0.055 | 1.170 | 1.247 | 1.321 | 1.393 | 1.513 | 1.082 | 0.054 | 1.215 | 1.289 | 1.363 | 1.437 | 1.510 |
| 14.5 | 1.501 | 0.055 | 1.174 | 1.251 | 1.324 | 1.396 | 1.501 | 1.070 | 0.054 | 1.217 | 1.292 | 1.365 | 1.439 | 1.512 |
| 14.6 | 1.489 | 0.055 | 1.179 | 1.254 | 1.328 | 1.400 | 1.489 | 1.058 | 0.054 | 1.220 | 1.294 | 1.368 | 1.441 | 1.514 |
| 14.7 | 1.477 | 0.054 | 1.183 | 1.258 | 1.332 | 1.403 | 1.477 | 1.046 | 0.054 | 1.222 | 1.296 | 1.370 | 1.443 | 1.516 |
| 14.8 | 1.465 | 0.054 | 1.187 | 1.262 | 1.335 | 1.406 | 1.465 | 1.034 | 0.053 | 1.225 | 1.298 | 1.372 | 1.445 | 1.518 |
| 14.9 | 1.453 | 0.054 | 1.191 | 1.266 | 1.339 | 1.410 | 1.453 | 1.023 | 0.053 | 1.227 | 1.300 | 1.373 | 1.447 | 1.520 |
| 15.0 | 1.441 | 0.053 | 1.195 | 1.270 | 1.342 | 1.413 | 1.441 | 1.011 | 0.053 | 1.229 | 1.302 | 1.375 | 1.448 | 1.521 |
| 15.1 | 1.429 | 0.053 | 1.200 | 1.274 | 1.346 | 1.417 | 1.429 | 1.000 | 0.053 | 1.231 | 1.304 | 1.377 | 1.450 | 1.523 |
| 15.2 | 1.417 | 0.053 | 1.204 | 1.278 | 1.350 | 1.420 | 1.417 | 0.990 | 0.053 | 1.233 | 1.306 | 1.378 | 1.451 | 1.524 |
| 15.3 | 1.406 | 0.052 | 1.208 | 1.281 | 1.353 | 1.423 | 1.406 | 0.979 | 0.053 | 1.235 | 1.307 | 1.380 | 1.453 | 1.525 |
| 15.4 | 1.394 | 0.052 | 1.212 | 1.285 | 1.356 | 1.426 | 1.394 | 0.969 | 0.052 | 1.237 | 1.309 | 1.381 | 1.454 | 1.526 |
| 15.5 | 1.382 | 0.052 | 1.216 | 1.289 | 1.360 | 1.430 | 1.382 | 0.958 | 0.052 | 1.238 | 1.310 | 1.383 | 1.455 | 1.527 |
| 15.6 | 1.370 | 0.052 | 1.220 | 1.292 | 1.363 | 1.433 | 1.370 | 0.948 | 0.052 | 1.240 | 1.312 | 1.384 | 1.456 | 1.528 |
| 15.7 | 1.358 | 0.051 | 1.223 | 1.296 | 1.366 | 1.436 | 1.358 | 0.938 | 0.052 | 1.242 | 1.313 | 1.385 | 1.457 | 1.529 |
| 15.8 | 1.346 | 0.051 | 1.227 | 1.299 | 1.369 | 1.439 | 1.346 | 0.929 | 0.052 | 1.243 | 1.314 | 1.386 | 1.458 | 1.530 |
| 15.9 | 1.334 | 0.051 | 1.231 | 1.302 | 1.372 | 1.441 | 1.334 | 0.920 | 0.052 | 1.244 | 1.315 | 1.387 | 1.459 | 1.530 |
| 16.0 | 1.322 | 0.050 | 1.234 | 1.305 | 1.375 | 1.444 | 1.322 | 0.910 | 0.051 | 1.246 | 1.317 | 1.388 | 1.459 | 1.531 |
| 16.1 | 1.310 | 0.050 | 1.238 | 1.308 | 1.378 | 1.447 | 1.310 | 0.901 | 0.051 | 1.247 | 1.318 | 1.389 | 1.460 | 1.532 |
| 16.2 | 1.298 | 0.050 | 1.241 | 1.311 | 1.381 | 1.449 | 1.298 | 0.893 | 0.051 | 1.248 | 1.319 | 1.389 | 1.461 | 1.532 |
| 16.3 | 1.286 | 0.050 | 1.244 | 1.314 | 1.383 | 1.452 | 1.286 | 0.884 | 0.051 | 1.249 | 1.320 | 1.390 | 1.461 | 1.533 |
| 16.4 | 1.274 | 0.049 | 1.247 | 1.317 | 1.386 | 1.454 | 1.274 | 0.876 | 0.051 | 1.250 | 1.320 | 1.391 | 1.462 | 1.533 |
| 16.5 | 1.263 | 0.049 | 1.250 | 1.320 | 1.388 | 1.456 | 1.263 | 0.867 | 0.051 | 1.252 | 1.321 | 1.391 | 1.462 | 1.533 |
| 16.6 | 1.251 | 0.049 | 1.253 | 1.322 | 1.391 | 1.458 | 1.251 | 0.859 | 0.050 | 1.253 | 1.322 | 1.392 | 1.463 | 1.534 |
| 16.7 | 1.239 | 0.049 | 1.256 | 1.325 | 1.393 | 1.460 | 1.239 | 0.852 | 0.050 | 1.254 | 1.323 | 1.393 | 1.463 | 1.534 |
| 16.8 | 1.227 | 0.048 | 1.258 | 1.327 | 1.395 | 1.462 | 1.227 | 0.844 | 0.050 | 1.255 | 1.324 | 1.393 | 1.464 | 1.534 |
| 16.9 | 1.215 | 0.048 | 1.261 | 1.329 | 1.397 | 1.464 | 1.215 | 0.837 | 0.050 | 1.256 | 1.325 | 1.394 | 1.464 | 1.535 |
| 17.0 | 1.203 | 0.048 | 1.263 | 1.331 | 1.399 | 1.466 | 1.203 | 0.829 | 0.050 | 1.257 | 1.326 | 1.395 | 1.464 | 1.535 |
| 17.1 | 1.191 | 0.048 | 1.265 | 1.333 | 1.401 | 1.467 | 1.191 | 0.822 | 0.050 | 1.258 | 1.326 | 1.395 | 1.465 | 1.535 |
| 17.2 | 1.179 | 0.048 | 1.267 | 1.335 | 1.402 | 1.469 | 1.179 | 0.815 | 0.049 | 1.259 | 1.327 | 1.396 | 1.465 | 1.535 |
| 17.3 | 1.167 | 0.048 | 1.269 | 1.337 | 1.404 | 1.470 | 1.167 | 0.809 | 0.049 | 1.260 | 1.328 | 1.397 | 1.466 | 1.536 |
| 17.4 | 1.155 | 0.047 | 1.271 | 1.338 | 1.405 | 1.472 | 1.155 | 0.802 | 0.049 | 1.261 | 1.329 | 1.397 | 1.466 | 1.536 |
| 17.5 | 1.143 | 0.047 | 1.273 | 1.340 | 1.407 | 1.473 | 1.143 | 0.796 | 0.049 | 1.262 | 1.330 | 1.398 | 1.467 | 1.536 |
| 17.6 | 1.131 | 0.047 | 1.274 | 1.341 | 1.408 | 1.474 | 1.131 | 0.789 | 0.049 | 1.263 | 1.331 | 1.399 | 1.467 | 1.537 |
| 17.7 | 1.119 | 0.047 | 1.276 | 1.343 | 1.409 | 1.475 | 1.119 | 0.783 | 0.049 | 1.264 | 1.332 | 1.399 | 1.468 | 1.537 |
| 17.8 | 1.108 | 0.047 | 1.277 | 1.344 | 1.410 | 1.476 | 1.108 | 0.777 | 0.049 | 1.266 | 1.332 | 1.400 | 1.468 | 1.537 |
| 17.9 | 1.096 | 0.047 | 1.279 | 1.345 | 1.411 | 1.477 | 1.096 | 0.772 | 0.048 | 1.267 | 1.333 | 1.401 | 1.469 | 1.538 |
| 18.0 | 1.084 | 0.047 | 1.280 | 1.346 | 1.412 | 1.478 | 1.084 | 0.766 | 0.048 | 1.268 | 1.334 | 1.401 | 1.469 | 1.538 |
| 18.1 | 1.072 | 0.047 | 1.281 | 1.347 | 1.413 | 1.479 | 1.072 | 0.760 | 0.048 | 1.269 | 1.335 | 1.402 | 1.470 | 1.538 |
| 18.2 | 1.060 | 0.047 | 1.282 | 1.348 | 1.414 | 1.480 | 1.060 | 0.755 | 0.048 | 1.270 | 1.336 | 1.402 | 1.470 | 1.538 |
| 18.3 | 1.048 | 0.046 | 1.283 | 1.349 | 1.415 | 1.481 | 1.048 | 0.750 | 0.048 | 1.270 | 1.336 | 1.403 | 1.470 | 1.539 |
| 18.4 | 1.036 | 0.046 | 1.284 | 1.350 | 1.416 | 1.482 | 1.036 | 0.745 | 0.048 | 1.271 | 1.337 | 1.403 | 1.471 | 1.539 |
| 18.5 | 1.024 | 0.046 | 1.285 | 1.351 | 1.417 | 1.482 | 1.024 | 0.740 | 0.047 | 1.272 | 1.338 | 1.404 | 1.471 | 1.539 |
| 18.6 | 1.012 | 0.046 | 1.286 | 1.352 | 1.417 | 1.483 | 1.012 | 0.735 | 0.047 | 1.273 | 1.338 | 1.404 | 1.471 | 1.539 |
| 18.7 | 1.000 | 0.046 | 1.287 | 1.352 | 1.418 | 1.484 | 1.000 | 0.730 | 0.047 | 1.274 | 1.339 | 1.405 | 1.471 | 1.539 |
| 18.8 | 0.988 | 0.046 | 1.288 | 1.353 | 1.419 | 1.485 | 0.988 | 0.725 | 0.047 | 1.275 | 1.339 | 1.405 | 1.471 | 1.539 |
| 18.9 | 0.976 | 0.046 | 1.288 | 1.354 | 1.420 | 1.485 | 0.976 | 0.720 | 0.047 | 1.275 | 1.340 | 1.405 | 1.472 | 1.539 |
| 19.0 | 0.964 | 0.046 | 1.289 | 1.355 | 1.420 | 1.486 | 0.964 | 0.716 | 0.047 | 1.276 | 1.340 | 1.405 | 1.472 | 1.539 |
| 19.1 | 0.953 | 0.046 | 1.290 | 1.355 | 1.421 | 1.487 | 0.953 | 0.711 | 0.047 | 1.277 | 1.341 | 1.406 | 1.472 | 1.538 |
| 19.2 | 0.941 | 0.046 | 1.291 | 1.356 | 1.422 | 1.487 | 0.941 | 0.707 | 0.046 | 1.277 | 1.341 | 1.406 | 1.472 | 1.538 |
| 19.3 | 0.929 | 0.046 | 1.291 | 1.357 | 1.422 | 1.488 | 0.929 | 0.703 | 0.046 | 1.278 | 1.341 | 1.406 | 1.471 | 1.538 |
| 19.4 | 0.917 | 0.046 | 1.292 | 1.357 | 1.423 | 1.489 | 0.917 | 0.698 | 0.046 | 1.278 | 1.342 | 1.406 | 1.471 | 1.538 |
| 19.5 | 0.905 | 0.046 | 1.293 | 1.358 | 1.423 | 1.489 | 0.905 | 0.694 | 0.046 | 1.279 | 1.342 | 1.406 | 1.471 | 1.537 |
| 19.6 | 0.893 | 0.046 | 1.293 | 1.358 | 1.424 | 1.490 | 0.893 | 0.690 | 0.046 | 1.279 | 1.342 | 1.406 | 1.471 | 1.537 |
| 19.7 | 0.881 | 0.046 | 1.294 | 1.359 | 1.425 | 1.490 | 0.881 | 0.685 | 0.046 | 1.280 | 1.342 | 1.406 | 1.471 | 1.537 |
| 19.8 | 0.869 | 0.046 | 1.294 | 1.360 | 1.425 | 1.491 | 0.869 | 0.681 | 0.046 | 1.280 | 1.343 | 1.406 | 1.471 | 1.536 |
| 19.9 | 0.857 | 0.046 | 1.295 | 1.360 | 1.426 | 1.492 | 0.857 | 0.677 | 0.045 | 1.280 | 1.343 | 1.406 | 1.471 | 1.536 |
| 20.0 | 0.845 | 0.046 | 1.295 | 1.361 | 1.426 | 1.492 | 0.845 | 0.673 | 0.045 | 1.281 | 1.343 | 1.406 | 1.470 | 1.535 |

| L2(L3)L4TBS | | | | | | | | | | | | | | |
| --- | --- | --- | --- | --- | --- | --- | --- | --- | --- | --- | --- | --- | --- | --- |
|  | Males | | | | | | | Females | | | | | | |
| Age | L | S | -2 SD | -1 SD | 0 SD (M) | 1 SD | 2 SD | L | S | -2 SD | -1 SD | 0 SD (M) | 1 SD | 2 SD |
| 5.0 | 4.166 | 0.058 | 1.103 | 1.210 | 1.293 | 1.362 | 1.421 | 2.103 | 0.061 | 1.094 | 1.179 | 1.259 | 1.333 | 1.403 |
| 5.1 | 4.112 | 0.058 | 1.104 | 1.210 | 1.293 | 1.362 | 1.422 | 2.105 | 0.061 | 1.093 | 1.179 | 1.259 | 1.333 | 1.404 |
| 5.2 | 4.059 | 0.058 | 1.105 | 1.210 | 1.293 | 1.363 | 1.423 | 2.106 | 0.061 | 1.093 | 1.179 | 1.259 | 1.334 | 1.404 |
| 5.3 | 4.006 | 0.058 | 1.105 | 1.210 | 1.294 | 1.363 | 1.424 | 2.108 | 0.061 | 1.093 | 1.179 | 1.259 | 1.334 | 1.404 |
| 5.4 | 3.953 | 0.058 | 1.106 | 1.211 | 1.294 | 1.364 | 1.424 | 2.110 | 0.061 | 1.093 | 1.179 | 1.259 | 1.334 | 1.405 |
| 5.5 | 3.900 | 0.059 | 1.107 | 1.211 | 1.294 | 1.364 | 1.425 | 2.112 | 0.061 | 1.093 | 1.179 | 1.260 | 1.335 | 1.405 |
| 5.6 | 3.848 | 0.059 | 1.107 | 1.211 | 1.295 | 1.365 | 1.426 | 2.113 | 0.062 | 1.092 | 1.179 | 1.260 | 1.335 | 1.405 |
| 5.7 | 3.795 | 0.059 | 1.108 | 1.211 | 1.295 | 1.365 | 1.427 | 2.115 | 0.062 | 1.092 | 1.179 | 1.260 | 1.335 | 1.406 |
| 5.8 | 3.743 | 0.059 | 1.109 | 1.212 | 1.295 | 1.366 | 1.428 | 2.117 | 0.062 | 1.092 | 1.179 | 1.260 | 1.335 | 1.406 |
| 5.9 | 3.691 | 0.059 | 1.109 | 1.212 | 1.295 | 1.366 | 1.429 | 2.118 | 0.062 | 1.092 | 1.179 | 1.260 | 1.335 | 1.406 |
| 6.0 | 3.639 | 0.059 | 1.110 | 1.212 | 1.296 | 1.367 | 1.430 | 2.120 | 0.062 | 1.092 | 1.179 | 1.260 | 1.336 | 1.406 |
| 6.1 | 3.587 | 0.059 | 1.111 | 1.213 | 1.296 | 1.368 | 1.430 | 2.122 | 0.062 | 1.092 | 1.179 | 1.260 | 1.336 | 1.407 |
| 6.2 | 3.536 | 0.059 | 1.111 | 1.213 | 1.296 | 1.368 | 1.431 | 2.123 | 0.062 | 1.091 | 1.179 | 1.260 | 1.336 | 1.407 |
| 6.3 | 3.485 | 0.059 | 1.112 | 1.213 | 1.297 | 1.369 | 1.432 | 2.125 | 0.062 | 1.091 | 1.179 | 1.260 | 1.336 | 1.407 |
| 6.4 | 3.434 | 0.060 | 1.113 | 1.213 | 1.297 | 1.369 | 1.433 | 2.127 | 0.062 | 1.091 | 1.179 | 1.260 | 1.336 | 1.407 |
| 6.5 | 3.384 | 0.060 | 1.113 | 1.214 | 1.297 | 1.370 | 1.434 | 2.129 | 0.062 | 1.091 | 1.179 | 1.260 | 1.336 | 1.407 |
| 6.6 | 3.334 | 0.060 | 1.114 | 1.214 | 1.298 | 1.370 | 1.435 | 2.130 | 0.062 | 1.090 | 1.179 | 1.260 | 1.336 | 1.407 |
| 6.7 | 3.284 | 0.060 | 1.115 | 1.214 | 1.298 | 1.371 | 1.436 | 2.132 | 0.062 | 1.090 | 1.179 | 1.260 | 1.336 | 1.407 |
| 6.8 | 3.235 | 0.060 | 1.116 | 1.215 | 1.298 | 1.371 | 1.437 | 2.134 | 0.062 | 1.090 | 1.179 | 1.260 | 1.336 | 1.407 |
| 6.9 | 3.186 | 0.060 | 1.116 | 1.215 | 1.299 | 1.372 | 1.438 | 2.135 | 0.062 | 1.090 | 1.178 | 1.260 | 1.336 | 1.407 |
| 7.0 | 3.138 | 0.060 | 1.117 | 1.215 | 1.299 | 1.372 | 1.438 | 2.137 | 0.062 | 1.090 | 1.178 | 1.260 | 1.336 | 1.407 |
| 7.1 | 3.091 | 0.060 | 1.118 | 1.216 | 1.299 | 1.373 | 1.439 | 2.139 | 0.062 | 1.089 | 1.178 | 1.260 | 1.336 | 1.407 |
| 7.2 | 3.044 | 0.060 | 1.118 | 1.216 | 1.300 | 1.373 | 1.440 | 2.140 | 0.062 | 1.089 | 1.178 | 1.260 | 1.336 | 1.407 |
| 7.3 | 2.998 | 0.060 | 1.119 | 1.216 | 1.300 | 1.374 | 1.441 | 2.142 | 0.062 | 1.089 | 1.178 | 1.260 | 1.336 | 1.407 |
| 7.4 | 2.952 | 0.060 | 1.120 | 1.217 | 1.300 | 1.374 | 1.441 | 2.144 | 0.062 | 1.089 | 1.178 | 1.260 | 1.336 | 1.407 |
| 7.5 | 2.907 | 0.060 | 1.121 | 1.217 | 1.300 | 1.375 | 1.442 | 2.146 | 0.062 | 1.089 | 1.178 | 1.259 | 1.335 | 1.407 |
| 7.6 | 2.862 | 0.060 | 1.121 | 1.217 | 1.301 | 1.375 | 1.443 | 2.147 | 0.062 | 1.089 | 1.178 | 1.259 | 1.335 | 1.407 |
| 7.7 | 2.818 | 0.060 | 1.122 | 1.217 | 1.301 | 1.375 | 1.443 | 2.149 | 0.062 | 1.089 | 1.178 | 1.259 | 1.335 | 1.407 |
| 7.8 | 2.775 | 0.060 | 1.123 | 1.218 | 1.301 | 1.376 | 1.444 | 2.151 | 0.062 | 1.089 | 1.178 | 1.259 | 1.335 | 1.407 |
| 7.9 | 2.733 | 0.060 | 1.124 | 1.218 | 1.301 | 1.376 | 1.444 | 2.152 | 0.062 | 1.089 | 1.178 | 1.259 | 1.335 | 1.407 |
| 8.0 | 2.691 | 0.060 | 1.124 | 1.218 | 1.301 | 1.376 | 1.445 | 2.154 | 0.062 | 1.090 | 1.178 | 1.260 | 1.335 | 1.406 |
| 8.1 | 2.649 | 0.060 | 1.125 | 1.218 | 1.301 | 1.376 | 1.445 | 2.156 | 0.062 | 1.090 | 1.178 | 1.260 | 1.335 | 1.406 |
| 8.2 | 2.609 | 0.060 | 1.126 | 1.219 | 1.301 | 1.376 | 1.445 | 2.157 | 0.062 | 1.090 | 1.178 | 1.260 | 1.335 | 1.406 |
| 8.3 | 2.569 | 0.060 | 1.127 | 1.219 | 1.301 | 1.376 | 1.445 | 2.159 | 0.062 | 1.090 | 1.179 | 1.260 | 1.336 | 1.406 |
| 8.4 | 2.530 | 0.060 | 1.127 | 1.219 | 1.301 | 1.376 | 1.445 | 2.161 | 0.062 | 1.091 | 1.179 | 1.260 | 1.336 | 1.407 |
| 8.5 | 2.491 | 0.060 | 1.128 | 1.219 | 1.301 | 1.376 | 1.445 | 2.163 | 0.062 | 1.091 | 1.179 | 1.260 | 1.336 | 1.407 |
| 8.6 | 2.453 | 0.060 | 1.129 | 1.219 | 1.301 | 1.376 | 1.446 | 2.164 | 0.062 | 1.092 | 1.180 | 1.261 | 1.336 | 1.407 |
| 8.7 | 2.416 | 0.060 | 1.129 | 1.219 | 1.301 | 1.376 | 1.446 | 2.166 | 0.062 | 1.092 | 1.180 | 1.261 | 1.336 | 1.407 |
| 8.8 | 2.379 | 0.060 | 1.130 | 1.219 | 1.301 | 1.376 | 1.446 | 2.168 | 0.062 | 1.093 | 1.181 | 1.261 | 1.336 | 1.407 |
| 8.9 | 2.343 | 0.060 | 1.130 | 1.220 | 1.301 | 1.376 | 1.445 | 2.169 | 0.062 | 1.093 | 1.181 | 1.262 | 1.337 | 1.407 |
| 9.0 | 2.307 | 0.060 | 1.131 | 1.220 | 1.301 | 1.375 | 1.445 | 2.171 | 0.061 | 1.094 | 1.182 | 1.262 | 1.337 | 1.407 |
| 9.1 | 2.272 | 0.060 | 1.131 | 1.220 | 1.300 | 1.375 | 1.445 | 2.173 | 0.061 | 1.095 | 1.182 | 1.263 | 1.337 | 1.408 |
| 9.2 | 2.237 | 0.060 | 1.132 | 1.220 | 1.300 | 1.375 | 1.445 | 2.174 | 0.061 | 1.096 | 1.183 | 1.263 | 1.338 | 1.408 |
| 9.3 | 2.203 | 0.060 | 1.132 | 1.220 | 1.300 | 1.375 | 1.445 | 2.176 | 0.061 | 1.097 | 1.184 | 1.264 | 1.338 | 1.408 |
| 9.4 | 2.170 | 0.059 | 1.133 | 1.220 | 1.300 | 1.375 | 1.445 | 2.178 | 0.061 | 1.098 | 1.184 | 1.264 | 1.339 | 1.409 |
| 9.5 | 2.137 | 0.059 | 1.133 | 1.220 | 1.300 | 1.374 | 1.445 | 2.180 | 0.061 | 1.099 | 1.185 | 1.265 | 1.339 | 1.409 |
| 9.6 | 2.104 | 0.059 | 1.134 | 1.220 | 1.299 | 1.374 | 1.445 | 2.181 | 0.061 | 1.100 | 1.186 | 1.266 | 1.340 | 1.409 |
| 9.7 | 2.072 | 0.059 | 1.134 | 1.220 | 1.299 | 1.374 | 1.444 | 2.183 | 0.060 | 1.101 | 1.187 | 1.267 | 1.341 | 1.410 |
| 9.8 | 2.041 | 0.059 | 1.134 | 1.220 | 1.299 | 1.374 | 1.444 | 2.185 | 0.060 | 1.102 | 1.188 | 1.268 | 1.341 | 1.411 |
| 9.9 | 2.010 | 0.059 | 1.135 | 1.220 | 1.299 | 1.373 | 1.444 | 2.186 | 0.060 | 1.104 | 1.190 | 1.269 | 1.342 | 1.411 |
| 10.0 | 1.979 | 0.059 | 1.135 | 1.220 | 1.299 | 1.373 | 1.444 | 2.188 | 0.060 | 1.105 | 1.191 | 1.270 | 1.343 | 1.412 |
| 10.1 | 1.949 | 0.059 | 1.136 | 1.220 | 1.298 | 1.373 | 1.444 | 2.190 | 0.060 | 1.107 | 1.192 | 1.271 | 1.344 | 1.413 |
| 10.2 | 1.919 | 0.059 | 1.136 | 1.220 | 1.298 | 1.373 | 1.444 | 2.191 | 0.059 | 1.109 | 1.194 | 1.272 | 1.345 | 1.414 |
| 10.3 | 1.890 | 0.059 | 1.136 | 1.220 | 1.298 | 1.373 | 1.444 | 2.193 | 0.059 | 1.111 | 1.195 | 1.274 | 1.347 | 1.415 |
| 10.4 | 1.861 | 0.059 | 1.137 | 1.220 | 1.298 | 1.373 | 1.444 | 2.195 | 0.059 | 1.113 | 1.197 | 1.275 | 1.348 | 1.416 |
| 10.5 | 1.832 | 0.059 | 1.137 | 1.220 | 1.298 | 1.373 | 1.444 | 2.197 | 0.059 | 1.115 | 1.199 | 1.277 | 1.350 | 1.418 |
| 10.6 | 1.804 | 0.059 | 1.138 | 1.220 | 1.298 | 1.373 | 1.444 | 2.198 | 0.059 | 1.117 | 1.201 | 1.279 | 1.351 | 1.419 |
| 10.7 | 1.776 | 0.059 | 1.138 | 1.220 | 1.298 | 1.373 | 1.444 | 2.200 | 0.058 | 1.119 | 1.203 | 1.281 | 1.353 | 1.421 |
| 10.8 | 1.749 | 0.059 | 1.139 | 1.220 | 1.298 | 1.373 | 1.444 | 2.202 | 0.058 | 1.122 | 1.206 | 1.283 | 1.355 | 1.423 |
| 10.9 | 1.723 | 0.059 | 1.139 | 1.221 | 1.298 | 1.373 | 1.445 | 2.203 | 0.058 | 1.125 | 1.208 | 1.285 | 1.357 | 1.425 |
| 11.0 | 1.696 | 0.058 | 1.140 | 1.221 | 1.299 | 1.373 | 1.445 | 2.205 | 0.058 | 1.128 | 1.211 | 1.288 | 1.360 | 1.427 |
| 11.1 | 1.670 | 0.058 | 1.141 | 1.221 | 1.299 | 1.373 | 1.445 | 2.207 | 0.057 | 1.131 | 1.214 | 1.291 | 1.362 | 1.430 |
| 11.2 | 1.645 | 0.058 | 1.141 | 1.222 | 1.299 | 1.374 | 1.446 | 2.208 | 0.057 | 1.134 | 1.217 | 1.294 | 1.365 | 1.432 |
| 11.3 | 1.620 | 0.058 | 1.142 | 1.222 | 1.300 | 1.374 | 1.446 | 2.210 | 0.057 | 1.138 | 1.220 | 1.297 | 1.368 | 1.435 |
| 11.4 | 1.596 | 0.058 | 1.143 | 1.223 | 1.300 | 1.375 | 1.447 | 2.212 | 0.057 | 1.141 | 1.224 | 1.300 | 1.371 | 1.438 |
| 11.5 | 1.572 | 0.058 | 1.144 | 1.224 | 1.301 | 1.375 | 1.447 | 2.214 | 0.056 | 1.145 | 1.227 | 1.303 | 1.374 | 1.441 |
| 11.6 | 1.549 | 0.058 | 1.145 | 1.224 | 1.301 | 1.376 | 1.448 | 2.215 | 0.056 | 1.149 | 1.231 | 1.307 | 1.378 | 1.444 |
| 11.7 | 1.527 | 0.058 | 1.146 | 1.225 | 1.302 | 1.376 | 1.449 | 2.217 | 0.056 | 1.153 | 1.235 | 1.310 | 1.381 | 1.448 |
| 11.8 | 1.504 | 0.058 | 1.147 | 1.226 | 1.303 | 1.377 | 1.449 | 2.219 | 0.055 | 1.157 | 1.239 | 1.314 | 1.385 | 1.451 |
| 11.9 | 1.483 | 0.058 | 1.149 | 1.227 | 1.304 | 1.378 | 1.450 | 2.220 | 0.055 | 1.161 | 1.243 | 1.318 | 1.388 | 1.455 |
| 12.0 | 1.462 | 0.058 | 1.150 | 1.229 | 1.305 | 1.379 | 1.451 | 2.222 | 0.055 | 1.166 | 1.247 | 1.322 | 1.392 | 1.458 |
| 12.1 | 1.441 | 0.057 | 1.152 | 1.230 | 1.306 | 1.380 | 1.452 | 2.224 | 0.055 | 1.170 | 1.251 | 1.326 | 1.396 | 1.462 |
| 12.2 | 1.421 | 0.057 | 1.153 | 1.231 | 1.307 | 1.381 | 1.454 | 2.225 | 0.054 | 1.174 | 1.255 | 1.330 | 1.400 | 1.466 |
| 12.3 | 1.401 | 0.057 | 1.155 | 1.233 | 1.309 | 1.383 | 1.455 | 2.227 | 0.054 | 1.179 | 1.260 | 1.334 | 1.404 | 1.470 |
| 12.4 | 1.382 | 0.057 | 1.157 | 1.235 | 1.310 | 1.384 | 1.456 | 2.229 | 0.054 | 1.184 | 1.264 | 1.338 | 1.408 | 1.474 |
| 12.5 | 1.363 | 0.057 | 1.159 | 1.236 | 1.312 | 1.386 | 1.458 | 2.231 | 0.053 | 1.188 | 1.268 | 1.342 | 1.412 | 1.477 |
| 12.6 | 1.345 | 0.057 | 1.162 | 1.239 | 1.314 | 1.387 | 1.460 | 2.232 | 0.053 | 1.193 | 1.272 | 1.347 | 1.416 | 1.481 |
| 12.7 | 1.327 | 0.056 | 1.164 | 1.241 | 1.316 | 1.389 | 1.462 | 2.234 | 0.053 | 1.197 | 1.277 | 1.351 | 1.420 | 1.485 |
| 12.8 | 1.310 | 0.056 | 1.167 | 1.243 | 1.318 | 1.391 | 1.463 | 2.236 | 0.053 | 1.202 | 1.281 | 1.355 | 1.424 | 1.489 |
| 12.9 | 1.293 | 0.056 | 1.170 | 1.246 | 1.320 | 1.393 | 1.465 | 2.237 | 0.052 | 1.206 | 1.285 | 1.359 | 1.428 | 1.492 |
| 13.0 | 1.277 | 0.056 | 1.173 | 1.248 | 1.322 | 1.396 | 1.468 | 2.239 | 0.052 | 1.211 | 1.289 | 1.363 | 1.431 | 1.496 |
| 13.1 | 1.261 | 0.055 | 1.176 | 1.251 | 1.325 | 1.398 | 1.470 | 2.241 | 0.052 | 1.215 | 1.293 | 1.366 | 1.435 | 1.500 |
| 13.2 | 1.245 | 0.055 | 1.179 | 1.254 | 1.328 | 1.400 | 1.472 | 2.242 | 0.051 | 1.219 | 1.297 | 1.370 | 1.438 | 1.503 |
| 13.3 | 1.231 | 0.055 | 1.183 | 1.257 | 1.331 | 1.403 | 1.475 | 2.244 | 0.051 | 1.223 | 1.301 | 1.374 | 1.442 | 1.506 |
| 13.4 | 1.216 | 0.055 | 1.186 | 1.260 | 1.333 | 1.406 | 1.477 | 2.246 | 0.051 | 1.227 | 1.305 | 1.377 | 1.445 | 1.509 |
| 13.5 | 1.202 | 0.054 | 1.190 | 1.264 | 1.337 | 1.409 | 1.480 | 2.248 | 0.051 | 1.231 | 1.309 | 1.381 | 1.449 | 1.513 |
| 13.6 | 1.189 | 0.054 | 1.194 | 1.267 | 1.340 | 1.412 | 1.483 | 2.249 | 0.050 | 1.235 | 1.312 | 1.384 | 1.452 | 1.516 |
| 13.7 | 1.176 | 0.054 | 1.198 | 1.271 | 1.343 | 1.415 | 1.485 | 2.251 | 0.050 | 1.239 | 1.316 | 1.388 | 1.455 | 1.518 |
| 13.8 | 1.164 | 0.053 | 1.202 | 1.274 | 1.346 | 1.418 | 1.488 | 2.253 | 0.050 | 1.243 | 1.319 | 1.391 | 1.458 | 1.521 |
| 13.9 | 1.152 | 0.053 | 1.206 | 1.278 | 1.350 | 1.421 | 1.491 | 2.254 | 0.049 | 1.246 | 1.322 | 1.394 | 1.460 | 1.524 |
| 14.0 | 1.141 | 0.052 | 1.210 | 1.282 | 1.353 | 1.424 | 1.494 | 2.256 | 0.049 | 1.249 | 1.326 | 1.396 | 1.463 | 1.526 |
| 14.1 | 1.130 | 0.052 | 1.214 | 1.286 | 1.357 | 1.427 | 1.497 | 2.258 | 0.049 | 1.253 | 1.328 | 1.399 | 1.466 | 1.528 |
| 14.2 | 1.121 | 0.052 | 1.219 | 1.290 | 1.360 | 1.431 | 1.500 | 2.259 | 0.049 | 1.256 | 1.331 | 1.402 | 1.468 | 1.531 |
| 14.3 | 1.111 | 0.051 | 1.223 | 1.294 | 1.364 | 1.434 | 1.503 | 2.261 | 0.048 | 1.259 | 1.334 | 1.404 | 1.470 | 1.533 |
| 14.4 | 1.103 | 0.051 | 1.228 | 1.298 | 1.368 | 1.437 | 1.506 | 2.263 | 0.048 | 1.262 | 1.337 | 1.407 | 1.472 | 1.535 |
| 14.5 | 1.095 | 0.051 | 1.232 | 1.302 | 1.372 | 1.441 | 1.510 | 2.265 | 0.048 | 1.265 | 1.339 | 1.409 | 1.475 | 1.537 |
| 14.6 | 1.088 | 0.050 | 1.237 | 1.306 | 1.375 | 1.444 | 1.513 | 2.266 | 0.048 | 1.267 | 1.342 | 1.411 | 1.477 | 1.538 |
| 14.7 | 1.082 | 0.050 | 1.241 | 1.310 | 1.379 | 1.447 | 1.516 | 2.268 | 0.047 | 1.270 | 1.344 | 1.413 | 1.478 | 1.540 |
| 14.8 | 1.076 | 0.049 | 1.246 | 1.314 | 1.383 | 1.451 | 1.519 | 2.270 | 0.047 | 1.272 | 1.346 | 1.415 | 1.480 | 1.542 |
| 14.9 | 1.072 | 0.049 | 1.250 | 1.318 | 1.386 | 1.454 | 1.522 | 2.271 | 0.047 | 1.275 | 1.348 | 1.417 | 1.482 | 1.543 |
| 15.0 | 1.068 | 0.049 | 1.255 | 1.322 | 1.390 | 1.457 | 1.525 | 2.273 | 0.047 | 1.277 | 1.350 | 1.419 | 1.484 | 1.545 |
| 15.1 | 1.065 | 0.048 | 1.259 | 1.326 | 1.394 | 1.461 | 1.528 | 2.275 | 0.047 | 1.279 | 1.352 | 1.421 | 1.485 | 1.546 |
| 15.2 | 1.063 | 0.048 | 1.263 | 1.330 | 1.397 | 1.464 | 1.530 | 2.276 | 0.046 | 1.282 | 1.354 | 1.422 | 1.487 | 1.547 |
| 15.3 | 1.061 | 0.047 | 1.267 | 1.334 | 1.401 | 1.467 | 1.533 | 2.278 | 0.046 | 1.284 | 1.356 | 1.424 | 1.488 | 1.549 |
| 15.4 | 1.061 | 0.047 | 1.272 | 1.338 | 1.404 | 1.470 | 1.536 | 2.280 | 0.046 | 1.285 | 1.358 | 1.425 | 1.489 | 1.550 |
| 15.5 | 1.061 | 0.047 | 1.276 | 1.342 | 1.408 | 1.473 | 1.539 | 2.282 | 0.046 | 1.287 | 1.359 | 1.427 | 1.490 | 1.551 |
| 15.6 | 1.063 | 0.046 | 1.280 | 1.345 | 1.411 | 1.476 | 1.541 | 2.283 | 0.046 | 1.289 | 1.361 | 1.428 | 1.492 | 1.552 |
| 15.7 | 1.065 | 0.046 | 1.284 | 1.349 | 1.414 | 1.479 | 1.544 | 2.285 | 0.045 | 1.291 | 1.362 | 1.429 | 1.493 | 1.552 |
| 15.8 | 1.069 | 0.046 | 1.287 | 1.352 | 1.417 | 1.482 | 1.546 | 2.287 | 0.045 | 1.292 | 1.364 | 1.430 | 1.493 | 1.553 |
| 15.9 | 1.073 | 0.045 | 1.291 | 1.356 | 1.420 | 1.485 | 1.549 | 2.288 | 0.045 | 1.294 | 1.365 | 1.432 | 1.494 | 1.554 |
| 16.0 | 1.079 | 0.045 | 1.294 | 1.359 | 1.423 | 1.487 | 1.551 | 2.290 | 0.045 | 1.295 | 1.366 | 1.433 | 1.495 | 1.555 |
| 16.1 | 1.085 | 0.045 | 1.298 | 1.362 | 1.426 | 1.490 | 1.553 | 2.292 | 0.045 | 1.297 | 1.367 | 1.434 | 1.496 | 1.555 |
| 16.2 | 1.093 | 0.044 | 1.301 | 1.365 | 1.429 | 1.492 | 1.555 | 2.293 | 0.045 | 1.298 | 1.369 | 1.434 | 1.497 | 1.556 |
| 16.3 | 1.102 | 0.044 | 1.304 | 1.368 | 1.431 | 1.494 | 1.557 | 2.295 | 0.044 | 1.300 | 1.370 | 1.435 | 1.497 | 1.556 |
| 16.4 | 1.112 | 0.044 | 1.307 | 1.371 | 1.434 | 1.497 | 1.559 | 2.297 | 0.044 | 1.301 | 1.371 | 1.436 | 1.498 | 1.557 |
| 16.5 | 1.123 | 0.044 | 1.310 | 1.373 | 1.436 | 1.499 | 1.561 | 2.299 | 0.044 | 1.302 | 1.372 | 1.437 | 1.498 | 1.557 |
| 16.6 | 1.135 | 0.043 | 1.313 | 1.376 | 1.439 | 1.501 | 1.563 | 2.300 | 0.044 | 1.303 | 1.373 | 1.438 | 1.499 | 1.557 |
| 16.7 | 1.148 | 0.043 | 1.315 | 1.378 | 1.441 | 1.503 | 1.565 | 2.302 | 0.044 | 1.304 | 1.373 | 1.438 | 1.499 | 1.558 |
| 16.8 | 1.162 | 0.043 | 1.318 | 1.380 | 1.443 | 1.505 | 1.566 | 2.304 | 0.044 | 1.305 | 1.374 | 1.439 | 1.500 | 1.558 |
| 16.9 | 1.178 | 0.043 | 1.320 | 1.383 | 1.445 | 1.507 | 1.568 | 2.305 | 0.043 | 1.307 | 1.375 | 1.440 | 1.500 | 1.558 |
| 17.0 | 1.195 | 0.043 | 1.322 | 1.385 | 1.447 | 1.508 | 1.569 | 2.307 | 0.043 | 1.308 | 1.376 | 1.440 | 1.501 | 1.559 |
| 17.1 | 1.212 | 0.043 | 1.324 | 1.387 | 1.449 | 1.510 | 1.571 | 2.309 | 0.043 | 1.309 | 1.377 | 1.441 | 1.502 | 1.559 |
| 17.2 | 1.231 | 0.042 | 1.326 | 1.388 | 1.450 | 1.512 | 1.572 | 2.310 | 0.043 | 1.310 | 1.378 | 1.442 | 1.502 | 1.559 |
| 17.3 | 1.251 | 0.042 | 1.328 | 1.390 | 1.452 | 1.513 | 1.574 | 2.312 | 0.043 | 1.311 | 1.379 | 1.442 | 1.503 | 1.560 |
| 17.4 | 1.272 | 0.042 | 1.329 | 1.392 | 1.454 | 1.515 | 1.575 | 2.314 | 0.043 | 1.312 | 1.380 | 1.443 | 1.503 | 1.560 |
| 17.5 | 1.295 | 0.042 | 1.331 | 1.393 | 1.455 | 1.516 | 1.576 | 2.316 | 0.043 | 1.314 | 1.381 | 1.444 | 1.504 | 1.561 |
| 17.6 | 1.318 | 0.042 | 1.332 | 1.395 | 1.456 | 1.517 | 1.577 | 2.317 | 0.042 | 1.315 | 1.382 | 1.445 | 1.504 | 1.561 |
| 17.7 | 1.342 | 0.042 | 1.334 | 1.396 | 1.458 | 1.518 | 1.578 | 2.319 | 0.042 | 1.316 | 1.383 | 1.446 | 1.505 | 1.561 |
| 17.8 | 1.367 | 0.042 | 1.335 | 1.397 | 1.459 | 1.520 | 1.579 | 2.321 | 0.042 | 1.317 | 1.384 | 1.446 | 1.506 | 1.562 |
| 17.9 | 1.393 | 0.042 | 1.336 | 1.399 | 1.460 | 1.521 | 1.581 | 2.322 | 0.042 | 1.318 | 1.385 | 1.447 | 1.506 | 1.562 |
| 18.0 | 1.419 | 0.042 | 1.337 | 1.400 | 1.461 | 1.522 | 1.582 | 2.324 | 0.042 | 1.319 | 1.386 | 1.448 | 1.507 | 1.563 |
| 18.1 | 1.447 | 0.042 | 1.338 | 1.401 | 1.462 | 1.523 | 1.583 | 2.326 | 0.042 | 1.320 | 1.386 | 1.448 | 1.507 | 1.563 |
| 18.2 | 1.475 | 0.042 | 1.339 | 1.402 | 1.463 | 1.524 | 1.584 | 2.327 | 0.041 | 1.322 | 1.387 | 1.449 | 1.508 | 1.563 |
| 18.3 | 1.504 | 0.042 | 1.339 | 1.403 | 1.464 | 1.525 | 1.584 | 2.329 | 0.041 | 1.323 | 1.388 | 1.450 | 1.508 | 1.564 |
| 18.4 | 1.534 | 0.042 | 1.340 | 1.403 | 1.465 | 1.526 | 1.585 | 2.331 | 0.041 | 1.324 | 1.389 | 1.450 | 1.509 | 1.564 |
| 18.5 | 1.565 | 0.042 | 1.340 | 1.404 | 1.466 | 1.527 | 1.586 | 2.333 | 0.041 | 1.325 | 1.390 | 1.451 | 1.509 | 1.564 |
| 18.6 | 1.596 | 0.042 | 1.341 | 1.405 | 1.467 | 1.528 | 1.587 | 2.334 | 0.041 | 1.326 | 1.391 | 1.452 | 1.509 | 1.564 |
| 18.7 | 1.627 | 0.042 | 1.341 | 1.406 | 1.468 | 1.529 | 1.588 | 2.336 | 0.041 | 1.327 | 1.391 | 1.452 | 1.510 | 1.565 |
| 18.8 | 1.660 | 0.042 | 1.342 | 1.406 | 1.469 | 1.530 | 1.589 | 2.338 | 0.041 | 1.327 | 1.392 | 1.452 | 1.510 | 1.565 |
| 18.9 | 1.692 | 0.042 | 1.342 | 1.407 | 1.469 | 1.530 | 1.590 | 2.339 | 0.040 | 1.328 | 1.392 | 1.453 | 1.510 | 1.565 |
| 19.0 | 1.725 | 0.042 | 1.342 | 1.407 | 1.470 | 1.531 | 1.590 | 2.341 | 0.040 | 1.329 | 1.393 | 1.453 | 1.510 | 1.565 |
| 19.1 | 1.759 | 0.042 | 1.343 | 1.408 | 1.471 | 1.532 | 1.591 | 2.343 | 0.040 | 1.330 | 1.393 | 1.453 | 1.510 | 1.564 |
| 19.2 | 1.793 | 0.042 | 1.343 | 1.408 | 1.472 | 1.533 | 1.592 | 2.344 | 0.040 | 1.330 | 1.394 | 1.454 | 1.510 | 1.564 |
| 19.3 | 1.827 | 0.042 | 1.343 | 1.409 | 1.472 | 1.533 | 1.593 | 2.346 | 0.040 | 1.331 | 1.394 | 1.454 | 1.510 | 1.564 |
| 19.4 | 1.862 | 0.042 | 1.343 | 1.409 | 1.473 | 1.534 | 1.594 | 2.348 | 0.040 | 1.331 | 1.394 | 1.454 | 1.510 | 1.564 |
| 19.5 | 1.897 | 0.043 | 1.343 | 1.409 | 1.473 | 1.535 | 1.594 | 2.350 | 0.040 | 1.332 | 1.395 | 1.454 | 1.510 | 1.563 |
| 19.6 | 1.932 | 0.043 | 1.343 | 1.410 | 1.474 | 1.536 | 1.595 | 2.351 | 0.039 | 1.332 | 1.395 | 1.454 | 1.510 | 1.563 |
| 19.7 | 1.968 | 0.043 | 1.343 | 1.410 | 1.474 | 1.536 | 1.596 | 2.353 | 0.039 | 1.333 | 1.395 | 1.454 | 1.509 | 1.562 |
| 19.8 | 2.004 | 0.043 | 1.343 | 1.410 | 1.475 | 1.537 | 1.596 | 2.355 | 0.039 | 1.333 | 1.395 | 1.453 | 1.509 | 1.562 |
| 19.9 | 2.040 | 0.043 | 1.342 | 1.411 | 1.475 | 1.537 | 1.597 | 2.356 | 0.039 | 1.333 | 1.395 | 1.453 | 1.509 | 1.561 |
| 20.0 | 2.076 | 0.043 | 1.342 | 1.411 | 1.476 | 1.538 | 1.597 | 2.358 | 0.039 | 1.334 | 1.395 | 1.453 | 1.508 | 1.561 |

| L1L2(L3)L4 TBS | | | | | | | | | | | | | | |
| --- | --- | --- | --- | --- | --- | --- | --- | --- | --- | --- | --- | --- | --- | --- |
|  | Males | | | | | | | Females | | | | | | |
| Age | L | S | -2 SD | -1 SD | 0 SD (M) | 1 SD | 2 SD | L | S | -2 SD | -1 SD | 0 SD (M) | 1 SD | 2 SD |
| 5.0 | 3.622 | 0.058 | 1.083 | 1.180 | 1.260 | 1.329 | 1.389 | 2.638 | 0.056 | 1.077 | 1.158 | 1.231 | 1.297 | 1.358 |
| 5.1 | 3.588 | 0.058 | 1.084 | 1.181 | 1.261 | 1.329 | 1.390 | 2.625 | 0.056 | 1.077 | 1.158 | 1.231 | 1.297 | 1.359 |
| 5.2 | 3.555 | 0.058 | 1.084 | 1.181 | 1.261 | 1.330 | 1.391 | 2.613 | 0.057 | 1.076 | 1.158 | 1.231 | 1.298 | 1.359 |
| 5.3 | 3.522 | 0.058 | 1.085 | 1.182 | 1.262 | 1.330 | 1.391 | 2.601 | 0.057 | 1.076 | 1.158 | 1.231 | 1.298 | 1.360 |
| 5.4 | 3.489 | 0.059 | 1.086 | 1.182 | 1.262 | 1.331 | 1.392 | 2.589 | 0.057 | 1.076 | 1.158 | 1.231 | 1.298 | 1.360 |
| 5.5 | 3.456 | 0.059 | 1.086 | 1.182 | 1.262 | 1.331 | 1.393 | 2.577 | 0.057 | 1.076 | 1.157 | 1.231 | 1.298 | 1.360 |
| 5.6 | 3.422 | 0.059 | 1.087 | 1.183 | 1.263 | 1.332 | 1.394 | 2.564 | 0.057 | 1.075 | 1.157 | 1.231 | 1.298 | 1.361 |
| 5.7 | 3.389 | 0.059 | 1.088 | 1.183 | 1.263 | 1.332 | 1.394 | 2.552 | 0.057 | 1.075 | 1.157 | 1.231 | 1.299 | 1.361 |
| 5.8 | 3.356 | 0.059 | 1.088 | 1.183 | 1.263 | 1.333 | 1.395 | 2.540 | 0.057 | 1.075 | 1.157 | 1.231 | 1.299 | 1.361 |
| 5.9 | 3.323 | 0.059 | 1.089 | 1.184 | 1.264 | 1.333 | 1.396 | 2.528 | 0.058 | 1.075 | 1.157 | 1.231 | 1.299 | 1.362 |
| 6.0 | 3.291 | 0.059 | 1.089 | 1.184 | 1.264 | 1.334 | 1.396 | 2.515 | 0.058 | 1.074 | 1.157 | 1.231 | 1.299 | 1.362 |
| 6.1 | 3.258 | 0.059 | 1.090 | 1.184 | 1.264 | 1.334 | 1.397 | 2.503 | 0.058 | 1.074 | 1.156 | 1.231 | 1.299 | 1.362 |
| 6.2 | 3.225 | 0.059 | 1.091 | 1.185 | 1.265 | 1.335 | 1.398 | 2.491 | 0.058 | 1.074 | 1.156 | 1.231 | 1.299 | 1.363 |
| 6.3 | 3.192 | 0.059 | 1.091 | 1.185 | 1.265 | 1.335 | 1.398 | 2.479 | 0.058 | 1.073 | 1.156 | 1.231 | 1.299 | 1.363 |
| 6.4 | 3.160 | 0.059 | 1.092 | 1.185 | 1.265 | 1.336 | 1.399 | 2.466 | 0.058 | 1.073 | 1.156 | 1.231 | 1.300 | 1.363 |
| 6.5 | 3.127 | 0.059 | 1.092 | 1.186 | 1.266 | 1.336 | 1.399 | 2.454 | 0.058 | 1.073 | 1.156 | 1.231 | 1.300 | 1.364 |
| 6.6 | 3.094 | 0.059 | 1.093 | 1.186 | 1.266 | 1.336 | 1.400 | 2.442 | 0.058 | 1.073 | 1.155 | 1.231 | 1.300 | 1.364 |
| 6.7 | 3.062 | 0.059 | 1.093 | 1.186 | 1.266 | 1.337 | 1.401 | 2.429 | 0.059 | 1.072 | 1.155 | 1.230 | 1.300 | 1.364 |
| 6.8 | 3.030 | 0.059 | 1.094 | 1.187 | 1.266 | 1.337 | 1.401 | 2.417 | 0.059 | 1.072 | 1.155 | 1.230 | 1.300 | 1.364 |
| 6.9 | 2.997 | 0.059 | 1.095 | 1.187 | 1.267 | 1.338 | 1.402 | 2.405 | 0.059 | 1.072 | 1.155 | 1.230 | 1.300 | 1.364 |
| 7.0 | 2.965 | 0.059 | 1.095 | 1.187 | 1.267 | 1.338 | 1.402 | 2.392 | 0.059 | 1.071 | 1.155 | 1.230 | 1.300 | 1.364 |
| 7.1 | 2.933 | 0.059 | 1.096 | 1.187 | 1.267 | 1.338 | 1.403 | 2.380 | 0.059 | 1.071 | 1.154 | 1.230 | 1.300 | 1.365 |
| 7.2 | 2.901 | 0.059 | 1.096 | 1.188 | 1.267 | 1.338 | 1.403 | 2.367 | 0.059 | 1.071 | 1.154 | 1.230 | 1.300 | 1.365 |
| 7.3 | 2.869 | 0.059 | 1.097 | 1.188 | 1.267 | 1.339 | 1.403 | 2.355 | 0.059 | 1.071 | 1.154 | 1.230 | 1.300 | 1.365 |
| 7.4 | 2.837 | 0.059 | 1.097 | 1.188 | 1.268 | 1.339 | 1.404 | 2.343 | 0.059 | 1.070 | 1.154 | 1.230 | 1.300 | 1.365 |
| 7.5 | 2.805 | 0.059 | 1.098 | 1.188 | 1.268 | 1.339 | 1.404 | 2.330 | 0.059 | 1.070 | 1.153 | 1.229 | 1.299 | 1.365 |
| 7.6 | 2.773 | 0.059 | 1.098 | 1.189 | 1.268 | 1.339 | 1.404 | 2.318 | 0.059 | 1.070 | 1.153 | 1.229 | 1.299 | 1.365 |
| 7.7 | 2.741 | 0.059 | 1.099 | 1.189 | 1.268 | 1.339 | 1.405 | 2.305 | 0.059 | 1.070 | 1.153 | 1.229 | 1.299 | 1.365 |
| 7.8 | 2.709 | 0.059 | 1.100 | 1.189 | 1.268 | 1.339 | 1.405 | 2.293 | 0.059 | 1.070 | 1.153 | 1.229 | 1.299 | 1.365 |
| 7.9 | 2.678 | 0.059 | 1.100 | 1.189 | 1.268 | 1.339 | 1.405 | 2.280 | 0.059 | 1.070 | 1.153 | 1.229 | 1.299 | 1.365 |
| 8.0 | 2.646 | 0.059 | 1.101 | 1.189 | 1.268 | 1.340 | 1.405 | 2.268 | 0.059 | 1.070 | 1.153 | 1.229 | 1.299 | 1.365 |
| 8.1 | 2.614 | 0.059 | 1.101 | 1.189 | 1.268 | 1.339 | 1.405 | 2.255 | 0.059 | 1.070 | 1.153 | 1.229 | 1.300 | 1.366 |
| 8.2 | 2.583 | 0.059 | 1.102 | 1.189 | 1.268 | 1.339 | 1.405 | 2.242 | 0.059 | 1.070 | 1.153 | 1.229 | 1.300 | 1.366 |
| 8.3 | 2.551 | 0.059 | 1.102 | 1.190 | 1.268 | 1.339 | 1.405 | 2.230 | 0.060 | 1.070 | 1.153 | 1.229 | 1.300 | 1.366 |
| 8.4 | 2.520 | 0.059 | 1.103 | 1.190 | 1.268 | 1.339 | 1.405 | 2.217 | 0.060 | 1.071 | 1.153 | 1.229 | 1.300 | 1.366 |
| 8.5 | 2.488 | 0.059 | 1.103 | 1.190 | 1.268 | 1.339 | 1.405 | 2.205 | 0.060 | 1.071 | 1.153 | 1.229 | 1.300 | 1.366 |
| 8.6 | 2.457 | 0.059 | 1.104 | 1.190 | 1.268 | 1.339 | 1.405 | 2.192 | 0.060 | 1.071 | 1.154 | 1.230 | 1.300 | 1.367 |
| 8.7 | 2.425 | 0.059 | 1.104 | 1.190 | 1.267 | 1.339 | 1.405 | 2.180 | 0.059 | 1.072 | 1.154 | 1.230 | 1.301 | 1.367 |
| 8.8 | 2.394 | 0.059 | 1.105 | 1.190 | 1.267 | 1.339 | 1.405 | 2.168 | 0.059 | 1.072 | 1.154 | 1.230 | 1.301 | 1.368 |
| 8.9 | 2.362 | 0.059 | 1.105 | 1.190 | 1.267 | 1.339 | 1.405 | 2.155 | 0.059 | 1.073 | 1.155 | 1.231 | 1.301 | 1.368 |
| 9.0 | 2.330 | 0.058 | 1.105 | 1.190 | 1.267 | 1.338 | 1.405 | 2.143 | 0.059 | 1.073 | 1.155 | 1.231 | 1.302 | 1.368 |
| 9.1 | 2.299 | 0.058 | 1.106 | 1.190 | 1.267 | 1.338 | 1.405 | 2.130 | 0.059 | 1.074 | 1.156 | 1.231 | 1.302 | 1.369 |
| 9.2 | 2.267 | 0.058 | 1.106 | 1.190 | 1.267 | 1.338 | 1.405 | 2.118 | 0.059 | 1.075 | 1.156 | 1.232 | 1.303 | 1.370 |
| 9.3 | 2.236 | 0.058 | 1.107 | 1.190 | 1.267 | 1.338 | 1.405 | 2.106 | 0.059 | 1.075 | 1.157 | 1.233 | 1.303 | 1.370 |
| 9.4 | 2.204 | 0.058 | 1.107 | 1.190 | 1.266 | 1.338 | 1.405 | 2.093 | 0.059 | 1.076 | 1.158 | 1.233 | 1.304 | 1.371 |
| 9.5 | 2.172 | 0.058 | 1.107 | 1.190 | 1.266 | 1.337 | 1.405 | 2.081 | 0.059 | 1.077 | 1.158 | 1.234 | 1.305 | 1.372 |
| 9.6 | 2.140 | 0.058 | 1.108 | 1.190 | 1.266 | 1.337 | 1.404 | 2.069 | 0.059 | 1.078 | 1.159 | 1.235 | 1.306 | 1.373 |
| 9.7 | 2.109 | 0.058 | 1.108 | 1.190 | 1.266 | 1.337 | 1.404 | 2.057 | 0.059 | 1.079 | 1.160 | 1.236 | 1.307 | 1.374 |
| 9.8 | 2.077 | 0.058 | 1.108 | 1.190 | 1.266 | 1.337 | 1.404 | 2.045 | 0.059 | 1.081 | 1.161 | 1.237 | 1.308 | 1.375 |
| 9.9 | 2.045 | 0.058 | 1.109 | 1.190 | 1.266 | 1.337 | 1.404 | 2.033 | 0.059 | 1.082 | 1.163 | 1.238 | 1.309 | 1.376 |
| 10.0 | 2.013 | 0.058 | 1.109 | 1.190 | 1.265 | 1.337 | 1.404 | 2.021 | 0.059 | 1.083 | 1.164 | 1.239 | 1.310 | 1.377 |
| 10.1 | 1.981 | 0.058 | 1.110 | 1.190 | 1.265 | 1.337 | 1.404 | 2.010 | 0.059 | 1.085 | 1.165 | 1.240 | 1.311 | 1.378 |
| 10.2 | 1.948 | 0.058 | 1.110 | 1.190 | 1.265 | 1.337 | 1.404 | 1.998 | 0.059 | 1.087 | 1.167 | 1.242 | 1.313 | 1.380 |
| 10.3 | 1.916 | 0.058 | 1.110 | 1.190 | 1.265 | 1.337 | 1.404 | 1.986 | 0.059 | 1.088 | 1.168 | 1.243 | 1.314 | 1.381 |
| 10.4 | 1.884 | 0.058 | 1.111 | 1.190 | 1.265 | 1.337 | 1.405 | 1.975 | 0.058 | 1.090 | 1.170 | 1.245 | 1.316 | 1.383 |
| 10.5 | 1.852 | 0.058 | 1.111 | 1.190 | 1.265 | 1.337 | 1.405 | 1.963 | 0.058 | 1.092 | 1.172 | 1.247 | 1.318 | 1.385 |
| 10.6 | 1.820 | 0.058 | 1.112 | 1.191 | 1.265 | 1.337 | 1.405 | 1.952 | 0.058 | 1.094 | 1.174 | 1.249 | 1.320 | 1.387 |
| 10.7 | 1.787 | 0.058 | 1.112 | 1.191 | 1.266 | 1.337 | 1.405 | 1.940 | 0.058 | 1.097 | 1.176 | 1.251 | 1.322 | 1.389 |
| 10.8 | 1.755 | 0.058 | 1.113 | 1.191 | 1.266 | 1.337 | 1.406 | 1.929 | 0.058 | 1.099 | 1.179 | 1.253 | 1.324 | 1.391 |
| 10.9 | 1.723 | 0.058 | 1.114 | 1.192 | 1.266 | 1.337 | 1.406 | 1.918 | 0.058 | 1.102 | 1.181 | 1.256 | 1.326 | 1.394 |
| 11.0 | 1.691 | 0.057 | 1.114 | 1.192 | 1.266 | 1.338 | 1.407 | 1.907 | 0.058 | 1.105 | 1.184 | 1.258 | 1.329 | 1.396 |
| 11.1 | 1.659 | 0.057 | 1.115 | 1.192 | 1.267 | 1.338 | 1.407 | 1.896 | 0.058 | 1.108 | 1.187 | 1.261 | 1.332 | 1.399 |
| 11.2 | 1.628 | 0.057 | 1.116 | 1.193 | 1.267 | 1.338 | 1.408 | 1.885 | 0.057 | 1.111 | 1.190 | 1.264 | 1.335 | 1.402 |
| 11.3 | 1.596 | 0.057 | 1.117 | 1.193 | 1.267 | 1.339 | 1.408 | 1.874 | 0.057 | 1.114 | 1.193 | 1.267 | 1.338 | 1.406 |
| 11.4 | 1.565 | 0.057 | 1.118 | 1.194 | 1.268 | 1.339 | 1.409 | 1.864 | 0.057 | 1.118 | 1.196 | 1.271 | 1.341 | 1.409 |
| 11.5 | 1.534 | 0.057 | 1.118 | 1.195 | 1.268 | 1.340 | 1.410 | 1.853 | 0.057 | 1.121 | 1.200 | 1.274 | 1.345 | 1.413 |
| 11.6 | 1.503 | 0.057 | 1.119 | 1.195 | 1.269 | 1.341 | 1.410 | 1.843 | 0.057 | 1.125 | 1.203 | 1.278 | 1.349 | 1.416 |
| 11.7 | 1.473 | 0.057 | 1.121 | 1.196 | 1.270 | 1.341 | 1.411 | 1.832 | 0.057 | 1.129 | 1.207 | 1.281 | 1.352 | 1.420 |
| 11.8 | 1.443 | 0.057 | 1.122 | 1.197 | 1.271 | 1.342 | 1.412 | 1.822 | 0.056 | 1.133 | 1.211 | 1.285 | 1.356 | 1.424 |
| 11.9 | 1.413 | 0.057 | 1.123 | 1.198 | 1.271 | 1.343 | 1.413 | 1.812 | 0.056 | 1.137 | 1.215 | 1.289 | 1.360 | 1.428 |
| 12.0 | 1.384 | 0.057 | 1.124 | 1.199 | 1.272 | 1.344 | 1.414 | 1.802 | 0.056 | 1.141 | 1.219 | 1.293 | 1.364 | 1.432 |
| 12.1 | 1.354 | 0.057 | 1.126 | 1.201 | 1.274 | 1.345 | 1.415 | 1.792 | 0.056 | 1.146 | 1.224 | 1.298 | 1.368 | 1.436 |
| 12.2 | 1.326 | 0.057 | 1.128 | 1.202 | 1.275 | 1.346 | 1.417 | 1.782 | 0.056 | 1.150 | 1.228 | 1.302 | 1.373 | 1.441 |
| 12.3 | 1.297 | 0.057 | 1.129 | 1.203 | 1.276 | 1.348 | 1.418 | 1.772 | 0.055 | 1.155 | 1.232 | 1.306 | 1.377 | 1.445 |
| 12.4 | 1.269 | 0.056 | 1.131 | 1.205 | 1.278 | 1.349 | 1.420 | 1.763 | 0.055 | 1.159 | 1.236 | 1.310 | 1.381 | 1.449 |
| 12.5 | 1.242 | 0.056 | 1.133 | 1.207 | 1.279 | 1.351 | 1.421 | 1.753 | 0.055 | 1.164 | 1.241 | 1.315 | 1.385 | 1.454 |
| 12.6 | 1.215 | 0.056 | 1.135 | 1.209 | 1.281 | 1.352 | 1.423 | 1.744 | 0.055 | 1.168 | 1.245 | 1.319 | 1.390 | 1.458 |
| 12.7 | 1.188 | 0.056 | 1.138 | 1.211 | 1.283 | 1.354 | 1.425 | 1.735 | 0.054 | 1.173 | 1.250 | 1.323 | 1.394 | 1.462 |
| 12.8 | 1.162 | 0.056 | 1.140 | 1.213 | 1.285 | 1.356 | 1.427 | 1.725 | 0.054 | 1.177 | 1.254 | 1.327 | 1.398 | 1.466 |
| 12.9 | 1.136 | 0.056 | 1.143 | 1.215 | 1.287 | 1.359 | 1.429 | 1.716 | 0.054 | 1.182 | 1.258 | 1.332 | 1.402 | 1.470 |
| 13.0 | 1.110 | 0.055 | 1.146 | 1.218 | 1.290 | 1.361 | 1.432 | 1.707 | 0.054 | 1.186 | 1.262 | 1.336 | 1.406 | 1.474 |
| 13.1 | 1.085 | 0.055 | 1.149 | 1.220 | 1.292 | 1.363 | 1.434 | 1.699 | 0.054 | 1.190 | 1.267 | 1.340 | 1.410 | 1.478 |
| 13.2 | 1.061 | 0.055 | 1.152 | 1.223 | 1.295 | 1.366 | 1.437 | 1.690 | 0.053 | 1.195 | 1.271 | 1.344 | 1.414 | 1.482 |
| 13.3 | 1.037 | 0.055 | 1.155 | 1.226 | 1.297 | 1.369 | 1.439 | 1.681 | 0.053 | 1.199 | 1.275 | 1.348 | 1.418 | 1.486 |
| 13.4 | 1.013 | 0.055 | 1.158 | 1.229 | 1.300 | 1.371 | 1.442 | 1.673 | 0.053 | 1.203 | 1.278 | 1.351 | 1.422 | 1.490 |
| 13.5 | 0.990 | 0.054 | 1.162 | 1.232 | 1.303 | 1.374 | 1.445 | 1.664 | 0.053 | 1.207 | 1.282 | 1.355 | 1.425 | 1.493 |
| 13.6 | 0.968 | 0.054 | 1.165 | 1.236 | 1.306 | 1.377 | 1.448 | 1.656 | 0.052 | 1.211 | 1.286 | 1.358 | 1.428 | 1.496 |
| 13.7 | 0.946 | 0.054 | 1.169 | 1.239 | 1.310 | 1.380 | 1.451 | 1.648 | 0.052 | 1.214 | 1.289 | 1.362 | 1.432 | 1.500 |
| 13.8 | 0.925 | 0.054 | 1.173 | 1.243 | 1.313 | 1.384 | 1.454 | 1.640 | 0.052 | 1.218 | 1.293 | 1.365 | 1.435 | 1.503 |
| 13.9 | 0.904 | 0.053 | 1.176 | 1.246 | 1.316 | 1.387 | 1.458 | 1.632 | 0.052 | 1.221 | 1.296 | 1.368 | 1.438 | 1.505 |
| 14.0 | 0.884 | 0.053 | 1.180 | 1.250 | 1.320 | 1.390 | 1.461 | 1.624 | 0.052 | 1.225 | 1.299 | 1.371 | 1.441 | 1.508 |
| 14.1 | 0.864 | 0.053 | 1.184 | 1.254 | 1.323 | 1.393 | 1.464 | 1.617 | 0.051 | 1.228 | 1.302 | 1.374 | 1.443 | 1.511 |
| 14.2 | 0.846 | 0.053 | 1.188 | 1.257 | 1.327 | 1.397 | 1.468 | 1.609 | 0.051 | 1.231 | 1.305 | 1.376 | 1.446 | 1.513 |
| 14.3 | 0.828 | 0.052 | 1.192 | 1.261 | 1.330 | 1.400 | 1.471 | 1.601 | 0.051 | 1.234 | 1.308 | 1.379 | 1.448 | 1.516 |
| 14.4 | 0.810 | 0.052 | 1.197 | 1.265 | 1.334 | 1.404 | 1.474 | 1.594 | 0.051 | 1.237 | 1.310 | 1.382 | 1.451 | 1.518 |
| 14.5 | 0.794 | 0.052 | 1.201 | 1.269 | 1.338 | 1.407 | 1.477 | 1.587 | 0.051 | 1.239 | 1.313 | 1.384 | 1.453 | 1.520 |
| 14.6 | 0.778 | 0.051 | 1.205 | 1.273 | 1.341 | 1.411 | 1.481 | 1.580 | 0.050 | 1.242 | 1.315 | 1.386 | 1.455 | 1.522 |
| 14.7 | 0.763 | 0.051 | 1.209 | 1.277 | 1.345 | 1.414 | 1.484 | 1.573 | 0.050 | 1.245 | 1.318 | 1.388 | 1.457 | 1.524 |
| 14.8 | 0.749 | 0.051 | 1.213 | 1.281 | 1.349 | 1.418 | 1.487 | 1.566 | 0.050 | 1.247 | 1.320 | 1.390 | 1.459 | 1.525 |
| 14.9 | 0.735 | 0.050 | 1.218 | 1.284 | 1.352 | 1.421 | 1.491 | 1.559 | 0.050 | 1.249 | 1.322 | 1.392 | 1.461 | 1.527 |
| 15.0 | 0.723 | 0.050 | 1.222 | 1.288 | 1.356 | 1.424 | 1.494 | 1.552 | 0.050 | 1.252 | 1.324 | 1.394 | 1.462 | 1.529 |
| 15.1 | 0.711 | 0.050 | 1.226 | 1.292 | 1.359 | 1.428 | 1.497 | 1.545 | 0.049 | 1.254 | 1.326 | 1.396 | 1.464 | 1.530 |
| 15.2 | 0.700 | 0.049 | 1.230 | 1.296 | 1.363 | 1.431 | 1.500 | 1.539 | 0.049 | 1.256 | 1.328 | 1.397 | 1.465 | 1.532 |
| 15.3 | 0.690 | 0.049 | 1.234 | 1.300 | 1.366 | 1.434 | 1.503 | 1.532 | 0.049 | 1.258 | 1.329 | 1.399 | 1.467 | 1.533 |
| 15.4 | 0.681 | 0.049 | 1.238 | 1.304 | 1.370 | 1.437 | 1.506 | 1.526 | 0.049 | 1.260 | 1.331 | 1.400 | 1.468 | 1.534 |
| 15.5 | 0.673 | 0.049 | 1.242 | 1.307 | 1.373 | 1.440 | 1.509 | 1.520 | 0.049 | 1.261 | 1.333 | 1.402 | 1.469 | 1.535 |
| 15.6 | 0.666 | 0.048 | 1.246 | 1.311 | 1.377 | 1.443 | 1.511 | 1.513 | 0.049 | 1.263 | 1.334 | 1.403 | 1.471 | 1.536 |
| 15.7 | 0.659 | 0.048 | 1.250 | 1.314 | 1.380 | 1.446 | 1.514 | 1.507 | 0.048 | 1.265 | 1.336 | 1.404 | 1.472 | 1.537 |
| 15.8 | 0.654 | 0.048 | 1.254 | 1.318 | 1.383 | 1.449 | 1.517 | 1.501 | 0.048 | 1.266 | 1.337 | 1.406 | 1.473 | 1.538 |
| 15.9 | 0.649 | 0.047 | 1.257 | 1.321 | 1.386 | 1.452 | 1.519 | 1.495 | 0.048 | 1.268 | 1.338 | 1.407 | 1.474 | 1.539 |
| 16.0 | 0.646 | 0.047 | 1.261 | 1.324 | 1.389 | 1.455 | 1.522 | 1.489 | 0.048 | 1.269 | 1.340 | 1.408 | 1.475 | 1.540 |
| 16.1 | 0.643 | 0.047 | 1.264 | 1.328 | 1.392 | 1.458 | 1.524 | 1.484 | 0.048 | 1.271 | 1.341 | 1.409 | 1.475 | 1.541 |
| 16.2 | 0.642 | 0.046 | 1.268 | 1.331 | 1.395 | 1.460 | 1.526 | 1.478 | 0.048 | 1.272 | 1.342 | 1.410 | 1.476 | 1.541 |
| 16.3 | 0.642 | 0.046 | 1.271 | 1.334 | 1.398 | 1.463 | 1.529 | 1.472 | 0.047 | 1.274 | 1.343 | 1.411 | 1.477 | 1.542 |
| 16.4 | 0.642 | 0.046 | 1.274 | 1.336 | 1.400 | 1.465 | 1.531 | 1.467 | 0.047 | 1.275 | 1.344 | 1.412 | 1.478 | 1.543 |
| 16.5 | 0.644 | 0.046 | 1.277 | 1.339 | 1.403 | 1.467 | 1.533 | 1.461 | 0.047 | 1.276 | 1.345 | 1.413 | 1.479 | 1.543 |
| 16.6 | 0.646 | 0.045 | 1.280 | 1.342 | 1.405 | 1.469 | 1.535 | 1.456 | 0.047 | 1.277 | 1.346 | 1.413 | 1.479 | 1.544 |
| 16.7 | 0.650 | 0.045 | 1.282 | 1.344 | 1.408 | 1.472 | 1.537 | 1.451 | 0.047 | 1.279 | 1.347 | 1.414 | 1.480 | 1.544 |
| 16.8 | 0.654 | 0.045 | 1.285 | 1.347 | 1.410 | 1.474 | 1.538 | 1.445 | 0.047 | 1.280 | 1.348 | 1.415 | 1.480 | 1.545 |
| 16.9 | 0.660 | 0.045 | 1.288 | 1.349 | 1.412 | 1.476 | 1.540 | 1.440 | 0.047 | 1.281 | 1.349 | 1.416 | 1.481 | 1.545 |
| 17.0 | 0.667 | 0.045 | 1.290 | 1.351 | 1.414 | 1.477 | 1.542 | 1.435 | 0.046 | 1.282 | 1.350 | 1.417 | 1.482 | 1.546 |
| 17.1 | 0.674 | 0.044 | 1.292 | 1.354 | 1.416 | 1.479 | 1.543 | 1.430 | 0.046 | 1.283 | 1.351 | 1.417 | 1.482 | 1.546 |
| 17.2 | 0.683 | 0.044 | 1.294 | 1.356 | 1.418 | 1.481 | 1.545 | 1.425 | 0.046 | 1.284 | 1.352 | 1.418 | 1.483 | 1.547 |
| 17.3 | 0.693 | 0.044 | 1.296 | 1.357 | 1.419 | 1.482 | 1.546 | 1.420 | 0.046 | 1.285 | 1.353 | 1.419 | 1.484 | 1.547 |
| 17.4 | 0.703 | 0.044 | 1.298 | 1.359 | 1.421 | 1.484 | 1.547 | 1.415 | 0.046 | 1.287 | 1.354 | 1.420 | 1.484 | 1.548 |
| 17.5 | 0.715 | 0.044 | 1.300 | 1.361 | 1.423 | 1.485 | 1.549 | 1.410 | 0.046 | 1.288 | 1.355 | 1.420 | 1.485 | 1.548 |
| 17.6 | 0.727 | 0.044 | 1.301 | 1.362 | 1.424 | 1.487 | 1.550 | 1.405 | 0.046 | 1.289 | 1.356 | 1.421 | 1.486 | 1.549 |
| 17.7 | 0.741 | 0.044 | 1.303 | 1.364 | 1.426 | 1.488 | 1.551 | 1.401 | 0.045 | 1.290 | 1.357 | 1.422 | 1.486 | 1.549 |
| 17.8 | 0.755 | 0.043 | 1.304 | 1.365 | 1.427 | 1.489 | 1.552 | 1.396 | 0.045 | 1.291 | 1.358 | 1.423 | 1.487 | 1.550 |
| 17.9 | 0.770 | 0.043 | 1.306 | 1.367 | 1.428 | 1.490 | 1.553 | 1.391 | 0.045 | 1.292 | 1.359 | 1.423 | 1.487 | 1.550 |
| 18.0 | 0.786 | 0.043 | 1.307 | 1.368 | 1.429 | 1.491 | 1.554 | 1.387 | 0.045 | 1.293 | 1.359 | 1.424 | 1.488 | 1.550 |
| 18.1 | 0.803 | 0.043 | 1.308 | 1.369 | 1.430 | 1.492 | 1.555 | 1.382 | 0.045 | 1.295 | 1.360 | 1.425 | 1.488 | 1.551 |
| 18.2 | 0.820 | 0.043 | 1.309 | 1.370 | 1.431 | 1.493 | 1.556 | 1.378 | 0.045 | 1.296 | 1.361 | 1.426 | 1.489 | 1.551 |
| 18.3 | 0.838 | 0.043 | 1.310 | 1.371 | 1.432 | 1.494 | 1.557 | 1.373 | 0.045 | 1.297 | 1.362 | 1.426 | 1.489 | 1.551 |
| 18.4 | 0.857 | 0.043 | 1.311 | 1.372 | 1.433 | 1.495 | 1.558 | 1.369 | 0.044 | 1.298 | 1.363 | 1.427 | 1.490 | 1.552 |
| 18.5 | 0.876 | 0.043 | 1.312 | 1.373 | 1.434 | 1.496 | 1.558 | 1.364 | 0.044 | 1.298 | 1.363 | 1.427 | 1.490 | 1.552 |
| 18.6 | 0.896 | 0.043 | 1.312 | 1.374 | 1.435 | 1.497 | 1.559 | 1.360 | 0.044 | 1.299 | 1.364 | 1.428 | 1.490 | 1.552 |
| 18.7 | 0.917 | 0.043 | 1.313 | 1.374 | 1.436 | 1.498 | 1.560 | 1.355 | 0.044 | 1.300 | 1.365 | 1.428 | 1.490 | 1.552 |
| 18.8 | 0.938 | 0.043 | 1.314 | 1.375 | 1.437 | 1.499 | 1.561 | 1.351 | 0.044 | 1.301 | 1.365 | 1.428 | 1.491 | 1.552 |
| 18.9 | 0.959 | 0.043 | 1.314 | 1.376 | 1.438 | 1.499 | 1.561 | 1.346 | 0.044 | 1.302 | 1.366 | 1.429 | 1.491 | 1.552 |
| 19.0 | 0.981 | 0.043 | 1.315 | 1.377 | 1.438 | 1.500 | 1.562 | 1.342 | 0.044 | 1.302 | 1.366 | 1.429 | 1.491 | 1.552 |
| 19.1 | 1.003 | 0.043 | 1.315 | 1.377 | 1.439 | 1.501 | 1.563 | 1.338 | 0.043 | 1.303 | 1.367 | 1.429 | 1.491 | 1.552 |
| 19.2 | 1.026 | 0.043 | 1.316 | 1.378 | 1.440 | 1.502 | 1.564 | 1.333 | 0.043 | 1.304 | 1.367 | 1.429 | 1.491 | 1.551 |
| 19.3 | 1.049 | 0.043 | 1.316 | 1.379 | 1.441 | 1.503 | 1.564 | 1.329 | 0.043 | 1.304 | 1.367 | 1.429 | 1.491 | 1.551 |
| 19.4 | 1.072 | 0.043 | 1.317 | 1.379 | 1.441 | 1.503 | 1.565 | 1.325 | 0.043 | 1.305 | 1.368 | 1.429 | 1.491 | 1.551 |
| 19.5 | 1.096 | 0.043 | 1.317 | 1.380 | 1.442 | 1.504 | 1.566 | 1.320 | 0.043 | 1.305 | 1.368 | 1.429 | 1.490 | 1.550 |
| 19.6 | 1.120 | 0.043 | 1.318 | 1.380 | 1.443 | 1.505 | 1.566 | 1.316 | 0.043 | 1.306 | 1.368 | 1.429 | 1.490 | 1.550 |
| 19.7 | 1.144 | 0.043 | 1.318 | 1.381 | 1.443 | 1.505 | 1.567 | 1.312 | 0.043 | 1.306 | 1.368 | 1.429 | 1.490 | 1.550 |
| 19.8 | 1.169 | 0.043 | 1.318 | 1.381 | 1.444 | 1.506 | 1.568 | 1.308 | 0.042 | 1.306 | 1.368 | 1.429 | 1.490 | 1.549 |
| 19.9 | 1.193 | 0.043 | 1.318 | 1.382 | 1.444 | 1.506 | 1.568 | 1.303 | 0.042 | 1.307 | 1.368 | 1.429 | 1.489 | 1.548 |
| 20.0 | 1.218 | 0.043 | 1.319 | 1.382 | 1.445 | 1.507 | 1.569 | 1.299 | 0.042 | 1.307 | 1.369 | 1.429 | 1.489 | 1.548 |

| L1(L2)L3L4 TBS | | | | | | | | | | | | | | |
| --- | --- | --- | --- | --- | --- | --- | --- | --- | --- | --- | --- | --- | --- | --- |
|  | Males | | | | | | | Females | | | | | | |
| Age | L | S | -2 SD | -1 SD | 0 SD (M) | 1 SD | 2 SD | L | S | -2 SD | -1 SD | 0 SD (M) | 1 SD | 2 SD |
| 5.0 | 4.018 | 0.057 | 1.096 | 1.196 | 1.276 | 1.343 | 1.401 | 2.230 | 0.059 | 1.089 | 1.172 | 1.248 | 1.319 | 1.386 |
| 5.1 | 3.968 | 0.057 | 1.097 | 1.197 | 1.276 | 1.344 | 1.402 | 2.221 | 0.059 | 1.089 | 1.172 | 1.248 | 1.319 | 1.385 |
| 5.2 | 3.919 | 0.057 | 1.098 | 1.197 | 1.277 | 1.344 | 1.403 | 2.213 | 0.059 | 1.088 | 1.171 | 1.247 | 1.318 | 1.385 |
| 5.3 | 3.870 | 0.057 | 1.099 | 1.198 | 1.277 | 1.344 | 1.403 | 2.204 | 0.059 | 1.088 | 1.171 | 1.247 | 1.318 | 1.384 |
| 5.4 | 3.821 | 0.057 | 1.101 | 1.198 | 1.277 | 1.345 | 1.404 | 2.195 | 0.059 | 1.088 | 1.170 | 1.246 | 1.317 | 1.384 |
| 5.5 | 3.772 | 0.057 | 1.102 | 1.199 | 1.278 | 1.345 | 1.404 | 2.186 | 0.059 | 1.087 | 1.170 | 1.246 | 1.317 | 1.383 |
| 5.6 | 3.723 | 0.057 | 1.103 | 1.199 | 1.278 | 1.346 | 1.405 | 2.178 | 0.059 | 1.087 | 1.169 | 1.245 | 1.316 | 1.383 |
| 5.7 | 3.675 | 0.057 | 1.104 | 1.200 | 1.279 | 1.346 | 1.406 | 2.169 | 0.059 | 1.087 | 1.169 | 1.245 | 1.316 | 1.383 |
| 5.8 | 3.627 | 0.057 | 1.105 | 1.200 | 1.279 | 1.346 | 1.406 | 2.160 | 0.059 | 1.086 | 1.168 | 1.244 | 1.315 | 1.382 |
| 5.9 | 3.579 | 0.057 | 1.106 | 1.201 | 1.279 | 1.347 | 1.407 | 2.151 | 0.059 | 1.086 | 1.168 | 1.244 | 1.315 | 1.382 |
| 6.0 | 3.531 | 0.057 | 1.107 | 1.201 | 1.279 | 1.347 | 1.407 | 2.143 | 0.059 | 1.086 | 1.168 | 1.244 | 1.315 | 1.381 |
| 6.1 | 3.483 | 0.057 | 1.108 | 1.201 | 1.280 | 1.347 | 1.407 | 2.134 | 0.059 | 1.085 | 1.167 | 1.243 | 1.314 | 1.381 |
| 6.2 | 3.436 | 0.056 | 1.109 | 1.202 | 1.280 | 1.348 | 1.408 | 2.125 | 0.059 | 1.085 | 1.167 | 1.243 | 1.314 | 1.381 |
| 6.3 | 3.389 | 0.056 | 1.110 | 1.202 | 1.280 | 1.348 | 1.408 | 2.116 | 0.059 | 1.085 | 1.167 | 1.242 | 1.313 | 1.380 |
| 6.4 | 3.343 | 0.056 | 1.111 | 1.202 | 1.280 | 1.348 | 1.409 | 2.107 | 0.059 | 1.084 | 1.166 | 1.242 | 1.313 | 1.380 |
| 6.5 | 3.297 | 0.056 | 1.112 | 1.203 | 1.280 | 1.348 | 1.409 | 2.099 | 0.059 | 1.084 | 1.166 | 1.242 | 1.313 | 1.380 |
| 6.6 | 3.251 | 0.056 | 1.113 | 1.203 | 1.280 | 1.348 | 1.409 | 2.090 | 0.059 | 1.084 | 1.166 | 1.241 | 1.313 | 1.380 |
| 6.7 | 3.205 | 0.056 | 1.113 | 1.203 | 1.280 | 1.348 | 1.409 | 2.081 | 0.059 | 1.084 | 1.165 | 1.241 | 1.312 | 1.379 |
| 6.8 | 3.160 | 0.056 | 1.114 | 1.203 | 1.280 | 1.348 | 1.410 | 2.072 | 0.059 | 1.084 | 1.165 | 1.241 | 1.312 | 1.379 |
| 6.9 | 3.115 | 0.056 | 1.115 | 1.204 | 1.280 | 1.348 | 1.410 | 2.063 | 0.059 | 1.084 | 1.165 | 1.241 | 1.312 | 1.379 |
| 7.0 | 3.071 | 0.056 | 1.116 | 1.204 | 1.280 | 1.348 | 1.410 | 2.054 | 0.059 | 1.084 | 1.165 | 1.241 | 1.312 | 1.379 |
| 7.1 | 3.027 | 0.056 | 1.116 | 1.204 | 1.280 | 1.348 | 1.410 | 2.045 | 0.059 | 1.084 | 1.165 | 1.240 | 1.311 | 1.379 |
| 7.2 | 2.983 | 0.056 | 1.117 | 1.204 | 1.280 | 1.348 | 1.410 | 2.036 | 0.059 | 1.084 | 1.165 | 1.240 | 1.311 | 1.379 |
| 7.3 | 2.940 | 0.056 | 1.118 | 1.204 | 1.280 | 1.348 | 1.410 | 2.027 | 0.059 | 1.084 | 1.165 | 1.240 | 1.311 | 1.379 |
| 7.4 | 2.897 | 0.056 | 1.118 | 1.204 | 1.280 | 1.348 | 1.410 | 2.017 | 0.059 | 1.084 | 1.165 | 1.240 | 1.311 | 1.378 |
| 7.5 | 2.854 | 0.056 | 1.119 | 1.204 | 1.280 | 1.348 | 1.411 | 2.008 | 0.059 | 1.084 | 1.165 | 1.240 | 1.311 | 1.378 |
| 7.6 | 2.812 | 0.056 | 1.119 | 1.205 | 1.280 | 1.348 | 1.411 | 1.999 | 0.059 | 1.084 | 1.165 | 1.240 | 1.311 | 1.378 |
| 7.7 | 2.770 | 0.056 | 1.120 | 1.205 | 1.280 | 1.348 | 1.411 | 1.990 | 0.059 | 1.084 | 1.165 | 1.240 | 1.311 | 1.378 |
| 7.8 | 2.729 | 0.056 | 1.120 | 1.205 | 1.280 | 1.348 | 1.411 | 1.981 | 0.059 | 1.084 | 1.165 | 1.240 | 1.311 | 1.378 |
| 7.9 | 2.688 | 0.056 | 1.121 | 1.205 | 1.280 | 1.348 | 1.411 | 1.971 | 0.059 | 1.084 | 1.165 | 1.240 | 1.311 | 1.378 |
| 8.0 | 2.647 | 0.056 | 1.121 | 1.205 | 1.280 | 1.348 | 1.411 | 1.962 | 0.059 | 1.085 | 1.165 | 1.240 | 1.311 | 1.378 |
| 8.1 | 2.607 | 0.056 | 1.122 | 1.205 | 1.279 | 1.348 | 1.411 | 1.953 | 0.059 | 1.085 | 1.165 | 1.240 | 1.311 | 1.378 |
| 8.2 | 2.567 | 0.056 | 1.122 | 1.205 | 1.279 | 1.348 | 1.411 | 1.943 | 0.059 | 1.085 | 1.165 | 1.240 | 1.311 | 1.378 |
| 8.3 | 2.527 | 0.055 | 1.123 | 1.205 | 1.279 | 1.347 | 1.411 | 1.934 | 0.059 | 1.085 | 1.165 | 1.240 | 1.311 | 1.378 |
| 8.4 | 2.488 | 0.055 | 1.123 | 1.205 | 1.279 | 1.347 | 1.411 | 1.924 | 0.059 | 1.086 | 1.165 | 1.240 | 1.311 | 1.378 |
| 8.5 | 2.449 | 0.055 | 1.124 | 1.205 | 1.279 | 1.347 | 1.411 | 1.915 | 0.059 | 1.086 | 1.165 | 1.240 | 1.311 | 1.378 |
| 8.6 | 2.411 | 0.055 | 1.124 | 1.205 | 1.279 | 1.347 | 1.411 | 1.906 | 0.058 | 1.087 | 1.166 | 1.240 | 1.311 | 1.378 |
| 8.7 | 2.373 | 0.055 | 1.125 | 1.205 | 1.279 | 1.347 | 1.411 | 1.896 | 0.058 | 1.087 | 1.166 | 1.240 | 1.311 | 1.378 |
| 8.8 | 2.335 | 0.055 | 1.125 | 1.205 | 1.279 | 1.347 | 1.411 | 1.887 | 0.058 | 1.088 | 1.166 | 1.241 | 1.311 | 1.379 |
| 8.9 | 2.298 | 0.055 | 1.126 | 1.205 | 1.278 | 1.347 | 1.411 | 1.877 | 0.058 | 1.088 | 1.167 | 1.241 | 1.311 | 1.379 |
| 9.0 | 2.261 | 0.055 | 1.126 | 1.205 | 1.278 | 1.347 | 1.411 | 1.868 | 0.058 | 1.089 | 1.167 | 1.241 | 1.312 | 1.379 |
| 9.1 | 2.225 | 0.055 | 1.126 | 1.205 | 1.278 | 1.346 | 1.411 | 1.858 | 0.058 | 1.089 | 1.168 | 1.242 | 1.312 | 1.379 |
| 9.2 | 2.188 | 0.055 | 1.127 | 1.205 | 1.278 | 1.346 | 1.411 | 1.849 | 0.058 | 1.090 | 1.168 | 1.242 | 1.312 | 1.380 |
| 9.3 | 2.153 | 0.055 | 1.127 | 1.205 | 1.278 | 1.346 | 1.411 | 1.840 | 0.058 | 1.091 | 1.169 | 1.243 | 1.313 | 1.380 |
| 9.4 | 2.117 | 0.055 | 1.127 | 1.205 | 1.278 | 1.346 | 1.411 | 1.830 | 0.058 | 1.092 | 1.169 | 1.243 | 1.313 | 1.381 |
| 9.5 | 2.082 | 0.055 | 1.128 | 1.205 | 1.278 | 1.346 | 1.411 | 1.821 | 0.058 | 1.092 | 1.170 | 1.244 | 1.314 | 1.381 |
| 9.6 | 2.048 | 0.055 | 1.128 | 1.205 | 1.278 | 1.346 | 1.411 | 1.812 | 0.058 | 1.093 | 1.171 | 1.244 | 1.314 | 1.382 |
| 9.7 | 2.014 | 0.055 | 1.128 | 1.205 | 1.277 | 1.346 | 1.411 | 1.802 | 0.058 | 1.094 | 1.172 | 1.245 | 1.315 | 1.382 |
| 9.8 | 1.980 | 0.055 | 1.128 | 1.205 | 1.277 | 1.346 | 1.411 | 1.793 | 0.057 | 1.096 | 1.173 | 1.246 | 1.316 | 1.383 |
| 9.9 | 1.947 | 0.055 | 1.129 | 1.205 | 1.277 | 1.346 | 1.411 | 1.784 | 0.057 | 1.097 | 1.174 | 1.247 | 1.317 | 1.384 |
| 10.0 | 1.914 | 0.055 | 1.129 | 1.205 | 1.277 | 1.345 | 1.411 | 1.775 | 0.057 | 1.098 | 1.175 | 1.248 | 1.318 | 1.385 |
| 10.1 | 1.881 | 0.055 | 1.129 | 1.205 | 1.277 | 1.345 | 1.411 | 1.765 | 0.057 | 1.100 | 1.176 | 1.249 | 1.319 | 1.386 |
| 10.2 | 1.849 | 0.055 | 1.129 | 1.205 | 1.277 | 1.345 | 1.411 | 1.756 | 0.057 | 1.101 | 1.178 | 1.250 | 1.320 | 1.387 |
| 10.3 | 1.818 | 0.055 | 1.129 | 1.205 | 1.277 | 1.345 | 1.411 | 1.747 | 0.057 | 1.103 | 1.179 | 1.252 | 1.322 | 1.389 |
| 10.4 | 1.787 | 0.055 | 1.130 | 1.205 | 1.277 | 1.345 | 1.411 | 1.738 | 0.057 | 1.105 | 1.181 | 1.253 | 1.323 | 1.390 |
| 10.5 | 1.756 | 0.055 | 1.130 | 1.205 | 1.277 | 1.345 | 1.411 | 1.729 | 0.057 | 1.107 | 1.183 | 1.255 | 1.325 | 1.392 |
| 10.6 | 1.726 | 0.055 | 1.130 | 1.205 | 1.277 | 1.345 | 1.412 | 1.720 | 0.056 | 1.109 | 1.184 | 1.257 | 1.326 | 1.393 |
| 10.7 | 1.696 | 0.055 | 1.130 | 1.205 | 1.277 | 1.345 | 1.412 | 1.712 | 0.056 | 1.111 | 1.187 | 1.259 | 1.328 | 1.395 |
| 10.8 | 1.667 | 0.055 | 1.131 | 1.205 | 1.277 | 1.345 | 1.412 | 1.703 | 0.056 | 1.113 | 1.189 | 1.261 | 1.331 | 1.398 |
| 10.9 | 1.638 | 0.055 | 1.131 | 1.205 | 1.277 | 1.346 | 1.412 | 1.694 | 0.056 | 1.116 | 1.191 | 1.263 | 1.333 | 1.400 |
| 11.0 | 1.610 | 0.055 | 1.132 | 1.205 | 1.277 | 1.346 | 1.413 | 1.685 | 0.056 | 1.119 | 1.194 | 1.266 | 1.335 | 1.402 |
| 11.1 | 1.582 | 0.055 | 1.132 | 1.206 | 1.277 | 1.346 | 1.413 | 1.677 | 0.056 | 1.122 | 1.197 | 1.269 | 1.338 | 1.405 |
| 11.2 | 1.555 | 0.055 | 1.132 | 1.206 | 1.277 | 1.346 | 1.413 | 1.668 | 0.056 | 1.125 | 1.200 | 1.272 | 1.341 | 1.408 |
| 11.3 | 1.528 | 0.055 | 1.133 | 1.206 | 1.277 | 1.346 | 1.414 | 1.660 | 0.055 | 1.128 | 1.203 | 1.275 | 1.344 | 1.411 |
| 11.4 | 1.502 | 0.055 | 1.134 | 1.207 | 1.278 | 1.347 | 1.414 | 1.652 | 0.055 | 1.131 | 1.206 | 1.278 | 1.347 | 1.414 |
| 11.5 | 1.477 | 0.055 | 1.134 | 1.207 | 1.278 | 1.347 | 1.415 | 1.643 | 0.055 | 1.135 | 1.210 | 1.281 | 1.351 | 1.418 |
| 11.6 | 1.452 | 0.055 | 1.135 | 1.208 | 1.279 | 1.348 | 1.415 | 1.635 | 0.055 | 1.139 | 1.213 | 1.285 | 1.354 | 1.421 |
| 11.7 | 1.428 | 0.055 | 1.136 | 1.208 | 1.279 | 1.348 | 1.416 | 1.627 | 0.055 | 1.142 | 1.217 | 1.288 | 1.358 | 1.425 |
| 11.8 | 1.404 | 0.055 | 1.137 | 1.209 | 1.280 | 1.349 | 1.416 | 1.619 | 0.054 | 1.146 | 1.221 | 1.292 | 1.361 | 1.428 |
| 11.9 | 1.381 | 0.055 | 1.138 | 1.210 | 1.280 | 1.349 | 1.417 | 1.611 | 0.054 | 1.150 | 1.225 | 1.296 | 1.365 | 1.432 |
| 12.0 | 1.358 | 0.054 | 1.139 | 1.211 | 1.281 | 1.350 | 1.418 | 1.603 | 0.054 | 1.155 | 1.229 | 1.300 | 1.369 | 1.436 |
| 12.1 | 1.336 | 0.054 | 1.140 | 1.212 | 1.282 | 1.351 | 1.419 | 1.595 | 0.054 | 1.159 | 1.233 | 1.304 | 1.373 | 1.440 |
| 12.2 | 1.314 | 0.054 | 1.141 | 1.213 | 1.283 | 1.352 | 1.420 | 1.588 | 0.054 | 1.163 | 1.237 | 1.308 | 1.377 | 1.444 |
| 12.3 | 1.293 | 0.054 | 1.143 | 1.214 | 1.285 | 1.354 | 1.422 | 1.580 | 0.053 | 1.168 | 1.241 | 1.312 | 1.381 | 1.448 |
| 12.4 | 1.272 | 0.054 | 1.145 | 1.216 | 1.286 | 1.355 | 1.423 | 1.572 | 0.053 | 1.172 | 1.245 | 1.316 | 1.385 | 1.452 |
| 12.5 | 1.252 | 0.054 | 1.147 | 1.218 | 1.287 | 1.356 | 1.425 | 1.565 | 0.053 | 1.176 | 1.250 | 1.321 | 1.390 | 1.457 |
| 12.6 | 1.233 | 0.054 | 1.149 | 1.219 | 1.289 | 1.358 | 1.426 | 1.558 | 0.053 | 1.181 | 1.254 | 1.325 | 1.394 | 1.461 |
| 12.7 | 1.214 | 0.054 | 1.151 | 1.221 | 1.291 | 1.360 | 1.428 | 1.550 | 0.052 | 1.185 | 1.258 | 1.329 | 1.398 | 1.465 |
| 12.8 | 1.196 | 0.054 | 1.153 | 1.223 | 1.293 | 1.362 | 1.430 | 1.543 | 0.052 | 1.190 | 1.263 | 1.333 | 1.402 | 1.469 |
| 12.9 | 1.178 | 0.053 | 1.156 | 1.226 | 1.295 | 1.364 | 1.433 | 1.536 | 0.052 | 1.194 | 1.267 | 1.337 | 1.406 | 1.473 |
| 13.0 | 1.161 | 0.053 | 1.158 | 1.228 | 1.298 | 1.367 | 1.435 | 1.529 | 0.052 | 1.198 | 1.271 | 1.341 | 1.410 | 1.477 |
| 13.1 | 1.144 | 0.053 | 1.161 | 1.231 | 1.300 | 1.369 | 1.437 | 1.522 | 0.052 | 1.203 | 1.275 | 1.345 | 1.414 | 1.481 |
| 13.2 | 1.128 | 0.053 | 1.164 | 1.234 | 1.303 | 1.372 | 1.440 | 1.516 | 0.051 | 1.207 | 1.279 | 1.349 | 1.418 | 1.484 |
| 13.3 | 1.113 | 0.053 | 1.167 | 1.237 | 1.306 | 1.374 | 1.443 | 1.509 | 0.051 | 1.211 | 1.283 | 1.353 | 1.421 | 1.488 |
| 13.4 | 1.098 | 0.053 | 1.170 | 1.240 | 1.309 | 1.377 | 1.446 | 1.503 | 0.051 | 1.215 | 1.287 | 1.357 | 1.425 | 1.491 |
| 13.5 | 1.084 | 0.052 | 1.174 | 1.243 | 1.312 | 1.381 | 1.449 | 1.496 | 0.051 | 1.219 | 1.290 | 1.360 | 1.428 | 1.495 |
| 13.6 | 1.071 | 0.052 | 1.177 | 1.246 | 1.315 | 1.384 | 1.452 | 1.490 | 0.050 | 1.223 | 1.294 | 1.364 | 1.432 | 1.498 |
| 13.7 | 1.058 | 0.052 | 1.181 | 1.250 | 1.319 | 1.387 | 1.455 | 1.484 | 0.050 | 1.226 | 1.298 | 1.367 | 1.435 | 1.501 |
| 13.8 | 1.047 | 0.052 | 1.185 | 1.254 | 1.322 | 1.390 | 1.459 | 1.478 | 0.050 | 1.230 | 1.301 | 1.370 | 1.438 | 1.504 |
| 13.9 | 1.035 | 0.052 | 1.189 | 1.257 | 1.326 | 1.394 | 1.462 | 1.472 | 0.050 | 1.233 | 1.304 | 1.373 | 1.441 | 1.507 |
| 14.0 | 1.025 | 0.051 | 1.193 | 1.261 | 1.329 | 1.398 | 1.466 | 1.466 | 0.050 | 1.237 | 1.307 | 1.376 | 1.444 | 1.510 |
| 14.1 | 1.015 | 0.051 | 1.197 | 1.265 | 1.333 | 1.401 | 1.469 | 1.460 | 0.049 | 1.240 | 1.310 | 1.379 | 1.446 | 1.512 |
| 14.2 | 1.007 | 0.051 | 1.201 | 1.269 | 1.337 | 1.405 | 1.473 | 1.454 | 0.049 | 1.243 | 1.313 | 1.382 | 1.449 | 1.515 |
| 14.3 | 0.999 | 0.051 | 1.205 | 1.273 | 1.341 | 1.408 | 1.476 | 1.449 | 0.049 | 1.246 | 1.316 | 1.384 | 1.451 | 1.517 |
| 14.4 | 0.991 | 0.050 | 1.209 | 1.277 | 1.344 | 1.412 | 1.480 | 1.443 | 0.049 | 1.249 | 1.319 | 1.387 | 1.454 | 1.519 |
| 14.5 | 0.985 | 0.050 | 1.213 | 1.281 | 1.348 | 1.416 | 1.483 | 1.438 | 0.048 | 1.252 | 1.321 | 1.389 | 1.456 | 1.521 |
| 14.6 | 0.979 | 0.050 | 1.217 | 1.285 | 1.352 | 1.419 | 1.487 | 1.433 | 0.048 | 1.254 | 1.324 | 1.392 | 1.458 | 1.523 |
| 14.7 | 0.975 | 0.050 | 1.222 | 1.289 | 1.356 | 1.423 | 1.490 | 1.428 | 0.048 | 1.257 | 1.326 | 1.394 | 1.460 | 1.525 |
| 14.8 | 0.971 | 0.049 | 1.226 | 1.293 | 1.360 | 1.427 | 1.494 | 1.423 | 0.048 | 1.259 | 1.328 | 1.396 | 1.462 | 1.527 |
| 14.9 | 0.968 | 0.049 | 1.230 | 1.297 | 1.363 | 1.430 | 1.497 | 1.418 | 0.048 | 1.262 | 1.331 | 1.398 | 1.464 | 1.529 |
| 15.0 | 0.966 | 0.049 | 1.234 | 1.300 | 1.367 | 1.434 | 1.501 | 1.413 | 0.047 | 1.264 | 1.333 | 1.400 | 1.466 | 1.530 |
| 15.1 | 0.965 | 0.049 | 1.238 | 1.304 | 1.371 | 1.437 | 1.504 | 1.409 | 0.047 | 1.266 | 1.335 | 1.402 | 1.467 | 1.532 |
| 15.2 | 0.964 | 0.048 | 1.242 | 1.308 | 1.374 | 1.441 | 1.507 | 1.404 | 0.047 | 1.268 | 1.337 | 1.403 | 1.469 | 1.533 |
| 15.3 | 0.965 | 0.048 | 1.246 | 1.312 | 1.378 | 1.444 | 1.510 | 1.400 | 0.047 | 1.270 | 1.338 | 1.405 | 1.470 | 1.535 |
| 15.4 | 0.966 | 0.048 | 1.250 | 1.316 | 1.381 | 1.447 | 1.513 | 1.396 | 0.047 | 1.272 | 1.340 | 1.406 | 1.472 | 1.536 |
| 15.5 | 0.968 | 0.047 | 1.254 | 1.319 | 1.385 | 1.451 | 1.516 | 1.391 | 0.047 | 1.274 | 1.342 | 1.408 | 1.473 | 1.537 |
| 15.6 | 0.972 | 0.047 | 1.257 | 1.323 | 1.388 | 1.454 | 1.519 | 1.387 | 0.046 | 1.276 | 1.343 | 1.409 | 1.474 | 1.538 |
| 15.7 | 0.976 | 0.047 | 1.261 | 1.326 | 1.391 | 1.457 | 1.522 | 1.383 | 0.046 | 1.278 | 1.345 | 1.411 | 1.475 | 1.539 |
| 15.8 | 0.981 | 0.047 | 1.265 | 1.330 | 1.395 | 1.460 | 1.525 | 1.380 | 0.046 | 1.279 | 1.346 | 1.412 | 1.476 | 1.540 |
| 15.9 | 0.987 | 0.046 | 1.268 | 1.333 | 1.398 | 1.463 | 1.528 | 1.376 | 0.046 | 1.281 | 1.347 | 1.413 | 1.477 | 1.541 |
| 16.0 | 0.994 | 0.046 | 1.271 | 1.336 | 1.401 | 1.465 | 1.530 | 1.372 | 0.046 | 1.282 | 1.349 | 1.414 | 1.478 | 1.542 |
| 16.1 | 1.002 | 0.046 | 1.275 | 1.339 | 1.404 | 1.468 | 1.532 | 1.369 | 0.046 | 1.283 | 1.350 | 1.415 | 1.479 | 1.542 |
| 16.2 | 1.010 | 0.046 | 1.278 | 1.342 | 1.406 | 1.471 | 1.535 | 1.365 | 0.046 | 1.285 | 1.351 | 1.416 | 1.480 | 1.543 |
| 16.3 | 1.020 | 0.045 | 1.281 | 1.345 | 1.409 | 1.473 | 1.537 | 1.362 | 0.045 | 1.286 | 1.352 | 1.417 | 1.481 | 1.544 |
| 16.4 | 1.031 | 0.045 | 1.284 | 1.348 | 1.412 | 1.475 | 1.539 | 1.359 | 0.045 | 1.287 | 1.353 | 1.418 | 1.481 | 1.544 |
| 16.5 | 1.042 | 0.045 | 1.286 | 1.350 | 1.414 | 1.478 | 1.541 | 1.356 | 0.045 | 1.289 | 1.354 | 1.419 | 1.482 | 1.545 |
| 16.6 | 1.055 | 0.045 | 1.289 | 1.353 | 1.416 | 1.480 | 1.543 | 1.353 | 0.045 | 1.290 | 1.355 | 1.420 | 1.483 | 1.545 |
| 16.7 | 1.068 | 0.045 | 1.292 | 1.355 | 1.419 | 1.482 | 1.545 | 1.350 | 0.045 | 1.291 | 1.356 | 1.420 | 1.484 | 1.546 |
| 16.8 | 1.083 | 0.044 | 1.294 | 1.358 | 1.421 | 1.484 | 1.547 | 1.347 | 0.045 | 1.292 | 1.357 | 1.421 | 1.484 | 1.546 |
| 16.9 | 1.098 | 0.044 | 1.296 | 1.360 | 1.423 | 1.486 | 1.548 | 1.344 | 0.045 | 1.293 | 1.358 | 1.422 | 1.485 | 1.547 |
| 17.0 | 1.114 | 0.044 | 1.299 | 1.362 | 1.425 | 1.487 | 1.550 | 1.342 | 0.044 | 1.294 | 1.359 | 1.423 | 1.485 | 1.547 |
| 17.1 | 1.131 | 0.044 | 1.301 | 1.364 | 1.427 | 1.489 | 1.551 | 1.339 | 0.044 | 1.296 | 1.360 | 1.424 | 1.486 | 1.548 |
| 17.2 | 1.149 | 0.044 | 1.303 | 1.366 | 1.428 | 1.491 | 1.553 | 1.336 | 0.044 | 1.297 | 1.361 | 1.424 | 1.487 | 1.548 |
| 17.3 | 1.168 | 0.044 | 1.305 | 1.368 | 1.430 | 1.492 | 1.554 | 1.334 | 0.044 | 1.298 | 1.362 | 1.425 | 1.487 | 1.549 |
| 17.4 | 1.188 | 0.043 | 1.306 | 1.369 | 1.432 | 1.494 | 1.555 | 1.332 | 0.044 | 1.299 | 1.363 | 1.426 | 1.488 | 1.549 |
| 17.5 | 1.208 | 0.043 | 1.308 | 1.371 | 1.433 | 1.495 | 1.556 | 1.329 | 0.044 | 1.300 | 1.364 | 1.427 | 1.489 | 1.550 |
| 17.6 | 1.229 | 0.043 | 1.309 | 1.372 | 1.435 | 1.496 | 1.558 | 1.327 | 0.044 | 1.301 | 1.365 | 1.428 | 1.489 | 1.550 |
| 17.7 | 1.251 | 0.043 | 1.311 | 1.374 | 1.436 | 1.498 | 1.559 | 1.325 | 0.043 | 1.303 | 1.366 | 1.428 | 1.490 | 1.551 |
| 17.8 | 1.274 | 0.043 | 1.312 | 1.375 | 1.437 | 1.499 | 1.560 | 1.323 | 0.043 | 1.304 | 1.367 | 1.429 | 1.491 | 1.551 |
| 17.9 | 1.297 | 0.043 | 1.314 | 1.376 | 1.439 | 1.500 | 1.561 | 1.321 | 0.043 | 1.305 | 1.368 | 1.430 | 1.491 | 1.552 |
| 18.0 | 1.321 | 0.043 | 1.315 | 1.378 | 1.440 | 1.501 | 1.562 | 1.319 | 0.043 | 1.306 | 1.369 | 1.431 | 1.492 | 1.552 |
| 18.1 | 1.346 | 0.043 | 1.316 | 1.379 | 1.441 | 1.502 | 1.562 | 1.317 | 0.043 | 1.307 | 1.370 | 1.431 | 1.492 | 1.552 |
| 18.2 | 1.371 | 0.043 | 1.317 | 1.380 | 1.442 | 1.503 | 1.563 | 1.315 | 0.043 | 1.308 | 1.370 | 1.432 | 1.493 | 1.553 |
| 18.3 | 1.397 | 0.043 | 1.318 | 1.381 | 1.443 | 1.504 | 1.564 | 1.313 | 0.043 | 1.309 | 1.371 | 1.433 | 1.493 | 1.553 |
| 18.4 | 1.423 | 0.043 | 1.319 | 1.382 | 1.444 | 1.505 | 1.565 | 1.311 | 0.042 | 1.310 | 1.372 | 1.433 | 1.494 | 1.553 |
| 18.5 | 1.450 | 0.043 | 1.320 | 1.383 | 1.445 | 1.506 | 1.566 | 1.310 | 0.042 | 1.311 | 1.373 | 1.434 | 1.494 | 1.553 |
| 18.6 | 1.477 | 0.042 | 1.320 | 1.384 | 1.446 | 1.507 | 1.566 | 1.308 | 0.042 | 1.312 | 1.373 | 1.434 | 1.494 | 1.554 |
| 18.7 | 1.505 | 0.042 | 1.321 | 1.385 | 1.447 | 1.508 | 1.567 | 1.306 | 0.042 | 1.313 | 1.374 | 1.435 | 1.494 | 1.554 |
| 18.8 | 1.533 | 0.042 | 1.322 | 1.385 | 1.448 | 1.508 | 1.568 | 1.304 | 0.042 | 1.313 | 1.375 | 1.435 | 1.495 | 1.554 |
| 18.9 | 1.561 | 0.042 | 1.322 | 1.386 | 1.448 | 1.509 | 1.569 | 1.303 | 0.042 | 1.314 | 1.375 | 1.435 | 1.495 | 1.554 |
| 19.0 | 1.590 | 0.042 | 1.323 | 1.387 | 1.449 | 1.510 | 1.569 | 1.301 | 0.042 | 1.315 | 1.376 | 1.436 | 1.495 | 1.553 |
| 19.1 | 1.619 | 0.042 | 1.324 | 1.388 | 1.450 | 1.511 | 1.570 | 1.299 | 0.041 | 1.315 | 1.376 | 1.436 | 1.495 | 1.553 |
| 19.2 | 1.648 | 0.042 | 1.324 | 1.389 | 1.451 | 1.512 | 1.571 | 1.298 | 0.041 | 1.316 | 1.376 | 1.436 | 1.495 | 1.553 |
| 19.3 | 1.677 | 0.042 | 1.325 | 1.389 | 1.452 | 1.512 | 1.571 | 1.296 | 0.041 | 1.317 | 1.377 | 1.436 | 1.495 | 1.553 |
| 19.4 | 1.707 | 0.042 | 1.325 | 1.390 | 1.452 | 1.513 | 1.572 | 1.294 | 0.041 | 1.317 | 1.377 | 1.436 | 1.495 | 1.552 |
| 19.5 | 1.737 | 0.042 | 1.326 | 1.391 | 1.453 | 1.514 | 1.573 | 1.293 | 0.041 | 1.318 | 1.377 | 1.436 | 1.494 | 1.552 |
| 19.6 | 1.767 | 0.042 | 1.326 | 1.391 | 1.454 | 1.514 | 1.573 | 1.291 | 0.041 | 1.318 | 1.377 | 1.436 | 1.494 | 1.552 |
| 19.7 | 1.797 | 0.042 | 1.327 | 1.392 | 1.454 | 1.515 | 1.574 | 1.290 | 0.040 | 1.318 | 1.378 | 1.436 | 1.494 | 1.551 |
| 19.8 | 1.827 | 0.042 | 1.327 | 1.392 | 1.455 | 1.516 | 1.574 | 1.288 | 0.040 | 1.319 | 1.378 | 1.436 | 1.494 | 1.551 |
| 19.9 | 1.858 | 0.042 | 1.328 | 1.393 | 1.456 | 1.516 | 1.575 | 1.286 | 0.040 | 1.319 | 1.378 | 1.436 | 1.493 | 1.550 |
| 20.0 | 1.888 | 0.042 | 1.328 | 1.393 | 1.456 | 1.517 | 1.575 | 1.285 | 0.040 | 1.320 | 1.378 | 1.436 | 1.493 | 1.550 |

| L1 TBS | | | | | | | | | | | | | | |
| --- | --- | --- | --- | --- | --- | --- | --- | --- | --- | --- | --- | --- | --- | --- |
|  | Males | | | | | | | Females | | | | | | |
| Age | L | S | -2 SD | -1 SD | 0 SD (M) | 1 SD | 2 SD | L | S | -2 SD | -1 SD | 0 SD (M) | 1 SD | 2 SD |
| 5.0 | 2.076 | 0.085 | 0.972 | 1.092 | 1.199 | 1.297 | 1.388 | 1.879 | 0.089 | 0.946 | 1.066 | 1.174 | 1.275 | 1.369 |
| 5.1 | 2.066 | 0.085 | 0.973 | 1.093 | 1.200 | 1.298 | 1.388 | 1.870 | 0.089 | 0.947 | 1.066 | 1.174 | 1.274 | 1.368 |
| 5.2 | 2.056 | 0.085 | 0.974 | 1.093 | 1.200 | 1.298 | 1.388 | 1.862 | 0.088 | 0.947 | 1.066 | 1.174 | 1.274 | 1.367 |
| 5.3 | 2.045 | 0.085 | 0.974 | 1.094 | 1.200 | 1.298 | 1.389 | 1.853 | 0.088 | 0.948 | 1.066 | 1.173 | 1.273 | 1.367 |
| 5.4 | 2.035 | 0.085 | 0.975 | 1.094 | 1.201 | 1.299 | 1.389 | 1.844 | 0.088 | 0.948 | 1.066 | 1.173 | 1.273 | 1.366 |
| 5.5 | 2.025 | 0.085 | 0.976 | 1.094 | 1.201 | 1.299 | 1.390 | 1.835 | 0.088 | 0.949 | 1.066 | 1.173 | 1.272 | 1.366 |
| 5.6 | 2.014 | 0.085 | 0.976 | 1.095 | 1.201 | 1.299 | 1.390 | 1.827 | 0.088 | 0.949 | 1.066 | 1.172 | 1.272 | 1.365 |
| 5.7 | 2.004 | 0.085 | 0.977 | 1.095 | 1.202 | 1.300 | 1.390 | 1.818 | 0.087 | 0.950 | 1.066 | 1.172 | 1.271 | 1.364 |
| 5.8 | 1.994 | 0.085 | 0.978 | 1.096 | 1.202 | 1.300 | 1.391 | 1.809 | 0.087 | 0.950 | 1.066 | 1.172 | 1.271 | 1.364 |
| 5.9 | 1.983 | 0.085 | 0.978 | 1.096 | 1.202 | 1.300 | 1.391 | 1.800 | 0.087 | 0.951 | 1.066 | 1.172 | 1.270 | 1.363 |
| 6.0 | 1.973 | 0.085 | 0.979 | 1.096 | 1.202 | 1.300 | 1.391 | 1.792 | 0.087 | 0.951 | 1.066 | 1.171 | 1.270 | 1.363 |
| 6.1 | 1.962 | 0.084 | 0.979 | 1.097 | 1.203 | 1.300 | 1.391 | 1.783 | 0.087 | 0.952 | 1.066 | 1.171 | 1.269 | 1.362 |
| 6.2 | 1.952 | 0.084 | 0.980 | 1.097 | 1.203 | 1.300 | 1.392 | 1.774 | 0.087 | 0.952 | 1.066 | 1.171 | 1.269 | 1.362 |
| 6.3 | 1.942 | 0.084 | 0.981 | 1.097 | 1.203 | 1.301 | 1.392 | 1.765 | 0.086 | 0.953 | 1.066 | 1.171 | 1.269 | 1.361 |
| 6.4 | 1.931 | 0.084 | 0.981 | 1.097 | 1.203 | 1.301 | 1.392 | 1.757 | 0.086 | 0.953 | 1.066 | 1.170 | 1.268 | 1.360 |
| 6.5 | 1.921 | 0.084 | 0.982 | 1.097 | 1.203 | 1.301 | 1.392 | 1.748 | 0.086 | 0.954 | 1.066 | 1.170 | 1.268 | 1.360 |
| 6.6 | 1.911 | 0.084 | 0.982 | 1.098 | 1.203 | 1.301 | 1.392 | 1.739 | 0.086 | 0.954 | 1.066 | 1.170 | 1.267 | 1.359 |
| 6.7 | 1.900 | 0.084 | 0.983 | 1.098 | 1.203 | 1.301 | 1.392 | 1.730 | 0.086 | 0.955 | 1.066 | 1.170 | 1.267 | 1.359 |
| 6.8 | 1.890 | 0.084 | 0.983 | 1.098 | 1.203 | 1.301 | 1.392 | 1.722 | 0.085 | 0.955 | 1.066 | 1.169 | 1.266 | 1.358 |
| 6.9 | 1.880 | 0.084 | 0.983 | 1.098 | 1.203 | 1.301 | 1.392 | 1.713 | 0.085 | 0.956 | 1.066 | 1.169 | 1.266 | 1.358 |
| 7.0 | 1.869 | 0.084 | 0.984 | 1.098 | 1.203 | 1.300 | 1.392 | 1.704 | 0.085 | 0.956 | 1.066 | 1.169 | 1.266 | 1.357 |
| 7.1 | 1.859 | 0.084 | 0.984 | 1.098 | 1.203 | 1.300 | 1.392 | 1.695 | 0.085 | 0.957 | 1.067 | 1.169 | 1.265 | 1.357 |
| 7.2 | 1.849 | 0.084 | 0.985 | 1.098 | 1.203 | 1.300 | 1.392 | 1.687 | 0.085 | 0.957 | 1.067 | 1.169 | 1.265 | 1.356 |
| 7.3 | 1.838 | 0.084 | 0.985 | 1.098 | 1.203 | 1.300 | 1.391 | 1.678 | 0.085 | 0.958 | 1.067 | 1.168 | 1.265 | 1.356 |
| 7.4 | 1.828 | 0.083 | 0.985 | 1.099 | 1.203 | 1.300 | 1.391 | 1.669 | 0.084 | 0.958 | 1.067 | 1.168 | 1.264 | 1.355 |
| 7.5 | 1.818 | 0.083 | 0.986 | 1.099 | 1.202 | 1.300 | 1.391 | 1.660 | 0.084 | 0.959 | 1.067 | 1.168 | 1.264 | 1.355 |
| 7.6 | 1.807 | 0.083 | 0.986 | 1.099 | 1.202 | 1.299 | 1.391 | 1.652 | 0.084 | 0.959 | 1.067 | 1.168 | 1.263 | 1.354 |
| 7.7 | 1.797 | 0.083 | 0.987 | 1.099 | 1.202 | 1.299 | 1.391 | 1.643 | 0.084 | 0.960 | 1.067 | 1.168 | 1.263 | 1.354 |
| 7.8 | 1.786 | 0.083 | 0.987 | 1.099 | 1.202 | 1.299 | 1.390 | 1.634 | 0.084 | 0.960 | 1.067 | 1.167 | 1.263 | 1.353 |
| 7.9 | 1.776 | 0.083 | 0.987 | 1.099 | 1.202 | 1.299 | 1.390 | 1.625 | 0.083 | 0.961 | 1.067 | 1.167 | 1.262 | 1.353 |
| 8.0 | 1.766 | 0.083 | 0.988 | 1.099 | 1.202 | 1.298 | 1.390 | 1.617 | 0.083 | 0.961 | 1.067 | 1.167 | 1.262 | 1.353 |
| 8.1 | 1.755 | 0.083 | 0.988 | 1.099 | 1.201 | 1.298 | 1.389 | 1.608 | 0.083 | 0.962 | 1.067 | 1.167 | 1.262 | 1.352 |
| 8.2 | 1.745 | 0.083 | 0.988 | 1.099 | 1.201 | 1.298 | 1.389 | 1.599 | 0.083 | 0.962 | 1.068 | 1.167 | 1.261 | 1.352 |
| 8.3 | 1.735 | 0.083 | 0.989 | 1.099 | 1.201 | 1.297 | 1.389 | 1.590 | 0.083 | 0.963 | 1.068 | 1.167 | 1.261 | 1.352 |
| 8.4 | 1.724 | 0.083 | 0.989 | 1.099 | 1.201 | 1.297 | 1.389 | 1.582 | 0.083 | 0.964 | 1.068 | 1.167 | 1.261 | 1.351 |
| 8.5 | 1.714 | 0.082 | 0.989 | 1.099 | 1.201 | 1.297 | 1.388 | 1.573 | 0.082 | 0.964 | 1.069 | 1.167 | 1.261 | 1.351 |
| 8.6 | 1.704 | 0.082 | 0.990 | 1.099 | 1.201 | 1.297 | 1.388 | 1.564 | 0.082 | 0.965 | 1.069 | 1.167 | 1.261 | 1.351 |
| 8.7 | 1.693 | 0.082 | 0.990 | 1.099 | 1.200 | 1.296 | 1.388 | 1.555 | 0.082 | 0.966 | 1.069 | 1.167 | 1.261 | 1.351 |
| 8.8 | 1.683 | 0.082 | 0.990 | 1.099 | 1.200 | 1.296 | 1.387 | 1.547 | 0.082 | 0.967 | 1.070 | 1.168 | 1.261 | 1.351 |
| 8.9 | 1.673 | 0.082 | 0.991 | 1.099 | 1.200 | 1.296 | 1.387 | 1.538 | 0.082 | 0.968 | 1.071 | 1.168 | 1.262 | 1.351 |
| 9.0 | 1.662 | 0.082 | 0.991 | 1.099 | 1.200 | 1.296 | 1.387 | 1.529 | 0.082 | 0.969 | 1.071 | 1.169 | 1.262 | 1.352 |
| 9.1 | 1.652 | 0.082 | 0.991 | 1.099 | 1.200 | 1.295 | 1.387 | 1.520 | 0.081 | 0.970 | 1.072 | 1.169 | 1.262 | 1.352 |
| 9.2 | 1.642 | 0.082 | 0.992 | 1.099 | 1.200 | 1.295 | 1.387 | 1.512 | 0.081 | 0.971 | 1.073 | 1.170 | 1.263 | 1.353 |
| 9.3 | 1.631 | 0.082 | 0.992 | 1.099 | 1.199 | 1.295 | 1.386 | 1.503 | 0.081 | 0.972 | 1.074 | 1.171 | 1.263 | 1.353 |
| 9.4 | 1.621 | 0.082 | 0.992 | 1.099 | 1.199 | 1.295 | 1.386 | 1.494 | 0.081 | 0.974 | 1.075 | 1.171 | 1.264 | 1.354 |
| 9.5 | 1.610 | 0.082 | 0.993 | 1.099 | 1.199 | 1.295 | 1.386 | 1.486 | 0.081 | 0.975 | 1.076 | 1.172 | 1.265 | 1.355 |
| 9.6 | 1.600 | 0.081 | 0.993 | 1.099 | 1.199 | 1.295 | 1.386 | 1.477 | 0.080 | 0.976 | 1.077 | 1.173 | 1.266 | 1.356 |
| 9.7 | 1.590 | 0.081 | 0.993 | 1.099 | 1.199 | 1.295 | 1.386 | 1.468 | 0.080 | 0.978 | 1.078 | 1.175 | 1.267 | 1.357 |
| 9.8 | 1.579 | 0.081 | 0.994 | 1.099 | 1.199 | 1.294 | 1.386 | 1.459 | 0.080 | 0.980 | 1.080 | 1.176 | 1.268 | 1.358 |
| 9.9 | 1.569 | 0.081 | 0.994 | 1.099 | 1.199 | 1.294 | 1.386 | 1.451 | 0.080 | 0.981 | 1.081 | 1.177 | 1.270 | 1.359 |
| 10.0 | 1.559 | 0.081 | 0.995 | 1.100 | 1.199 | 1.294 | 1.386 | 1.442 | 0.080 | 0.983 | 1.083 | 1.179 | 1.271 | 1.361 |
| 10.1 | 1.548 | 0.081 | 0.995 | 1.100 | 1.199 | 1.294 | 1.386 | 1.433 | 0.080 | 0.985 | 1.085 | 1.180 | 1.273 | 1.362 |
| 10.2 | 1.538 | 0.081 | 0.995 | 1.100 | 1.199 | 1.295 | 1.386 | 1.424 | 0.079 | 0.987 | 1.086 | 1.182 | 1.274 | 1.364 |
| 10.3 | 1.528 | 0.081 | 0.996 | 1.100 | 1.200 | 1.295 | 1.386 | 1.416 | 0.079 | 0.989 | 1.088 | 1.184 | 1.276 | 1.366 |
| 10.4 | 1.517 | 0.081 | 0.996 | 1.101 | 1.200 | 1.295 | 1.386 | 1.407 | 0.079 | 0.992 | 1.091 | 1.186 | 1.278 | 1.368 |
| 10.5 | 1.507 | 0.081 | 0.997 | 1.101 | 1.200 | 1.295 | 1.387 | 1.398 | 0.079 | 0.994 | 1.093 | 1.188 | 1.280 | 1.370 |
| 10.6 | 1.497 | 0.081 | 0.998 | 1.101 | 1.200 | 1.295 | 1.387 | 1.389 | 0.079 | 0.996 | 1.095 | 1.190 | 1.283 | 1.373 |
| 10.7 | 1.486 | 0.081 | 0.998 | 1.102 | 1.201 | 1.296 | 1.387 | 1.381 | 0.079 | 0.999 | 1.098 | 1.193 | 1.285 | 1.375 |
| 10.8 | 1.476 | 0.081 | 0.999 | 1.102 | 1.201 | 1.296 | 1.388 | 1.372 | 0.078 | 1.002 | 1.100 | 1.195 | 1.288 | 1.378 |
| 10.9 | 1.466 | 0.081 | 1.000 | 1.103 | 1.201 | 1.296 | 1.388 | 1.363 | 0.078 | 1.005 | 1.103 | 1.198 | 1.291 | 1.381 |
| 11.0 | 1.455 | 0.080 | 1.001 | 1.103 | 1.202 | 1.297 | 1.389 | 1.354 | 0.078 | 1.008 | 1.106 | 1.201 | 1.294 | 1.384 |
| 11.1 | 1.445 | 0.080 | 1.001 | 1.104 | 1.202 | 1.297 | 1.389 | 1.346 | 0.078 | 1.011 | 1.109 | 1.204 | 1.297 | 1.387 |
| 11.2 | 1.434 | 0.080 | 1.002 | 1.105 | 1.203 | 1.298 | 1.390 | 1.337 | 0.078 | 1.014 | 1.112 | 1.207 | 1.300 | 1.391 |
| 11.3 | 1.424 | 0.080 | 1.003 | 1.105 | 1.203 | 1.298 | 1.390 | 1.328 | 0.078 | 1.018 | 1.115 | 1.211 | 1.303 | 1.394 |
| 11.4 | 1.414 | 0.080 | 1.004 | 1.106 | 1.204 | 1.299 | 1.391 | 1.319 | 0.077 | 1.021 | 1.119 | 1.214 | 1.307 | 1.398 |
| 11.5 | 1.403 | 0.080 | 1.005 | 1.107 | 1.205 | 1.300 | 1.392 | 1.311 | 0.077 | 1.025 | 1.123 | 1.218 | 1.311 | 1.402 |
| 11.6 | 1.393 | 0.080 | 1.006 | 1.108 | 1.206 | 1.300 | 1.393 | 1.302 | 0.077 | 1.028 | 1.126 | 1.222 | 1.315 | 1.406 |
| 11.7 | 1.383 | 0.080 | 1.007 | 1.109 | 1.206 | 1.301 | 1.393 | 1.293 | 0.077 | 1.032 | 1.130 | 1.226 | 1.319 | 1.410 |
| 11.8 | 1.372 | 0.080 | 1.009 | 1.110 | 1.207 | 1.302 | 1.394 | 1.284 | 0.077 | 1.036 | 1.134 | 1.230 | 1.323 | 1.415 |
| 11.9 | 1.362 | 0.079 | 1.010 | 1.111 | 1.208 | 1.303 | 1.395 | 1.276 | 0.077 | 1.040 | 1.138 | 1.234 | 1.327 | 1.419 |
| 12.0 | 1.352 | 0.079 | 1.012 | 1.112 | 1.209 | 1.304 | 1.396 | 1.267 | 0.076 | 1.045 | 1.142 | 1.238 | 1.332 | 1.424 |
| 12.1 | 1.341 | 0.079 | 1.013 | 1.113 | 1.211 | 1.305 | 1.398 | 1.258 | 0.076 | 1.049 | 1.147 | 1.242 | 1.336 | 1.429 |
| 12.2 | 1.331 | 0.079 | 1.015 | 1.115 | 1.212 | 1.306 | 1.399 | 1.249 | 0.076 | 1.053 | 1.151 | 1.247 | 1.341 | 1.433 |
| 12.3 | 1.321 | 0.079 | 1.016 | 1.116 | 1.213 | 1.308 | 1.400 | 1.241 | 0.076 | 1.057 | 1.155 | 1.251 | 1.346 | 1.438 |
| 12.4 | 1.310 | 0.079 | 1.018 | 1.118 | 1.215 | 1.309 | 1.402 | 1.232 | 0.076 | 1.062 | 1.160 | 1.256 | 1.350 | 1.443 |
| 12.5 | 1.300 | 0.079 | 1.020 | 1.120 | 1.216 | 1.311 | 1.403 | 1.223 | 0.076 | 1.066 | 1.164 | 1.260 | 1.355 | 1.448 |
| 12.6 | 1.290 | 0.078 | 1.022 | 1.122 | 1.218 | 1.313 | 1.405 | 1.214 | 0.075 | 1.071 | 1.169 | 1.265 | 1.360 | 1.453 |
| 12.7 | 1.279 | 0.078 | 1.025 | 1.124 | 1.220 | 1.314 | 1.407 | 1.206 | 0.075 | 1.075 | 1.173 | 1.269 | 1.364 | 1.458 |
| 12.8 | 1.269 | 0.078 | 1.027 | 1.126 | 1.222 | 1.316 | 1.409 | 1.197 | 0.075 | 1.079 | 1.177 | 1.274 | 1.369 | 1.463 |
| 12.9 | 1.258 | 0.078 | 1.030 | 1.128 | 1.224 | 1.318 | 1.411 | 1.188 | 0.075 | 1.084 | 1.182 | 1.278 | 1.374 | 1.468 |
| 13.0 | 1.248 | 0.078 | 1.032 | 1.130 | 1.226 | 1.321 | 1.413 | 1.179 | 0.075 | 1.088 | 1.186 | 1.283 | 1.378 | 1.472 |
| 13.1 | 1.238 | 0.077 | 1.035 | 1.133 | 1.229 | 1.323 | 1.415 | 1.171 | 0.075 | 1.092 | 1.190 | 1.287 | 1.383 | 1.477 |
| 13.2 | 1.227 | 0.077 | 1.038 | 1.135 | 1.231 | 1.325 | 1.418 | 1.162 | 0.075 | 1.096 | 1.194 | 1.291 | 1.387 | 1.482 |
| 13.3 | 1.217 | 0.077 | 1.041 | 1.138 | 1.234 | 1.328 | 1.420 | 1.153 | 0.074 | 1.100 | 1.199 | 1.295 | 1.391 | 1.486 |
| 13.4 | 1.207 | 0.077 | 1.044 | 1.141 | 1.237 | 1.330 | 1.423 | 1.144 | 0.074 | 1.104 | 1.202 | 1.299 | 1.395 | 1.490 |
| 13.5 | 1.196 | 0.076 | 1.047 | 1.144 | 1.239 | 1.333 | 1.426 | 1.136 | 0.074 | 1.108 | 1.206 | 1.303 | 1.399 | 1.494 |
| 13.6 | 1.186 | 0.076 | 1.051 | 1.147 | 1.242 | 1.336 | 1.429 | 1.127 | 0.074 | 1.112 | 1.210 | 1.307 | 1.403 | 1.498 |
| 13.7 | 1.176 | 0.076 | 1.054 | 1.150 | 1.245 | 1.339 | 1.432 | 1.118 | 0.074 | 1.116 | 1.214 | 1.311 | 1.407 | 1.502 |
| 13.8 | 1.165 | 0.075 | 1.058 | 1.154 | 1.248 | 1.342 | 1.435 | 1.109 | 0.074 | 1.119 | 1.217 | 1.314 | 1.410 | 1.506 |
| 13.9 | 1.155 | 0.075 | 1.061 | 1.157 | 1.252 | 1.345 | 1.438 | 1.101 | 0.073 | 1.122 | 1.220 | 1.317 | 1.414 | 1.509 |
| 14.0 | 1.145 | 0.075 | 1.065 | 1.161 | 1.255 | 1.348 | 1.441 | 1.092 | 0.073 | 1.126 | 1.224 | 1.321 | 1.417 | 1.513 |
| 14.1 | 1.134 | 0.075 | 1.069 | 1.164 | 1.258 | 1.352 | 1.444 | 1.083 | 0.073 | 1.129 | 1.227 | 1.324 | 1.420 | 1.516 |
| 14.2 | 1.124 | 0.074 | 1.073 | 1.168 | 1.262 | 1.355 | 1.447 | 1.074 | 0.073 | 1.132 | 1.229 | 1.326 | 1.423 | 1.519 |
| 14.3 | 1.113 | 0.074 | 1.076 | 1.171 | 1.265 | 1.358 | 1.450 | 1.066 | 0.073 | 1.135 | 1.232 | 1.329 | 1.426 | 1.522 |
| 14.4 | 1.103 | 0.074 | 1.080 | 1.175 | 1.268 | 1.361 | 1.454 | 1.057 | 0.073 | 1.138 | 1.235 | 1.332 | 1.428 | 1.525 |
| 14.5 | 1.093 | 0.073 | 1.084 | 1.178 | 1.272 | 1.365 | 1.457 | 1.048 | 0.072 | 1.140 | 1.237 | 1.334 | 1.431 | 1.527 |
| 14.6 | 1.082 | 0.073 | 1.088 | 1.182 | 1.275 | 1.368 | 1.460 | 1.039 | 0.072 | 1.143 | 1.240 | 1.337 | 1.433 | 1.529 |
| 14.7 | 1.072 | 0.072 | 1.092 | 1.186 | 1.279 | 1.371 | 1.463 | 1.031 | 0.072 | 1.145 | 1.242 | 1.339 | 1.435 | 1.532 |
| 14.8 | 1.062 | 0.072 | 1.097 | 1.190 | 1.282 | 1.375 | 1.466 | 1.022 | 0.072 | 1.148 | 1.244 | 1.341 | 1.437 | 1.534 |
| 14.9 | 1.051 | 0.072 | 1.101 | 1.193 | 1.286 | 1.378 | 1.470 | 1.013 | 0.072 | 1.150 | 1.246 | 1.343 | 1.439 | 1.536 |
| 15.0 | 1.041 | 0.071 | 1.105 | 1.197 | 1.289 | 1.381 | 1.473 | 1.005 | 0.072 | 1.152 | 1.248 | 1.345 | 1.441 | 1.538 |
| 15.1 | 1.031 | 0.071 | 1.109 | 1.201 | 1.293 | 1.384 | 1.476 | 0.996 | 0.072 | 1.154 | 1.250 | 1.347 | 1.443 | 1.539 |
| 15.2 | 1.020 | 0.071 | 1.113 | 1.205 | 1.296 | 1.388 | 1.479 | 0.987 | 0.071 | 1.156 | 1.252 | 1.348 | 1.445 | 1.541 |
| 15.3 | 1.010 | 0.070 | 1.117 | 1.208 | 1.300 | 1.391 | 1.482 | 0.978 | 0.071 | 1.158 | 1.254 | 1.350 | 1.446 | 1.543 |
| 15.4 | 1.000 | 0.070 | 1.121 | 1.212 | 1.303 | 1.394 | 1.485 | 0.970 | 0.071 | 1.160 | 1.256 | 1.352 | 1.448 | 1.544 |
| 15.5 | 0.989 | 0.069 | 1.125 | 1.216 | 1.306 | 1.397 | 1.488 | 0.961 | 0.071 | 1.162 | 1.257 | 1.353 | 1.449 | 1.546 |
| 15.6 | 0.979 | 0.069 | 1.129 | 1.219 | 1.310 | 1.400 | 1.491 | 0.952 | 0.071 | 1.163 | 1.259 | 1.355 | 1.451 | 1.547 |
| 15.7 | 0.969 | 0.069 | 1.133 | 1.223 | 1.313 | 1.403 | 1.494 | 0.943 | 0.071 | 1.165 | 1.260 | 1.356 | 1.452 | 1.548 |
| 15.8 | 0.958 | 0.068 | 1.137 | 1.226 | 1.316 | 1.406 | 1.496 | 0.935 | 0.070 | 1.167 | 1.262 | 1.357 | 1.453 | 1.549 |
| 15.9 | 0.948 | 0.068 | 1.141 | 1.230 | 1.319 | 1.409 | 1.499 | 0.926 | 0.070 | 1.168 | 1.263 | 1.358 | 1.454 | 1.550 |
| 16.0 | 0.937 | 0.068 | 1.145 | 1.233 | 1.322 | 1.412 | 1.502 | 0.917 | 0.070 | 1.170 | 1.264 | 1.360 | 1.455 | 1.552 |
| 16.1 | 0.927 | 0.067 | 1.148 | 1.237 | 1.325 | 1.415 | 1.504 | 0.908 | 0.070 | 1.171 | 1.266 | 1.361 | 1.456 | 1.553 |
| 16.2 | 0.917 | 0.067 | 1.152 | 1.240 | 1.328 | 1.417 | 1.507 | 0.900 | 0.070 | 1.173 | 1.267 | 1.362 | 1.457 | 1.554 |
| 16.3 | 0.906 | 0.066 | 1.156 | 1.243 | 1.331 | 1.420 | 1.509 | 0.891 | 0.070 | 1.174 | 1.268 | 1.363 | 1.458 | 1.555 |
| 16.4 | 0.896 | 0.066 | 1.159 | 1.246 | 1.334 | 1.422 | 1.511 | 0.882 | 0.070 | 1.176 | 1.270 | 1.364 | 1.459 | 1.555 |
| 16.5 | 0.886 | 0.066 | 1.163 | 1.249 | 1.337 | 1.425 | 1.514 | 0.873 | 0.069 | 1.177 | 1.271 | 1.365 | 1.460 | 1.556 |
| 16.6 | 0.875 | 0.065 | 1.166 | 1.252 | 1.339 | 1.427 | 1.516 | 0.865 | 0.069 | 1.179 | 1.272 | 1.366 | 1.461 | 1.557 |
| 16.7 | 0.865 | 0.065 | 1.169 | 1.255 | 1.342 | 1.430 | 1.518 | 0.856 | 0.069 | 1.180 | 1.273 | 1.367 | 1.462 | 1.558 |
| 16.8 | 0.855 | 0.065 | 1.172 | 1.258 | 1.344 | 1.432 | 1.520 | 0.847 | 0.069 | 1.181 | 1.274 | 1.368 | 1.463 | 1.559 |
| 16.9 | 0.844 | 0.064 | 1.175 | 1.261 | 1.347 | 1.434 | 1.522 | 0.838 | 0.069 | 1.183 | 1.275 | 1.369 | 1.464 | 1.560 |
| 17.0 | 0.834 | 0.064 | 1.178 | 1.263 | 1.349 | 1.436 | 1.523 | 0.830 | 0.069 | 1.184 | 1.276 | 1.370 | 1.465 | 1.560 |
| 17.1 | 0.824 | 0.064 | 1.181 | 1.266 | 1.351 | 1.438 | 1.525 | 0.821 | 0.069 | 1.185 | 1.277 | 1.371 | 1.465 | 1.561 |
| 17.2 | 0.813 | 0.063 | 1.184 | 1.268 | 1.353 | 1.440 | 1.527 | 0.812 | 0.068 | 1.186 | 1.278 | 1.372 | 1.466 | 1.562 |
| 17.3 | 0.803 | 0.063 | 1.186 | 1.270 | 1.355 | 1.441 | 1.528 | 0.803 | 0.068 | 1.188 | 1.279 | 1.372 | 1.467 | 1.562 |
| 17.4 | 0.793 | 0.063 | 1.189 | 1.272 | 1.357 | 1.443 | 1.530 | 0.795 | 0.068 | 1.189 | 1.280 | 1.373 | 1.468 | 1.563 |
| 17.5 | 0.782 | 0.063 | 1.191 | 1.274 | 1.359 | 1.444 | 1.531 | 0.786 | 0.068 | 1.190 | 1.281 | 1.374 | 1.468 | 1.564 |
| 17.6 | 0.772 | 0.062 | 1.194 | 1.276 | 1.360 | 1.446 | 1.532 | 0.777 | 0.068 | 1.191 | 1.282 | 1.375 | 1.469 | 1.564 |
| 17.7 | 0.761 | 0.062 | 1.196 | 1.278 | 1.362 | 1.447 | 1.533 | 0.768 | 0.068 | 1.192 | 1.283 | 1.376 | 1.469 | 1.565 |
| 17.8 | 0.751 | 0.062 | 1.198 | 1.280 | 1.363 | 1.448 | 1.534 | 0.760 | 0.068 | 1.193 | 1.284 | 1.376 | 1.470 | 1.565 |
| 17.9 | 0.741 | 0.062 | 1.200 | 1.282 | 1.365 | 1.449 | 1.535 | 0.751 | 0.067 | 1.195 | 1.285 | 1.377 | 1.471 | 1.566 |
| 18.0 | 0.730 | 0.061 | 1.202 | 1.283 | 1.366 | 1.451 | 1.536 | 0.742 | 0.067 | 1.196 | 1.286 | 1.378 | 1.471 | 1.566 |
| 18.1 | 0.720 | 0.061 | 1.203 | 1.285 | 1.367 | 1.452 | 1.537 | 0.733 | 0.067 | 1.197 | 1.287 | 1.378 | 1.471 | 1.566 |
| 18.2 | 0.710 | 0.061 | 1.205 | 1.286 | 1.369 | 1.453 | 1.538 | 0.725 | 0.067 | 1.198 | 1.287 | 1.379 | 1.472 | 1.567 |
| 18.3 | 0.699 | 0.061 | 1.207 | 1.287 | 1.370 | 1.454 | 1.539 | 0.716 | 0.067 | 1.198 | 1.288 | 1.379 | 1.472 | 1.567 |
| 18.4 | 0.689 | 0.060 | 1.208 | 1.289 | 1.371 | 1.454 | 1.540 | 0.707 | 0.067 | 1.199 | 1.289 | 1.380 | 1.472 | 1.567 |
| 18.5 | 0.679 | 0.060 | 1.210 | 1.290 | 1.372 | 1.455 | 1.540 | 0.698 | 0.067 | 1.200 | 1.289 | 1.380 | 1.473 | 1.567 |
| 18.6 | 0.668 | 0.060 | 1.211 | 1.291 | 1.373 | 1.456 | 1.541 | 0.690 | 0.066 | 1.201 | 1.290 | 1.380 | 1.473 | 1.567 |
| 18.7 | 0.658 | 0.060 | 1.212 | 1.292 | 1.374 | 1.457 | 1.542 | 0.681 | 0.066 | 1.202 | 1.290 | 1.381 | 1.473 | 1.567 |
| 18.8 | 0.648 | 0.060 | 1.214 | 1.293 | 1.374 | 1.458 | 1.542 | 0.672 | 0.066 | 1.202 | 1.291 | 1.381 | 1.473 | 1.567 |
| 18.9 | 0.637 | 0.060 | 1.215 | 1.294 | 1.375 | 1.458 | 1.543 | 0.663 | 0.066 | 1.203 | 1.291 | 1.381 | 1.473 | 1.567 |
| 19.0 | 0.627 | 0.060 | 1.216 | 1.295 | 1.376 | 1.459 | 1.544 | 0.655 | 0.066 | 1.204 | 1.291 | 1.381 | 1.473 | 1.567 |
| 19.1 | 0.617 | 0.059 | 1.217 | 1.296 | 1.377 | 1.460 | 1.544 | 0.646 | 0.066 | 1.204 | 1.292 | 1.382 | 1.473 | 1.567 |
| 19.2 | 0.606 | 0.059 | 1.218 | 1.297 | 1.378 | 1.460 | 1.545 | 0.637 | 0.066 | 1.205 | 1.292 | 1.382 | 1.473 | 1.567 |
| 19.3 | 0.596 | 0.059 | 1.219 | 1.298 | 1.379 | 1.461 | 1.546 | 0.628 | 0.065 | 1.205 | 1.292 | 1.382 | 1.473 | 1.567 |
| 19.4 | 0.585 | 0.059 | 1.221 | 1.299 | 1.379 | 1.462 | 1.546 | 0.620 | 0.065 | 1.206 | 1.293 | 1.382 | 1.473 | 1.567 |
| 19.5 | 0.575 | 0.059 | 1.222 | 1.300 | 1.380 | 1.462 | 1.547 | 0.611 | 0.065 | 1.207 | 1.293 | 1.382 | 1.473 | 1.566 |
| 19.6 | 0.565 | 0.059 | 1.223 | 1.301 | 1.381 | 1.463 | 1.547 | 0.602 | 0.065 | 1.207 | 1.293 | 1.382 | 1.473 | 1.566 |
| 19.7 | 0.554 | 0.059 | 1.224 | 1.302 | 1.382 | 1.464 | 1.548 | 0.593 | 0.065 | 1.208 | 1.294 | 1.382 | 1.473 | 1.566 |
| 19.8 | 0.544 | 0.059 | 1.225 | 1.302 | 1.382 | 1.464 | 1.549 | 0.585 | 0.065 | 1.208 | 1.294 | 1.382 | 1.473 | 1.566 |
| 19.9 | 0.534 | 0.059 | 1.226 | 1.303 | 1.383 | 1.465 | 1.549 | 0.576 | 0.065 | 1.208 | 1.294 | 1.382 | 1.472 | 1.565 |
| 20.0 | 0.523 | 0.058 | 1.226 | 1.304 | 1.384 | 1.466 | 1.550 | 0.567 | 0.064 | 1.209 | 1.294 | 1.382 | 1.472 | 1.565 |

| L2 TBS | | | | | | | | | | | | | | |
| --- | --- | --- | --- | --- | --- | --- | --- | --- | --- | --- | --- | --- | --- | --- |
|  | Males | | | | | | | Females | | | | | | |
| Age | L | S | -2 SD | -1 SD | 0 SD (M) | 1 SD | 2 SD | L | S | -2 SD | -1 SD | 0 SD (M) | 1 SD | 2 SD |
| 5.0 | 2.895 | 0.075 | 1.031 | 1.153 | 1.255 | 1.342 | 1.421 | 1.957 | 0.064 | 1.062 | 1.149 | 1.230 | 1.307 | 1.379 |
| 5.1 | 2.871 | 0.075 | 1.033 | 1.154 | 1.256 | 1.343 | 1.422 | 1.987 | 0.064 | 1.061 | 1.149 | 1.231 | 1.308 | 1.381 |
| 5.2 | 2.847 | 0.075 | 1.035 | 1.155 | 1.256 | 1.344 | 1.423 | 2.017 | 0.065 | 1.060 | 1.149 | 1.232 | 1.309 | 1.382 |
| 5.3 | 2.823 | 0.074 | 1.036 | 1.156 | 1.257 | 1.345 | 1.424 | 2.046 | 0.065 | 1.060 | 1.150 | 1.233 | 1.310 | 1.383 |
| 5.4 | 2.799 | 0.074 | 1.038 | 1.158 | 1.258 | 1.346 | 1.425 | 2.076 | 0.065 | 1.059 | 1.150 | 1.233 | 1.311 | 1.384 |
| 5.5 | 2.775 | 0.074 | 1.040 | 1.159 | 1.259 | 1.347 | 1.426 | 2.105 | 0.066 | 1.058 | 1.150 | 1.234 | 1.312 | 1.385 |
| 5.6 | 2.752 | 0.074 | 1.041 | 1.160 | 1.260 | 1.348 | 1.427 | 2.135 | 0.066 | 1.058 | 1.150 | 1.235 | 1.313 | 1.386 |
| 5.7 | 2.728 | 0.074 | 1.043 | 1.161 | 1.261 | 1.349 | 1.428 | 2.164 | 0.066 | 1.057 | 1.150 | 1.235 | 1.314 | 1.388 |
| 5.8 | 2.704 | 0.074 | 1.044 | 1.162 | 1.262 | 1.350 | 1.429 | 2.193 | 0.066 | 1.056 | 1.150 | 1.236 | 1.315 | 1.389 |
| 5.9 | 2.680 | 0.074 | 1.046 | 1.163 | 1.263 | 1.351 | 1.431 | 2.221 | 0.067 | 1.056 | 1.150 | 1.236 | 1.316 | 1.390 |
| 6.0 | 2.656 | 0.074 | 1.047 | 1.164 | 1.264 | 1.352 | 1.432 | 2.249 | 0.067 | 1.055 | 1.150 | 1.237 | 1.317 | 1.391 |
| 6.1 | 2.633 | 0.074 | 1.049 | 1.165 | 1.265 | 1.353 | 1.433 | 2.277 | 0.067 | 1.054 | 1.150 | 1.237 | 1.317 | 1.391 |
| 6.2 | 2.609 | 0.074 | 1.050 | 1.166 | 1.266 | 1.354 | 1.434 | 2.303 | 0.068 | 1.053 | 1.150 | 1.238 | 1.318 | 1.392 |
| 6.3 | 2.585 | 0.074 | 1.052 | 1.167 | 1.266 | 1.355 | 1.435 | 2.329 | 0.068 | 1.052 | 1.150 | 1.238 | 1.319 | 1.393 |
| 6.4 | 2.561 | 0.074 | 1.053 | 1.168 | 1.267 | 1.356 | 1.436 | 2.353 | 0.068 | 1.051 | 1.150 | 1.239 | 1.319 | 1.394 |
| 6.5 | 2.538 | 0.074 | 1.054 | 1.169 | 1.268 | 1.357 | 1.437 | 2.376 | 0.068 | 1.051 | 1.150 | 1.239 | 1.320 | 1.395 |
| 6.6 | 2.514 | 0.074 | 1.056 | 1.170 | 1.269 | 1.358 | 1.438 | 2.397 | 0.069 | 1.050 | 1.150 | 1.239 | 1.321 | 1.395 |
| 6.7 | 2.490 | 0.074 | 1.057 | 1.171 | 1.270 | 1.359 | 1.440 | 2.416 | 0.069 | 1.049 | 1.150 | 1.240 | 1.321 | 1.396 |
| 6.8 | 2.467 | 0.074 | 1.058 | 1.172 | 1.271 | 1.360 | 1.441 | 2.433 | 0.069 | 1.048 | 1.150 | 1.240 | 1.322 | 1.397 |
| 6.9 | 2.443 | 0.074 | 1.060 | 1.173 | 1.271 | 1.360 | 1.442 | 2.448 | 0.069 | 1.047 | 1.150 | 1.240 | 1.322 | 1.397 |
| 7.0 | 2.420 | 0.073 | 1.061 | 1.173 | 1.272 | 1.361 | 1.443 | 2.461 | 0.069 | 1.047 | 1.150 | 1.241 | 1.323 | 1.398 |
| 7.1 | 2.396 | 0.073 | 1.062 | 1.174 | 1.273 | 1.362 | 1.444 | 2.471 | 0.070 | 1.046 | 1.150 | 1.241 | 1.323 | 1.399 |
| 7.2 | 2.373 | 0.073 | 1.063 | 1.175 | 1.274 | 1.363 | 1.445 | 2.478 | 0.070 | 1.046 | 1.149 | 1.241 | 1.324 | 1.399 |
| 7.3 | 2.350 | 0.073 | 1.065 | 1.176 | 1.274 | 1.364 | 1.446 | 2.482 | 0.070 | 1.045 | 1.149 | 1.241 | 1.324 | 1.400 |
| 7.4 | 2.326 | 0.073 | 1.066 | 1.177 | 1.275 | 1.364 | 1.446 | 2.482 | 0.070 | 1.045 | 1.149 | 1.242 | 1.325 | 1.400 |
| 7.5 | 2.303 | 0.073 | 1.067 | 1.177 | 1.276 | 1.365 | 1.447 | 2.479 | 0.070 | 1.044 | 1.149 | 1.242 | 1.325 | 1.401 |
| 7.6 | 2.281 | 0.073 | 1.068 | 1.178 | 1.276 | 1.366 | 1.448 | 2.472 | 0.071 | 1.044 | 1.149 | 1.242 | 1.326 | 1.402 |
| 7.7 | 2.258 | 0.073 | 1.069 | 1.179 | 1.277 | 1.366 | 1.449 | 2.462 | 0.071 | 1.044 | 1.150 | 1.242 | 1.326 | 1.403 |
| 7.8 | 2.235 | 0.073 | 1.070 | 1.179 | 1.277 | 1.367 | 1.450 | 2.448 | 0.071 | 1.044 | 1.150 | 1.243 | 1.327 | 1.403 |
| 7.9 | 2.213 | 0.073 | 1.071 | 1.180 | 1.278 | 1.367 | 1.450 | 2.431 | 0.071 | 1.044 | 1.150 | 1.243 | 1.327 | 1.404 |
| 8.0 | 2.190 | 0.073 | 1.072 | 1.180 | 1.278 | 1.368 | 1.451 | 2.410 | 0.071 | 1.045 | 1.150 | 1.243 | 1.328 | 1.405 |
| 8.1 | 2.168 | 0.073 | 1.073 | 1.181 | 1.279 | 1.368 | 1.452 | 2.386 | 0.071 | 1.045 | 1.150 | 1.244 | 1.328 | 1.406 |
| 8.2 | 2.146 | 0.073 | 1.073 | 1.181 | 1.279 | 1.369 | 1.452 | 2.359 | 0.071 | 1.046 | 1.151 | 1.244 | 1.329 | 1.407 |
| 8.3 | 2.124 | 0.073 | 1.074 | 1.182 | 1.279 | 1.369 | 1.453 | 2.329 | 0.071 | 1.046 | 1.151 | 1.245 | 1.330 | 1.408 |
| 8.4 | 2.102 | 0.073 | 1.075 | 1.182 | 1.280 | 1.370 | 1.454 | 2.296 | 0.071 | 1.047 | 1.152 | 1.245 | 1.330 | 1.409 |
| 8.5 | 2.081 | 0.073 | 1.076 | 1.183 | 1.280 | 1.370 | 1.454 | 2.260 | 0.072 | 1.048 | 1.152 | 1.246 | 1.331 | 1.410 |
| 8.6 | 2.059 | 0.073 | 1.077 | 1.183 | 1.280 | 1.370 | 1.455 | 2.222 | 0.072 | 1.049 | 1.153 | 1.246 | 1.332 | 1.411 |
| 8.7 | 2.038 | 0.073 | 1.077 | 1.183 | 1.281 | 1.371 | 1.455 | 2.182 | 0.072 | 1.050 | 1.154 | 1.247 | 1.333 | 1.413 |
| 8.8 | 2.016 | 0.073 | 1.078 | 1.184 | 1.281 | 1.371 | 1.455 | 2.140 | 0.072 | 1.052 | 1.154 | 1.248 | 1.334 | 1.414 |
| 8.9 | 1.995 | 0.073 | 1.079 | 1.184 | 1.281 | 1.371 | 1.456 | 2.097 | 0.072 | 1.053 | 1.155 | 1.249 | 1.335 | 1.415 |
| 9.0 | 1.974 | 0.073 | 1.079 | 1.184 | 1.281 | 1.371 | 1.456 | 2.054 | 0.072 | 1.054 | 1.156 | 1.249 | 1.336 | 1.417 |
| 9.1 | 1.953 | 0.073 | 1.080 | 1.185 | 1.281 | 1.372 | 1.456 | 2.009 | 0.072 | 1.056 | 1.157 | 1.250 | 1.337 | 1.418 |
| 9.2 | 1.933 | 0.073 | 1.081 | 1.185 | 1.282 | 1.372 | 1.457 | 1.965 | 0.072 | 1.058 | 1.158 | 1.251 | 1.338 | 1.420 |
| 9.3 | 1.912 | 0.073 | 1.081 | 1.185 | 1.282 | 1.372 | 1.457 | 1.922 | 0.072 | 1.059 | 1.160 | 1.252 | 1.339 | 1.421 |
| 9.4 | 1.891 | 0.073 | 1.082 | 1.186 | 1.282 | 1.372 | 1.457 | 1.879 | 0.072 | 1.061 | 1.161 | 1.254 | 1.341 | 1.423 |
| 9.5 | 1.871 | 0.073 | 1.082 | 1.186 | 1.282 | 1.372 | 1.458 | 1.837 | 0.071 | 1.063 | 1.162 | 1.255 | 1.342 | 1.425 |
| 9.6 | 1.850 | 0.073 | 1.083 | 1.186 | 1.282 | 1.372 | 1.458 | 1.796 | 0.071 | 1.065 | 1.164 | 1.256 | 1.344 | 1.427 |
| 9.7 | 1.830 | 0.072 | 1.084 | 1.186 | 1.282 | 1.373 | 1.458 | 1.757 | 0.071 | 1.067 | 1.165 | 1.258 | 1.345 | 1.428 |
| 9.8 | 1.810 | 0.072 | 1.084 | 1.187 | 1.282 | 1.373 | 1.459 | 1.720 | 0.071 | 1.069 | 1.167 | 1.259 | 1.347 | 1.430 |
| 9.9 | 1.789 | 0.072 | 1.085 | 1.187 | 1.283 | 1.373 | 1.459 | 1.686 | 0.071 | 1.072 | 1.169 | 1.261 | 1.349 | 1.432 |
| 10.0 | 1.769 | 0.072 | 1.086 | 1.187 | 1.283 | 1.373 | 1.459 | 1.654 | 0.071 | 1.074 | 1.171 | 1.263 | 1.350 | 1.434 |
| 10.1 | 1.749 | 0.072 | 1.086 | 1.188 | 1.283 | 1.373 | 1.459 | 1.625 | 0.071 | 1.076 | 1.173 | 1.265 | 1.352 | 1.437 |
| 10.2 | 1.729 | 0.072 | 1.087 | 1.188 | 1.283 | 1.374 | 1.460 | 1.599 | 0.071 | 1.079 | 1.175 | 1.267 | 1.354 | 1.439 |
| 10.3 | 1.709 | 0.072 | 1.088 | 1.189 | 1.284 | 1.374 | 1.460 | 1.576 | 0.071 | 1.082 | 1.177 | 1.269 | 1.356 | 1.441 |
| 10.4 | 1.690 | 0.072 | 1.088 | 1.189 | 1.284 | 1.374 | 1.461 | 1.556 | 0.070 | 1.084 | 1.180 | 1.271 | 1.359 | 1.443 |
| 10.5 | 1.670 | 0.072 | 1.089 | 1.190 | 1.284 | 1.375 | 1.461 | 1.540 | 0.070 | 1.087 | 1.182 | 1.273 | 1.361 | 1.446 |
| 10.6 | 1.650 | 0.072 | 1.090 | 1.190 | 1.285 | 1.375 | 1.462 | 1.526 | 0.070 | 1.090 | 1.185 | 1.276 | 1.363 | 1.448 |
| 10.7 | 1.631 | 0.072 | 1.091 | 1.191 | 1.285 | 1.376 | 1.462 | 1.516 | 0.070 | 1.093 | 1.187 | 1.278 | 1.366 | 1.451 |
| 10.8 | 1.611 | 0.072 | 1.092 | 1.192 | 1.286 | 1.376 | 1.463 | 1.509 | 0.070 | 1.096 | 1.190 | 1.281 | 1.368 | 1.453 |
| 10.9 | 1.592 | 0.072 | 1.093 | 1.192 | 1.287 | 1.377 | 1.464 | 1.504 | 0.069 | 1.099 | 1.193 | 1.284 | 1.371 | 1.456 |
| 11.0 | 1.573 | 0.072 | 1.095 | 1.193 | 1.287 | 1.378 | 1.465 | 1.503 | 0.069 | 1.102 | 1.196 | 1.287 | 1.374 | 1.459 |
| 11.1 | 1.554 | 0.071 | 1.096 | 1.194 | 1.288 | 1.378 | 1.465 | 1.504 | 0.069 | 1.106 | 1.200 | 1.290 | 1.377 | 1.462 |
| 11.2 | 1.535 | 0.071 | 1.097 | 1.195 | 1.289 | 1.379 | 1.466 | 1.508 | 0.068 | 1.109 | 1.203 | 1.293 | 1.380 | 1.465 |
| 11.3 | 1.516 | 0.071 | 1.099 | 1.196 | 1.290 | 1.380 | 1.467 | 1.514 | 0.068 | 1.113 | 1.207 | 1.297 | 1.384 | 1.468 |
| 11.4 | 1.498 | 0.071 | 1.100 | 1.197 | 1.291 | 1.381 | 1.468 | 1.522 | 0.068 | 1.117 | 1.211 | 1.300 | 1.387 | 1.471 |
| 11.5 | 1.479 | 0.071 | 1.102 | 1.199 | 1.292 | 1.382 | 1.469 | 1.533 | 0.068 | 1.121 | 1.214 | 1.304 | 1.391 | 1.475 |
| 11.6 | 1.461 | 0.071 | 1.104 | 1.200 | 1.293 | 1.383 | 1.471 | 1.545 | 0.067 | 1.125 | 1.219 | 1.308 | 1.394 | 1.478 |
| 11.7 | 1.443 | 0.071 | 1.105 | 1.202 | 1.294 | 1.384 | 1.472 | 1.558 | 0.067 | 1.129 | 1.223 | 1.312 | 1.398 | 1.481 |
| 11.8 | 1.425 | 0.070 | 1.107 | 1.203 | 1.296 | 1.386 | 1.473 | 1.573 | 0.066 | 1.134 | 1.227 | 1.316 | 1.402 | 1.485 |
| 11.9 | 1.407 | 0.070 | 1.109 | 1.205 | 1.297 | 1.387 | 1.474 | 1.589 | 0.066 | 1.138 | 1.231 | 1.320 | 1.406 | 1.489 |
| 12.0 | 1.390 | 0.070 | 1.112 | 1.207 | 1.299 | 1.388 | 1.476 | 1.606 | 0.066 | 1.143 | 1.236 | 1.325 | 1.410 | 1.493 |
| 12.1 | 1.372 | 0.070 | 1.114 | 1.209 | 1.300 | 1.390 | 1.477 | 1.624 | 0.065 | 1.147 | 1.240 | 1.329 | 1.414 | 1.496 |
| 12.2 | 1.355 | 0.069 | 1.116 | 1.211 | 1.302 | 1.392 | 1.479 | 1.642 | 0.065 | 1.152 | 1.245 | 1.334 | 1.419 | 1.500 |
| 12.3 | 1.339 | 0.069 | 1.119 | 1.213 | 1.304 | 1.393 | 1.481 | 1.661 | 0.065 | 1.157 | 1.250 | 1.338 | 1.423 | 1.504 |
| 12.4 | 1.322 | 0.069 | 1.122 | 1.215 | 1.306 | 1.395 | 1.482 | 1.680 | 0.064 | 1.162 | 1.254 | 1.343 | 1.427 | 1.508 |
| 12.5 | 1.306 | 0.069 | 1.124 | 1.217 | 1.308 | 1.397 | 1.484 | 1.700 | 0.064 | 1.167 | 1.259 | 1.347 | 1.431 | 1.512 |
| 12.6 | 1.291 | 0.068 | 1.127 | 1.220 | 1.310 | 1.399 | 1.486 | 1.720 | 0.063 | 1.172 | 1.264 | 1.352 | 1.436 | 1.516 |
| 12.7 | 1.275 | 0.068 | 1.130 | 1.222 | 1.313 | 1.401 | 1.488 | 1.740 | 0.063 | 1.177 | 1.269 | 1.356 | 1.440 | 1.520 |
| 12.8 | 1.260 | 0.068 | 1.134 | 1.225 | 1.315 | 1.403 | 1.490 | 1.760 | 0.063 | 1.181 | 1.273 | 1.361 | 1.444 | 1.524 |
| 12.9 | 1.246 | 0.067 | 1.137 | 1.228 | 1.318 | 1.406 | 1.492 | 1.781 | 0.062 | 1.186 | 1.278 | 1.365 | 1.448 | 1.527 |
| 13.0 | 1.232 | 0.067 | 1.140 | 1.231 | 1.320 | 1.408 | 1.494 | 1.801 | 0.062 | 1.191 | 1.283 | 1.369 | 1.452 | 1.531 |
| 13.1 | 1.218 | 0.067 | 1.144 | 1.234 | 1.323 | 1.410 | 1.497 | 1.822 | 0.061 | 1.196 | 1.287 | 1.374 | 1.456 | 1.534 |
| 13.2 | 1.205 | 0.066 | 1.148 | 1.237 | 1.326 | 1.413 | 1.499 | 1.844 | 0.061 | 1.200 | 1.292 | 1.378 | 1.460 | 1.538 |
| 13.3 | 1.192 | 0.066 | 1.151 | 1.241 | 1.329 | 1.415 | 1.501 | 1.866 | 0.060 | 1.205 | 1.296 | 1.382 | 1.463 | 1.541 |
| 13.4 | 1.180 | 0.065 | 1.155 | 1.244 | 1.331 | 1.418 | 1.504 | 1.889 | 0.060 | 1.210 | 1.300 | 1.386 | 1.467 | 1.544 |
| 13.5 | 1.169 | 0.065 | 1.159 | 1.247 | 1.335 | 1.421 | 1.506 | 1.912 | 0.060 | 1.214 | 1.305 | 1.390 | 1.470 | 1.547 |
| 13.6 | 1.158 | 0.065 | 1.163 | 1.251 | 1.338 | 1.423 | 1.509 | 1.937 | 0.059 | 1.218 | 1.309 | 1.393 | 1.474 | 1.550 |
| 13.7 | 1.147 | 0.064 | 1.167 | 1.255 | 1.341 | 1.426 | 1.511 | 1.963 | 0.059 | 1.222 | 1.312 | 1.397 | 1.477 | 1.553 |
| 13.8 | 1.137 | 0.064 | 1.172 | 1.258 | 1.344 | 1.429 | 1.514 | 1.990 | 0.058 | 1.226 | 1.316 | 1.400 | 1.480 | 1.556 |
| 13.9 | 1.128 | 0.063 | 1.176 | 1.262 | 1.347 | 1.432 | 1.516 | 2.019 | 0.058 | 1.230 | 1.320 | 1.404 | 1.483 | 1.558 |
| 14.0 | 1.119 | 0.063 | 1.180 | 1.266 | 1.351 | 1.435 | 1.519 | 2.048 | 0.058 | 1.233 | 1.323 | 1.407 | 1.486 | 1.560 |
| 14.1 | 1.111 | 0.062 | 1.185 | 1.270 | 1.354 | 1.438 | 1.521 | 2.078 | 0.057 | 1.237 | 1.326 | 1.410 | 1.488 | 1.562 |
| 14.2 | 1.103 | 0.062 | 1.189 | 1.274 | 1.358 | 1.441 | 1.524 | 2.110 | 0.057 | 1.240 | 1.329 | 1.413 | 1.491 | 1.565 |
| 14.3 | 1.096 | 0.061 | 1.193 | 1.278 | 1.361 | 1.444 | 1.527 | 2.142 | 0.057 | 1.243 | 1.332 | 1.415 | 1.493 | 1.566 |
| 14.4 | 1.090 | 0.061 | 1.198 | 1.282 | 1.365 | 1.447 | 1.530 | 2.175 | 0.056 | 1.246 | 1.335 | 1.418 | 1.495 | 1.568 |
| 14.5 | 1.084 | 0.060 | 1.202 | 1.286 | 1.368 | 1.450 | 1.532 | 2.209 | 0.056 | 1.249 | 1.338 | 1.420 | 1.497 | 1.570 |
| 14.6 | 1.079 | 0.060 | 1.207 | 1.290 | 1.372 | 1.454 | 1.535 | 2.244 | 0.056 | 1.252 | 1.341 | 1.423 | 1.499 | 1.572 |
| 14.7 | 1.075 | 0.059 | 1.212 | 1.294 | 1.375 | 1.457 | 1.538 | 2.280 | 0.055 | 1.254 | 1.343 | 1.425 | 1.501 | 1.573 |
| 14.8 | 1.071 | 0.059 | 1.216 | 1.298 | 1.379 | 1.460 | 1.540 | 2.316 | 0.055 | 1.257 | 1.346 | 1.427 | 1.503 | 1.575 |
| 14.9 | 1.068 | 0.058 | 1.221 | 1.302 | 1.382 | 1.463 | 1.543 | 2.353 | 0.055 | 1.259 | 1.348 | 1.430 | 1.505 | 1.576 |
| 15.0 | 1.065 | 0.058 | 1.225 | 1.306 | 1.386 | 1.466 | 1.546 | 2.390 | 0.055 | 1.262 | 1.350 | 1.432 | 1.507 | 1.577 |
| 15.1 | 1.063 | 0.057 | 1.230 | 1.310 | 1.390 | 1.469 | 1.548 | 2.427 | 0.054 | 1.264 | 1.353 | 1.434 | 1.508 | 1.578 |
| 15.2 | 1.062 | 0.057 | 1.234 | 1.314 | 1.393 | 1.472 | 1.551 | 2.465 | 0.054 | 1.266 | 1.355 | 1.435 | 1.510 | 1.580 |
| 15.3 | 1.062 | 0.056 | 1.239 | 1.318 | 1.397 | 1.475 | 1.553 | 2.503 | 0.054 | 1.268 | 1.357 | 1.437 | 1.512 | 1.581 |
| 15.4 | 1.062 | 0.056 | 1.243 | 1.322 | 1.400 | 1.478 | 1.556 | 2.540 | 0.053 | 1.270 | 1.359 | 1.439 | 1.513 | 1.582 |
| 15.5 | 1.063 | 0.055 | 1.247 | 1.325 | 1.403 | 1.481 | 1.558 | 2.578 | 0.053 | 1.272 | 1.361 | 1.441 | 1.515 | 1.583 |
| 15.6 | 1.065 | 0.055 | 1.251 | 1.329 | 1.407 | 1.484 | 1.561 | 2.616 | 0.053 | 1.274 | 1.362 | 1.443 | 1.516 | 1.584 |
| 15.7 | 1.067 | 0.055 | 1.255 | 1.333 | 1.410 | 1.487 | 1.563 | 2.653 | 0.053 | 1.276 | 1.364 | 1.444 | 1.517 | 1.585 |
| 15.8 | 1.070 | 0.054 | 1.260 | 1.336 | 1.413 | 1.489 | 1.565 | 2.690 | 0.053 | 1.277 | 1.366 | 1.446 | 1.519 | 1.586 |
| 15.9 | 1.074 | 0.054 | 1.263 | 1.340 | 1.416 | 1.492 | 1.568 | 2.726 | 0.052 | 1.279 | 1.368 | 1.447 | 1.520 | 1.587 |
| 16.0 | 1.079 | 0.053 | 1.267 | 1.344 | 1.419 | 1.495 | 1.570 | 2.762 | 0.052 | 1.281 | 1.369 | 1.449 | 1.521 | 1.588 |
| 16.1 | 1.084 | 0.053 | 1.271 | 1.347 | 1.422 | 1.497 | 1.572 | 2.798 | 0.052 | 1.282 | 1.371 | 1.450 | 1.522 | 1.589 |
| 16.2 | 1.091 | 0.053 | 1.275 | 1.350 | 1.425 | 1.500 | 1.574 | 2.832 | 0.052 | 1.284 | 1.372 | 1.452 | 1.524 | 1.590 |
| 16.3 | 1.098 | 0.052 | 1.278 | 1.353 | 1.428 | 1.502 | 1.576 | 2.866 | 0.052 | 1.285 | 1.374 | 1.453 | 1.525 | 1.591 |
| 16.4 | 1.105 | 0.052 | 1.282 | 1.357 | 1.431 | 1.505 | 1.578 | 2.899 | 0.052 | 1.287 | 1.375 | 1.454 | 1.526 | 1.592 |
| 16.5 | 1.114 | 0.051 | 1.285 | 1.360 | 1.434 | 1.507 | 1.580 | 2.931 | 0.051 | 1.288 | 1.377 | 1.456 | 1.527 | 1.592 |
| 16.6 | 1.123 | 0.051 | 1.288 | 1.363 | 1.436 | 1.509 | 1.582 | 2.961 | 0.051 | 1.290 | 1.378 | 1.457 | 1.528 | 1.593 |
| 16.7 | 1.133 | 0.051 | 1.292 | 1.365 | 1.439 | 1.512 | 1.584 | 2.991 | 0.051 | 1.291 | 1.380 | 1.458 | 1.529 | 1.594 |
| 16.8 | 1.144 | 0.051 | 1.295 | 1.368 | 1.441 | 1.514 | 1.586 | 3.018 | 0.051 | 1.292 | 1.381 | 1.459 | 1.530 | 1.595 |
| 16.9 | 1.156 | 0.050 | 1.298 | 1.371 | 1.444 | 1.516 | 1.588 | 3.044 | 0.051 | 1.293 | 1.382 | 1.460 | 1.531 | 1.595 |
| 17.0 | 1.169 | 0.050 | 1.300 | 1.374 | 1.446 | 1.518 | 1.589 | 3.068 | 0.051 | 1.295 | 1.383 | 1.462 | 1.532 | 1.596 |
| 17.1 | 1.182 | 0.050 | 1.303 | 1.376 | 1.448 | 1.520 | 1.591 | 3.090 | 0.050 | 1.296 | 1.385 | 1.463 | 1.533 | 1.597 |
| 17.2 | 1.196 | 0.049 | 1.306 | 1.378 | 1.450 | 1.522 | 1.593 | 3.110 | 0.050 | 1.297 | 1.386 | 1.464 | 1.534 | 1.598 |
| 17.3 | 1.211 | 0.049 | 1.308 | 1.381 | 1.452 | 1.524 | 1.594 | 3.127 | 0.050 | 1.299 | 1.387 | 1.465 | 1.535 | 1.598 |
| 17.4 | 1.226 | 0.049 | 1.310 | 1.383 | 1.454 | 1.525 | 1.595 | 3.142 | 0.050 | 1.300 | 1.388 | 1.466 | 1.536 | 1.599 |
| 17.5 | 1.242 | 0.049 | 1.312 | 1.385 | 1.456 | 1.527 | 1.597 | 3.154 | 0.050 | 1.301 | 1.389 | 1.467 | 1.537 | 1.600 |
| 17.6 | 1.259 | 0.049 | 1.314 | 1.387 | 1.458 | 1.529 | 1.598 | 3.164 | 0.050 | 1.303 | 1.391 | 1.468 | 1.537 | 1.601 |
| 17.7 | 1.277 | 0.048 | 1.316 | 1.389 | 1.460 | 1.530 | 1.600 | 3.171 | 0.050 | 1.304 | 1.392 | 1.469 | 1.538 | 1.601 |
| 17.8 | 1.295 | 0.048 | 1.318 | 1.390 | 1.461 | 1.532 | 1.601 | 3.175 | 0.050 | 1.305 | 1.393 | 1.470 | 1.539 | 1.602 |
| 17.9 | 1.314 | 0.048 | 1.320 | 1.392 | 1.463 | 1.533 | 1.602 | 3.177 | 0.049 | 1.306 | 1.394 | 1.471 | 1.540 | 1.603 |
| 18.0 | 1.333 | 0.048 | 1.321 | 1.394 | 1.465 | 1.534 | 1.603 | 3.176 | 0.049 | 1.308 | 1.395 | 1.472 | 1.541 | 1.603 |
| 18.1 | 1.353 | 0.048 | 1.323 | 1.395 | 1.466 | 1.536 | 1.604 | 3.171 | 0.049 | 1.309 | 1.396 | 1.473 | 1.541 | 1.604 |
| 18.2 | 1.373 | 0.048 | 1.324 | 1.396 | 1.467 | 1.537 | 1.605 | 3.164 | 0.049 | 1.310 | 1.397 | 1.473 | 1.542 | 1.605 |
| 18.3 | 1.395 | 0.048 | 1.326 | 1.398 | 1.468 | 1.538 | 1.606 | 3.154 | 0.049 | 1.312 | 1.398 | 1.474 | 1.543 | 1.605 |
| 18.4 | 1.416 | 0.048 | 1.327 | 1.399 | 1.470 | 1.539 | 1.607 | 3.141 | 0.049 | 1.313 | 1.399 | 1.475 | 1.543 | 1.605 |
| 18.5 | 1.438 | 0.048 | 1.328 | 1.400 | 1.471 | 1.540 | 1.608 | 3.125 | 0.049 | 1.314 | 1.400 | 1.475 | 1.543 | 1.606 |
| 18.6 | 1.461 | 0.048 | 1.329 | 1.401 | 1.472 | 1.541 | 1.609 | 3.107 | 0.048 | 1.315 | 1.400 | 1.476 | 1.544 | 1.606 |
| 18.7 | 1.484 | 0.047 | 1.330 | 1.402 | 1.473 | 1.542 | 1.610 | 3.086 | 0.048 | 1.316 | 1.401 | 1.476 | 1.544 | 1.606 |
| 18.8 | 1.507 | 0.047 | 1.330 | 1.403 | 1.474 | 1.543 | 1.610 | 3.062 | 0.048 | 1.317 | 1.402 | 1.477 | 1.544 | 1.607 |
| 18.9 | 1.531 | 0.047 | 1.331 | 1.404 | 1.475 | 1.544 | 1.611 | 3.035 | 0.048 | 1.319 | 1.402 | 1.477 | 1.545 | 1.607 |
| 19.0 | 1.555 | 0.047 | 1.332 | 1.405 | 1.476 | 1.545 | 1.612 | 3.006 | 0.048 | 1.320 | 1.403 | 1.477 | 1.545 | 1.607 |
| 19.1 | 1.580 | 0.047 | 1.332 | 1.406 | 1.476 | 1.545 | 1.613 | 2.975 | 0.048 | 1.321 | 1.403 | 1.477 | 1.545 | 1.607 |
| 19.2 | 1.605 | 0.047 | 1.333 | 1.406 | 1.477 | 1.546 | 1.613 | 2.941 | 0.047 | 1.322 | 1.404 | 1.477 | 1.544 | 1.606 |
| 19.3 | 1.630 | 0.047 | 1.333 | 1.407 | 1.478 | 1.547 | 1.614 | 2.905 | 0.047 | 1.322 | 1.404 | 1.477 | 1.544 | 1.606 |
| 19.4 | 1.656 | 0.047 | 1.334 | 1.407 | 1.479 | 1.548 | 1.615 | 2.866 | 0.047 | 1.323 | 1.404 | 1.477 | 1.544 | 1.606 |
| 19.5 | 1.681 | 0.047 | 1.334 | 1.408 | 1.479 | 1.548 | 1.615 | 2.825 | 0.047 | 1.324 | 1.404 | 1.477 | 1.544 | 1.605 |
| 19.6 | 1.708 | 0.047 | 1.334 | 1.409 | 1.480 | 1.549 | 1.616 | 2.783 | 0.047 | 1.325 | 1.405 | 1.477 | 1.543 | 1.605 |
| 19.7 | 1.734 | 0.047 | 1.335 | 1.409 | 1.481 | 1.550 | 1.617 | 2.738 | 0.047 | 1.326 | 1.405 | 1.477 | 1.543 | 1.605 |
| 19.8 | 1.761 | 0.048 | 1.335 | 1.409 | 1.481 | 1.550 | 1.617 | 2.692 | 0.046 | 1.327 | 1.405 | 1.476 | 1.542 | 1.604 |
| 19.9 | 1.788 | 0.048 | 1.335 | 1.410 | 1.482 | 1.551 | 1.618 | 2.643 | 0.046 | 1.327 | 1.405 | 1.476 | 1.542 | 1.603 |
| 20.0 | 1.816 | 0.048 | 1.335 | 1.410 | 1.482 | 1.551 | 1.618 | 2.593 | 0.046 | 1.328 | 1.405 | 1.476 | 1.541 | 1.603 |

| L3TBS | | | | | | | | | | | | | | |
| --- | --- | --- | --- | --- | --- | --- | --- | --- | --- | --- | --- | --- | --- | --- |
|  | Males | | | | | | | Females | | | | | | |
| Age | L | S | -2 SD | -1 SD | 0 SD (M) | 1 SD | 2 SD | L | S | -2 SD | -1 SD | 0 SD (M) | 1 SD | 2 SD |
| 5.0 | 3.515 | 0.074 | 1.057 | 1.195 | 1.301 | 1.389 | 1.465 | 1.132 | 0.066 | 1.107 | 1.193 | 1.278 | 1.362 | 1.446 |
| 5.1 | 3.462 | 0.073 | 1.061 | 1.196 | 1.302 | 1.390 | 1.466 | 1.142 | 0.067 | 1.106 | 1.192 | 1.278 | 1.362 | 1.446 |
| 5.2 | 3.409 | 0.073 | 1.065 | 1.198 | 1.303 | 1.390 | 1.466 | 1.152 | 0.067 | 1.105 | 1.191 | 1.277 | 1.362 | 1.446 |
| 5.3 | 3.356 | 0.073 | 1.069 | 1.200 | 1.303 | 1.391 | 1.467 | 1.161 | 0.067 | 1.104 | 1.191 | 1.277 | 1.362 | 1.446 |
| 5.4 | 3.304 | 0.072 | 1.072 | 1.201 | 1.304 | 1.391 | 1.468 | 1.171 | 0.067 | 1.103 | 1.190 | 1.276 | 1.361 | 1.446 |
| 5.5 | 3.252 | 0.072 | 1.075 | 1.203 | 1.305 | 1.392 | 1.468 | 1.181 | 0.067 | 1.102 | 1.189 | 1.276 | 1.361 | 1.446 |
| 5.6 | 3.200 | 0.071 | 1.079 | 1.204 | 1.306 | 1.393 | 1.469 | 1.191 | 0.068 | 1.100 | 1.188 | 1.275 | 1.361 | 1.446 |
| 5.7 | 3.149 | 0.071 | 1.082 | 1.205 | 1.306 | 1.393 | 1.469 | 1.201 | 0.068 | 1.099 | 1.188 | 1.275 | 1.361 | 1.445 |
| 5.8 | 3.099 | 0.071 | 1.085 | 1.207 | 1.307 | 1.394 | 1.470 | 1.210 | 0.068 | 1.098 | 1.187 | 1.274 | 1.360 | 1.445 |
| 5.9 | 3.049 | 0.070 | 1.088 | 1.208 | 1.308 | 1.394 | 1.470 | 1.220 | 0.068 | 1.097 | 1.186 | 1.274 | 1.360 | 1.445 |
| 6.0 | 3.000 | 0.070 | 1.091 | 1.209 | 1.308 | 1.394 | 1.471 | 1.230 | 0.068 | 1.096 | 1.186 | 1.274 | 1.360 | 1.445 |
| 6.1 | 2.951 | 0.070 | 1.093 | 1.211 | 1.309 | 1.395 | 1.471 | 1.240 | 0.069 | 1.096 | 1.185 | 1.273 | 1.360 | 1.445 |
| 6.2 | 2.903 | 0.070 | 1.096 | 1.212 | 1.310 | 1.395 | 1.472 | 1.249 | 0.069 | 1.095 | 1.185 | 1.273 | 1.360 | 1.445 |
| 6.3 | 2.856 | 0.069 | 1.099 | 1.213 | 1.310 | 1.396 | 1.472 | 1.259 | 0.069 | 1.094 | 1.184 | 1.273 | 1.360 | 1.445 |
| 6.4 | 2.809 | 0.069 | 1.101 | 1.214 | 1.311 | 1.396 | 1.473 | 1.269 | 0.069 | 1.093 | 1.184 | 1.273 | 1.360 | 1.445 |
| 6.5 | 2.763 | 0.069 | 1.103 | 1.215 | 1.311 | 1.396 | 1.473 | 1.278 | 0.069 | 1.093 | 1.183 | 1.272 | 1.360 | 1.445 |
| 6.6 | 2.719 | 0.068 | 1.106 | 1.216 | 1.312 | 1.397 | 1.473 | 1.288 | 0.069 | 1.092 | 1.183 | 1.272 | 1.360 | 1.445 |
| 6.7 | 2.674 | 0.068 | 1.108 | 1.217 | 1.312 | 1.397 | 1.474 | 1.297 | 0.069 | 1.092 | 1.183 | 1.272 | 1.360 | 1.445 |
| 6.8 | 2.631 | 0.068 | 1.110 | 1.218 | 1.312 | 1.397 | 1.474 | 1.307 | 0.069 | 1.091 | 1.183 | 1.272 | 1.360 | 1.445 |
| 6.9 | 2.589 | 0.068 | 1.112 | 1.219 | 1.313 | 1.397 | 1.474 | 1.316 | 0.070 | 1.091 | 1.183 | 1.272 | 1.360 | 1.445 |
| 7.0 | 2.547 | 0.067 | 1.113 | 1.220 | 1.313 | 1.397 | 1.474 | 1.326 | 0.070 | 1.091 | 1.183 | 1.272 | 1.360 | 1.446 |
| 7.1 | 2.507 | 0.067 | 1.115 | 1.220 | 1.313 | 1.397 | 1.474 | 1.335 | 0.070 | 1.091 | 1.183 | 1.272 | 1.360 | 1.446 |
| 7.2 | 2.467 | 0.067 | 1.117 | 1.221 | 1.314 | 1.397 | 1.475 | 1.344 | 0.070 | 1.091 | 1.183 | 1.273 | 1.360 | 1.446 |
| 7.3 | 2.428 | 0.067 | 1.118 | 1.222 | 1.314 | 1.398 | 1.475 | 1.354 | 0.070 | 1.091 | 1.183 | 1.273 | 1.360 | 1.446 |
| 7.4 | 2.389 | 0.067 | 1.120 | 1.222 | 1.314 | 1.398 | 1.475 | 1.363 | 0.070 | 1.091 | 1.183 | 1.273 | 1.360 | 1.446 |
| 7.5 | 2.351 | 0.066 | 1.121 | 1.223 | 1.314 | 1.398 | 1.475 | 1.372 | 0.069 | 1.091 | 1.184 | 1.273 | 1.361 | 1.446 |
| 7.6 | 2.314 | 0.066 | 1.122 | 1.223 | 1.314 | 1.398 | 1.475 | 1.381 | 0.069 | 1.092 | 1.184 | 1.274 | 1.361 | 1.446 |
| 7.7 | 2.277 | 0.066 | 1.124 | 1.224 | 1.314 | 1.398 | 1.475 | 1.390 | 0.069 | 1.092 | 1.184 | 1.274 | 1.361 | 1.446 |
| 7.8 | 2.241 | 0.066 | 1.125 | 1.224 | 1.314 | 1.398 | 1.475 | 1.399 | 0.069 | 1.093 | 1.185 | 1.274 | 1.361 | 1.446 |
| 7.9 | 2.205 | 0.066 | 1.126 | 1.225 | 1.315 | 1.398 | 1.475 | 1.408 | 0.069 | 1.093 | 1.185 | 1.275 | 1.362 | 1.446 |
| 8.0 | 2.170 | 0.065 | 1.127 | 1.225 | 1.315 | 1.398 | 1.475 | 1.417 | 0.069 | 1.094 | 1.186 | 1.275 | 1.362 | 1.446 |
| 8.1 | 2.135 | 0.065 | 1.128 | 1.226 | 1.315 | 1.398 | 1.475 | 1.426 | 0.069 | 1.094 | 1.186 | 1.275 | 1.362 | 1.446 |
| 8.2 | 2.100 | 0.065 | 1.129 | 1.226 | 1.315 | 1.398 | 1.476 | 1.435 | 0.069 | 1.095 | 1.187 | 1.276 | 1.362 | 1.446 |
| 8.3 | 2.066 | 0.065 | 1.130 | 1.226 | 1.315 | 1.398 | 1.476 | 1.444 | 0.069 | 1.096 | 1.187 | 1.276 | 1.362 | 1.446 |
| 8.4 | 2.032 | 0.065 | 1.131 | 1.227 | 1.315 | 1.398 | 1.476 | 1.453 | 0.068 | 1.096 | 1.188 | 1.277 | 1.363 | 1.446 |
| 8.5 | 1.998 | 0.065 | 1.132 | 1.227 | 1.315 | 1.398 | 1.476 | 1.462 | 0.068 | 1.097 | 1.189 | 1.277 | 1.363 | 1.446 |
| 8.6 | 1.965 | 0.065 | 1.133 | 1.228 | 1.316 | 1.398 | 1.476 | 1.472 | 0.068 | 1.098 | 1.189 | 1.278 | 1.363 | 1.446 |
| 8.7 | 1.932 | 0.065 | 1.134 | 1.228 | 1.316 | 1.398 | 1.477 | 1.481 | 0.068 | 1.098 | 1.190 | 1.278 | 1.364 | 1.446 |
| 8.8 | 1.899 | 0.065 | 1.135 | 1.228 | 1.316 | 1.398 | 1.477 | 1.490 | 0.068 | 1.099 | 1.191 | 1.279 | 1.364 | 1.447 |
| 8.9 | 1.866 | 0.064 | 1.136 | 1.229 | 1.316 | 1.399 | 1.477 | 1.499 | 0.068 | 1.100 | 1.191 | 1.279 | 1.364 | 1.447 |
| 9.0 | 1.833 | 0.064 | 1.136 | 1.229 | 1.316 | 1.399 | 1.478 | 1.508 | 0.067 | 1.101 | 1.192 | 1.280 | 1.365 | 1.447 |
| 9.1 | 1.801 | 0.064 | 1.137 | 1.229 | 1.316 | 1.399 | 1.478 | 1.517 | 0.067 | 1.102 | 1.193 | 1.281 | 1.365 | 1.447 |
| 9.2 | 1.768 | 0.064 | 1.138 | 1.230 | 1.316 | 1.399 | 1.478 | 1.527 | 0.067 | 1.103 | 1.194 | 1.281 | 1.366 | 1.448 |
| 9.3 | 1.736 | 0.064 | 1.139 | 1.230 | 1.317 | 1.399 | 1.478 | 1.536 | 0.067 | 1.104 | 1.195 | 1.282 | 1.366 | 1.448 |
| 9.4 | 1.704 | 0.064 | 1.139 | 1.230 | 1.317 | 1.399 | 1.479 | 1.546 | 0.067 | 1.105 | 1.196 | 1.283 | 1.367 | 1.449 |
| 9.5 | 1.671 | 0.064 | 1.140 | 1.230 | 1.317 | 1.400 | 1.479 | 1.555 | 0.067 | 1.106 | 1.197 | 1.284 | 1.368 | 1.449 |
| 9.6 | 1.639 | 0.064 | 1.140 | 1.231 | 1.317 | 1.400 | 1.479 | 1.565 | 0.066 | 1.107 | 1.198 | 1.285 | 1.369 | 1.450 |
| 9.7 | 1.607 | 0.064 | 1.141 | 1.231 | 1.317 | 1.400 | 1.480 | 1.575 | 0.066 | 1.109 | 1.199 | 1.286 | 1.370 | 1.451 |
| 9.8 | 1.575 | 0.064 | 1.141 | 1.231 | 1.317 | 1.400 | 1.480 | 1.585 | 0.066 | 1.110 | 1.201 | 1.287 | 1.371 | 1.452 |
| 9.9 | 1.543 | 0.064 | 1.142 | 1.231 | 1.317 | 1.400 | 1.480 | 1.595 | 0.066 | 1.111 | 1.202 | 1.289 | 1.372 | 1.453 |
| 10.0 | 1.511 | 0.064 | 1.142 | 1.231 | 1.317 | 1.400 | 1.480 | 1.605 | 0.066 | 1.113 | 1.204 | 1.290 | 1.374 | 1.454 |
| 10.1 | 1.479 | 0.064 | 1.142 | 1.231 | 1.317 | 1.400 | 1.481 | 1.616 | 0.066 | 1.115 | 1.205 | 1.292 | 1.375 | 1.455 |
| 10.2 | 1.448 | 0.064 | 1.143 | 1.231 | 1.317 | 1.400 | 1.481 | 1.626 | 0.065 | 1.116 | 1.207 | 1.294 | 1.377 | 1.457 |
| 10.3 | 1.416 | 0.064 | 1.143 | 1.231 | 1.317 | 1.400 | 1.481 | 1.637 | 0.065 | 1.118 | 1.209 | 1.295 | 1.378 | 1.458 |
| 10.4 | 1.385 | 0.064 | 1.144 | 1.231 | 1.317 | 1.400 | 1.482 | 1.648 | 0.065 | 1.120 | 1.211 | 1.297 | 1.380 | 1.460 |
| 10.5 | 1.354 | 0.064 | 1.144 | 1.231 | 1.317 | 1.400 | 1.482 | 1.659 | 0.065 | 1.122 | 1.213 | 1.299 | 1.382 | 1.462 |
| 10.6 | 1.324 | 0.064 | 1.144 | 1.231 | 1.317 | 1.400 | 1.482 | 1.670 | 0.065 | 1.124 | 1.215 | 1.302 | 1.384 | 1.464 |
| 10.7 | 1.293 | 0.064 | 1.145 | 1.232 | 1.317 | 1.400 | 1.483 | 1.681 | 0.065 | 1.127 | 1.218 | 1.304 | 1.387 | 1.466 |
| 10.8 | 1.263 | 0.064 | 1.145 | 1.232 | 1.317 | 1.401 | 1.483 | 1.692 | 0.065 | 1.129 | 1.220 | 1.306 | 1.389 | 1.468 |
| 10.9 | 1.233 | 0.064 | 1.145 | 1.232 | 1.317 | 1.401 | 1.484 | 1.704 | 0.064 | 1.132 | 1.223 | 1.309 | 1.391 | 1.471 |
| 11.0 | 1.204 | 0.064 | 1.146 | 1.232 | 1.317 | 1.401 | 1.484 | 1.716 | 0.064 | 1.135 | 1.226 | 1.312 | 1.394 | 1.473 |
| 11.1 | 1.174 | 0.064 | 1.146 | 1.232 | 1.317 | 1.401 | 1.485 | 1.728 | 0.064 | 1.138 | 1.228 | 1.315 | 1.397 | 1.476 |
| 11.2 | 1.145 | 0.064 | 1.147 | 1.233 | 1.318 | 1.402 | 1.485 | 1.740 | 0.064 | 1.141 | 1.232 | 1.318 | 1.400 | 1.479 |
| 11.3 | 1.117 | 0.064 | 1.148 | 1.233 | 1.318 | 1.402 | 1.486 | 1.752 | 0.064 | 1.144 | 1.235 | 1.321 | 1.403 | 1.481 |
| 11.4 | 1.088 | 0.064 | 1.149 | 1.234 | 1.319 | 1.403 | 1.487 | 1.765 | 0.063 | 1.147 | 1.238 | 1.324 | 1.406 | 1.485 |
| 11.5 | 1.060 | 0.064 | 1.149 | 1.234 | 1.319 | 1.404 | 1.488 | 1.777 | 0.063 | 1.151 | 1.242 | 1.328 | 1.409 | 1.488 |
| 11.6 | 1.033 | 0.064 | 1.150 | 1.235 | 1.320 | 1.404 | 1.489 | 1.790 | 0.063 | 1.155 | 1.245 | 1.331 | 1.413 | 1.491 |
| 11.7 | 1.006 | 0.064 | 1.152 | 1.236 | 1.321 | 1.405 | 1.490 | 1.803 | 0.062 | 1.159 | 1.249 | 1.335 | 1.416 | 1.494 |
| 11.8 | 0.979 | 0.064 | 1.153 | 1.237 | 1.321 | 1.406 | 1.491 | 1.816 | 0.062 | 1.163 | 1.253 | 1.339 | 1.420 | 1.497 |
| 11.9 | 0.952 | 0.064 | 1.154 | 1.238 | 1.323 | 1.407 | 1.492 | 1.829 | 0.062 | 1.167 | 1.257 | 1.343 | 1.423 | 1.501 |
| 12.0 | 0.926 | 0.064 | 1.156 | 1.239 | 1.324 | 1.408 | 1.493 | 1.843 | 0.061 | 1.171 | 1.261 | 1.346 | 1.427 | 1.504 |
| 12.1 | 0.900 | 0.064 | 1.157 | 1.241 | 1.325 | 1.410 | 1.495 | 1.857 | 0.061 | 1.176 | 1.266 | 1.350 | 1.431 | 1.508 |
| 12.2 | 0.875 | 0.064 | 1.159 | 1.242 | 1.326 | 1.411 | 1.496 | 1.870 | 0.061 | 1.180 | 1.270 | 1.355 | 1.435 | 1.511 |
| 12.3 | 0.850 | 0.063 | 1.161 | 1.244 | 1.328 | 1.413 | 1.498 | 1.884 | 0.060 | 1.185 | 1.275 | 1.359 | 1.439 | 1.515 |
| 12.4 | 0.825 | 0.063 | 1.163 | 1.246 | 1.330 | 1.414 | 1.500 | 1.899 | 0.060 | 1.190 | 1.279 | 1.363 | 1.442 | 1.518 |
| 12.5 | 0.801 | 0.063 | 1.166 | 1.248 | 1.332 | 1.416 | 1.501 | 1.913 | 0.059 | 1.195 | 1.284 | 1.367 | 1.446 | 1.522 |
| 12.6 | 0.777 | 0.063 | 1.168 | 1.250 | 1.334 | 1.418 | 1.503 | 1.928 | 0.059 | 1.200 | 1.288 | 1.371 | 1.450 | 1.525 |
| 12.7 | 0.754 | 0.063 | 1.171 | 1.253 | 1.336 | 1.420 | 1.506 | 1.943 | 0.058 | 1.205 | 1.293 | 1.375 | 1.454 | 1.528 |
| 12.8 | 0.732 | 0.062 | 1.174 | 1.255 | 1.338 | 1.422 | 1.508 | 1.958 | 0.058 | 1.209 | 1.297 | 1.380 | 1.458 | 1.532 |
| 12.9 | 0.710 | 0.062 | 1.177 | 1.258 | 1.341 | 1.425 | 1.510 | 1.973 | 0.058 | 1.214 | 1.302 | 1.384 | 1.461 | 1.535 |
| 13.0 | 0.688 | 0.062 | 1.180 | 1.261 | 1.343 | 1.427 | 1.513 | 1.988 | 0.057 | 1.219 | 1.306 | 1.388 | 1.465 | 1.538 |
| 13.1 | 0.668 | 0.062 | 1.184 | 1.264 | 1.346 | 1.430 | 1.515 | 2.004 | 0.057 | 1.224 | 1.311 | 1.392 | 1.468 | 1.541 |
| 13.2 | 0.648 | 0.061 | 1.188 | 1.267 | 1.349 | 1.433 | 1.518 | 2.020 | 0.056 | 1.229 | 1.315 | 1.396 | 1.472 | 1.544 |
| 13.3 | 0.629 | 0.061 | 1.191 | 1.271 | 1.352 | 1.435 | 1.520 | 2.036 | 0.056 | 1.234 | 1.319 | 1.400 | 1.475 | 1.547 |
| 13.4 | 0.611 | 0.061 | 1.195 | 1.274 | 1.355 | 1.438 | 1.523 | 2.052 | 0.055 | 1.238 | 1.324 | 1.403 | 1.479 | 1.550 |
| 13.5 | 0.594 | 0.060 | 1.199 | 1.278 | 1.359 | 1.442 | 1.526 | 2.069 | 0.055 | 1.243 | 1.328 | 1.407 | 1.482 | 1.553 |
| 13.6 | 0.579 | 0.060 | 1.204 | 1.282 | 1.362 | 1.445 | 1.529 | 2.086 | 0.054 | 1.248 | 1.332 | 1.411 | 1.485 | 1.555 |
| 13.7 | 0.564 | 0.059 | 1.208 | 1.286 | 1.366 | 1.448 | 1.532 | 2.103 | 0.054 | 1.252 | 1.336 | 1.414 | 1.488 | 1.558 |
| 13.8 | 0.550 | 0.059 | 1.212 | 1.290 | 1.370 | 1.452 | 1.536 | 2.120 | 0.053 | 1.256 | 1.339 | 1.417 | 1.491 | 1.560 |
| 13.9 | 0.537 | 0.059 | 1.217 | 1.294 | 1.373 | 1.455 | 1.539 | 2.137 | 0.053 | 1.260 | 1.343 | 1.420 | 1.493 | 1.562 |
| 14.0 | 0.526 | 0.058 | 1.221 | 1.298 | 1.377 | 1.459 | 1.542 | 2.155 | 0.052 | 1.264 | 1.347 | 1.424 | 1.496 | 1.565 |
| 14.1 | 0.516 | 0.058 | 1.226 | 1.303 | 1.381 | 1.462 | 1.545 | 2.173 | 0.052 | 1.268 | 1.350 | 1.427 | 1.499 | 1.567 |
| 14.2 | 0.507 | 0.057 | 1.231 | 1.307 | 1.385 | 1.466 | 1.549 | 2.191 | 0.052 | 1.272 | 1.353 | 1.429 | 1.501 | 1.569 |
| 14.3 | 0.500 | 0.057 | 1.235 | 1.311 | 1.389 | 1.469 | 1.552 | 2.210 | 0.051 | 1.275 | 1.356 | 1.432 | 1.503 | 1.571 |
| 14.4 | 0.494 | 0.057 | 1.240 | 1.315 | 1.393 | 1.473 | 1.555 | 2.228 | 0.051 | 1.279 | 1.360 | 1.435 | 1.506 | 1.573 |
| 14.5 | 0.489 | 0.056 | 1.245 | 1.320 | 1.397 | 1.477 | 1.558 | 2.247 | 0.051 | 1.282 | 1.362 | 1.438 | 1.508 | 1.575 |
| 14.6 | 0.487 | 0.056 | 1.250 | 1.324 | 1.401 | 1.480 | 1.562 | 2.266 | 0.050 | 1.285 | 1.365 | 1.440 | 1.510 | 1.576 |
| 14.7 | 0.485 | 0.055 | 1.254 | 1.329 | 1.405 | 1.484 | 1.565 | 2.285 | 0.050 | 1.288 | 1.368 | 1.443 | 1.512 | 1.578 |
| 14.8 | 0.486 | 0.055 | 1.259 | 1.333 | 1.409 | 1.487 | 1.568 | 2.305 | 0.050 | 1.291 | 1.371 | 1.445 | 1.514 | 1.580 |
| 14.9 | 0.488 | 0.054 | 1.263 | 1.337 | 1.413 | 1.491 | 1.571 | 2.324 | 0.049 | 1.294 | 1.373 | 1.447 | 1.516 | 1.582 |
| 15.0 | 0.492 | 0.054 | 1.268 | 1.341 | 1.417 | 1.495 | 1.574 | 2.344 | 0.049 | 1.296 | 1.376 | 1.449 | 1.518 | 1.583 |
| 15.1 | 0.498 | 0.054 | 1.272 | 1.345 | 1.421 | 1.498 | 1.577 | 2.364 | 0.049 | 1.299 | 1.378 | 1.451 | 1.520 | 1.585 |
| 15.2 | 0.505 | 0.053 | 1.277 | 1.349 | 1.424 | 1.501 | 1.580 | 2.384 | 0.049 | 1.301 | 1.380 | 1.454 | 1.522 | 1.586 |
| 15.3 | 0.515 | 0.053 | 1.281 | 1.353 | 1.428 | 1.505 | 1.583 | 2.405 | 0.048 | 1.304 | 1.382 | 1.455 | 1.524 | 1.588 |
| 15.4 | 0.526 | 0.053 | 1.285 | 1.357 | 1.432 | 1.508 | 1.586 | 2.425 | 0.048 | 1.306 | 1.385 | 1.457 | 1.525 | 1.589 |
| 15.5 | 0.540 | 0.052 | 1.289 | 1.361 | 1.435 | 1.511 | 1.589 | 2.446 | 0.048 | 1.308 | 1.386 | 1.459 | 1.527 | 1.591 |
| 15.6 | 0.555 | 0.052 | 1.293 | 1.365 | 1.439 | 1.514 | 1.591 | 2.467 | 0.048 | 1.310 | 1.388 | 1.461 | 1.528 | 1.592 |
| 15.7 | 0.572 | 0.052 | 1.297 | 1.368 | 1.442 | 1.517 | 1.594 | 2.488 | 0.048 | 1.312 | 1.390 | 1.462 | 1.530 | 1.593 |
| 15.8 | 0.592 | 0.051 | 1.300 | 1.372 | 1.445 | 1.520 | 1.597 | 2.510 | 0.048 | 1.313 | 1.392 | 1.464 | 1.531 | 1.594 |
| 15.9 | 0.613 | 0.051 | 1.304 | 1.375 | 1.448 | 1.523 | 1.599 | 2.531 | 0.047 | 1.315 | 1.393 | 1.466 | 1.533 | 1.596 |
| 16.0 | 0.635 | 0.051 | 1.307 | 1.379 | 1.452 | 1.526 | 1.601 | 2.553 | 0.047 | 1.316 | 1.395 | 1.467 | 1.534 | 1.597 |
| 16.1 | 0.660 | 0.050 | 1.311 | 1.382 | 1.455 | 1.528 | 1.604 | 2.575 | 0.047 | 1.318 | 1.396 | 1.468 | 1.535 | 1.598 |
| 16.2 | 0.687 | 0.050 | 1.314 | 1.385 | 1.457 | 1.531 | 1.606 | 2.597 | 0.047 | 1.319 | 1.398 | 1.470 | 1.537 | 1.599 |
| 16.3 | 0.715 | 0.050 | 1.317 | 1.388 | 1.460 | 1.533 | 1.608 | 2.619 | 0.047 | 1.320 | 1.399 | 1.471 | 1.538 | 1.600 |
| 16.4 | 0.746 | 0.050 | 1.320 | 1.391 | 1.463 | 1.536 | 1.610 | 2.641 | 0.047 | 1.322 | 1.400 | 1.473 | 1.539 | 1.602 |
| 16.5 | 0.778 | 0.049 | 1.322 | 1.394 | 1.465 | 1.538 | 1.611 | 2.663 | 0.047 | 1.323 | 1.402 | 1.474 | 1.540 | 1.603 |
| 16.6 | 0.811 | 0.049 | 1.325 | 1.396 | 1.468 | 1.540 | 1.613 | 2.686 | 0.047 | 1.324 | 1.403 | 1.475 | 1.542 | 1.604 |
| 16.7 | 0.847 | 0.049 | 1.328 | 1.399 | 1.470 | 1.542 | 1.615 | 2.708 | 0.047 | 1.325 | 1.404 | 1.476 | 1.543 | 1.605 |
| 16.8 | 0.883 | 0.049 | 1.330 | 1.401 | 1.473 | 1.544 | 1.617 | 2.731 | 0.047 | 1.326 | 1.405 | 1.478 | 1.544 | 1.606 |
| 16.9 | 0.922 | 0.048 | 1.332 | 1.403 | 1.475 | 1.546 | 1.618 | 2.754 | 0.047 | 1.327 | 1.407 | 1.479 | 1.545 | 1.607 |
| 17.0 | 0.962 | 0.048 | 1.335 | 1.406 | 1.477 | 1.548 | 1.620 | 2.777 | 0.047 | 1.328 | 1.408 | 1.480 | 1.546 | 1.608 |
| 17.1 | 1.004 | 0.048 | 1.337 | 1.408 | 1.479 | 1.550 | 1.621 | 2.800 | 0.047 | 1.329 | 1.409 | 1.481 | 1.548 | 1.609 |
| 17.2 | 1.047 | 0.048 | 1.339 | 1.410 | 1.481 | 1.552 | 1.623 | 2.823 | 0.047 | 1.330 | 1.410 | 1.482 | 1.549 | 1.610 |
| 17.3 | 1.091 | 0.048 | 1.341 | 1.412 | 1.483 | 1.554 | 1.624 | 2.846 | 0.047 | 1.331 | 1.411 | 1.483 | 1.550 | 1.611 |
| 17.4 | 1.137 | 0.048 | 1.343 | 1.414 | 1.485 | 1.555 | 1.625 | 2.870 | 0.047 | 1.332 | 1.412 | 1.485 | 1.551 | 1.612 |
| 17.5 | 1.184 | 0.047 | 1.344 | 1.416 | 1.487 | 1.557 | 1.626 | 2.893 | 0.047 | 1.333 | 1.413 | 1.486 | 1.552 | 1.613 |
| 17.6 | 1.232 | 0.047 | 1.346 | 1.418 | 1.488 | 1.558 | 1.628 | 2.916 | 0.047 | 1.334 | 1.414 | 1.487 | 1.553 | 1.614 |
| 17.7 | 1.282 | 0.047 | 1.347 | 1.419 | 1.490 | 1.560 | 1.629 | 2.940 | 0.046 | 1.335 | 1.415 | 1.488 | 1.554 | 1.615 |
| 17.8 | 1.333 | 0.047 | 1.349 | 1.421 | 1.492 | 1.561 | 1.630 | 2.964 | 0.046 | 1.336 | 1.416 | 1.489 | 1.555 | 1.616 |
| 17.9 | 1.385 | 0.047 | 1.350 | 1.422 | 1.493 | 1.563 | 1.631 | 2.987 | 0.046 | 1.337 | 1.417 | 1.490 | 1.556 | 1.617 |
| 18.0 | 1.439 | 0.047 | 1.351 | 1.424 | 1.495 | 1.564 | 1.632 | 3.011 | 0.046 | 1.337 | 1.418 | 1.491 | 1.557 | 1.618 |
| 18.1 | 1.493 | 0.047 | 1.352 | 1.425 | 1.496 | 1.565 | 1.633 | 3.034 | 0.046 | 1.338 | 1.419 | 1.492 | 1.558 | 1.618 |
| 18.2 | 1.549 | 0.047 | 1.354 | 1.427 | 1.497 | 1.567 | 1.634 | 3.058 | 0.046 | 1.339 | 1.420 | 1.493 | 1.558 | 1.619 |
| 18.3 | 1.605 | 0.047 | 1.355 | 1.428 | 1.499 | 1.568 | 1.635 | 3.082 | 0.046 | 1.340 | 1.421 | 1.493 | 1.559 | 1.619 |
| 18.4 | 1.663 | 0.047 | 1.355 | 1.429 | 1.500 | 1.569 | 1.636 | 3.106 | 0.046 | 1.341 | 1.422 | 1.494 | 1.560 | 1.620 |
| 18.5 | 1.722 | 0.047 | 1.356 | 1.430 | 1.501 | 1.570 | 1.637 | 3.130 | 0.046 | 1.342 | 1.423 | 1.495 | 1.560 | 1.620 |
| 18.6 | 1.781 | 0.047 | 1.357 | 1.431 | 1.503 | 1.571 | 1.638 | 3.153 | 0.046 | 1.342 | 1.423 | 1.495 | 1.561 | 1.621 |
| 18.7 | 1.841 | 0.047 | 1.358 | 1.432 | 1.504 | 1.572 | 1.638 | 3.177 | 0.046 | 1.343 | 1.424 | 1.496 | 1.561 | 1.621 |
| 18.8 | 1.903 | 0.046 | 1.358 | 1.433 | 1.505 | 1.573 | 1.639 | 3.201 | 0.046 | 1.344 | 1.425 | 1.496 | 1.561 | 1.621 |
| 18.9 | 1.964 | 0.046 | 1.359 | 1.434 | 1.506 | 1.574 | 1.640 | 3.225 | 0.045 | 1.345 | 1.425 | 1.497 | 1.562 | 1.621 |
| 19.0 | 2.027 | 0.046 | 1.360 | 1.435 | 1.507 | 1.575 | 1.641 | 3.249 | 0.045 | 1.345 | 1.426 | 1.497 | 1.562 | 1.621 |
| 19.1 | 2.090 | 0.046 | 1.360 | 1.436 | 1.508 | 1.576 | 1.641 | 3.273 | 0.045 | 1.346 | 1.426 | 1.498 | 1.562 | 1.621 |
| 19.2 | 2.154 | 0.046 | 1.360 | 1.437 | 1.509 | 1.577 | 1.642 | 3.297 | 0.045 | 1.346 | 1.427 | 1.498 | 1.562 | 1.620 |
| 19.3 | 2.219 | 0.046 | 1.361 | 1.438 | 1.510 | 1.578 | 1.643 | 3.321 | 0.045 | 1.347 | 1.427 | 1.498 | 1.562 | 1.620 |
| 19.4 | 2.284 | 0.046 | 1.361 | 1.438 | 1.511 | 1.579 | 1.643 | 3.345 | 0.045 | 1.347 | 1.427 | 1.498 | 1.562 | 1.620 |
| 19.5 | 2.349 | 0.046 | 1.361 | 1.439 | 1.511 | 1.579 | 1.644 | 3.369 | 0.044 | 1.348 | 1.428 | 1.498 | 1.561 | 1.619 |
| 19.6 | 2.416 | 0.046 | 1.361 | 1.440 | 1.512 | 1.580 | 1.644 | 3.392 | 0.044 | 1.348 | 1.428 | 1.498 | 1.561 | 1.619 |
| 19.7 | 2.482 | 0.046 | 1.361 | 1.440 | 1.513 | 1.581 | 1.644 | 3.416 | 0.044 | 1.349 | 1.428 | 1.498 | 1.561 | 1.618 |
| 19.8 | 2.549 | 0.046 | 1.361 | 1.440 | 1.513 | 1.581 | 1.645 | 3.440 | 0.044 | 1.349 | 1.428 | 1.498 | 1.560 | 1.617 |
| 19.9 | 2.616 | 0.046 | 1.361 | 1.441 | 1.514 | 1.582 | 1.645 | 3.464 | 0.044 | 1.350 | 1.428 | 1.498 | 1.560 | 1.617 |
| 20.0 | 2.684 | 0.046 | 1.361 | 1.441 | 1.514 | 1.582 | 1.645 | 3.488 | 0.044 | 1.350 | 1.429 | 1.498 | 1.560 | 1.616 |

| L4 TBS | | | | | | | | | | | | | | |
| --- | --- | --- | --- | --- | --- | --- | --- | --- | --- | --- | --- | --- | --- | --- |
|  | Males | | | | | | | Females | | | | | | |
| Age | L | S | -2 SD | -1 SD | 0 SD (M) | 1 SD | 2 SD | L | S | -2 SD | -1 SD | 0 SD (M) | 1 SD | 2 SD |
| 5.0 | 2.427 | 0.077 | 1.098 | 1.222 | 1.330 | 1.427 | 1.516 | 1.906 | 0.086 | 1.048 | 1.175 | 1.290 | 1.397 | 1.497 |
| 5.1 | 2.411 | 0.077 | 1.098 | 1.222 | 1.330 | 1.427 | 1.516 | 1.905 | 0.086 | 1.048 | 1.175 | 1.290 | 1.396 | 1.496 |
| 5.2 | 2.395 | 0.077 | 1.098 | 1.221 | 1.330 | 1.427 | 1.515 | 1.904 | 0.085 | 1.048 | 1.174 | 1.289 | 1.396 | 1.495 |
| 5.3 | 2.379 | 0.077 | 1.098 | 1.221 | 1.329 | 1.426 | 1.515 | 1.903 | 0.085 | 1.049 | 1.174 | 1.289 | 1.395 | 1.494 |
| 5.4 | 2.363 | 0.077 | 1.098 | 1.221 | 1.329 | 1.426 | 1.515 | 1.902 | 0.085 | 1.049 | 1.174 | 1.288 | 1.394 | 1.493 |
| 5.5 | 2.347 | 0.077 | 1.098 | 1.221 | 1.329 | 1.426 | 1.515 | 1.901 | 0.085 | 1.049 | 1.174 | 1.288 | 1.393 | 1.492 |
| 5.6 | 2.331 | 0.077 | 1.098 | 1.221 | 1.328 | 1.426 | 1.515 | 1.900 | 0.085 | 1.049 | 1.174 | 1.287 | 1.393 | 1.491 |
| 5.7 | 2.316 | 0.077 | 1.098 | 1.220 | 1.328 | 1.425 | 1.515 | 1.899 | 0.085 | 1.049 | 1.173 | 1.287 | 1.392 | 1.491 |
| 5.8 | 2.300 | 0.077 | 1.098 | 1.220 | 1.328 | 1.425 | 1.514 | 1.898 | 0.085 | 1.049 | 1.173 | 1.287 | 1.391 | 1.490 |
| 5.9 | 2.284 | 0.077 | 1.099 | 1.220 | 1.327 | 1.425 | 1.514 | 1.897 | 0.084 | 1.049 | 1.173 | 1.286 | 1.391 | 1.489 |
| 6.0 | 2.269 | 0.077 | 1.099 | 1.220 | 1.327 | 1.424 | 1.514 | 1.896 | 0.084 | 1.050 | 1.173 | 1.286 | 1.390 | 1.488 |
| 6.1 | 2.254 | 0.077 | 1.099 | 1.219 | 1.327 | 1.424 | 1.514 | 1.895 | 0.084 | 1.050 | 1.173 | 1.285 | 1.389 | 1.487 |
| 6.2 | 2.238 | 0.077 | 1.099 | 1.219 | 1.326 | 1.424 | 1.514 | 1.894 | 0.084 | 1.050 | 1.173 | 1.285 | 1.389 | 1.486 |
| 6.3 | 2.223 | 0.077 | 1.099 | 1.219 | 1.326 | 1.424 | 1.513 | 1.893 | 0.084 | 1.050 | 1.173 | 1.284 | 1.388 | 1.485 |
| 6.4 | 2.208 | 0.077 | 1.099 | 1.219 | 1.326 | 1.423 | 1.513 | 1.892 | 0.083 | 1.051 | 1.172 | 1.284 | 1.387 | 1.484 |
| 6.5 | 2.194 | 0.077 | 1.099 | 1.219 | 1.326 | 1.423 | 1.513 | 1.890 | 0.083 | 1.051 | 1.172 | 1.284 | 1.387 | 1.484 |
| 6.6 | 2.179 | 0.077 | 1.099 | 1.218 | 1.325 | 1.423 | 1.513 | 1.889 | 0.083 | 1.051 | 1.172 | 1.283 | 1.386 | 1.483 |
| 6.7 | 2.164 | 0.077 | 1.099 | 1.218 | 1.325 | 1.422 | 1.513 | 1.888 | 0.083 | 1.051 | 1.172 | 1.283 | 1.385 | 1.482 |
| 6.8 | 2.150 | 0.077 | 1.100 | 1.218 | 1.325 | 1.422 | 1.513 | 1.887 | 0.083 | 1.052 | 1.172 | 1.282 | 1.385 | 1.481 |
| 6.9 | 2.136 | 0.077 | 1.100 | 1.218 | 1.325 | 1.422 | 1.512 | 1.886 | 0.083 | 1.052 | 1.172 | 1.282 | 1.384 | 1.480 |
| 7.0 | 2.122 | 0.077 | 1.100 | 1.218 | 1.324 | 1.422 | 1.512 | 1.885 | 0.082 | 1.052 | 1.172 | 1.281 | 1.383 | 1.479 |
| 7.1 | 2.108 | 0.077 | 1.100 | 1.218 | 1.324 | 1.422 | 1.512 | 1.884 | 0.082 | 1.052 | 1.172 | 1.281 | 1.382 | 1.478 |
| 7.2 | 2.095 | 0.077 | 1.101 | 1.218 | 1.324 | 1.421 | 1.512 | 1.883 | 0.082 | 1.053 | 1.171 | 1.280 | 1.382 | 1.477 |
| 7.3 | 2.081 | 0.077 | 1.101 | 1.218 | 1.324 | 1.421 | 1.512 | 1.882 | 0.082 | 1.053 | 1.171 | 1.280 | 1.381 | 1.476 |
| 7.4 | 2.068 | 0.076 | 1.101 | 1.218 | 1.323 | 1.421 | 1.512 | 1.881 | 0.081 | 1.054 | 1.171 | 1.280 | 1.380 | 1.475 |
| 7.5 | 2.055 | 0.076 | 1.102 | 1.218 | 1.323 | 1.421 | 1.511 | 1.880 | 0.081 | 1.054 | 1.171 | 1.279 | 1.379 | 1.474 |
| 7.6 | 2.042 | 0.076 | 1.102 | 1.218 | 1.323 | 1.420 | 1.511 | 1.879 | 0.081 | 1.054 | 1.171 | 1.279 | 1.379 | 1.473 |
| 7.7 | 2.030 | 0.076 | 1.102 | 1.218 | 1.323 | 1.420 | 1.511 | 1.878 | 0.081 | 1.055 | 1.171 | 1.278 | 1.378 | 1.472 |
| 7.8 | 2.017 | 0.076 | 1.103 | 1.218 | 1.323 | 1.420 | 1.510 | 1.877 | 0.080 | 1.056 | 1.171 | 1.278 | 1.377 | 1.471 |
| 7.9 | 2.005 | 0.076 | 1.103 | 1.218 | 1.322 | 1.419 | 1.510 | 1.876 | 0.080 | 1.056 | 1.171 | 1.278 | 1.377 | 1.470 |
| 8.0 | 1.994 | 0.076 | 1.104 | 1.218 | 1.322 | 1.419 | 1.510 | 1.875 | 0.080 | 1.057 | 1.172 | 1.277 | 1.376 | 1.469 |
| 8.1 | 1.982 | 0.076 | 1.104 | 1.218 | 1.322 | 1.419 | 1.509 | 1.874 | 0.080 | 1.057 | 1.172 | 1.277 | 1.375 | 1.468 |
| 8.2 | 1.971 | 0.076 | 1.105 | 1.218 | 1.322 | 1.418 | 1.509 | 1.873 | 0.079 | 1.058 | 1.172 | 1.277 | 1.375 | 1.467 |
| 8.3 | 1.959 | 0.075 | 1.105 | 1.218 | 1.322 | 1.418 | 1.508 | 1.872 | 0.079 | 1.059 | 1.172 | 1.277 | 1.374 | 1.466 |
| 8.4 | 1.948 | 0.075 | 1.105 | 1.218 | 1.321 | 1.417 | 1.508 | 1.871 | 0.079 | 1.059 | 1.172 | 1.276 | 1.374 | 1.465 |
| 8.5 | 1.938 | 0.075 | 1.106 | 1.218 | 1.321 | 1.417 | 1.507 | 1.869 | 0.078 | 1.060 | 1.173 | 1.276 | 1.373 | 1.464 |
| 8.6 | 1.927 | 0.075 | 1.106 | 1.218 | 1.321 | 1.416 | 1.507 | 1.868 | 0.078 | 1.061 | 1.173 | 1.276 | 1.372 | 1.463 |
| 8.7 | 1.917 | 0.075 | 1.107 | 1.218 | 1.320 | 1.416 | 1.506 | 1.867 | 0.078 | 1.062 | 1.173 | 1.276 | 1.372 | 1.462 |
| 8.8 | 1.907 | 0.075 | 1.107 | 1.218 | 1.320 | 1.415 | 1.505 | 1.866 | 0.077 | 1.063 | 1.173 | 1.276 | 1.371 | 1.461 |
| 8.9 | 1.897 | 0.075 | 1.107 | 1.218 | 1.320 | 1.415 | 1.505 | 1.865 | 0.077 | 1.064 | 1.174 | 1.276 | 1.371 | 1.461 |
| 9.0 | 1.888 | 0.074 | 1.108 | 1.217 | 1.319 | 1.414 | 1.504 | 1.864 | 0.077 | 1.065 | 1.174 | 1.276 | 1.370 | 1.460 |
| 9.1 | 1.878 | 0.074 | 1.108 | 1.217 | 1.319 | 1.414 | 1.503 | 1.863 | 0.076 | 1.066 | 1.175 | 1.275 | 1.370 | 1.459 |
| 9.2 | 1.869 | 0.074 | 1.108 | 1.217 | 1.318 | 1.413 | 1.503 | 1.862 | 0.076 | 1.067 | 1.175 | 1.275 | 1.369 | 1.458 |
| 9.3 | 1.861 | 0.074 | 1.108 | 1.217 | 1.318 | 1.413 | 1.502 | 1.861 | 0.076 | 1.068 | 1.176 | 1.275 | 1.369 | 1.457 |
| 9.4 | 1.852 | 0.074 | 1.109 | 1.217 | 1.318 | 1.412 | 1.501 | 1.860 | 0.075 | 1.069 | 1.176 | 1.276 | 1.369 | 1.457 |
| 9.5 | 1.844 | 0.074 | 1.109 | 1.217 | 1.317 | 1.411 | 1.501 | 1.859 | 0.075 | 1.070 | 1.177 | 1.276 | 1.368 | 1.456 |
| 9.6 | 1.836 | 0.074 | 1.109 | 1.217 | 1.317 | 1.411 | 1.500 | 1.858 | 0.075 | 1.071 | 1.177 | 1.276 | 1.368 | 1.455 |
| 9.7 | 1.828 | 0.073 | 1.109 | 1.216 | 1.316 | 1.410 | 1.499 | 1.857 | 0.074 | 1.073 | 1.178 | 1.276 | 1.368 | 1.455 |
| 9.8 | 1.820 | 0.073 | 1.109 | 1.216 | 1.316 | 1.410 | 1.499 | 1.856 | 0.074 | 1.074 | 1.179 | 1.276 | 1.368 | 1.454 |
| 9.9 | 1.813 | 0.073 | 1.110 | 1.216 | 1.316 | 1.409 | 1.498 | 1.855 | 0.073 | 1.076 | 1.180 | 1.277 | 1.368 | 1.453 |
| 10.0 | 1.806 | 0.073 | 1.110 | 1.216 | 1.315 | 1.409 | 1.497 | 1.854 | 0.073 | 1.077 | 1.181 | 1.277 | 1.368 | 1.453 |
| 10.1 | 1.799 | 0.073 | 1.110 | 1.216 | 1.315 | 1.408 | 1.497 | 1.853 | 0.073 | 1.079 | 1.182 | 1.278 | 1.368 | 1.453 |
| 10.2 | 1.792 | 0.073 | 1.110 | 1.216 | 1.314 | 1.408 | 1.496 | 1.852 | 0.072 | 1.081 | 1.183 | 1.278 | 1.368 | 1.453 |
| 10.3 | 1.786 | 0.073 | 1.110 | 1.216 | 1.314 | 1.407 | 1.495 | 1.851 | 0.072 | 1.083 | 1.185 | 1.279 | 1.368 | 1.453 |
| 10.4 | 1.779 | 0.073 | 1.111 | 1.215 | 1.314 | 1.406 | 1.495 | 1.850 | 0.071 | 1.085 | 1.186 | 1.280 | 1.369 | 1.453 |
| 10.5 | 1.774 | 0.072 | 1.111 | 1.215 | 1.313 | 1.406 | 1.494 | 1.848 | 0.071 | 1.087 | 1.188 | 1.281 | 1.370 | 1.453 |
| 10.6 | 1.768 | 0.072 | 1.111 | 1.215 | 1.313 | 1.405 | 1.493 | 1.847 | 0.070 | 1.090 | 1.190 | 1.283 | 1.370 | 1.454 |
| 10.7 | 1.762 | 0.072 | 1.111 | 1.215 | 1.313 | 1.405 | 1.493 | 1.846 | 0.070 | 1.092 | 1.192 | 1.284 | 1.371 | 1.454 |
| 10.8 | 1.757 | 0.072 | 1.112 | 1.215 | 1.312 | 1.405 | 1.492 | 1.845 | 0.069 | 1.095 | 1.194 | 1.286 | 1.373 | 1.455 |
| 10.9 | 1.752 | 0.072 | 1.112 | 1.215 | 1.312 | 1.404 | 1.492 | 1.844 | 0.069 | 1.098 | 1.196 | 1.288 | 1.374 | 1.456 |
| 11.0 | 1.748 | 0.072 | 1.112 | 1.215 | 1.312 | 1.404 | 1.491 | 1.843 | 0.069 | 1.101 | 1.198 | 1.290 | 1.376 | 1.457 |
| 11.1 | 1.743 | 0.072 | 1.113 | 1.215 | 1.312 | 1.403 | 1.491 | 1.842 | 0.068 | 1.104 | 1.201 | 1.292 | 1.377 | 1.459 |
| 11.2 | 1.739 | 0.071 | 1.113 | 1.216 | 1.312 | 1.403 | 1.490 | 1.841 | 0.068 | 1.108 | 1.204 | 1.294 | 1.379 | 1.460 |
| 11.3 | 1.735 | 0.071 | 1.114 | 1.216 | 1.312 | 1.403 | 1.490 | 1.840 | 0.067 | 1.111 | 1.207 | 1.297 | 1.381 | 1.462 |
| 11.4 | 1.731 | 0.071 | 1.115 | 1.216 | 1.312 | 1.402 | 1.489 | 1.839 | 0.067 | 1.115 | 1.210 | 1.299 | 1.384 | 1.464 |
| 11.5 | 1.728 | 0.071 | 1.115 | 1.216 | 1.312 | 1.402 | 1.489 | 1.838 | 0.066 | 1.119 | 1.214 | 1.302 | 1.386 | 1.466 |
| 11.6 | 1.724 | 0.070 | 1.116 | 1.217 | 1.312 | 1.402 | 1.488 | 1.837 | 0.066 | 1.123 | 1.217 | 1.305 | 1.389 | 1.468 |
| 11.7 | 1.721 | 0.070 | 1.117 | 1.217 | 1.312 | 1.402 | 1.488 | 1.836 | 0.065 | 1.127 | 1.221 | 1.309 | 1.392 | 1.471 |
| 11.8 | 1.719 | 0.070 | 1.118 | 1.218 | 1.312 | 1.402 | 1.488 | 1.835 | 0.065 | 1.131 | 1.224 | 1.312 | 1.395 | 1.473 |
| 11.9 | 1.716 | 0.070 | 1.120 | 1.219 | 1.313 | 1.402 | 1.487 | 1.834 | 0.064 | 1.136 | 1.228 | 1.315 | 1.398 | 1.476 |
| 12.0 | 1.714 | 0.069 | 1.121 | 1.220 | 1.313 | 1.402 | 1.487 | 1.833 | 0.064 | 1.140 | 1.232 | 1.319 | 1.401 | 1.479 |
| 12.1 | 1.711 | 0.069 | 1.123 | 1.221 | 1.314 | 1.402 | 1.487 | 1.832 | 0.063 | 1.144 | 1.236 | 1.322 | 1.404 | 1.482 |
| 12.2 | 1.710 | 0.069 | 1.124 | 1.222 | 1.315 | 1.403 | 1.487 | 1.831 | 0.063 | 1.149 | 1.240 | 1.326 | 1.407 | 1.485 |
| 12.3 | 1.708 | 0.068 | 1.126 | 1.223 | 1.316 | 1.403 | 1.487 | 1.830 | 0.062 | 1.153 | 1.244 | 1.329 | 1.410 | 1.487 |
| 12.4 | 1.706 | 0.068 | 1.128 | 1.225 | 1.317 | 1.404 | 1.488 | 1.829 | 0.062 | 1.158 | 1.248 | 1.333 | 1.413 | 1.490 |
| 12.5 | 1.705 | 0.068 | 1.130 | 1.227 | 1.318 | 1.405 | 1.488 | 1.827 | 0.062 | 1.162 | 1.252 | 1.336 | 1.417 | 1.493 |
| 12.6 | 1.704 | 0.067 | 1.133 | 1.229 | 1.319 | 1.406 | 1.489 | 1.826 | 0.061 | 1.167 | 1.256 | 1.340 | 1.420 | 1.496 |
| 12.7 | 1.703 | 0.067 | 1.135 | 1.231 | 1.321 | 1.407 | 1.490 | 1.825 | 0.061 | 1.171 | 1.260 | 1.344 | 1.423 | 1.499 |
| 12.8 | 1.702 | 0.066 | 1.138 | 1.233 | 1.323 | 1.409 | 1.491 | 1.824 | 0.060 | 1.176 | 1.264 | 1.347 | 1.427 | 1.502 |
| 12.9 | 1.701 | 0.066 | 1.141 | 1.235 | 1.325 | 1.410 | 1.492 | 1.823 | 0.060 | 1.180 | 1.268 | 1.351 | 1.430 | 1.505 |
| 13.0 | 1.701 | 0.065 | 1.144 | 1.238 | 1.327 | 1.412 | 1.494 | 1.822 | 0.060 | 1.184 | 1.272 | 1.354 | 1.433 | 1.508 |
| 13.1 | 1.701 | 0.065 | 1.148 | 1.241 | 1.329 | 1.414 | 1.495 | 1.821 | 0.059 | 1.189 | 1.275 | 1.358 | 1.436 | 1.511 |
| 13.2 | 1.701 | 0.065 | 1.151 | 1.244 | 1.332 | 1.416 | 1.497 | 1.820 | 0.059 | 1.193 | 1.279 | 1.361 | 1.439 | 1.514 |
| 13.3 | 1.701 | 0.064 | 1.155 | 1.247 | 1.335 | 1.418 | 1.499 | 1.819 | 0.058 | 1.197 | 1.283 | 1.365 | 1.442 | 1.517 |
| 13.4 | 1.701 | 0.064 | 1.159 | 1.250 | 1.337 | 1.421 | 1.501 | 1.818 | 0.058 | 1.201 | 1.287 | 1.368 | 1.445 | 1.520 |
| 13.5 | 1.701 | 0.063 | 1.163 | 1.254 | 1.341 | 1.423 | 1.503 | 1.817 | 0.058 | 1.205 | 1.290 | 1.371 | 1.448 | 1.522 |
| 13.6 | 1.702 | 0.063 | 1.167 | 1.257 | 1.344 | 1.426 | 1.506 | 1.816 | 0.057 | 1.209 | 1.293 | 1.374 | 1.451 | 1.525 |
| 13.7 | 1.703 | 0.062 | 1.171 | 1.261 | 1.347 | 1.429 | 1.508 | 1.815 | 0.057 | 1.212 | 1.297 | 1.377 | 1.454 | 1.527 |
| 13.8 | 1.704 | 0.062 | 1.175 | 1.265 | 1.350 | 1.432 | 1.511 | 1.814 | 0.057 | 1.216 | 1.300 | 1.380 | 1.456 | 1.530 |
| 13.9 | 1.705 | 0.061 | 1.180 | 1.269 | 1.354 | 1.435 | 1.514 | 1.813 | 0.056 | 1.219 | 1.303 | 1.383 | 1.459 | 1.532 |
| 14.0 | 1.706 | 0.061 | 1.184 | 1.273 | 1.358 | 1.439 | 1.516 | 1.812 | 0.056 | 1.223 | 1.306 | 1.386 | 1.461 | 1.534 |
| 14.1 | 1.707 | 0.060 | 1.189 | 1.277 | 1.361 | 1.442 | 1.519 | 1.811 | 0.056 | 1.226 | 1.309 | 1.388 | 1.464 | 1.536 |
| 14.2 | 1.709 | 0.060 | 1.194 | 1.281 | 1.365 | 1.445 | 1.522 | 1.810 | 0.055 | 1.229 | 1.312 | 1.391 | 1.466 | 1.538 |
| 14.3 | 1.711 | 0.059 | 1.198 | 1.286 | 1.369 | 1.449 | 1.525 | 1.809 | 0.055 | 1.232 | 1.314 | 1.393 | 1.468 | 1.540 |
| 14.4 | 1.713 | 0.059 | 1.203 | 1.290 | 1.373 | 1.452 | 1.528 | 1.808 | 0.055 | 1.235 | 1.317 | 1.395 | 1.470 | 1.542 |
| 14.5 | 1.715 | 0.059 | 1.208 | 1.294 | 1.376 | 1.455 | 1.531 | 1.807 | 0.055 | 1.237 | 1.319 | 1.398 | 1.472 | 1.544 |
| 14.6 | 1.717 | 0.058 | 1.212 | 1.298 | 1.380 | 1.459 | 1.534 | 1.805 | 0.054 | 1.240 | 1.322 | 1.400 | 1.474 | 1.546 |
| 14.7 | 1.719 | 0.058 | 1.217 | 1.303 | 1.384 | 1.462 | 1.538 | 1.804 | 0.054 | 1.243 | 1.324 | 1.402 | 1.476 | 1.547 |
| 14.8 | 1.722 | 0.057 | 1.222 | 1.307 | 1.388 | 1.466 | 1.541 | 1.803 | 0.054 | 1.245 | 1.326 | 1.404 | 1.478 | 1.549 |
| 14.9 | 1.724 | 0.057 | 1.226 | 1.311 | 1.392 | 1.469 | 1.544 | 1.802 | 0.054 | 1.247 | 1.328 | 1.405 | 1.479 | 1.550 |
| 15.0 | 1.727 | 0.056 | 1.231 | 1.315 | 1.395 | 1.472 | 1.547 | 1.801 | 0.053 | 1.249 | 1.330 | 1.407 | 1.481 | 1.552 |
| 15.1 | 1.730 | 0.056 | 1.235 | 1.319 | 1.399 | 1.476 | 1.550 | 1.800 | 0.053 | 1.252 | 1.332 | 1.409 | 1.482 | 1.553 |
| 15.2 | 1.733 | 0.056 | 1.240 | 1.323 | 1.403 | 1.479 | 1.553 | 1.799 | 0.053 | 1.253 | 1.334 | 1.410 | 1.483 | 1.554 |
| 15.3 | 1.737 | 0.055 | 1.244 | 1.327 | 1.406 | 1.482 | 1.555 | 1.798 | 0.053 | 1.255 | 1.335 | 1.411 | 1.485 | 1.555 |
| 15.4 | 1.740 | 0.055 | 1.248 | 1.331 | 1.410 | 1.485 | 1.558 | 1.797 | 0.053 | 1.257 | 1.337 | 1.413 | 1.486 | 1.556 |
| 15.5 | 1.744 | 0.054 | 1.252 | 1.334 | 1.413 | 1.488 | 1.561 | 1.796 | 0.052 | 1.259 | 1.338 | 1.414 | 1.486 | 1.556 |
| 15.6 | 1.748 | 0.054 | 1.256 | 1.338 | 1.416 | 1.491 | 1.564 | 1.795 | 0.052 | 1.260 | 1.339 | 1.415 | 1.487 | 1.557 |
| 15.7 | 1.752 | 0.054 | 1.260 | 1.341 | 1.419 | 1.494 | 1.566 | 1.794 | 0.052 | 1.261 | 1.340 | 1.416 | 1.488 | 1.558 |
| 15.8 | 1.756 | 0.053 | 1.264 | 1.345 | 1.422 | 1.497 | 1.568 | 1.793 | 0.052 | 1.263 | 1.341 | 1.416 | 1.489 | 1.558 |
| 15.9 | 1.760 | 0.053 | 1.267 | 1.348 | 1.425 | 1.499 | 1.571 | 1.792 | 0.052 | 1.264 | 1.342 | 1.417 | 1.489 | 1.558 |
| 16.0 | 1.765 | 0.053 | 1.271 | 1.351 | 1.428 | 1.502 | 1.573 | 1.791 | 0.052 | 1.265 | 1.343 | 1.418 | 1.489 | 1.558 |
| 16.1 | 1.770 | 0.052 | 1.274 | 1.354 | 1.431 | 1.504 | 1.575 | 1.790 | 0.051 | 1.266 | 1.344 | 1.418 | 1.490 | 1.559 |
| 16.2 | 1.775 | 0.052 | 1.277 | 1.357 | 1.433 | 1.506 | 1.577 | 1.789 | 0.051 | 1.267 | 1.344 | 1.419 | 1.490 | 1.559 |
| 16.3 | 1.780 | 0.052 | 1.280 | 1.359 | 1.435 | 1.509 | 1.579 | 1.788 | 0.051 | 1.268 | 1.345 | 1.419 | 1.490 | 1.559 |
| 16.4 | 1.785 | 0.052 | 1.282 | 1.362 | 1.438 | 1.511 | 1.581 | 1.787 | 0.051 | 1.268 | 1.346 | 1.419 | 1.490 | 1.559 |
| 16.5 | 1.791 | 0.052 | 1.285 | 1.364 | 1.440 | 1.512 | 1.583 | 1.786 | 0.051 | 1.269 | 1.346 | 1.420 | 1.491 | 1.559 |
| 16.6 | 1.796 | 0.051 | 1.287 | 1.366 | 1.442 | 1.514 | 1.584 | 1.784 | 0.051 | 1.270 | 1.347 | 1.420 | 1.491 | 1.559 |
| 16.7 | 1.802 | 0.051 | 1.289 | 1.368 | 1.444 | 1.516 | 1.586 | 1.783 | 0.050 | 1.271 | 1.347 | 1.420 | 1.491 | 1.559 |
| 16.8 | 1.808 | 0.051 | 1.291 | 1.370 | 1.445 | 1.518 | 1.587 | 1.782 | 0.050 | 1.272 | 1.348 | 1.421 | 1.491 | 1.559 |
| 16.9 | 1.814 | 0.051 | 1.293 | 1.372 | 1.447 | 1.519 | 1.588 | 1.781 | 0.050 | 1.272 | 1.348 | 1.421 | 1.491 | 1.558 |
| 17.0 | 1.821 | 0.051 | 1.295 | 1.373 | 1.448 | 1.520 | 1.590 | 1.780 | 0.050 | 1.273 | 1.349 | 1.421 | 1.491 | 1.558 |
| 17.1 | 1.828 | 0.051 | 1.297 | 1.375 | 1.450 | 1.522 | 1.591 | 1.779 | 0.050 | 1.274 | 1.349 | 1.422 | 1.491 | 1.558 |
| 17.2 | 1.834 | 0.050 | 1.298 | 1.376 | 1.451 | 1.523 | 1.592 | 1.778 | 0.050 | 1.275 | 1.350 | 1.422 | 1.491 | 1.558 |
| 17.3 | 1.841 | 0.050 | 1.299 | 1.378 | 1.452 | 1.524 | 1.593 | 1.777 | 0.050 | 1.276 | 1.351 | 1.422 | 1.492 | 1.558 |
| 17.4 | 1.849 | 0.050 | 1.301 | 1.379 | 1.454 | 1.525 | 1.594 | 1.776 | 0.049 | 1.276 | 1.351 | 1.423 | 1.492 | 1.558 |
| 17.5 | 1.856 | 0.050 | 1.302 | 1.380 | 1.455 | 1.526 | 1.595 | 1.775 | 0.049 | 1.277 | 1.352 | 1.423 | 1.492 | 1.559 |
| 17.6 | 1.864 | 0.050 | 1.303 | 1.381 | 1.456 | 1.527 | 1.596 | 1.774 | 0.049 | 1.278 | 1.353 | 1.424 | 1.492 | 1.559 |
| 17.7 | 1.871 | 0.050 | 1.304 | 1.382 | 1.457 | 1.528 | 1.597 | 1.773 | 0.049 | 1.279 | 1.353 | 1.424 | 1.493 | 1.559 |
| 17.8 | 1.879 | 0.050 | 1.305 | 1.383 | 1.458 | 1.529 | 1.598 | 1.772 | 0.049 | 1.280 | 1.354 | 1.425 | 1.493 | 1.559 |
| 17.9 | 1.887 | 0.050 | 1.305 | 1.384 | 1.458 | 1.530 | 1.598 | 1.771 | 0.049 | 1.281 | 1.355 | 1.425 | 1.494 | 1.559 |
| 18.0 | 1.895 | 0.050 | 1.306 | 1.385 | 1.459 | 1.531 | 1.599 | 1.770 | 0.049 | 1.282 | 1.355 | 1.426 | 1.494 | 1.560 |
| 18.1 | 1.904 | 0.050 | 1.307 | 1.385 | 1.460 | 1.531 | 1.600 | 1.769 | 0.048 | 1.283 | 1.356 | 1.426 | 1.494 | 1.560 |
| 18.2 | 1.912 | 0.050 | 1.307 | 1.386 | 1.461 | 1.532 | 1.601 | 1.768 | 0.048 | 1.284 | 1.357 | 1.427 | 1.495 | 1.560 |
| 18.3 | 1.921 | 0.050 | 1.308 | 1.387 | 1.461 | 1.533 | 1.602 | 1.767 | 0.048 | 1.285 | 1.357 | 1.427 | 1.495 | 1.560 |
| 18.4 | 1.929 | 0.050 | 1.308 | 1.387 | 1.462 | 1.534 | 1.602 | 1.766 | 0.048 | 1.286 | 1.358 | 1.428 | 1.495 | 1.560 |
| 18.5 | 1.938 | 0.050 | 1.309 | 1.388 | 1.463 | 1.534 | 1.603 | 1.765 | 0.048 | 1.286 | 1.359 | 1.428 | 1.496 | 1.560 |
| 18.6 | 1.947 | 0.050 | 1.309 | 1.388 | 1.463 | 1.535 | 1.604 | 1.763 | 0.048 | 1.287 | 1.359 | 1.429 | 1.496 | 1.561 |
| 18.7 | 1.956 | 0.050 | 1.309 | 1.389 | 1.464 | 1.536 | 1.605 | 1.762 | 0.048 | 1.288 | 1.360 | 1.429 | 1.496 | 1.561 |
| 18.8 | 1.965 | 0.050 | 1.310 | 1.389 | 1.465 | 1.537 | 1.605 | 1.761 | 0.047 | 1.289 | 1.361 | 1.430 | 1.496 | 1.561 |
| 18.9 | 1.974 | 0.050 | 1.310 | 1.390 | 1.465 | 1.537 | 1.606 | 1.760 | 0.047 | 1.290 | 1.361 | 1.430 | 1.497 | 1.561 |
| 19.0 | 1.983 | 0.050 | 1.310 | 1.390 | 1.466 | 1.538 | 1.607 | 1.759 | 0.047 | 1.290 | 1.362 | 1.430 | 1.497 | 1.561 |
| 19.1 | 1.992 | 0.050 | 1.310 | 1.390 | 1.466 | 1.538 | 1.607 | 1.758 | 0.047 | 1.291 | 1.362 | 1.431 | 1.497 | 1.561 |
| 19.2 | 2.001 | 0.051 | 1.310 | 1.391 | 1.467 | 1.539 | 1.608 | 1.757 | 0.047 | 1.291 | 1.363 | 1.431 | 1.497 | 1.561 |
| 19.3 | 2.010 | 0.051 | 1.310 | 1.391 | 1.467 | 1.540 | 1.609 | 1.756 | 0.047 | 1.292 | 1.363 | 1.431 | 1.497 | 1.561 |
| 19.4 | 2.019 | 0.051 | 1.310 | 1.391 | 1.468 | 1.540 | 1.609 | 1.755 | 0.047 | 1.293 | 1.363 | 1.431 | 1.497 | 1.560 |
| 19.5 | 2.028 | 0.051 | 1.310 | 1.392 | 1.468 | 1.541 | 1.610 | 1.754 | 0.047 | 1.293 | 1.364 | 1.431 | 1.497 | 1.560 |
| 19.6 | 2.038 | 0.051 | 1.310 | 1.392 | 1.469 | 1.541 | 1.611 | 1.753 | 0.046 | 1.294 | 1.364 | 1.431 | 1.497 | 1.560 |
| 19.7 | 2.047 | 0.051 | 1.310 | 1.392 | 1.469 | 1.542 | 1.611 | 1.752 | 0.046 | 1.294 | 1.364 | 1.431 | 1.497 | 1.560 |
| 19.8 | 2.056 | 0.051 | 1.310 | 1.392 | 1.469 | 1.542 | 1.612 | 1.751 | 0.046 | 1.294 | 1.364 | 1.431 | 1.496 | 1.559 |
| 19.9 | 2.065 | 0.051 | 1.310 | 1.392 | 1.470 | 1.543 | 1.612 | 1.750 | 0.046 | 1.295 | 1.364 | 1.431 | 1.496 | 1.559 |
| 20.0 | 2.074 | 0.051 | 1.310 | 1.392 | 1.470 | 1.543 | 1.613 | 1.749 | 0.046 | 1.295 | 1.364 | 1.431 | 1.496 | 1.558 |

# **Supplemental Table 3:** Precision of other TBS Outcomes in Children 5-20 Years

| **New TBS values for TBS Insight v4.0** | | | | | | | | | | | | |
| --- | --- | --- | --- | --- | --- | --- | --- | --- | --- | --- | --- | --- |
|  | **All Ages (n=152)** | | | **Ages 6 to 9y (n=47)** | | | **Ages 10 to 13y (n=54)** | | | **Ages 14 to 16y (n=51)** | | |
| **TBS** | **RMSE** | **%CV** | **LSC** | **RMSE** | **%CV** | **LSC** | **RMSE** | **%CV** | **LSC** | **RMSE** | **%CV** | **LSC** |
| **L1 to L3** | 0.034 | 2.69 | 0.09 | 0.038 | 3.10 | 0.11 | 0.036 | 2.86 | 0.10 | 0.027 | 1.99 | 0.07 |
| **L2 to L4** | 0.037 | 2.83 | 0.10 | 0.043 | 3.42 | 0.12 | 0.039 | 3.00 | 0.11 | 0.026 | 1.87 | 0.07 |
| **L1 to L2** | 0.037 | 2.99 | 0.10 | 0.042 | 3.46 | 0.12 | 0.039 | 3.11 | 0.11 | 0.031 | 2.33 | 0.09 |
| **L2 to L3** | 0.043 | 3.32 | 0.12 | 0.051 | 3.98 | 0.14 | 0.047 | 3.61 | 0.13 | 0.030 | 2.14 | 0.08 |
| **L3 to L4** | 0.046 | 3.53 | 0.13 | 0.054 | 4.31 | 0.15 | 0.050 | 3.82 | 0.14 | 0.031 | 2.13 | 0.09 |
| **L1(L2)L3** | 0.040 | 3.21 | 0.11 | 0.043 | 3.48 | 0.12 | 0.044 | 3.53 | 0.12 | 0.033 | 2.51 | 0.09 |
| **L1(L2)(l3)L4** | 0.039 | 3.12 | 0.11 | 0.042 | 3.44 | 0.12 | 0.043 | 3.50 | 0.12 | 0.031 | 2.28 | 0.09 |
| **L2(L3)L4** | 0.036 | 2.79 | 0.10 | 0.043 | 3.45 | 0.12 | 0.036 | 2.75 | 0.10 | 0.028 | 2.05 | 0.08 |
| **L1(L2)L3L4** | 0.036 | 2.85 | 0.10 | 0.040 | 3.23 | 0.11 | 0.040 | 3.14 | 0.11 | 0.028 | 2.05 | 0.08 |
| **L1L2(L3)L4** | 0.031 | 2.40 | 0.09 | 0.035 | 2.81 | 0.10 | 0.032 | 2.49 | 0.09 | 0.025 | 1.83 | 0.07 |
| **L1** | 0.055 | 4.54 | 0.15 | 0.053 | 4.54 | 0.15 | 0.061 | 5.15 | 0.17 | 0.048 | 3.79 | 0.13 |
| **L2** | 0.052 | 4.08 | 0.14 | 0.062 | 4.95 | 0.17 | 0.051 | 4.08 | 0.14 | 0.041 | 3.08 | 0.11 |
| **L3** | 0.058 | 4.45 | 0.16 | 0.069 | 5.36 | 0.19 | 0.065 | 4.98 | 0.18 | 0.036 | 2.53 | 0.10 |
| **L4** | 0.054 | 4.15 | 0.15 | 0.065 | 5.21 | 0.18 | 0.057 | 4.36 | 0.16 | 0.035 | 2.48 | 0.10 |

%CV, percent coefficient of variation; LSC, least significant change; RMSE, root mean square error.

# **Supplemental Table 4:** Fracture Details in Children 5 to 20 Years of Age

| **Factor** |  | **n (%)** |
| --- | --- | --- |
| Did the child have an x-ray^1^? | Yes | 247 (94.6%) |
|  | No | 14 (5.4%) |
|  |  |  |
| Fracture location | Face | 13 (5.0%) |
|  | Femur | 3 (1.1%) |
|  | Foot/toe | 26 (10.0%) |
|  | Forearm/wrist/elbow | 91 (34.9%) |
|  | Hand/fingers | 70 (26.8%) |
|  | Humerus | 6 (2.3%) |
|  | Lower leg/ankle/knee | 39 (14.9%) |
|  | Other | 1 (0.4%) |
|  | Rib/clavicle | 8 (3.1%) |
|  | pelvis | 1 (0.4%) |
|  | spine | 3 (1.1%) |
|  |  |  |
| Reason for fracture | Accident | 5 (1.9%) |
|  | Childplay | 28 (10.7%) |
|  | Fell | 39 (14.9%) |
|  | Fight | 5 (1.9%) |
|  | Motor vehicle accident | 5 (1.9%) |
|  | Skating/biking/skiing/horseback | 42 (16.1%) |
|  | Sports | 127 (48.7%) |
|  | Trampoline | 8 (3.1%) |
|  | Unknown | 2 (0.8%) |
|  |  |  |
| Fracture energy | Low | 40 (15.3%) |
|  | Moderate | 184 (70.5%) |
|  | High | 35 (13.4%) |
|  | Unknown | 2 (0.8%) |

A total of 261 fractures occurred in 218 children over the 6-year follow-up period

# **Supplemental Table 5:** Associations between trabecular bone score (TBS) and incident low and moderate energy fracture risk over 6 years in children 5-20 years of age

| Cox Proportional Hazard Analysis: Prediction of Low and Moderate Impact Fractures | | | | | | | |
| --- | --- | --- | --- | --- | --- | --- | --- |
| **Model** | **Variable** | **Hazard Ratio** | **p** | **[95% CIl]** | **N Subjects** | **N Failures^2^** | **N Observations** |
| Model 1 | TBS L1-L4 Z-score | 0.98 | 0.767 | [0.84, 1.14] | 1918 | 190 | 7327 |
|  |  |  |  |  |  |  |  |
| Model 2 | TBS L1L4 Z-score | 0.98 | 0.755 | [0.85, 1.13] | 1918 | 190 | 7327 |
|  | Age group (reference ages 5 to 10 yr) |  |  |  |  |  |  |
|  | 10-14.9 yr | 3.03 | 0.000 | [2.02, 4.55] |  |  |  |
|  | 15-19.9 yr | 1.43 | 0.112 | [0.92, 2.22] |  |  |  |
|  |  |  |  |  |  |  |  |
| Model 3 | TBS L1-L4 Z-score | 1.02 | 0.971 | [0.86,1.12] | 1910 | 178 | 6946 |
|  | TS 2 | 2.53 | <0.001 | [1.52-4.22] |  |  |  |
|  | TS 3 | 2.82 | 0.001 | [1.55-5.16] |  |  |  |
|  | TS 4 | 3.34 | <0.001 | [1.06-5.41] |  |  |  |
|  | TS 5 | 1.46 | 0.093 | [0.94-2.27] |  |  |  |
|  |  |  |  |  |  |  |  |
| Model 4 | TBS L1-L4 Z-score | 0.99 | 0.912 | [0.87-1.14] | 1699 | 186 | 6811 |
|  | Growth velocity (cm/yr) | 1.11 | <0.001 | 1.06-1.16 |  |  |  |
|  |  |  |  |  |  |  |  |
| Model 5 | Spine aBMD Z-score^1^ | 0.8 | 0.012 | [0.68, 0.95] | 1902 | 190 | 7250 |
|  | TBS L1L4 Z-score | 1.06 | 0.428 | [0.92, 1.22] |  |  |  |
|  |  |  |  |  |  |  |  |
| Model 6 | Subtotal body aBMD Z-score^1^ | 0.79 | 0.007 | [0.67, 0.94] | 1898 | 183 | 7081 |
|  | TBS L1L4 Z-score | 1.06 | 0.404 | [0.92, 1.22] |  |  |  |
|  |  |  |  |  |  |  |  |
| Model 7 | Total Hip aBMD Z-score^1^ | 0.81 | 0.007 | [0.70, 0.95] | 1903 | 188 | 7230 |
|  | TBS L1L4 Z-score | 1.06 | 0.457 | [0.91, 1.24] |  |  |  |
|  |  |  |  |  |  |  |  |
| Model 8 | Femoral Neck aBMD Z-score^1^ | 0.82 | 0.010 | [0.70, 0.95] | 1903 | 188 | 7230 |
|  | TBS L1L4 Z-score | 1.05 | 0.550 | [0.89, 1.25] |  |  |  |
|  |  |  |  |  |  |  |  |
| Model 9 | Distal 1/3 Radius aBMD Z-score^1^ | 0.79 | 0.002 | [0.68, 0.91] | 1900 | 183 | 7191 |
|  | TBS L1L4 Z-score | 1.02 | 0.739 | [0.89, 1.18] |  |  |  |
|  |  |  |  |  |  |  |  |
| Model 10 | Spine BMAD Z-score | 0.79 | 0.000 | [0.69, 0.90] | 1918 | 190 | 7319 |
|  | TBS L1L4 Z-score | 1.05 | 0.389 | [0.93, 1.19] |  |  |  |
|  |  |  |  |  |  |  |  |
| Model 11 | Ultradistal Radius aBMD Z-score | 0.86 | 0.055 | [0.73, 1.00] | 1915 | 183 | 7265 |
|  | TBS L1L4 Z-score | 1.03 | 0.659 | [0.89, 1.19] |  |  |  |

^1^ Adjusted for height Z-score

^2^ failures = first occurrence of low or moderate energy fracture

Note: Baseline fracture data not available. aBMD, areal bone mineral density; BMAD, bone mineral apparent density; CI, confidence interval; yr, year.

# Supplemental Figure Legends

**Supplemental Figure 1**: Study sample derivation

Eligible lumbar spine DXA images from participants in the Bone Mineral and Density Childhood Study (BMDCS) were used to create reference data for trabecular bone score (TBS). The entire cohort (age 5 to 23.4 years) was used to generate TBS reference data (including scans of older individuals outside the desired reference range improves accuracy of the curves). Study analyses were limited to scans obtained in individuals 5-20 years (pediatric population).

**Supplemental Figure 2:** Trabecular bone score (TBS) reference curves for other combinations of lumbar spine vertebral bodies (L2-L3, L2-L4, L1-L3, L1-L2, L3-L4) for males and females 5-20 years of age. Lines represent median and 1 and 2 SD above and below the median, respectively.

**Supplemental Figure 3:** Trabecular bone score (TBS) reference curves for each individual lumbar spine vertebral body (L1, L2, L3, L4) for males and females 5-20 years of age. Lines represent median and 1 and 2 SD above and below the median, respectively.
